# Drivers of genetic diversity in secondary metabolic gene clusters within a fungal species

**DOI:** 10.1371/journal.pbio.2003583

**Published:** 2017-11-17

**Authors:** Abigail L. Lind, Jennifer H. Wisecaver, Catarina Lameiras, Philipp Wiemann, Jonathan M. Palmer, Nancy P. Keller, Fernando Rodrigues, Gustavo H. Goldman, Antonis Rokas

**Affiliations:** 1 Department of Biomedical Informatics, Vanderbilt University School of Medicine, Nashville, Tennessee, United States of America; 2 Department of Biological Sciences, Vanderbilt University, Nashville, Tennessee, United States of America; 3 Department of Microbiology, Portuguese Oncology Institute of Porto, Porto, Portugal; 4 Department of Medical Microbiology & Immunology, University of Wisconsin-Madison, Madison, Wisconsin, United States of America; 5 Center for Forest Mycology Research, Northern Research Station, US Forest Service, Madison, Wisconsin, United States of America; 6 Life and Health Sciences Research Institute (ICVS), School of Medicine, University of Minho, Braga, Portugal; 7 ICVS/3B′s - PT Government Associate Laboratory, Braga/Guimarães, Portugal; 8 Faculdade de Ciências Farmacêuticas de Ribeirão Preto, Universidade de São Paulo, São Paulo, Brazil; The Sainsbury Laboratory, UNITED KINGDOM

## Abstract

Filamentous fungi produce a diverse array of secondary metabolites (SMs) critical for defense, virulence, and communication. The metabolic pathways that produce SMs are found in contiguous gene clusters in fungal genomes, an atypical arrangement for metabolic pathways in other eukaryotes. Comparative studies of filamentous fungal species have shown that SM gene clusters are often either highly divergent or uniquely present in one or a handful of species, hampering efforts to determine the genetic basis and evolutionary drivers of SM gene cluster divergence. Here, we examined SM variation in 66 cosmopolitan strains of a single species, the opportunistic human pathogen *Aspergillus fumigatus*. Investigation of genome-wide within-species variation revealed 5 general types of variation in SM gene clusters: nonfunctional gene polymorphisms; gene gain and loss polymorphisms; whole cluster gain and loss polymorphisms; allelic polymorphisms, in which different alleles corresponded to distinct, nonhomologous clusters; and location polymorphisms, in which a cluster was found to differ in its genomic location across strains. These polymorphisms affect the function of representative *A*. *fumigatus* SM gene clusters, such as those involved in the production of gliotoxin, fumigaclavine, and helvolic acid as well as the function of clusters with undefined products. In addition to enabling the identification of polymorphisms, the detection of which requires extensive genome-wide synteny conservation (e.g., mobile gene clusters and nonhomologous cluster alleles), our approach also implicated multiple underlying genetic drivers, including point mutations, recombination, and genomic deletion and insertion events as well as horizontal gene transfer from distant fungi. Finally, most of the variants that we uncover within *A*. *fumigatus* have been previously hypothesized to contribute to SM gene cluster diversity across entire fungal classes and phyla. We suggest that the drivers of genetic diversity operating within a fungal species shown here are sufficient to explain SM cluster macroevolutionary patterns.

## Author summary

All organisms produce metabolites, which are small molecules important for growth, reproduction, and other essential functions. Some organisms, including fungi, plants, and bacteria, make specialized forms of metabolites known as “secondary” metabolites that are ecologically important and improve their producers’ chances of survival and reproduction. In fungi, the genes in pathways that synthesize secondary metabolites are typically located next to each other in the genome and organized in contiguous gene clusters. These gene clusters, along with the metabolites they produce, are highly distinct, even between otherwise similar fungi, and it is often difficult to reconstruct how these differences evolved. To understand how secondary metabolic pathways evolve in fungi, we compared secondary metabolic gene clusters in 66 strains of one species of filamentous fungus, the human pathogen *Aspergillus fumigatus*. We show that these gene clusters vary extensively within this species, and describe the genetic processes that cause these differences. We identify 5 types of variants: single nucleotide changes, gene and gene cluster gain and loss, different gene clusters at the same genomic position, and mobile gene clusters that “jump” around the genome. These results provide a road map to the types and frequencies of genomic changes underlying the extensive diversity of fungal secondary metabolites.

## Introduction

Filamentous fungi produce a diverse array of small molecules that function as toxins, antibiotics, and pigments [1]. Although by definition, these so-called specialized or secondary metabolites (SMs) are not strictly necessary for growth and development, they are critical to the lifestyle of filamentous fungi [2]. For example, antibiotic SMs give their fungal producers a competitive edge in environments crowded with other microbes [3]. SMs can additionally mediate communication between and within species as well as contribute to virulence on animal and plant hosts in pathogenic fungi [4,5].

A genomic hallmark of SMs in filamentous fungi is that the biosynthetic pathways that produce them are typically organized into contiguous gene clusters in the genome [6]. These gene clusters contain the chemical backbone synthesis genes whose enzymatic products produce a core metabolite, such as nonribosomal peptide synthases (NRPSs) and polyketide synthases (PKSs), tailoring enzymes that chemically modify the metabolite, transporters involved in product export, and, often, transcription factors that control the expression of the clustered genes [6]. These gene clusters also occasionally contain resistance genes that confer self-protection against reactive or toxic metabolites [6]. Filamentous fungal genomes, particularly those in the phylum Ascomycota [6], typically contain dozens of SM gene clusters. However, most individual SM gene clusters appear to be either species specific or narrowly taxonomically distributed in only a handful of species [6,7]. SM gene clusters that are more broadly distributed show discontinuous taxonomic distributions and are often highly divergent between species. Consequently, the identity and total number of SM gene clusters can vary widely even between very closely related species whose genomes exhibit very high sequence and synteny conservation [8,9].

In the last decade, several comparative studies have described macroevolutionary patterns of SM gene cluster diversity. For example, studies centered on genomic comparisons of closely related species have identified several different types of interspecies divergence, from single nucleotide substitutions (e.g., differences in fumonisins produced by *Fusarium* species are caused by variants in 1 gene [10]) to gene gain/loss events (e.g., the trichothecene gene clusters in *Fusarium* species and the aflatoxin family SM gene clusters in *Aspergillus* species) [11–16] and genomic rearrangements (e.g., the trichothecene gene clusters in *Fusarium*) [11]. Additionally, genetic and genomic comparisons across fungal orders and classes have identified several instances of gene gain or loss [17–19] and horizontal gene transfer (HGT) [13,20–23] acting on individual genes or on entire gene clusters, providing explanations for the diversity and discontinuity of the taxonomic distribution of certain SM gene clusters across fungal species.

Although interspecies comparative studies have substantially contributed to our understanding of SM diversity, the high levels of evolutionary divergence of SM clusters make inference of the genetic drivers of SM gene cluster evolution challenging; put simply, it has been difficult to “catch” the mechanisms that generate SM gene cluster variation “in the act.” Several previous studies have examined intraspecies or population-level differences in individual SM gene clusters, typically focusing on the presence and frequency of nonfunctional alleles of clusters involved in the production of mycotoxins. Examples of clusters exhibiting such polymorphisms include the gibberellin gene cluster in *F*. *oxysporum* [24], the fumonisin gene cluster in *F*. *fujikuroi* [25], the aflatoxin and cyclopiazonic acid gene clusters in *A*. *flavus* [26], and the bikaverin gene cluster in *Botrytis cinerea* [27]. While these studies have greatly advanced our understanding of SM gene cluster genetic variation and highlighted the importance of within-species analyses, studies examining the entirety of SM gene cluster polymorphisms within fungal species are so far lacking. We currently do not know the types and frequency of SM gene cluster polymorphisms within fungal species, whether these polymorphisms affect all types of SM gene clusters, or the genetic drivers of SM gene cluster evolution.

To address these questions, we investigated the genetic diversity of all 36 known and predicted SM gene clusters in whole genome sequence data from 66 strains, 8 of which were sequenced in this study, of the opportunistic human pathogen *A*. *fumigatus*, a species with cosmopolitan distribution and panmictic population structure [28]. We found that 13 SM gene clusters were generally conserved and harbored low amounts of variation. In contrast, the remaining 23 SM gene clusters were highly variable and contained 1 or more of 5 different types of genetic variation: single nucleotide polymorphisms (SNPs), including nonsense and frameshift variants, individual gene gain and loss polymorphisms, entire cluster gain and loss polymorphisms, polymorphisms associated with changes in cluster genomic location, and clusters with nonhomologous alleles resembling the idiomorphs of fungal mating loci. Many clusters contained interesting combinations of these types of polymorphisms, such as pseudogenization in some strains and entire cluster loss in others. The types of variants we find are likely generated by a combination of DNA replication and repair errors, recombination, genomic insertions and deletions, and horizontal transfer. We additionally find an enrichment for transposable elements (TEs) around horizontally transferred clusters, clusters that change in genomic locations, and idiomorphic clusters. Taken together, our results provide a guide to both the types of polymorphisms and the genetic drivers of SM gene cluster diversification in filamentous fungi. As most of the genetic variants that we observe have been previously associated with SM gene cluster diversity across much larger evolutionary distances and timescales, we argue that processes influencing SM gene cluster diversity within species are sufficient to explain SM cluster macroevolutionary patterns.

## Results

**Fig 1:**
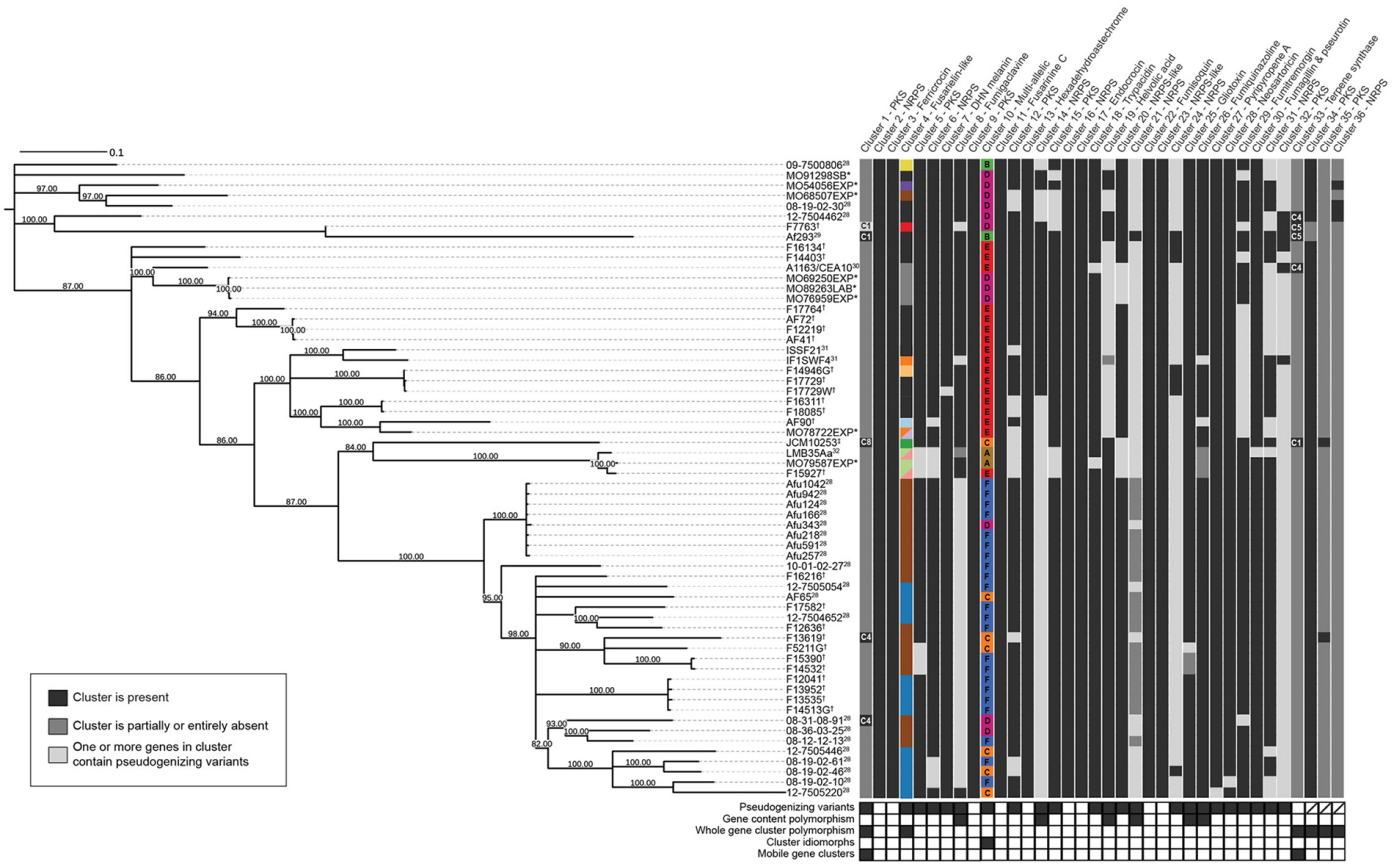
Genetic diversity of secondary metabolic gene clusters within a fungal species. The phylogeny was constructed using 15,274 biallelic SNPs with no missing data. The tree is midpoint rooted and all branches with bootstrap support less than 80% are collapsed. This phylogeny does not include strains Af10, Af210, Z5, or RP-2014, as short-read data were not available. Superfixes following strain names indicate publications associated with DNA sequencing. * indicates strains sequenced in this study, † indicates strains sequenced at JCVI with no associated publication, and ‡ indicates strains sequenced by Rikenwith no associated publication. Heat maps show presence, absence, and polymorphisms in SM gene clusters. Black indicates the cluster is present in a strain with no polymorphisms, aside from missense variants, light gray indicates 1 or more genes in the cluster are pseudogenized, and dark gray indicates the cluster is partially or entirely absent (see Fig 2). Colors for cluster 4 indicate which pseudogenizing variants are present (see Fig 3) and colors for cluster 10 indicate which allele of the cluster is present (see Fig 4). Chromosomal location of clusters 1 and 33 are indicated. If more than one type of polymorphism is present within a cluster in a strain, only 1 is depicted. Types of polymorphisms found in each cluster are summarized below the cluster heat map. DHN, dihydroxynaphthalene; JCVI, J. Craig Venter Institute; NRPS, nonribosomal peptide synthase; PKS, polyketide synthase; SM, secondary metabolite; SNP, single nucleotide polymorphism.

We analyzed the genomes of 66 globally distributed strains of *A*. *fumigatus* for polymorphisms in SM gene clusters. We performed whole genome sequencing on 8 strains and collected the remaining 58 strains from publicly available databases, including NCBI Genome and NCBI Sequence Read Archive (Fig 1, S1 Table) [28–32]. All publicly available strains of *A*. *fumigatus* with sequencing data passing quality thresholds (see Materials and methods) or with assembled genomes were included in our analysis. The resulting dataset contains strains sampled from 12 sites worldwide and from clinical and environmental sources (S1 Table).

We analyzed all strains for polymorphisms in 33 curated SM gene clusters present in the reference Af293 genome and additionally searched for novel SM gene clusters (see Materials and methods). These examinations revealed 5 distinct types of polymorphisms in SM gene clusters (Fig 1, Table 1):

1. SNPs and short indel polymorphisms. Thirty-three of 33 SM gene clusters (present in the reference Af293 strain) contained multiple genes with missense SNPs and short indel variants in 1 or more strains. Twenty-three of 33 SM gene clusters contained 1 or more genes with frameshift or nonsense variants.
2. Gene content polymorphisms involving loss or gain of 1 or more genes. Six of 33 SM gene clusters contained a gene content polymorphism.
3. Whole SM gene cluster gain and loss polymorphisms. Three of 33 SM gene clusters were entirely absent in 1 or more strains and an additional 3 previously unknown SM gene clusters were discovered.
4. Idiomorphic polymorphisms. One locus contained multiple nonhomologous SM gene cluster alleles in different strains.
5. Genomic location polymorphisms. Two of 33 SM gene clusters were found on different chromosomes between strains.

**Table 1:** Types and rates of SM gene cluster variants in *Aspergillus fumigatus* strains.

Description	Phenotype	Drivers	Frequency at cluster level	Frequency at strain level	Previous reports
Single-nucleotide polymorphisms and indels	Potential for protein function change (missense); abrogation of protein function (nonsense and frameshift)	DNA replication errors; relaxation of purifying selection	100% (33/33 clusters; missense); 70% (23/33 clusters; nonsense and frameshift)	Every strain affected	Bikaverin in *Botrytis* [17,27], aflatoxin in *A*. *oryzae* and *A*. *flavus* [26], fumonisins in *Fusarium* [10], many others
Gene content polymorphisms	Loss of gene cluster function; structural changes in the metabolite; change in cluster expression or metabolite transport	Deletion and insertion events; recombination; transposable elements	6 clusters	27/66 strains	Trichothecene in *Fusarium*, aflatoxin and sterigmatocyst in *Aspergillus* [11–15], HC toxin in *Cochliobolus carbonarum* [33]
Whole gene cluster polymorphisms	Loss or gain of novel metabolites	Deletion and insertion events; horizontal gene transfer; transposable elements	6 clusters	13/66 strains	Gibberellin and fumonisin in *Fusarium* [24,25]
Cluster idiomorphs	Changes in metabolites produced or structure of metabolites	Transposable elements; recombination; other mechanisms?	1 gene cluster	8 unique identified alleles	Putative SM gene clusters in dermatophytes; putative SM gene cluster in *A*. *flavus* and *A*. *oryzae* [34,35]
Mobile gene clusters	Potential for change in gene regulation	Transposable elements; horizontal gene transfer; other mechanisms?	2 gene clusters	8/66 strains	None

**Abbreviation:** SM; secondary metabolite.

Both genomic location polymorphisms and idiomorphic polymorphisms are novel types of variants that have not been previously described for secondary metabolic gene clusters, likely because they can only be identified when genome-wide synteny and sequence conservation are high. The remaining types of variants, including single-nucleotide changes and gene gain and loss events, have been implicated at the species level as major drivers of secondary metabolic gene cluster evolution (Table 1), suggesting that the diversity-generating processes observed within a species are sufficient to explain SM gene cluster evolution across species.

### SNPs and indel polymorphisms

It is well established that SNPs and short indel polymorphisms, which are caused by errors in DNA replication and repair, are a major source of genomic variation [36]. Nonsynonymous SNPs and indels with missense, frameshift, and nonsense effects were widespread across the 33 SM reference gene clusters (Fig 1, S2 Table). Every strain contained numerous missense mutations and at least 1 nonsense or frameshift mutation in its SM gene clusters. Although missense mutations are likely to influence SM production, the functional effects of nonsense and frameshift mutations are comparatively easier to infer from genomic sequence data because they often result in truncated proteins and loss of protein function.

SNPs and short indel polymorphisms can affect secondary metabolite production, as in the case of the lack of trypacidin production in the A1163 strain because of a previously identified frameshift mutation in the PKS of the trypacidin gene cluster [37]. Interestingly, we identified a premature stop codon (Gln273*) in a transcription factor required for trypacidin production, *tpcD* (Afu4g14550), in a strain sequenced in this study (MO79587EXP) (S2 Table). These data suggest that function of this SM gene cluster has been lost at least twice, independently, in *A*. *fumigatus*.

Individual nonsense or frameshift variants varied in frequency. For example, the NRPS *pes3* gene (Afu5g12730) in SM gene cluster 21 harbors 16 nonsense or frameshift polymorphisms in 55 strains, 7 of which are common (present in ≥10 strains) and another 7 of which are rare (≤5 strains). Strains with lab-mutated null alleles of the *pes3* gene are more virulent than strains with functional copies [38], which may explain the widespread occurrence of null *pes3* alleles within *A*. *fumigatus*.

### Gene content polymorphisms

We additionally identified several SM gene clusters that gained or lost genes in some strains. These gene content polymorphisms were most likely generated through genomic deletion or insertion events and were sometimes found at high frequencies among strains (Fig 1, Table 1). In 3 cases, these polymorphisms impacted backbone synthesis genes, rendering the SM gene cluster nonfunctional.

**Fig 2:**
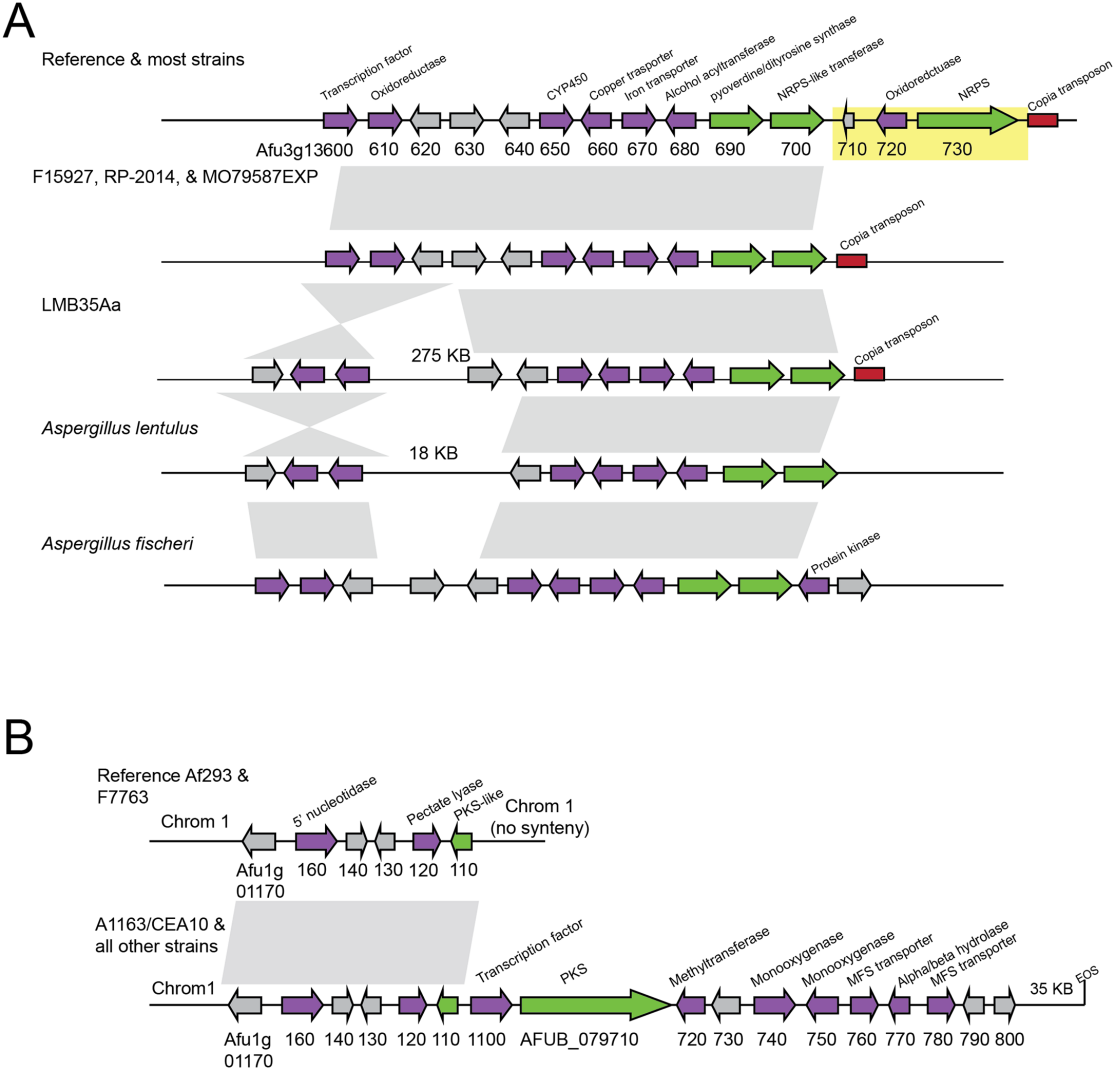
Gene gains and deletions in SM gene clusters. (A) Differences in gene content in SM gene cluster 14 in *Aspergillus fumigatus* strains and closely related species. Four *A*. *fumigatus* strains lack an 11-kb region in this cluster, including an NRPS backbone gene, highlighted in yellow. Regions upstream and downstream of this cluster are syntenic. LMB35Aa also contains a large inversion that moves a transcription factor, oxidoreductase, and hypothetical protein 275 kb away from the cluster. *A*. *fischeri* and *A*. *lentulus*, close relatives of *A*. *fumigatus*, contain a cluster lacking the 11-kb region. (B) SM gene cluster found in most *A*. *fumigatus* strains but absent from the Af293 reference and from the F7763 strain. EOS, end of scaffold; MFS, major facilitator superfamily; NRPS, nonribosomal peptide synthase; SM, secondary metabolite.

One example involves SM gene cluster 14, whose standard composition includes a pyoverdine synthase gene, an NRPS-like gene, an NRPS backbone gene, and several additional modification genes (Fig 2A). Four of the 66 strains examined lack an 11-kb region on the 3′ end of the cluster, which normally contains an NRPS gene and 2 additional cluster genes, and the first non-SM genes on the 3′ end flanking the cluster. All *A*. *fumigatus* strains contain a *copia* family TE [39,40] at the 3′ end of the cluster, suggesting that TEs may have been involved in the generation of this polymorphism. While this polymorphism could have arisen through a deletion event, a homologous cluster lacking the 11-kb region is also present in the reference genomes of *A*. *lentulus* and *A*. *fischeri*, close relatives of *A*. *fumigatus* (Fig 2A). The most parsimonious explanation is that the genome of the *A*. *fumigatus* ancestor contained an SM gene cluster that lacked the 11-kb region and that this genomic region was subsequently gained and increased in frequency within *A*. *fumigatus*.

The remaining 2 gene content polymorphisms affecting SM backbone genes were restricted to 1 strain each and appear to have arisen through genomic deletion events. Specifically, strain IF1SWF4 lacks an 8-kb region near the helvolic acid SM gene cluster, resulting in the loss of the backbone oxidosqualene cyclase gene as well an upstream region containing 2 non-SM genes (S1 Fig). Strain LMB35Aa lacks a 54-kb region on the end of chromosome 2, which includes 5 genes from the telomere-proximal fumigaclavine C cluster (S1 Fig).

Three other cases of gene content polymorphisms involved gene loss or truncation events of non-backbone structural genes. The second half of the open reading frame (ORF) of the *gliM O*-methyltransferase gene in the gliotoxin gene cluster has been lost in 2 of 66 strains (S1 Fig) and the first half of the permease *fmqE* in the fumiquinazoline gene cluster has been lost in 4 of 66 strains (S1 Fig). Finally, an ATP-binding cassette (ABC) transporter gene in SM cluster 21 has been almost entirely lost in 21 of 66 strains (S1 Fig). This deletion event is found in strains that are related in the SNP-based strain phylogeny but does not perfectly mirror the phylogeny (Fig 1).

### Whole gene cluster loss polymorphisms

Several SM gene clusters were gained or lost entirely across strains. We observed several instances in which a cluster present in the genome of either the reference Af293 or A1163 (also known as CEA10) strain was absent or pseudogenized in other strains, which we present in this section.

One of the novel SM gene clusters, cluster 34, was present in all but 2 of the strains (Af293 and F7763). Cluster 34 contains a PKS backbone gene, 1 PKS-like gene with a single PKS-associated domain, 9 genes with putative biosynthetic functions involved in secondary metabolism, and 6 hypothetical proteins (Fig 2B). The 2 strains that lack cluster 34 contain a likely nonfunctional cluster fragment that includes the PKS-like gene, 2 biosynthetic genes, and 3 hypothetical proteins. Interestingly, the 3′ region flanking cluster 34 is syntenic across all 66 strains but the 5′ region is not, suggesting that a recombination or deletion event may have resulted in its loss in the Af293 and F7763 strains. These 2 strains form a clade in the strain phylogeny (Fig 1), so it is likely that this deletion or recombination event occurred once.

One notable example of an SM gene cluster present in the Af293 reference genome but absent or pseudogenized in others was SM cluster 4. This cluster contains 5 genes on the tip of the Af293 chromosome 1 and contains orthologs to 5 of the 6 genes in the fusarielin-producing gene cluster in *F*. *graminearum* [41]. Cluster 4 is also present in several other *Aspergillus* species, including *A*. *clavatus* and *A*. *niger* [41], as well as in whole or in part in other non-*Aspergillus* fungi in the class Eurotiomycetes and in fungi in the class Sordariomycetes (S3 Fig) [30,42–50]. Phylogenetic analysis of the genes in cluster 4 does not provide a clear view of the origin of this cluster, which is consistent either with extensive gene loss in both Sordariomycetes and Eurotiomycetes or, alternatively, with HGT between fungi belonging to the 2 classes (S2 and S3 Figs).

**Fig 3:**
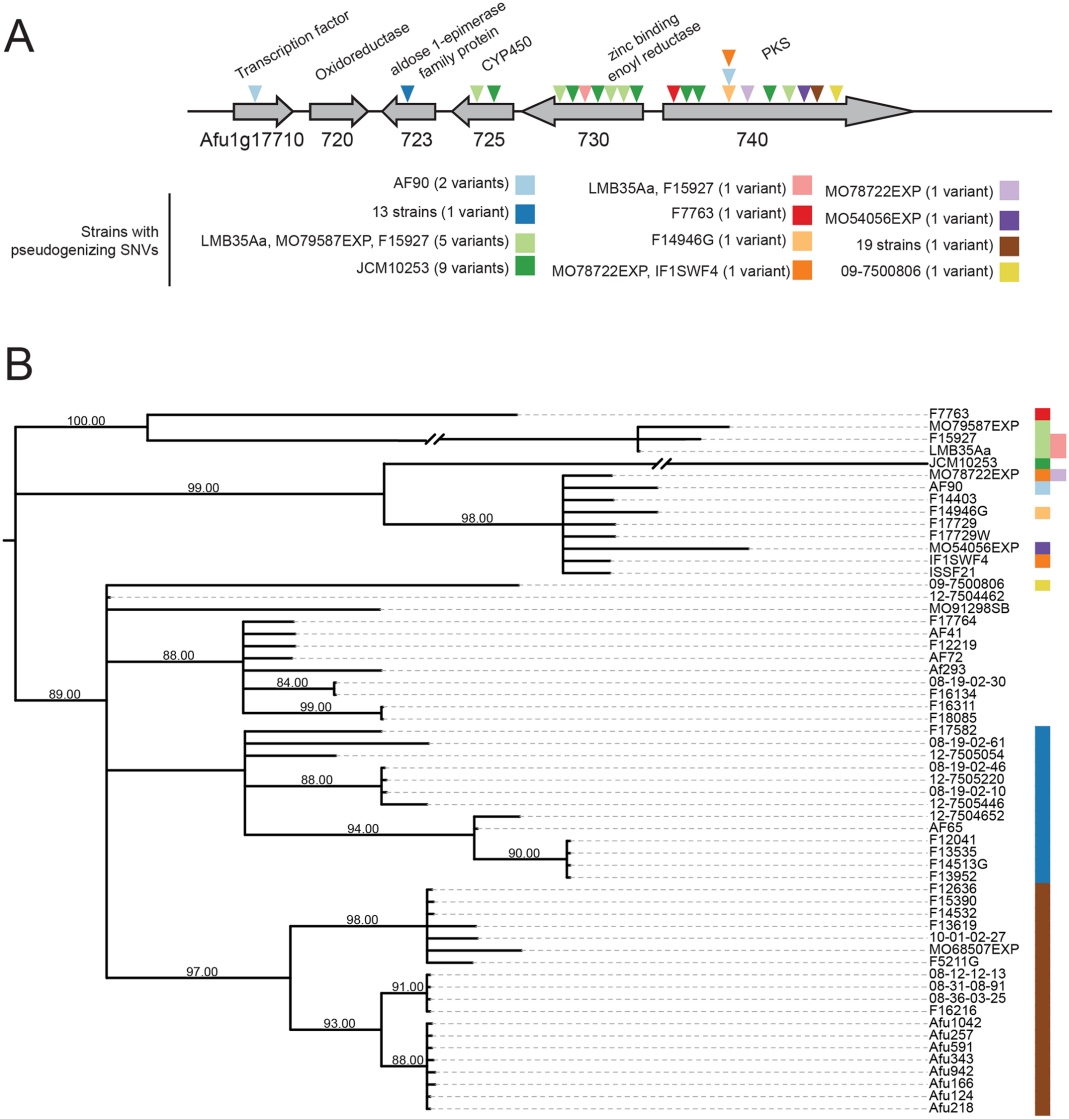
Pseudogenization in the fusarielin-like SM gene cluster. (A) Positions of frameshift variants and nonsense variants in the fusarielin-like SM gene cluster 4. (B) Locus phylogeny of the fusarielin-like SM gene cluster based on a nucleotide alignment of the entire gene cluster, including intergenic and noncoding regions. The phylogeny is midpoint rooted and branches with bootstrap support <80% are collapsed. Two branches were shortened for visualization purposes. Strains with pseudogenizing variants are indicated with colored boxes. Colors correspond to variants shown in (A). PKS, polyketide synthase; SM, secondary metabolite; SNV, single nucleotide variant.

Cluster 4 is entirely absent in 4 of 66 strains, and its genes are undergoing pseudogenization in an additional 43 strains via multiple independent mutational events (Fig 3). The 4 strains lacking the cluster form a single clade on the strain phylogeny, suggesting that the cluster was lost in a single deletion event (Fig 1). Further, 19 strains shared a single frameshift variant in the PKS gene (4380_4381insAATGGGCT; frameshift at Glu1461 in Afu1g17740) and an additional 13 strains shared a single frameshift variant (242delG; frameshift at Gly81) in an aldose 1-epimerase gene (Afu1g17723) (Fig 3A, S2 Table). Eleven other strains each contained 1 to several frameshift or nonsense polymorphisms involving 9 unique mutational sites. Five of these strains contained multiple distinct frameshifts and premature stop codons in more than 1 gene in the cluster, indicating that the entire pathway is pseudogenized in these strains.

A phylogeny of the entire cluster 4 locus across all 62 strains with short-read data shows that 2 pseudogenizing variants shared across multiple strains, one in the aldose 1-epimerase gene and one in the PKS, are found in loci that form well-supported clades (Fig 3B), suggesting that these variants arose once. Similarly, a set of variants shared across 3 strains and 1 variant shared in 2 strains are found in loci that form well-supported clades in the locus phylogeny. Two strains sharing a pseudogenizing variant in the PKS do not group together in the locus phylogeny, a discordance likely stemming from within-locus recombination events. Finally, functional alleles of cluster 4 are distributed throughout the locus phylogeny, suggesting that the functional allele is ancestral and the pseudogenized variants are derived.

Perhaps surprisingly, loss-of-function polymorphisms (from nonsense and frameshift mutations to wholesale cluster loss) are common and sometimes frequent within *A*. *fumigatus*. The majority of these polymorphisms are presumably neutral and reflect the fact that any mutation is more likely to result in loss of a function than in gain. Consistent with this hypothesis is our observation that these loss events were often found at low frequencies. However, the possibility also exists that some of the high-frequency, recurrent loss-of-function polymorphisms may be adaptive. Given that many secondary metabolites are primarily secreted in the extracellular environment and can benefit nearby conspecifics that are not themselves producing the metabolite [51], individual strains may be circumventing the energetically costly process of producing the metabolite themselves in a situation analogous to the Black Queen Hypothesis [52].

### Whole gene cluster gain polymorphisms

By searching for novel SM gene clusters in the genomes of the other 65 *A*. *fumigatus* strains, we found 3 SM gene clusters that were absent from the genome of the Af293 reference strain. As SM gene clusters are often present in repeat-rich and subtelomeric regions that are challenging to assemble [53,54], the strains analyzed here might harbor additional novel SM gene clusters that were not captured here.

One of these SM gene clusters, cluster 34, was mentioned earlier as an example of whole gene cluster loss polymorphism (Fig 2B) and is present in most strains but has been lost in 2 strains. The other 2 SM gene clusters absent from the Af293 genome are present at lower frequencies and likely reflect gene cluster gain events; cluster 35 is present in 2 of 66 strains and cluster 36 in 4 of 66 strains. Cluster 35 is located in a region syntenic with an Af293 chromosome 4 region and is flanked on both sides by TEs (S4 Fig). Eight of the 14 genes in this SM gene cluster are homologous to genes in an SM gene cluster in the genome of the insect pathogenic fungus *Metarhizium anisopliae* (S4 Fig) [55]. Phylogenetic analysis of these 8 genes is consistent with a horizontal transfer event (S5 Fig). The 2 strains that contain this novel cluster are not sister to each other on the strain phylogeny (Fig 1).

Cluster 36 is an NRPS-containing cluster located on shorter genomic scaffolds that lack homology to either the Af293 or A1163 genomes, making it impossible to determine on which chromosome this cluster is located (S4 Fig). Two of the strains containing this novel cluster are sister to each other on the strain phylogeny, while the third is distantly related to these 2 (Fig 1). The evolutionary histories of the genes in the cluster are consistent with vertical inheritance, and these genes are present in multiple *Aspergillus* species.

### Idiomorph polymorphisms

**Fig 4:**
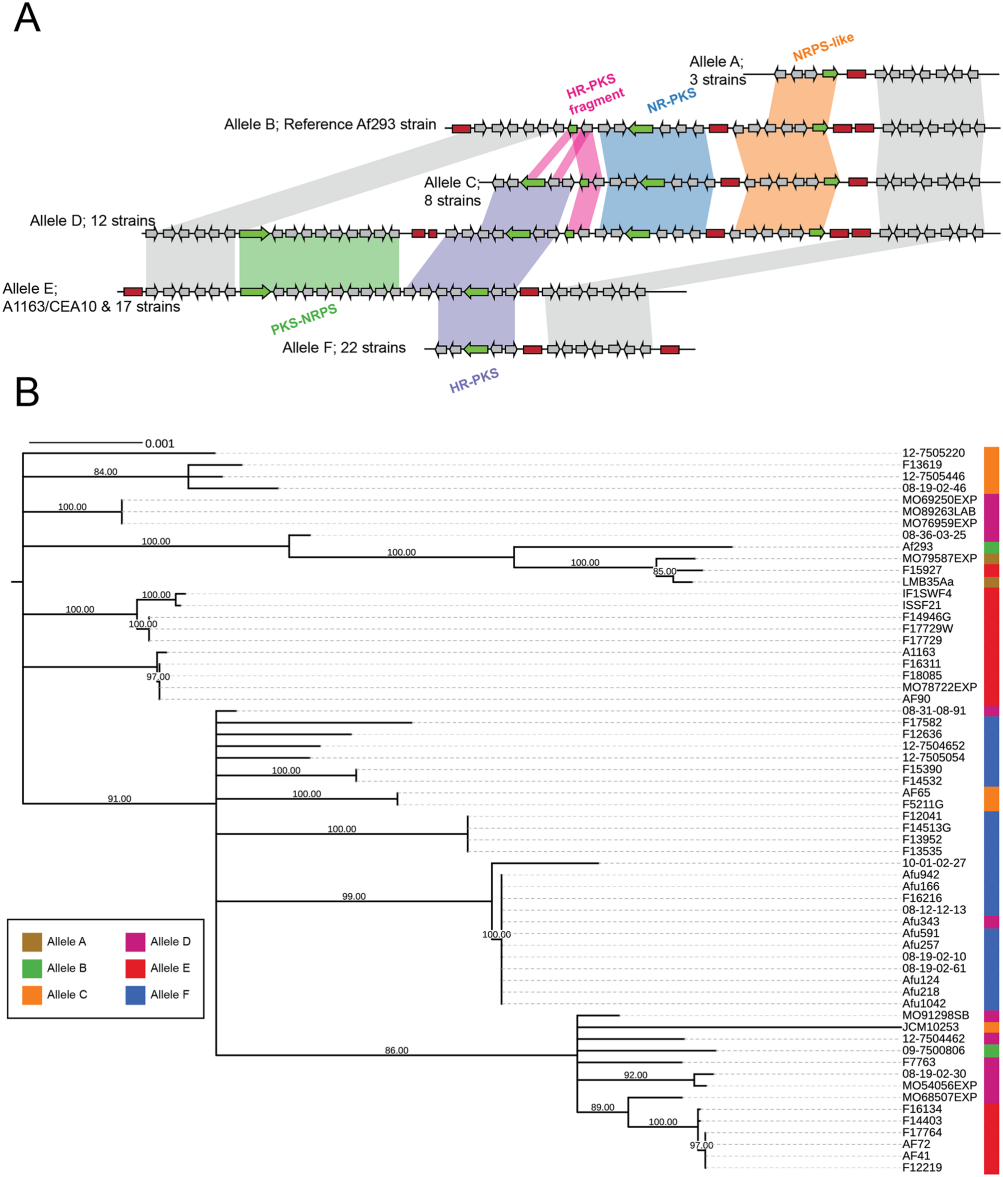
Six alleles of an idiomorphic SM gene cluster. (A) Alleles of SM gene cluster 10 on chromosome 3. Red boxes denote transposable elements. Green arrows denote backbone genes (PKS or NRPS). (B) Locus phylogeny of conserved downstream of the idiomorph cluster (highlighted in gray in [A]). Phylogeny was constructed using a 48-mb nucleotide alignment with the GTRGAMMA model and midpoint rooted. Branches with bootstrap support <80% were collapsed. HR, highly reducing; NR, nonreducing; NRPS, nonribosomal peptide synthase; PKS, polyketide synthase; SM, secondary metabolite.

One of the most peculiar types of polymorphisms that we identified is a locus containing different unrelated alleles of SM gene clusters, reminiscent of the idiomorph alleles at the fungal mating loci [56]. This locus, which resides on chromosome 3 and corresponds to cluster 10 in the Af293 genome (Fig 4), was previously described as being strain specific in a comparison between Af293 and A1163 strains [30] and is thought to reside in a recombination hot spot [28]. Our analysis showed that there are at least 6 different alleles of this cluster in *A*. *fumigatus* containing 4 different types of key enzymes involved in natural product biosynthesis: a PKS-NRPS hybrid, a highly reducing (HR) PKS, a nonreducing (NR) PKS, and an NRPS-like enzyme (Fig 4). Two additional alleles were present in only 1 strain each (S6 Fig).

In the Af293 reference genome, the cluster present at the idiomorph locus contains 1 NR-PKS along with an NRPS-like gene (allele B). In the A1163 reference genome and 17 other strains, there is a PKS-NRPS and an HR-NRPS at this locus (allele E). These alleles show an almost complete lack of sequence similarity except for a conserved hypothetical protein and a fragment of the HR-PKS in the Af293 allele; in contrast, the upstream and downstream flanking regions of the 2 alleles, which do not contain any backbone genes, are syntenic. Remarkably, another allele, present in 12 strains, contains all of the genes from both the Af293 and A1163 clusters (allele D). The remaining 3 alleles contain various combinations of these genes. One allele found in 22 strains contains some A1163-specific genes, including the HR-PKS, and no Af293-specific genes (allele F), while another allele found in 3 strains contains some Af293-specific genes, including the NRPS-like gene, but no A1163 genes (allele A). The final allele, present in 8 strains, contains the entire Af293 allele as well as part of the A1163 allele containing the HR-PKS (allele C). Every allele is littered with multiple long terminal repeat sequence fragments from *gypsy* and *copia* TE families as well as with sequence fragments from DNA transposons from the *mariner* family [39]. In some cases, these TEs correspond with break points in synteny between alleles, suggesting that the diverse alleles of this SM gene cluster may have arisen via TE-driven recombination. Furthermore, both of the alleles that are restricted to a single strain have an insertion event of several genes near a TE, while the rest of the locus is highly similar to one of the more common alleles (S6 Fig).

Untargeted XCMS analysis [57] of an allele D strain (08-19-02-30) and 2 allele F strains (08-12-12-13 and 08-19-02-10) and comparison of their metabolite profiles revealed the presence of 2 unique masses in 08-19-02-30 (S4 Table; S7 Fig), raising the possibility that variation at the idiomorph locus is functional. Further analysis is underway to investigate whether any of these mass to charge ratios can be directly linked to the allele D sequence.

To gain insight into the evolutionary history of this locus, we constructed a phylogeny based on its conserved downstream flanking region (Fig 4B). The resulting phylogeny shows some grouping of strains that share alleles, but there are no clades that contain all instances of a particular allele. This is likely to be the consequence of within-locus recombination between strains of *A*. *fumigatus*, which has been previously described at this locus [28] and which is potentially driven by the high number of repetitive sequences at this locus.

While it is tempting to speculate that allele D, the longest allele containing all observed genes, represents the ancestral state, this does not explain the presence of a shared hypothetical protein and PKS gene fragment between allele C and allele B. Furthermore, 2 close relatives of *A*. *fumigatus*, *A*. *lentulus* and *A*. *fischeri*, contain a similar region with conserved upstream and downstream flanking genes that is highly dissimilar to any of the alleles observed in *A*. *fumigatus* (S8 Fig). In both species, this locus contains numerous TEs as well as genes homologous to portions of allele E in *A*. *fumigatus* (S8 Fig). *A*. *fischeri* additionally contains 2 hypothetical proteins from the PKS-NRPS region of *A*. *fumigatus* and an additional hybrid PKS-NRPS-containing gene cluster not found in either *A*. *lentulus* or any *A*. *fumigatus* strain (S8 Fig). Other genes at this locus in both *A*. *lentulus* and *A*. *fischeri* have functions likely not related to SM. Interestingly, *A*. *lentulus* contains a gene with a heterokaryon incompatibility protein domain, which may be involved in determining vegetative incompatibility [58]. Only 1 representative genome from each species has been sequenced, but based on the high concentration of TEs and lack of sequence similarity with any *A*. *fumigatus* alleles, it is likely that this locus is highly variable within both *A*. *lentulus* and *A*. *fischeri*.

It is possible that polymorphism at this locus originated via SM gene cluster fusion or fission events driven by TEs, which are present in large numbers. Interestingly, 2 other previously described instances of SM gene cluster variation bear some resemblance to the *A*. *fumigatus* idiomorphic SM gene cluster 10 locus. The first is the presence of 2 nonhomologous *A*. *flavus* alleles, for which some strains contain a 9-gene sesquiterpene-like SM gene cluster and others contain a nonhomologous 6-gene SM gene cluster at the same genomic location [35]. The second is the presence of 2 nonhomologous SM gene clusters at the same well-conserved locus in a comparison of 6 species of dermatophyte fungi [34]. Based on these results, we hypothesize that idiomorphic clusters may be common in fungal populations and contribute to the broad diversity of SM gene clusters across filamentous fungi.

### Genomic location polymorphisms

The final type of polymorphism that we observed is associated with SM gene clusters that are found in different genomic locations in different strains, suggesting that these SM gene clusters are behaving like mobile genetic elements. This type of polymorphism was observed in SM gene clusters 1 and 33, both of which produce as-yet-identified products and are present at low frequencies in *A*. *fumigatus* strains.

**Fig 5:**
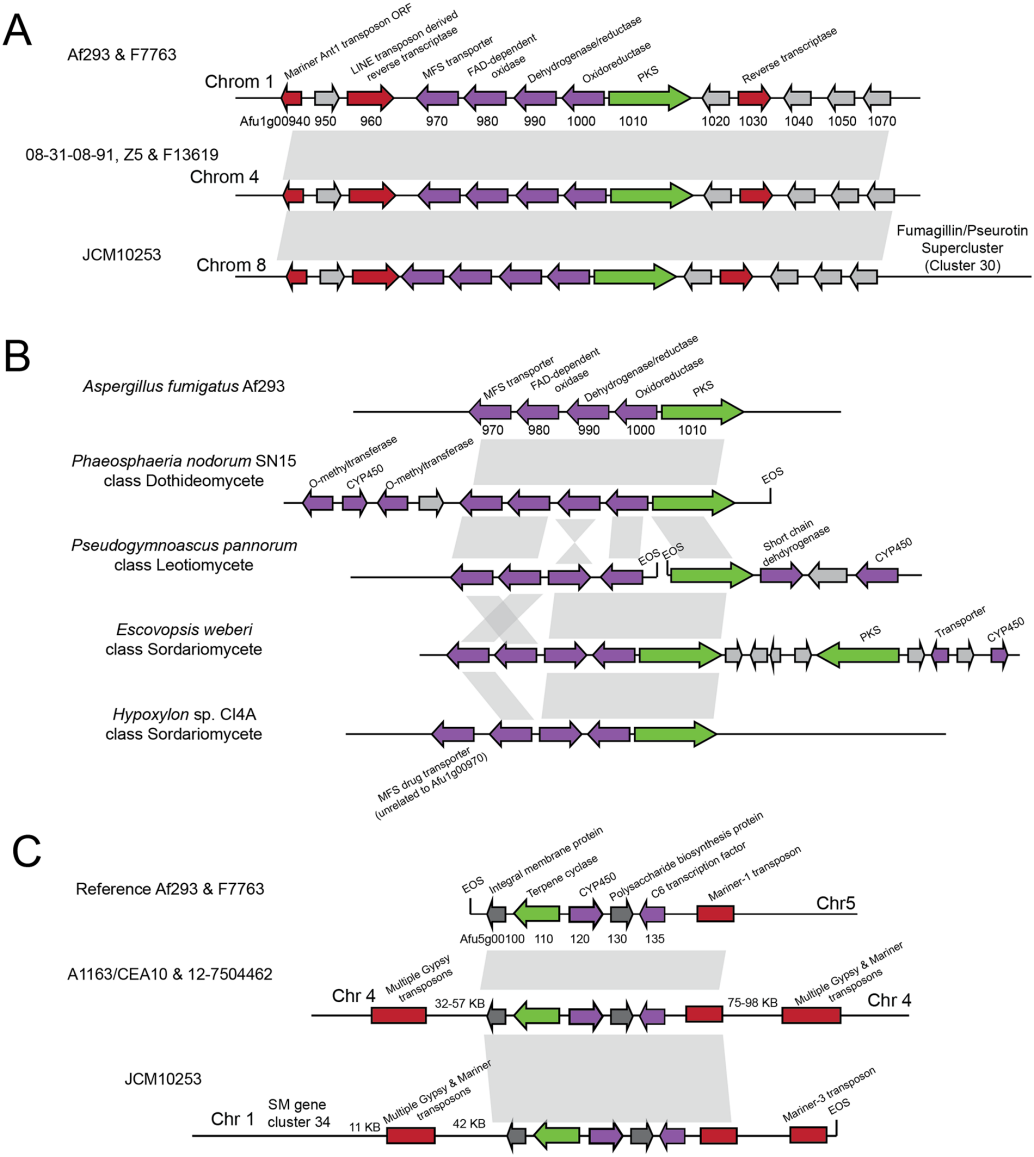
Multiple genomic locations of 2 SM gene clusters. (A) SM gene cluster 1 (Afu1g00970-01010) and flanking region are found in different genomic locations. The flanking regions contain transposon-derived open reading frames, including 2 putative reverse transcriptases. In one strain, SM gene cluster 1 is found adjacent to SM gene cluster 30. (B) Synteny of *A*. *fumigatus* SM gene cluster 1 with clusters in *Phaeosphaeria nodorum*, *Pseudogymnoascus pannorum*, *Escovopsis weberi*, and *Hypoxylon* sp. CI4A. All species contain nonsyntenic genes predicted by antiSMASH to be part of a biosynthetic gene cluster. (C) SM gene cluster 33 (Afu5g00100-00135) is found in different genomic locations in different strains. In one strain, the cluster is adjacent to SM gene cluster 34. Multiple transposable elements flank the cluster in each strain. EOS, end of scaffold; FAD, flavin adenine dinucleotide; MFS, major facilitator superfamily; ORF, open reading frame; PKS, polyketide synthase; SM, secondary metabolite.

SM gene cluster 1, which is present in 6 strains at 3 different genomic locations (Fig 5A), consists of a PKS and 4 other structural genes that are always flanked by a 15-kb region (upstream) and a 43-kb region (downstream) containing TEs. In the reference Af293 strain and in strain F7763, cluster 1 and its flanking regions are located on chromosome 1, while in strains 08-31-08-91, F13619, and Z5 they are located between Afu4g07320 and Afu4g07340 on chromosome 4. In contrast, in strain JCM10253, the cluster and flanking regions are located on chromosome 8 immediately adjacent to the 3′ end of the intertwined fumagillin and pseurotin SM gene supercluster [59]. The strains containing the allele on chromosome 1 are sister to each other on the strain phylogeny, while the other strains are scattered across the tree and do not reflect the phylogeny (Fig 1).

In 5 of 6 strains, cluster 1 appears to be functional and does not contain nonsense SNPs or indels. However, the cluster found on chromosome 1 in strain F7763 contains 2 stop codons in the oxidoreductase gene (Gln121* and Gln220*) and 2 premature stop codons in the PKS (Gln1156* and Gln1542*), suggesting this strain contains a null allele.

This “jumping” gene cluster is not present in any other sequenced genome in the genus *Aspergillus*, and phylogenetic analysis of its constituent genes is consistent with HGT between fungi (S9 Fig). Specifically, this gene cluster is also present in *Phaeosphaeria nodorum* [60], a plant pathogen from the class Dothideomycetes, *Pseudogymnoascus pannorum* [61], a fungus isolated from permafrost from the Leotiomycetes, and *Escovopsis weberi* [62], a fungal parasite of fungus-growing ants from the Sordariomycetes (Fig 5B). One additional species, the endophyte *Hypoxylon* sp. CI4A from the class Sordariomycetes [63], contains 4 of the 5 cluster genes but is missing Afu1g00970, an MFS drug transporter. However, this species contains a gene unrelated to Afu1g00970 that is annotated as an MFS drug transporter immediately adjacent to this cluster (Fig 5B). None of these fungi contain the upstream or downstream TE-rich flanking regions present in *A*. *fumigatus*, and each fungus contains additional unique genes with putative biosynthetic functions adjacent to the transferred cluster. The most likely explanation for this change in flanking regions is that this SM gene cluster was transferred into *A*. *fumigatus* once and has subsequently “jumped” in different genomic locations in different strains.

The second SM gene cluster that shows variation in its genomic location across strains, cluster 33, contains a terpene synthase. This cluster is present in only 5 strains at 3 distinct locations (Fig 5C). Similar to cluster 1, cluster 33 is also flanked by TEs, and in 1 strain the cluster is located in a new region 58 Kb from SM gene cluster 34. Two strains that contain the cluster in the same genomic location are sister to each other on the strain phylogeny, while the placement of the other 3 strains containing the cluster does not reflect the phylogeny (Fig 1). In contrast to cluster 1, cluster 33 does not appear to have been horizontally transferred between fungi and its genes are present in other sequenced *Aspergillus* species [64], suggesting that the mobility of clusters 1 and 33 may be driven by different mechanisms.

Interestingly, both cases of mobile gene clusters are located near or immediately adjacent to other SM gene clusters in some strains. Cluster 33 is located 58 kb away from cluster 34 in one strain, and cluster 1 is located immediately adjacent to the intertwined fumagillin and pseurotin supercluster [59] in another. This supercluster is regulated by the transcriptional factor *fapR* (Afu8g00420) and is located in a chromosomal region controlled by the master SM regulators *laeA* (Afu1g14660) and *veA* (Afu1g12490) [59,65], raising the hypothesis that mobile gene clusters might be co-opting the regulatory machinery acting on adjacent SM gene clusters. Previous work has hypothesized that the fumagillin and pseurotin supercluster formed through genomic rearrangement events, placing the once-independent gene clusters in close proximity to each other [59]. Our observation that the mobile cluster 1 is located in this same region not only supports this hypothesis but also implicates TEs as one of the mechanisms by which superclusters are formed. These superclusters may also represent an intermediate stage in the formation of new SM gene clusters. Supercluster formation, potentially mediated by mobile gene clusters and followed by gene loss, could explain macroevolutionary patterns of SM gene clusters in which clustered genes in one species are found to be dispersed over multiple gene clusters in other species [9,11].

## Discussion

**Fig 6:**
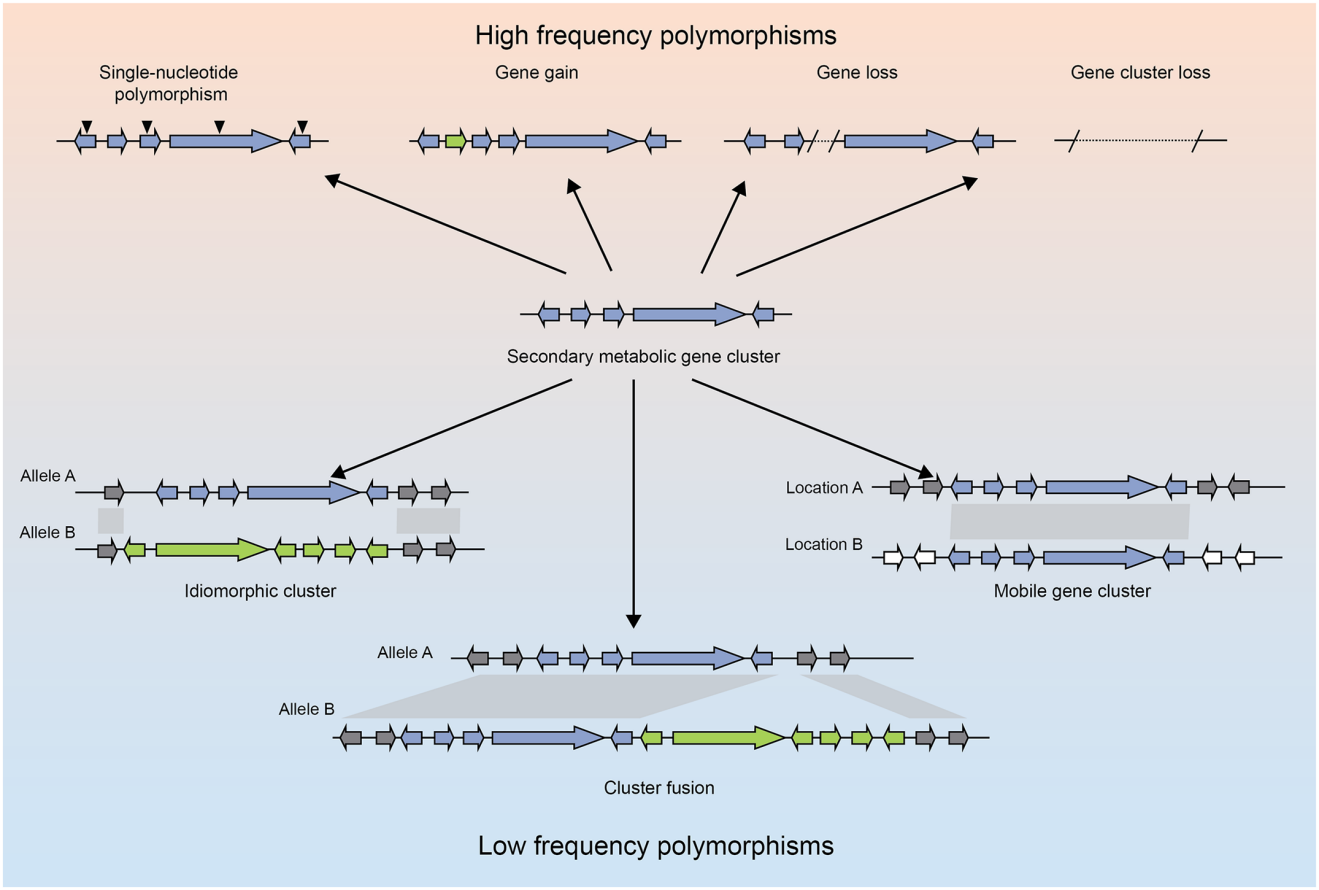
Types and frequencies of all SM gene cluster variants within *A*. *fumigatus*. SM, secondary metabolite.

Our examination of the genomes of 66 strains of *A*. *fumigatus* revealed 5 general types of polymorphisms that describe variation in SM gene clusters. These polymorphisms include variation in SNPs and short indels, gene and gene cluster gains and losses, nonhomologous (idiomorph) gene clusters at the same genomic position, and mobile clusters that differ in their genomic location across strains (Fig 6). Previous work has demonstrated that SM gene clusters, like the metabolites that they produce, are highly divergent between fungal species [8,9,19,64]. Our examination of genome-wide variation shows that these SM gene clusters are also diverse across strains of a single fungal species. These results also demonstrate that the diversity of SM gene clusters within *A*. *fumigatus* cannot be captured by sequencing a single representative strain, which is the current standard practice for determining the SM gene cluster content of a fungal species.

The quantification of diversity in SM gene clusters within a species is dependent on both numbers and types of strains analyzed. The types of polymorphisms detected as well as their observed frequency, especially for rare polymorphisms, will increase with the number of genomes examined. In addition, both the frequencies of the different types of polymorphisms and the polymorphisms themselves may also change with sampling design or in a manner corresponding to the population structure or ecology of the species under study. *A*. *fumigatus* is a cosmopolitan species with panmictic population structure [28], characteristics that do not always apply to other filamentous fungi. Fungi exhibiting strong population structure or fungi adapted to different ecological niches might contain different patterns of genetic diversity.

Nevertheless, the variants and genetic drivers we observe at the within-species level are also implicated as driving SM gene cluster variation at the between-species level, suggesting that the observed microevolutionary processes are sufficient to explain macroevolutionary patterns of SM gene cluster evolution. For example, the narrow and discontinuous distribution of SM gene clusters across the fungal phylogeny has been attributed to HGT as well as to gene cluster loss [13,15,20,22,30,66–68]. Here, we find evidence that both processes also influence the distribution of SM gene clusters within a species (Figs 2 and 5, S2–S5 Figs). Interestingly, the fraction of SM gene clusters within *A*. *fumigatus* that harbor loss of function polymorphisms is substantial, consistent with the macroevolutionary view that SM gene cluster loss is rampant [18,19,68]. However, our within-species observations are also consistent with the macroevolutionary importance of HGT to SM gene cluster evolution. Once thought to be nonexistent in eukaryotes, HGT is now considered to be responsible for the presence of several different SM gene clusters in diverse filamentous fungi [13,68,69]. The instances of HGT of SM gene clusters within *A*. *fumigatus* suggests that acquisition of foreign genetic material containing SM gene clusters is likely a common and ongoing occurrence in fungal populations.

One recurring theme across different types of SM gene cluster polymorphisms in *A*. *fumigatus* was the perpetual presence of TEs adjacent to or within clusters. One particularly striking case is the “idiomorphic” cluster 10, in which TEs seem to correspond with break points in synteny both within *A*. *fumigatus* and also between *A*. *fumigatus* and its close relatives (Fig 4, S8 Fig). TEs were also present flanking mobile and horizontally transferred SM gene clusters and were located adjacent to gene gain sites. There are several potential explanations for the observed TE enrichment. First, TE presence may promote repeat-driven recombination and gene rearrangement, or the TEs themselves may be the agents of horizontally transferred clusters (either on their own or through a viral vector). Alternatively, it may simply be the case that SM gene clusters preferentially reside in TE-rich genomic regions.

In summary, examination of SM gene cluster variation within a single fungal species revealed 5 distinct types of polymorphism that are widespread across different types of SM gene clusters and are caused by many underlying genetic drivers, including errors in DNA transcription and repair, nonhomologous recombination, gene duplication and loss, and HGT. The net effect of the observed variation raises the hypothesis that the chemical products of filamentous fungal species are in a state of evolutionary flux, each population constantly altering its SM gene cluster repertoire and consequently modifying its chemodiversity.

## Materials and methods

### Strains analyzed

Eight strains of *A*. *fumigatus* were isolated from 4 patients with recurrent cases of aspergillosis in the Portuguese Oncology Institute in Porto, Portugal. Each strain was determined to be *A*. *fumigatus* using macroscopic features of the culture and microscopic morphology observed in the slide preparation from the colonies with lactophenol solution [70]. Based on the morphological characterization, all clinical strains were classified as *A*. *fumigatus complex*-Fumigati. After whole genome sequencing, retrieval and examination of the beta tubulin and calmodulin sequences of each strain confirmed that all strains belonged to *A*. *fumigatus* (see Phylogenetic analysis and S9 Fig). The genomes of all 8 strains were sequenced using 150-bp Illumina paired-end sequence reads at the Genomic Services Lab of Hudson Alpha (Huntsville, Alabama, USA). Genomic libraries were constructed with the Illumina TruSeq library kit and sequenced on an Illumina HiSeq 2500 sequencer. Samples of all 8 strains were sequenced at greater than 180X coverage or depth (S1 Table). Short-read sequences for these 8 strains are available in the NCBI Sequence Read Archive (SRA) under accession SRP109032 (https://trace.ncbi.nlm.nih.gov/Traces/sra/?study=SRP109032).

In addition to the 8 strains sequenced in this study, we retrieved 58 *A*. *fumigatus* strains with publicly available whole genome sequencing data, resulting in a dataset of 66 strains (S1 Table). The strains used included both environmental and clinical strains and were isolated from multiple continents. Genome assemblies for 10 of these strains, including the Af293 and A1163 reference strains, were available for download from GenBank [28–32,71]. For 6 of these strains, short-read sequences were also available from the NCBI SRA, which were used for variant discovery only (see Single nucleotide variant [SNV] and indel discovery) and not for genome assembly. Short-read sequences were not available for the remaining 4 strains. Short-read sequences were downloaded for an additional 48 strains from the NCBI SRA if they were sequenced with paired-end reads and at greater than 30X coverage.

### Single nucleotide variant (SNV) and indel discovery

All strains with available short-read data (62 of 66 strains) were aligned to both the Af293 and A1163 reference genomes using BWA mem version 0.7.12-r1044 [72]. Coverage of genes present in the reference genome was calculated using bedtools v2.25.0 [73]. SNV and indel discovery and genotyping were performed relative to the Af293 reference genome and were conducted across all samples simultaneously using the Genome Analysis Toolkit version 3.5-0-g36282e4 with recommended hard filtering parameters [74–76] and annotated using snpEff version 4.2 [77].

### De novo genome assembly and gene annotation

All 56 strains without publicly available genome assemblies were de novo assembled using the iWGS pipeline [78]. Specifically, all strains were assembled using SPAdes v3.6.2 and MaSuRCA v3.1.3 and resulting assemblies were evaluated using QUAST v3.2 [79–81]. The average N50 of assemblies constructed with this strategy was 463 kb (S1 Table). Genes were annotated in these assemblies as well as in 5 GenBank assemblies with no predicted genes using augustus v3.2.2 trained on *A*. *fumigatus* gene models [82]. Repetitive elements were annotated in all assemblies using RepeatMasker version open-4.0.6 [83].

### Secondary metabolic gene cluster annotation and discovery

Secondary metabolic gene clusters in the Af293 reference genome were taken from 2 recent reviews, both of which considered computational and experimental data to delineate cluster boundaries [84,85] (S3 Table). The genomes of the other 65 strains were scanned for novel SM gene clusters using antiSMASH v3.0.5.1 [86]. To prevent potential assembly errors from confounding the analysis, any inference about changes in genomic locations of genes or gene clusters was additionally verified by manually inspecting alignments and ensuring that paired end reads supported an alternative genomic location (see Single nucleotide variant [SNV] and indel discovery). Cases in which paired end reads did not support the change in genomic location (i.e., all 3′ read mapping to chromosome 1 and all 5′ pairs mapping to chromosome 8) or mapping was ambiguous or low quality were discarded.

### Phylogenetic analysis

To confirm all strains in this analysis belonged to the species *A*. *fumigatus*, the genomic sequences of the beta tubulin and calmodulin genes were extracted from the assembled genomes of all strains. Gene phylogenies were constructed using *A*. *fischerianus* as an out-group using RAxML v8.0.25 with the GTRGAMMA substitution model [87]. The tree was midpoint rooted and all branches with bootstrap support less than 80% were collapsed (S10 Fig).

To construct an SNP-based strain phylogeny, biallelic SNPs with no missing data were pruned using SNPRelate v1.8.0 with a linkage disequilibrium threshold of 0.8 [88]. A total of 15,274 SNVs were used to create a phylogeny using RAxML v8.0.25 with the ASC_BINGAMMA substitution model [87]. The tree was midpoint rooted and all branches with bootstrap support less than 80% were collapsed. The phylogeny was visualized using ITOL version 3.0 [89].

To understand the evolutionary histories of specific SM gene clusters showing unusual taxonomic distributions, we reconstructed the phylogenetic trees of their SM genes. Specifically, SM cluster protein sequences were queried against a local copy of the NCBI nonredundant protein database (downloaded May 30, 2017) using phmmer, a member of the HMMER3 software suite [90], using acceleration parameters—F1 1e-5—F2 1e-7—F3 1e-10. A custom perl script sorted the phmmer results based on the normalized bitscore (nbs), in which nbs was calculated as the bitscore of the single best-scoring domain in the hit sequence divided by the best bitscore possible for the query sequence (i.e., the bitscore of the query aligned to itself). No more than 5 hits were retained for each unique NCBI Taxonomy ID. Full-length proteins corresponding to the top 100 hits (E-value < 1 × 10 − 10) to each query sequence were extracted from the local database using esl-sfetch [90]. Sequences were aligned with MAFFT v7.310 using the E-INS-i strategy and the BLOSUM30 amino acid scoring matrix [91] and trimmed with trimAL v1.4.rev15 using its gappyout strategy [92]. The topologies were inferred using maximum likelihood, as implemented in RAxML v8.2.9 [87], using empirically determined substitution models and rapid bootstrapping (1,000 replications). The phylogenies were midpoint rooted and branches with less than 80% bootstrap support were collapsed using the ape and phangorn R packages [93,94]. Phylogenies were visualized using ITOL version 3.0 [89].

To understand the evolutionary histories of SM gene clusters 4 and 10, full-length nucleotide sequences of all 62 strains with short-read sequence data were extracted for the entire cluster region (SM gene cluster 4) or the downstream flanking region (SM gene cluster 10) using the previously described SNV analysis procedure followed by Genome Analysis Toolkit’s “ExtractAlternativeReferenceFasta” tool [75]. The resulting nucleotide sequences were aligned using MAFFT v7.310 [91]. Phylogenies were constructed using maximum likelihood as implemented in RAxML v 8.0.25, using the GTRGAMMA substitution model and rapid bootstrapping (1,000 replications) [87]. Phylogenies were midpoint rooted and branches with less than 80% bootstrap support were collapsed. Phylogenies were visualized using ITOL version 3.0 [89].

All sequence alignments and phylogenies generated in this study are available on the Figshare repository (https://figshare.com/projects/Data_for_Drivers_of_genetic_diversity_in_secondary_metabolic_gene_clusters_within_a_fungal_species_/26089).

### Differential metabolite analysis

For natural product analysis, 5 × 10^6^ spores/mL for the indicated strains were grown in 50 mL liquid GMM [95] for 5 days at 25°C and 250 rpm in duplicates. Supernatants were extracted with equal volumes of ethyl acetate, dried down and resuspended in 20% acetonitrile (ACN). Each sample was analyzed by ultra high-performance liquid chromatography (UHPLC) coupled with mass spectrometry (MS). The samples were separated on a ZORBAX Eclipse XDB-C18 column (Agilent, 2.1 × 150 mm with a 1.8 μM particle size) using a binary gradient of 0.5% (v/v) formic acid (FA) as solvent A and 0.5% (v/v) FA in ACN as solvent B that was delivered by a VanquishTM UHPLC system (Thermo Scientific) with a flow rate of 0.2 mL/min. The binary gradient started with 20% B that was increased with a linear gradient to 100% B in 15 min followed by an isocratic step at 100% B for 5 min. Before every run, the system was equilibrated for 5 min at 20% B. The UHPLC system was coupled to a Q Exactive hybrid quadrupole OritrapTM MS (Thermo Scientific). For electrospray ionization, the ion voltage was set at ±3.5 kV in positive and negative mode. Nitrogen was used as sheath gas at a flow rate of 45 and as sweep gas at a flow rate of 2. Data analysis was performed using XCMS [57] and Maven [96] software.

## Supporting information

S1 Fig Alignments showing deletion of genes in SM gene clusters. (A) Deletion of helvolic acid genes in IF1SWF4. (B) Deletion of fumigaclavine genes in LMB35Aa. (C) Partial deletion of *gliM* in the gliotoxin gene cluster in 2 strains. (D) Partial deletion of *fmqE* in the fumiquinazoline gene cluster in 3 strains. (E,F) Coverage of 21 strains with partial deletion of ABC transporter gene in SM gene cluster 21. ABC, ATP-binding cassette; SM, secondary metabolite.
(PDF)

S2 Fig Gene phylogenies of the fusarielin-like SM gene cluster 4. These phylogenies are consistent with horizontal transfer between Eurotiomycete and Sordariomycete fungi or with extensive gene loss. SM, secondary metabolite.
(PDF)

S3 Fig Fusarielin-like clusters in Eurotiomycetes and Sordariomycetes. All species with genes grouping together with each *Aspergillus fumigatus* gene from the fusarielin-like cluster (see S2 Fig). *Beauveria bassiana* and *A*. *udagawae* were excluded, as they only contained the transcription factor from the cluster. The gene cluster in *Fusarium graminearum* has been functionally characterized as producing fusarielin.
(PDF)

S4 Fig Novel SM gene clusters in *Aspergillus fumigatus* strains. (A) Synteny between a novel PKS-containing cluster in 2 strains with an SM gene cluster in *Metarhizium anisopliae*. This novel PKS cluster is located between transposable elements in a region syntenic with the reference Af293 chromosome 4. (B) Novel SM gene cluster in MO54056EXP and 3 additional strains. This cluster is only located on 1 scaffold in MO540556EXP and is fragmented across the other strains (ends of scaffolds are marked). (C) Coverage data from short-read alignments for MO54056EXP, 12–7504462, and 08-19-02-30 relative to the MO54056EXP scaffold containing the novel SM gene cluster. PKS, polyketide synthase; SM, secondary metabolite.
(PDF)

S5 Fig Gene phylogenies of SM gene cluster 24. The phylogenies of several genes in this cluster are consistent with horizontal transfer between *Aspergillus fumigatus* and *Metarhizium* fungi. SM, secondary metabolite.
(PDF)

S6 Fig Two alleles of the idiomorphic SM gene cluster 10 present in 1 strain each. (A) This allele contains an insertion of genes from chromosome 6 immediately upstream of allele C (see main text Fig 4). None of these genes is likely an SM gene cluster backbone gene. An additional transposable element is found flanking this insertion. (B) This allele contains an insertion of genes present in the A1163 reference but not in the Af293 reference in the middle of allele A (see main text Fig 4). None of these genes is likely an SM gene cluster backbone gene. One additional transposable element is contained in this insertion. SM, secondary metabolite.
(PDF)

S7 Fig Metabolomics analysis of strains with different alleles of the idiomorphic cluster indicates the presence of different metabolites. Extracted ion chromatograms for the 2 mass to charge ratios identified in negative mode from XCMS analysis comparing extracts from strains with alleles D and F.
(PDF)

S8 Fig Idiomorph locus in other species. Structure of the idiomorph locus in (A) *Aspergillus lentulus* and (B) *A*. *fischeri* and homology with *A*. *fumigatus* allele E (main text Fig 4). Green arrows denote backbone biosynthetic genes and red boxes denote transposable elements as detected by RepeatMasker. *A*. *fischeri* contains a novel SM gene cluster not found in *A*. *fumig*atus strains. Other genes at this locus have various functions that may not be related to secondary metabolism. *A*. *lentulu*s contains 1 gene with a heterokaryon incompatibility domain, which may play a role in vegetative incompatibility. SM, secondary metabolite.
(PDF)

S9 Fig Gene phylogenies of the mobile SM gene cluster 1. These phylogenies are consistent with horizontal transfer between Eurotiomycete, Dothidiomycete, Leotiomycete, and Sordariomycete fungi. SM, secondary metabolite.
(PDF)

S10 Fig Marker gene phylogenies of all strains and *Aspergillus fischeri*. (A) Phylogeny of beta tubulin gene. (B) Phylogeny of calmodulin gene.
(PDF)

S1 Table *Aspergillus fumigatus* strain information. (XLSX)

S2 Table Nonsynonymous variants in *Aspergillus fumigatus* strains. (XLSX)

S3 Table Secondary metabolic gene clusters in *Aspergillus fumigatus* Af293. (XLSX)

S4 Table Metabolite analysis. (XLSX)

## Abbreviations

ABC: ATP-binding cassette
HGT: horizontal gene transfer
HR: highly reducing
NR: nonreducing
NRPS: nonribosomal peptide synthase
ORF: open reading frame
PKS: polyketide synthase
SM: secondary metabolite
SNP: single nucleotide polymorphism
SNV: single nucleotide variant
TE: transposable element

## References

[1] Vining LC. Functions of secondary metabolites. Annu Rev Microbiol. Annual Reviews 4139 El Camino Way, P.O. Box 10139, Palo Alto, CA 94303–0139, USA; 1990;44: 395–427. doi: 10.1146/annurev.mi.44.100190.002143 2252388 10.1146/annurev.mi.44.100190.002143

[2] Schimek C. Evolution of Special Metabolism in Fungi: Concepts, Mechanisms, and Pathways In: Pöggler S, Wöstmeyer J, editors. Evolution of Fungi and Fungal-Like Organisms, The Mycota. XIV Berlin, Heidelberg: Springer-Verlag; 2011 pp. 293–328.

[3] Fox EM, Howlett BJ. Secondary metabolism: regulation and role in fungal biology. Curr Opin Microbiol. 2008;11: 481–7. doi: 10.1016/j.mib.2008.10.007 18973828 10.1016/j.mib.2008.10.007

[4] Scharf DH, Heinekamp T, Brakhage AA. Human and Plant Fungal Pathogens: The Role of Secondary Metabolites. PLoS Pathog. 2014;10(1): e1003859 doi: 10.1371/journal.ppat.1003859 24497825 10.1371/journal.ppat.1003859 PMC3907374

[5] Yim G, Wang HH, Davies J. Antibiotics as signalling molecules. Philos Trans R Soc Lond B Biol Sci. 2007;362: 1195–200. doi: 10.1098/rstb.2007.2044 17360275 10.1098/rstb.2007.2044 PMC2435582

[6] Keller NP. Translating biosynthetic gene clusters into fungal armor and weaponry. Nat Chem Biol. Nature Publishing Group, a division of Macmillan Publishers Limited. All Rights Reserved.; 2015;11: 671–7. doi: 10.1038/nchembio.1897 26284674 10.1038/nchembio.1897 PMC4682562

[7] Bennett J, Bentley R. What’s in a name?—Microbial secondary metabolism. Adv Appl Microbiol. 1989;34.

[8] Khaldi N, Seifuddin FT, Turner G, Haft D, Nierman WC, Wolfe KH, et al SMURF: Genomic mapping of fungal secondary metabolite clusters. Fungal Genet Biol. 2010;47: 736–41. doi: 10.1016/j.fgb.2010.06.003 20554054 10.1016/j.fgb.2010.06.003 PMC2916752

[9] Lind AL, Wisecaver JH, Smith TD, Feng X, Calvo AM, Rokas A. Examining the evolution of the regulatory circuit controlling secondary metabolism and development in the fungal genus Aspergillus. PLoS Genet. 2015;11(3): e1005096 doi: 10.1371/journal.pgen.1005096 25786130 10.1371/journal.pgen.1005096 PMC4364702

[10] Proctor RH, Busman M, Seo J-A, Lee YW, Plattner RD. A fumonisin biosynthetic gene cluster in Fusarium oxysporum strain O-1890 and the genetic basis for B versus C fumonisin production. Fungal Genet Biol. 2008;45: 1016–1026. doi: 10.1016/j.fgb.2008.02.004 18375156 10.1016/j.fgb.2008.02.004

[11] Proctor RH, McCormick SP, Alexander NJ, Desjardins AE. Evidence that a secondary metabolic biosynthetic gene cluster has grown by gene relocation during evolution of the filamentous fungus Fusarium. Mol Microbiol. 2009;74: 1128–42. doi: 10.1111/j.1365-2958.2009.06927.x 19843228 10.1111/j.1365-2958.2009.06927.x

[12] Berry DB, Guan Q, Hose J, Haroon S, Gebbia M, Heisler LE, et al Multiple Means to the Same End: The Genetic Basis of Acquired Stress Resistance in Yeast. PLoS Genet. 2011;7(11): e1002353 doi: 10.1371/journal.pgen.1002353 22102822 10.1371/journal.pgen.1002353 PMC3213159

[13] Slot JC, Rokas A. Horizontal transfer of a large and highly toxic secondary metabolic gene cluster between fungi. Curr Biol. Elsevier Ltd; 2011;21: 134–9. doi: 10.1016/j.cub.2010.12.020 21194949 10.1016/j.cub.2010.12.020

[14] Ehrlich KC, Chang P-K, Yu J, Cotty PJ. Aflatoxin Biosynthesis Cluster Gene cypA Is Required for G Aflatoxin Formation. Appl Environ Microbiol. 2004;70: 6518–6524. doi: 10.1128/AEM.70.11.6518-6524.2004 15528514 10.1128/AEM.70.11.6518-6524.2004 PMC525170

[15] Carbone I, Ramirez-Prado JH, Jakobek JL, Horn BW. Gene duplication, modularity and adaptation in the evolution of the aflatoxin gene cluster. BMC Evol Biol. 2007;7: 111 doi: 10.1186/1471-2148-7-111 17620135 10.1186/1471-2148-7-111 PMC1949824

[16] Yu J, Chang P-K, Ehrlich KC, Cary JW, Bhatnagar D, Cleveland TE, et al Clustered pathway genes in aflatoxin biosynthesis. Appl Environ Microbiol. American Society for Microbiology; 2004;70: 1253–62. doi: 10.1128/AEM.70.3.1253-1262.2004 10.1128/AEM.70.3.1253-1262.2004 PMC368384 15006741

[17] Campbell MA, Rokas A, Slot JC. Horizontal transfer and death of a fungal secondary metabolic gene cluster. Genome Biol Evol. 2012;4: 289–93. doi: 10.1093/gbe/evs011 22294497 10.1093/gbe/evs011 PMC3318441

[18] Kroken S, Glass NL, Taylor JW, Yoder OC, Turgeon BG. Phylogenomic analysis of type I polyketide synthase genes in pathogenic and saprobic ascomycetes. Proc Natl Acad Sci U S A. 2003;100: 15670–5. doi: 10.1073/pnas.2532165100 14676319 10.1073/pnas.2532165100 PMC307626

[19] Bushley KE, Turgeon BG. Phylogenomics reveals subfamilies of fungal nonribosomal peptide synthetases and their evolutionary relationships. BMC Evol Biol. 2010;10: 26 doi: 10.1186/1471-2148-10-26 20100353 10.1186/1471-2148-10-26 PMC2823734

[20] Patron NJ, Waller RF, Cozijnsen AJ, Straney DC, Gardiner DM, Nierman WC, et al Origin and distribution of epipolythiodioxopiperazine (ETP) gene clusters in filamentous ascomycetes. BMC Evol Biol. 2007;7: 174 doi: 10.1186/1471-2148-7-174 17897469 10.1186/1471-2148-7-174 PMC2045112

[21] Khaldi N, Collemare J, Lebrun M-H, Wolfe KH. Evidence for horizontal transfer of a secondary metabolite gene cluster between fungi. Genome Biol. BioMed Central; 2008;9: R18 doi: 10.1186/gb-2008-9-1-r18 18218086 10.1186/gb-2008-9-1-r18 PMC2395248

[22] Khaldi N, Wolfe KH. Evolutionary Origins of the Fumonisin Secondary Metabolite Gene Cluster in Fusarium verticillioides and Aspergillus niger. Int J Evol Biol. Hindawi Publishing Corporation; 2011;2011: 423821 doi: 10.4061/2011/423821 21716743 10.4061/2011/423821 PMC3119522

[23] Reynolds HT, Slot JC, Divon HH, Lysøe E, Proctor RH, Brown DW. Differential Retention of Gene Functions in a Secondary Metabolite Cluster. Mol Biol Evol. Oxford University Press (OUP); 2017;10: e1004816 doi: 10.1093/molbev/msx145 28460114 10.1093/molbev/msx145

[24] Wiemann P, Sieber CMK, von Bargen KW, Studt L, Niehaus E-M, Espino JJ, et al Deciphering the Cryptic Genome: Genome-wide Analyses of the Rice Pathogen Fusarium fujikuroi Reveal Complex Regulation of Secondary Metabolism and Novel Metabolites. PLoS Pathog. 2013;9(6): e1003475 doi: 10.1371/journal.ppat.1003475 23825955 10.1371/journal.ppat.1003475 PMC3694855

[25] Chiara M, Fanelli F, Mulè G, Logrieco AF, Pesole G, Leslie JF, et al Genome Sequencing of Multiple Isolates Highlights Subtelomeric Genomic Diversity within *Fusarium fujikuroi*. Genome Biol Evol. 2015;7: 3062–3069. doi: 10.1093/gbe/evv198 26475319 10.1093/gbe/evv198 PMC5635591

[26] Chang P-K, Horn BW, Dorner JW. Sequence breakpoints in the aflatoxin biosynthesis gene cluster and flanking regions in nonaflatoxigenic Aspergillus flavus isolates. Fungal Genet Biol. 2005;42: 914–923. doi: 10.1016/j.fgb.2005.07.004 16154781 10.1016/j.fgb.2005.07.004

[27] Schumacher J, Gautier A, Morgant G, Studt L, Ducrot P-H, Le Pêcheur P, et al A Functional Bikaverin Biosynthesis Gene Cluster in Rare Strains of Botrytis cinerea Is Positively Controlled by VELVET. PLoS ONE. 2013;8(1): e53729 doi: 10.1371/journal.pone.0053729 23308280 10.1371/journal.pone.0053729 PMC3538735

[28] Abdolrasouli A, Rhodes J, Beale MA, Hagen F, Rogers TR, Chowdhary A, et al Genomic Context of Azole Resistance Mutations in Aspergillus fumigatus Determined Using Whole-Genome Sequencing. MBio. 2015;6 doi: 10.1128/mBio.00536-15 26037120 10.1128/mBio.00536-15 PMC4453006

[29] Nierman WC, Pain A, Anderson MJ, Wortman JR, Kim HS, Arroyo J, et al Genomic sequence of the pathogenic and allergenic filamentous fungus Aspergillus fumigatus. Nature. 2005;438: 1151–6. doi: 10.1038/nature04332 16372009 10.1038/nature04332

[30] Fedorova ND, Khaldi N, Joardar VS, Maiti R, Amedeo P, Anderson MJ, et al Genomic islands in the pathogenic filamentous fungus Aspergillus fumigatus. Richardson PM, editor. PLoS Genet. 2008;4(4): e1000046 doi: 10.1371/journal.pgen.1000046 18404212 10.1371/journal.pgen.1000046 PMC2289846

[31] Knox BP, Blachowicz A, Palmer JM, Romsdahl J, Huttenlocher A, Wang CCC, et al Characterization of Aspergillus fumigatus Isolates from Air and Surfaces of the International Space Station. mSphere. 2016;1. 10.1128/mSphere.00227-16 PMC5082629 27830189

[32] Paul S, Zhang A, Ludeña Y, Villena GK, Yu F, Sherman DH, et al Insights from the genome of a high alkaline cellulase producing Aspergillus fumigatus strain obtained from Peruvian Amazon rainforest. J Biotechnol. 2017;251: 53–58. doi: 10.1016/j.jbiotec.2017.04.010 28412514 10.1016/j.jbiotec.2017.04.010

[33] Ahn JH, Walton JD. Chromosomal organization of TOX2, a complex locus controlling host-selective toxin biosynthesis in Cochliobolus carbonum. Plant Cell. American Society of Plant Biologists; 1996;8: 887–97. doi: 10.1105/tpc.8.5.887 8672886 10.1105/tpc.8.5.887 PMC161146

[34] Zhang H, Rokas A, Slot JCJ. Two different secondary metabolism gene clusters occupied the same ancestral locus in fungal dermatophytes of the Arthrodermataceae. PLoS ONE. 2012;7(7): e41903 doi: 10.1371/journal.pone.0041903 22860027 10.1371/journal.pone.0041903 PMC3408471

[35] Gibbons JG, Salichos L, Slot JC, Rinker DC, McGary KL, King JG, et al The evolutionary imprint of domestication on genome variation and function of the filamentous fungus Aspergillus oryzae. Curr Biol. 2012;22: 1403–9. doi: 10.1016/j.cub.2012.05.033 22795693 10.1016/j.cub.2012.05.033 PMC3416971

[36] Roberts JD, Kunkel TA. Fidelity of DNA replication In: DePamphilis ML, editor. DNA replication in eukaryotic cells. Cold Spring Harbor, New York: Cold Spring Harbor Laboratory Press; 1996 pp. 217–247.

[37] Throckmorton K, Lim FY, Kontoyiannis DP, Zheng W, Keller NP. Redundant synthesis of a conidial polyketide by two distinct secondary metabolite clusters in Aspergillus fumigatus. Environ Microbiol. 2015; doi: 10.1111/1462-2920.13007 26242966 10.1111/1462-2920.13007 PMC4750049

[38] O’Hanlon KA, Cairns T, Stack D, Schrettl M, Bignell EM, Kavanagh K, et al Targeted Disruption of Nonribosomal Peptide Synthetase pes3 Augments the Virulence of Aspergillus fumigatus. Infect Immun. 2011;79: 3978–3992. doi: 10.1128/IAI.00192-11 21746855 10.1128/IAI.00192-11 PMC3187245

[39] Kapitonov V V., Jurka J. A universal classification of eukaryotic transposable elements implemented in Repbase. Nat Rev Genet. Nature Publishing Group; 2008;9: 411–412. doi: 10.1038/nrg2165-c1 18421312 10.1038/nrg2165-c1

[40] Mount SM, Rubin GM. Complete nucleotide sequence of the Drosophila transposable element copia: homology between copia and retroviral proteins. Mol Cell Biol. 1985;5: 1630–8. 2410772 10.1128/mcb.5.7.1630-1638.1985 PMC367281

[41] Sørensen JL, Hansen FT, Sondergaard TE, Staerk D, Lee TV, Wimmer R, et al Production of novel fusarielins by ectopic activation of the polyketide synthase 9 cluster in Fusarium graminearum. Environ Microbiol. Blackwell Publishing Ltd; 2012;14: 1159–1170. doi: 10.1111/j.1462-2920.2011.02696.x 22252016 10.1111/j.1462-2920.2011.02696.x

[42] Futagami T, Mori K, Yamashita A, Wada S, Kajiwara Y, Takashita H, et al Genome Sequence of the White Koji Mold Aspergillus kawachii IFO 4308, Used for Brewing the Japanese Distilled Spirit Shochu. Eukaryot Cell. 2011;10: 1586–1587. doi: 10.1128/EC.05224-11 22045919 10.1128/EC.05224-11 PMC3209066

[43] Andersen MR, Salazar MP, Schaap PJ, van de Vondervoort PJI, Culley D, Thykaer J, et al Comparative genomics of citric-acid-producing Aspergillus niger ATCC 1015 versus enzyme-producing CBS 513.88. Genome Res. 2011;21: 885–97. doi: 10.1101/gr.112169.110 21543515 10.1101/gr.112169.110 PMC3106321

[44] Yu J, Wu G, Jurick WM, Gaskins VL, Yin Y, Yin G, et al Genome Sequence of *Penicillium solitum* RS1, Which Causes Postharvest Apple Decay. Genome Announc. 2016;4: e00363–16. doi: 10.1128/genomeA.00363-16 27174276 10.1128/genomeA.00363-16 PMC4866853

[45] Yamada O, Machida M, Hosoyama A, Goto M, Takahashi T, Futagami T, et al Genome sequence of *Aspergillus luchuensis* NBRC 4314. DNA Res. 2016;23: 507–515. doi: 10.1093/dnares/dsw032 27651094 10.1093/dnares/dsw032 PMC5144674

[46] Cheeseman K, Ropars J, Renault P, Dupont J, Gouzy J, Branca A, et al Multiple recent horizontal transfers of a large genomic region in cheese making fungi. Nat Commun. 2014;5: 2876 doi: 10.1038/ncomms3876 24407037 10.1038/ncomms3876 PMC3896755

[47] Baroncelli R, Sreenivasaprasad S, Sukno SA, Thon MR, Holub E. Draft Genome Sequence of Colletotrichum acutatum Sensu Lato (Colletotrichum fioriniae). Genome Announc. 2014;2: e00112-14–e00112-14. doi: 10.1128/genomeA.00112-14 24723700 10.1128/genomeA.00112-14 PMC3983289

[48] Hacquard S, Kracher B, Hiruma K, Münch PC, Garrido-Oter R, Thon MR, et al Survival trade-offs in plant roots during colonization by closely related beneficial and pathogenic fungi. Nat Commun. 2016;7: 11362 doi: 10.1038/ncomms11362 27150427 10.1038/ncomms11362 PMC4859067

[49] King R, Urban M, Hammond-Kosack MCU, Hassani-Pak K, Hammond-Kosack KE. The completed genome sequence of the pathogenic ascomycete fungus Fusarium graminearum. BMC Genomics. 2015;16: 544 doi: 10.1186/s12864-015-1756-1 26198851 10.1186/s12864-015-1756-1 PMC4511438

[50] Kazan K, Gardiner DM, Manners JM. On the trail of a cereal killer: Recent advances in Fusarium graminearum pathogenomics and host resistance. Molecular Plant Pathology. 2012 pp. 399–413. doi: 10.1111/j.1364-3703.2011.00762.x 22098555 10.1111/j.1364-3703.2011.00762.x PMC6638652

[51] Richards TA, Talbot NJ. Horizontal gene transfer in osmotrophs: playing with public goods. Nat Rev Microbiol. Nature Research; 2013;11: 720–727. doi: 10.1038/nrmicro3108 24018383 10.1038/nrmicro3108

[52] Morris JJ, Lenski RE, Zinser ER. The Black Queen Hypothesis: evolution of dependencies through adaptive gene loss. MBio. American Society for Microbiology; 2012;3: e00036–12. doi: 10.1128/mBio.00036-12 22448042 10.1128/mBio.00036-12 PMC3315703

[53] Treangen TJ, Salzberg SL. Repetitive DNA and next-generation sequencing: computational challenges and solutions. Nat Rev Genet. NIH Public Access; 2011;13: 36–46. doi: 10.1038/nrg3117 22124482 10.1038/nrg3117 PMC3324860

[54] Palmer JM, Keller NP. Secondary metabolism in fungi: does chromosomal location matter? Curr Opin Microbiol. 2010;13: 431–6. doi: 10.1016/j.mib.2010.04.008 20627806 10.1016/j.mib.2010.04.008 PMC2922032

[55] Gao Q, Jin K, Ying S-H, Zhang Y, Xiao G, Shang Y, et al Genome sequencing and comparative transcriptomics of the model entomopathogenic fungi Metarhizium anisopliae and M. acridum. PLoS Genet. 2011;7(1): e1001264 doi: 10.1371/journal.pgen.1001264 21253567 10.1371/journal.pgen.1001264 PMC3017113

[56] Metzenberg RL, Glass NL. Mating type and mating strategies inNeurospora. BioEssays. Wiley Subscription Services, Inc., A Wiley Company; 1990;12: 53–59. doi: 10.1002/bies.950120202 2140508 10.1002/bies.950120202

[57] Smith CA, Want EJ, O’Maille G, Abagyan R, Siuzdak G. XCMS: Processing Mass Spectrometry Data for Metabolite Profiling Using Nonlinear Peak Alignment, Matching, and Identification. Anal Chem. 2006;78: 779–787. doi: 10.1021/ac051437y 16448051 10.1021/ac051437y

[58] Espagne E, Balhadère P, Penin M-L, Barreau C, Turcq B. HET-E and HET-D belong to a new subfamily of WD40 proteins involved in vegetative incompatibility specificity in the fungus Podospora anserina. Genetics. 2002;161: 71–81. 12019224 10.1093/genetics/161.1.71 PMC1462119

[59] Wiemann P, Guo C-J, Palmer JM, Sekonyela R, Wang CCC, Keller NP. Prototype of an intertwined secondary-metabolite supercluster. Proc Natl Acad Sci U S A. 2013;110: 17065–70. doi: 10.1073/pnas.1313258110 24082142 10.1073/pnas.1313258110 PMC3801025

[60] Syme RA, Hane JK, Friesen TL, Oliver RP. Resequencing and Comparative Genomics of *Stagonospora nodorum* : Sectional Gene Absence and Effector Discovery. G3: Genes|Genomes|Genetics. 2013;3: 959–969. doi: 10.1534/g3.112.004994 23589517 10.1534/g3.112.004994 PMC3689807

[61] Chibucos MC, Crabtree J, Nagaraj S, Chaturvedi S, Chaturvedi V. Draft Genome Sequences of Human Pathogenic Fungus Geomyces pannorum Sensu Lato and Bat White Nose Syndrome Pathogen Geomyces (Pseudogymnoascus) destructans. Genome Announc. 2013;1: e01045-13–e01045-13. doi: 10.1128/genomeA.01045-13 24356829 10.1128/genomeA.01045-13 PMC3868853

[62] de Man TJB, Stajich JE, Kubicek CP, Teiling C, Chenthamara K, Atanasova L, et al Small genome of the fungus *Escovopsis weberi*, a specialized disease agent of ant agriculture. Proc Natl Acad Sci. 2016;113: 3567–3572. doi: 10.1073/pnas.1518501113 26976598 10.1073/pnas.1518501113 PMC4822581

[63] Wu W, Davis RW, Tran-Gyamfi MB, Kuo A, LaButti K, Mihaltcheva S, et al Characterization of four endophytic fungi as potential consolidated bioprocessing hosts for conversion of lignocellulose into advanced biofuels. Appl Microbiol Biotechnol. Springer Berlin Heidelberg; 2017;101: 2603–2618. doi: 10.1007/s00253-017-8091-1 28078400 10.1007/s00253-017-8091-1

[64] de Vries RP, Riley R, Wiebenga A, Aguilar-Osorio G, Amillis S, Uchima CA, et al Comparative genomics reveals high biological diversity and specific adaptations in the industrially and medically important fungal genus Aspergillus. Genome Biol. BioMed Central; 2017;18: 28 doi: 10.1186/s13059-017-1151-0 28196534 10.1186/s13059-017-1151-0 PMC5307856

[65] Lin H-C, Chooi Y-H, Dhingra S, Xu W, Calvo AM, Tang Y. The Fumagillin Biosynthetic Gene Cluster in Aspergillus fumigatus Encodes a Cryptic Terpene Cyclase Involved in the Formation of β-trans-Bergamotene. J Am Chem Soc. 2013;135: 4616–9. doi: 10.1021/ja312503y 23488861 10.1021/ja312503y PMC3652892

[66] Proctor RH, Van Hove F, Susca A, Stea G, Busman M, van der Lee T, et al Birth, death and horizontal transfer of the fumonisin biosynthetic gene cluster during the evolutionary diversification of Fusarium. Mol Microbiol. 2013;90: 290–306. doi: 10.1111/mmi.12362 23937442 10.1111/mmi.12362

[67] Wong S, Wolfe KH. Birth of a metabolic gene cluster in yeast by adaptive gene relocation. Nat Genet. Nature Publishing Group; 2005;37: 777–782. doi: 10.1038/ng1584 15951822 10.1038/ng1584

[68] Khaldi N, Collemare J, Lebrun M-H, Wolfe KH. Evidence for horizontal transfer of a secondary metabolite gene cluster between fungi. Genome Biol. 2008;9: R18 doi: 10.1186/gb-2008-9-1-r18 18218086 10.1186/gb-2008-9-1-r18 PMC2395248

[69] Wisecaver JH, Rokas A. Fungal metabolic gene clusters-caravans traveling across genomes and environments. Frontiers in Microbiology. Frontiers; 2015 p. 161 doi: 10.3389/fmicb.2015.00161 25784900 10.3389/fmicb.2015.00161 PMC4347624

[70] de Hoog G, Guarro J, Gené J, Figueras M. Atlas of Clinical Fungi. Washington, DC: ASM Press; 2001.

[71] Liu D, Zhang R, Yang X, Wu H, Xu D, Tang Z, et al Thermostable cellulase production of Aspergillus fumigatus Z5 under solid-state fermentation and its application in degradation of agricultural wastes. Int Biodeterior Biodegradation. 2011;65: 717–725. doi: 10.1016/j.ibiod.2011.04.005

[72] Li H, Durbin R. Fast and accurate short read alignment with Burrows-Wheeler transform. Bioinformatics. 2009;25: 1754–1760. doi: 10.1093/bioinformatics/btp324 19451168 10.1093/bioinformatics/btp324 PMC2705234

[73] Quinlan AR, Hall IM. BEDTools: a flexible suite of utilities for comparing genomic features. Bioinformatics. Oxford University Press; 2010;26: 841–2. doi: 10.1093/bioinformatics/btq033 20110278 10.1093/bioinformatics/btq033 PMC2832824

[74] McKenna A, Hanna M, Banks E, Sivachenko A, Cibulskis K, Kernytsky A, et al The Genome Analysis Toolkit: a MapReduce framework for analyzing next-generation DNA sequencing data. Genome Res. Cold Spring Harbor Laboratory Press; 2010;20: 1297–303. doi: 10.1101/gr.107524.110 20644199 10.1101/gr.107524.110 PMC2928508

[75] Van der Auwera GA, Carneiro MO, Hartl C, Poplin R, del Angel G, Levy-Moonshine A, et al From FastQ Data to High-Confidence Variant Calls: The Genome Analysis Toolkit Best Practices Pipeline. Current Protocols in Bioinformatics. Hoboken, NJ, USA: John Wiley & Sons, Inc.; 2013 p. 11.10.1–11.10.33. doi: 10.1002/0471250953.bi1110s43 25431634 10.1002/0471250953.bi1110s43 PMC4243306

[76] DePristo MA, Banks E, Poplin R, Garimella K V, Maguire JR, Hartl C, et al A framework for variation discovery and genotyping using next-generation DNA sequencing data. Nat Genet. 2011;43: 491–498. doi: 10.1038/ng.806 21478889 10.1038/ng.806 PMC3083463

[77] Cingolani P, Platts A, Wang LL, Coon M, Nguyen T, Wang L, et al A program for annotating and predicting the effects of single nucleotide polymorphisms, SnpEff: SNPs in the genome of Drosophila melanogaster strain w1118; iso-2; iso-3. Fly (Austin). Taylor & Francis; 2012;6: 80–92. doi: 10.4161/fly.19695 10.4161/fly.19695 PMC3679285 22728672

[78] Zhou X, Peris D, Kominek J, Kurtzman CP, Hittinger CT, Rokas A. in silico Whole Genome Sequencer & Analyzer (iWGS): A Computational Pipeline to Guide the Design and Analysis of de novo Genome Sequencing Studies. G3: Genes|Genomes|Genetics. 2016; doi: 10.1534/g3.116.034249 27638685 10.1534/g3.116.034249 PMC5100864

[79] Bankevich A, Nurk S, Antipov D, Gurevich AA, Dvorkin M, Kulikov AS, et al SPAdes: a new genome assembly algorithm and its applications to single-cell sequencing. J Comput Biol. Mary Ann Liebert, Inc.; 2012;19: 455–77. doi: 10.1089/cmb.2012.0021 22506599 10.1089/cmb.2012.0021 PMC3342519

[80] Zimin A V., Marcais G, Puiu D, Roberts M, Salzberg SL, Yorke JA. The MaSuRCA genome assembler. Bioinformatics. 2013;29: 2669–2677. doi: 10.1093/bioinformatics/btt476 23990416 10.1093/bioinformatics/btt476 PMC3799473

[81] Gurevich A, Saveliev V, Vyahhi N, Tesler G. QUAST: quality assessment tool for genome assemblies. Bioinformatics. 2013;29: 1072–1075. doi: 10.1093/bioinformatics/btt086 23422339 10.1093/bioinformatics/btt086 PMC3624806

[82] Stanke M, Morgenstern B. AUGUSTUS: a web server for gene prediction in eukaryotes that allows user-defined constraints. Nucleic Acids Res. Oxford University Press; 2005;33: W465–7. doi: 10.1093/nar/gki458 15980513 10.1093/nar/gki458 PMC1160219

[83] Smit A, Hubley R, Green P. Repeatmasker Open-4.0 [Internet]. [cited 10 Jan 2015]. http://www.repeatmasker.org

[84] Inglis DO, Binkley J, Skrzypek MS, Arnaud MB, Cerqueira GC, Shah P, et al Comprehensive annotation of secondary metabolite biosynthetic genes and gene clusters of Aspergillus nidulans, A. fumigatus, A. niger and A. oryzae. BMC Microbiol. 2013;13: 91 doi: 10.1186/1471-2180-13-91 23617571 10.1186/1471-2180-13-91 PMC3689640

[85] Bignell E, Cairns TC, Throckmorton K, Nierman WC, Keller NP. Secondary metabolite arsenal of an opportunistic pathogenic fungus. Philos Trans R Soc B Biol Sci. 2016;371. 10.1098/rstb.2016.0023 PMC5095546 28080993

[86] Medema MH, Blin K, Cimermancic P, de Jager V, Zakrzewski P, Fischbach MA, et al antiSMASH: rapid identification, annotation and analysis of secondary metabolite biosynthesis gene clusters in bacterial and fungal genome sequences. Nucleic Acids Res. 2011;39: W339–46. doi: 10.1093/nar/gkr466 21672958 10.1093/nar/gkr466 PMC3125804

[87] Stamatakis A. RAxML version 8: a tool for phylogenetic analysis and post-analysis of large phylogenies. Bioinformatics. 2014;30: 1312–1313. doi: 10.1093/bioinformatics/btu033 24451623 10.1093/bioinformatics/btu033 PMC3998144

[88] Zheng X, Levine D, Shen J, Gogarten SM, Laurie C, Weir BS. A high-performance computing toolset for relatedness and principal component analysis of SNP data. Bioinformatics. Oxford University Press; 2012;28: 3326–3328. doi: 10.1093/bioinformatics/bts606 23060615 10.1093/bioinformatics/bts606 PMC3519454

[89] Letunic I, Bork P. Interactive tree of life (iTOL) v3: an online tool for the display and annotation of phylogenetic and other trees. Nucleic Acids Res. 2016;44: W242–W245. doi: 10.1093/nar/gkw290 27095192 10.1093/nar/gkw290 PMC4987883

[90] Eddy SR. A new generation of homology search tools based on probabilistic inference. Genome Inform. 2009;23: 205–11. 20180275

[91] Katoh K, Standley DM. MAFFT Multiple Sequence Alignment Software Version 7: Improvements in Performance and Usability. Mol Biol Evol. 2013;30: 772–780. doi: 10.1093/molbev/mst010 23329690 10.1093/molbev/mst010 PMC3603318

[92] Capella-Gutierrez S, Silla-Martinez JM, Gabaldon T. trimAl: a tool for automated alignment trimming in large-scale phylogenetic analyses. Bioinformatics. 2009;25: 1972–1973. doi: 10.1093/bioinformatics/btp348 19505945 10.1093/bioinformatics/btp348 PMC2712344

[93] Paradis E, Claude J, Strimmer K. APE: Analyses of Phylogenetics and Evolution in R language. Bioinformatics. Oxford University Press; 2004;20: 289–290. doi: 10.1093/bioinformatics/btg412 10.1093/bioinformatics/btg412 14734327

[94] Schliep KP. phangorn: phylogenetic analysis in R. Bioinformatics. Oxford University Press; 2011;27: 592–3. doi: 10.1093/bioinformatics/btq706 21169378 10.1093/bioinformatics/btq706 PMC3035803

[95] Shimizu K, Keller NP. Genetic involvement of a cAMP-dependent protein kinase in a G protein signaling pathway regulating morphological and chemical transitions in Aspergillus nidulans. Genetics. 2001;157: 591–600. 11156981 10.1093/genetics/157.2.591 PMC1461531

[96] Melamud E, Vastag L, Rabinowitz JD. Metabolomic Analysis and Visualization Engine for LC−MS Data. Anal Chem. 2010;82: 9818–9826. doi: 10.1021/ac1021166 21049934 10.1021/ac1021166 PMC5748896

