# Supplementary figures and images for: Drivers of genetic diversity in secondary metabolic gene clusters within a fungal species
Source: PLoS Biol. 2017 Nov 17;15(11):e2003583. doi: 10.1371/journal.pbio.2003583 (PMC5711037; doi:10.1371/journal.pbio.2003583)

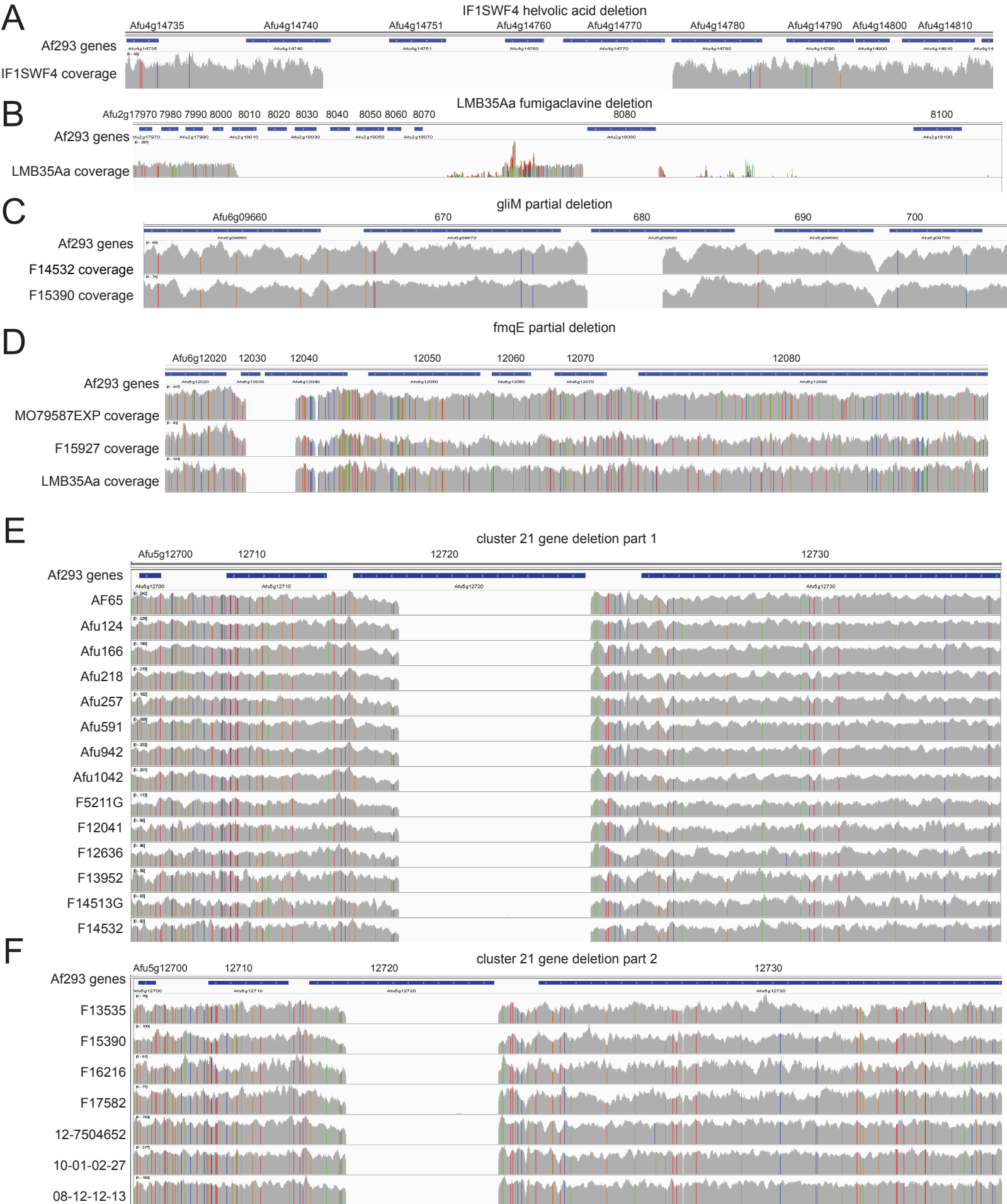

Supplement: S1 Fig — (A) Deletion of helvolic acid genes in IF1SWF4. (B) Deletion of fumigaclavine genes in LMB35Aa. (C) Partial deletion of gliM in the gliotoxin gene cluster in 2 strains. (D) Partial deletion of fmqE in the fumiquinazoline gene cluster in 3 strains. (E,F) Coverage of 21 strains with partial deletion of ABC transporter gene in SM gene cluster 21. ABC, ATP-binding cassette; SM, secondary metabolite. (PDF) [file pbio.2003583.s001.pdf]

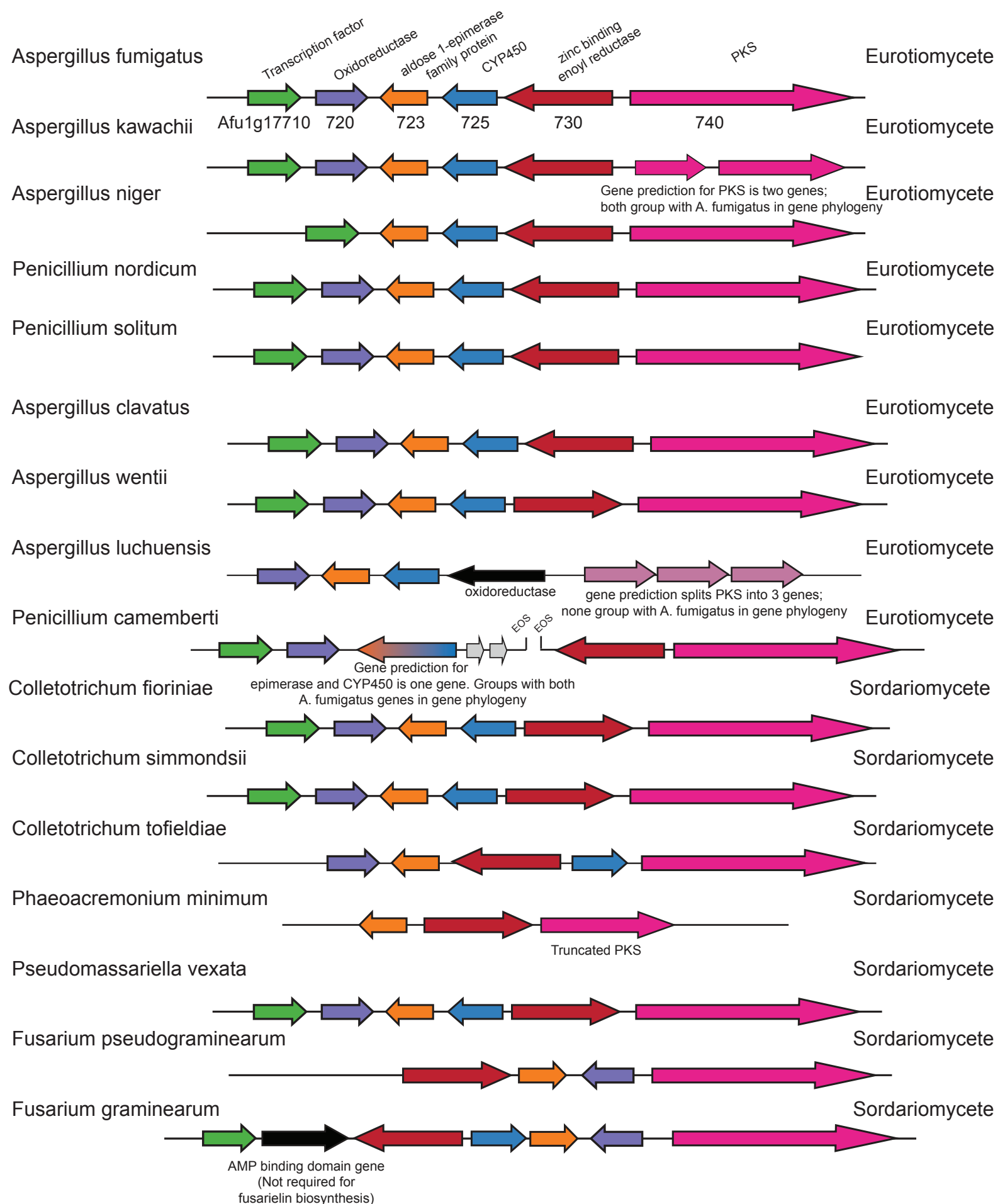

Supplement: S3 Fig — All species with genes grouping together with each Aspergillus fumigatus gene from the fusarielin-like cluster (see S2 Fig). Beauveria bassiana and A. udagawae were excluded, as they only contained the transcription factor from the cluster. The gene cluster in Fusarium graminearum has been functionally characterized as producing fusarielin. (PDF) [file pbio.2003583.s003.pdf]

# JCM10253 & F13619

A

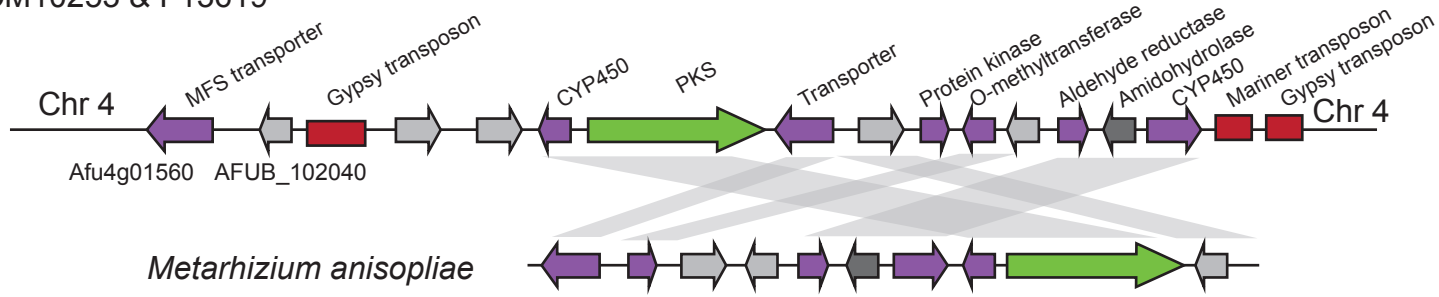

B

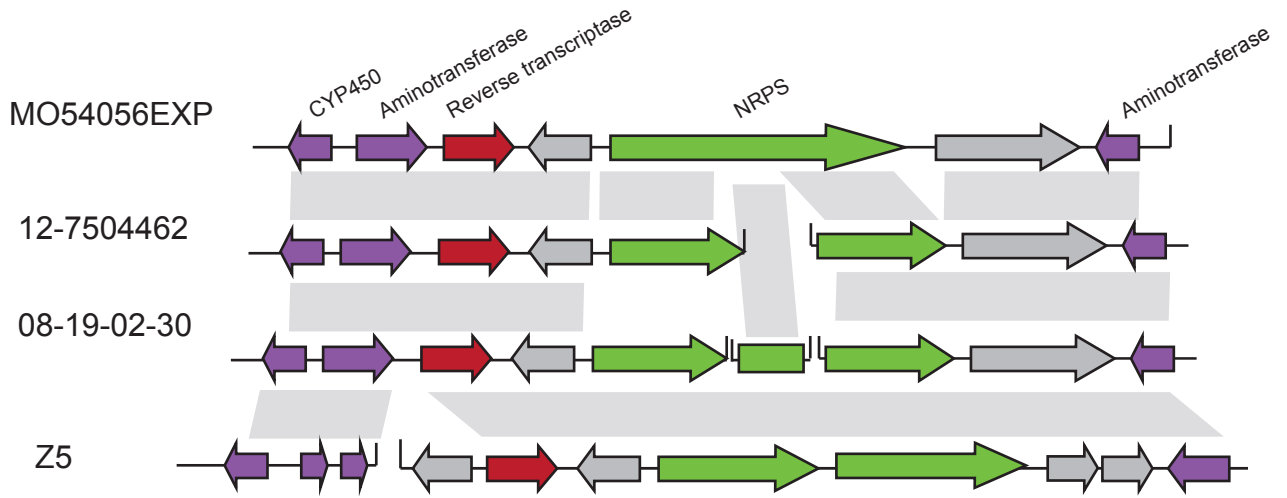

C

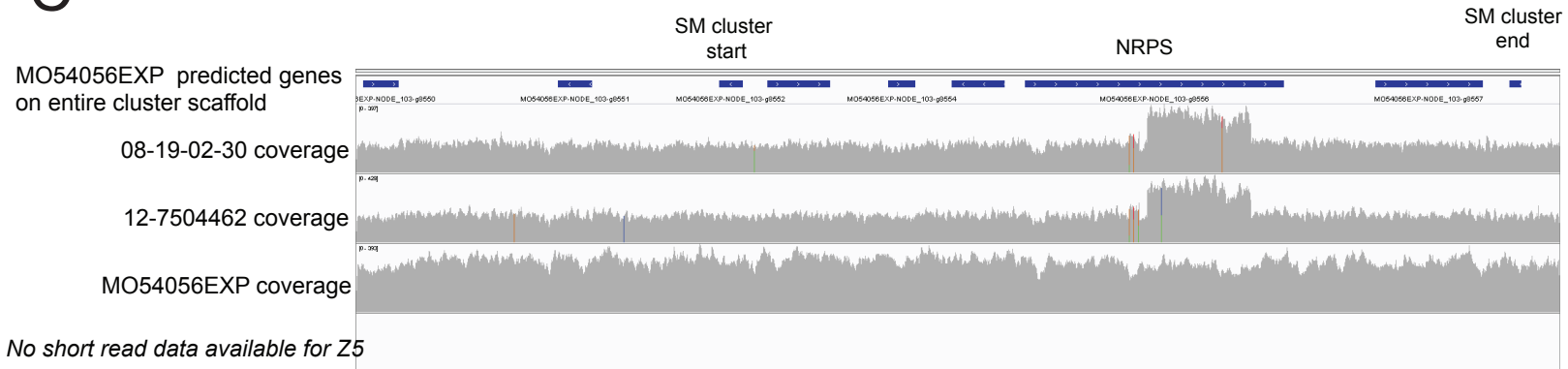

Supplement: S4 Fig — (A) Synteny between a novel PKS-containing cluster in 2 strains with an SM gene cluster in Metarhizium anisopliae. This novel PKS cluster is located between transposable elements in a region syntenic with the reference Af293 chromosome 4. (B) Novel SM gene cluster in MO54056EXP and 3 additional strains. This cluster is only located on 1 scaffold in MO540556EXP and is fragmented across the other strains (ends of scaffolds are marked). (C) Coverage data from short-read alignments for MO54056EXP, 12–7504462, and 08-19-02-30 relative to the MO54056EXP scaffold containing the novel SM gene cluster. PKS, polyketide synthase; SM, secondary metabolite. (PDF) [file pbio.2003583.s004.pdf]

A

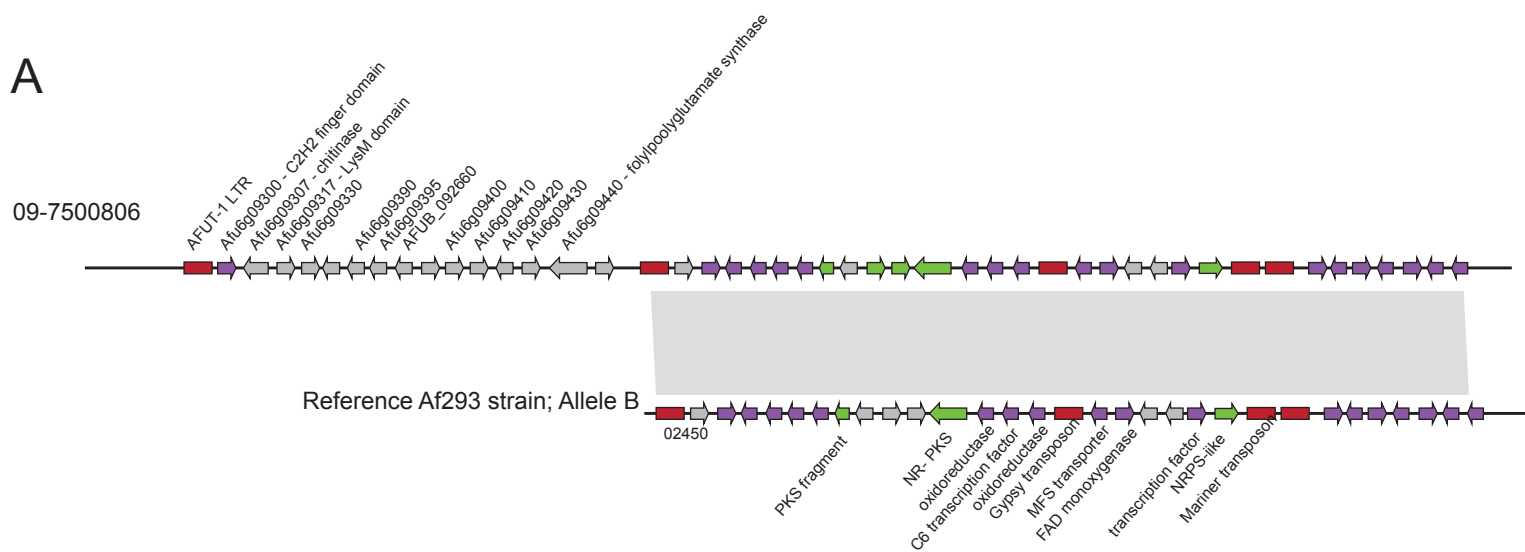

B

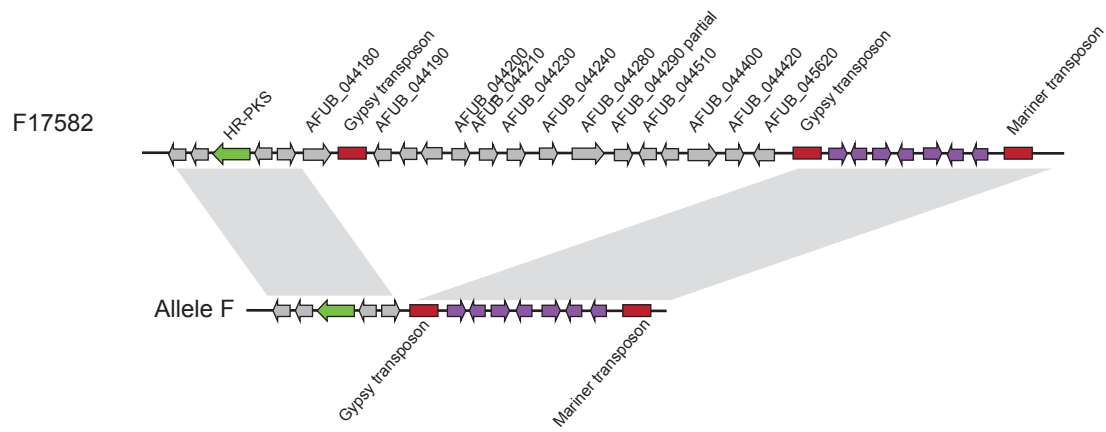

Supplement: S6 Fig — (A) This allele contains an insertion of genes from chromosome 6 immediately upstream of allele C (see main text Fig 4). None of these genes is likely an SM gene cluster backbone gene. An additional transposable element is found flanking this insertion. (B) This allele contains an insertion of genes present in the A1163 reference but not in the Af293 reference in the middle of allele A (see main text Fig 4). None of these genes is likely an SM gene cluster backbone gene. One additional transposable element is contained in this insertion. SM, secondary metabolite. (PDF) [file pbio.2003583.s006.pdf]

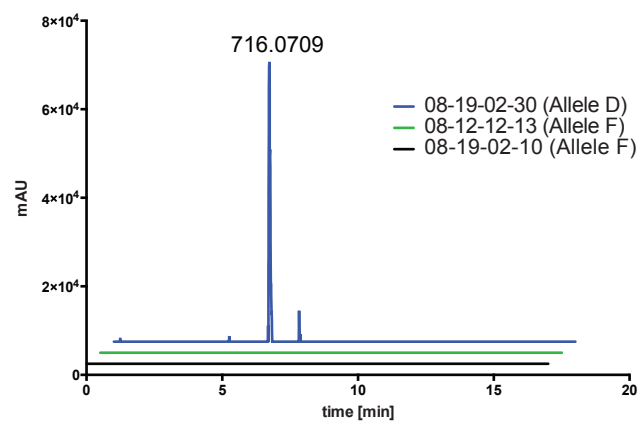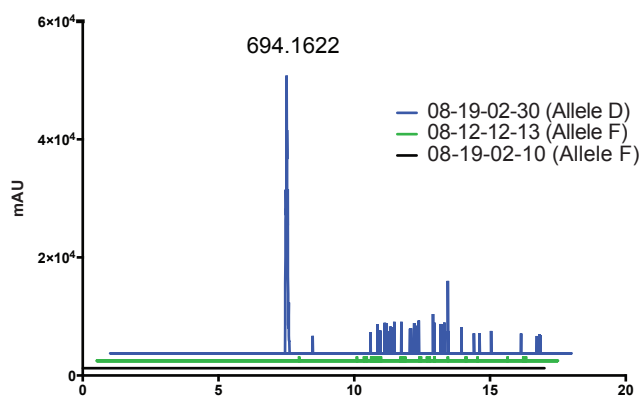

Supplement: S7 Fig — Extracted ion chromatograms for the 2 mass to charge ratios identified in negative mode from XCMS analysis comparing extracts from strains with alleles D and F. (PDF) [file pbio.2003583.s007.pdf]

# A

Aspergillus lentulus:  
GAQ05110.1- GAQ05072.1

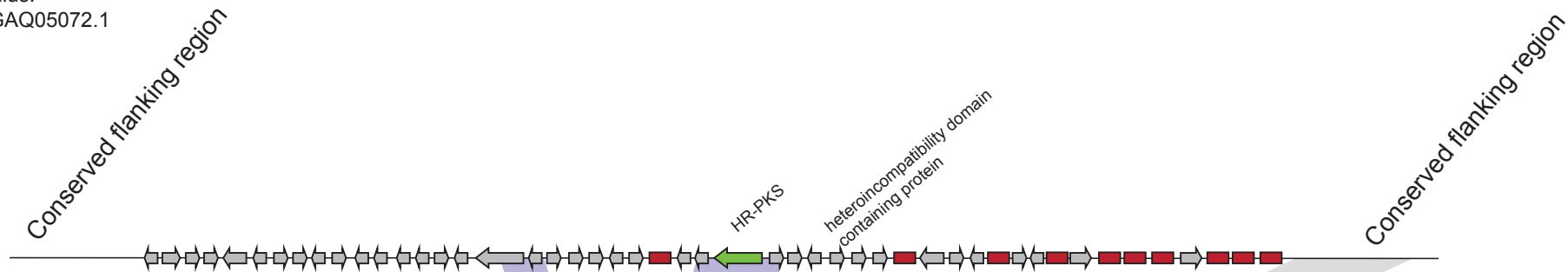

# B

Allele E;  
A1163 & 17 strains

Aspergillus fischeri:  
NFIA\_003580-NFIA\_004090

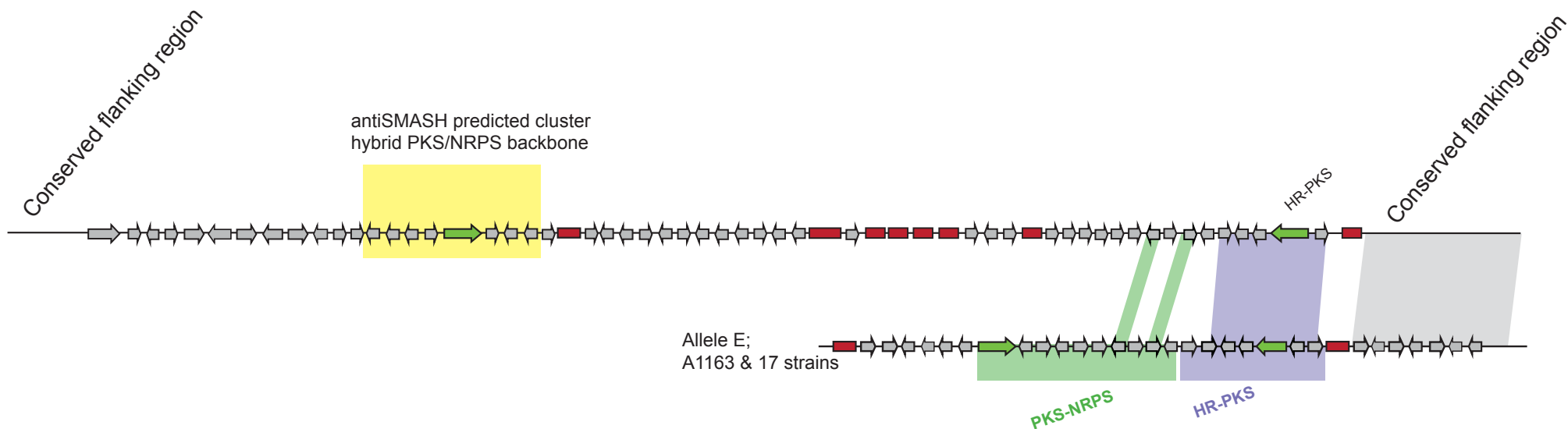

Supplement: S8 Fig — Structure of the idiomorph locus in (A) Aspergillus lentulus and (B) A. fischeri and homology with A. fumigatus allele E (main text Fig 4). Green arrows denote backbone biosynthetic genes and red boxes denote transposable elements as detected by RepeatMasker. A. fischeri contains a novel SM gene cluster not found in A. fumigatus strains. Other genes at this locus have various functions that may not be related to secondary metabolism. A. lentulus contains 1 gene with a heterokaryon incompatibility domain, which may play a role in vegetative incompatibility. SM, secondary metabolite. (PDF) [file pbio.2003583.s008.pdf]

A

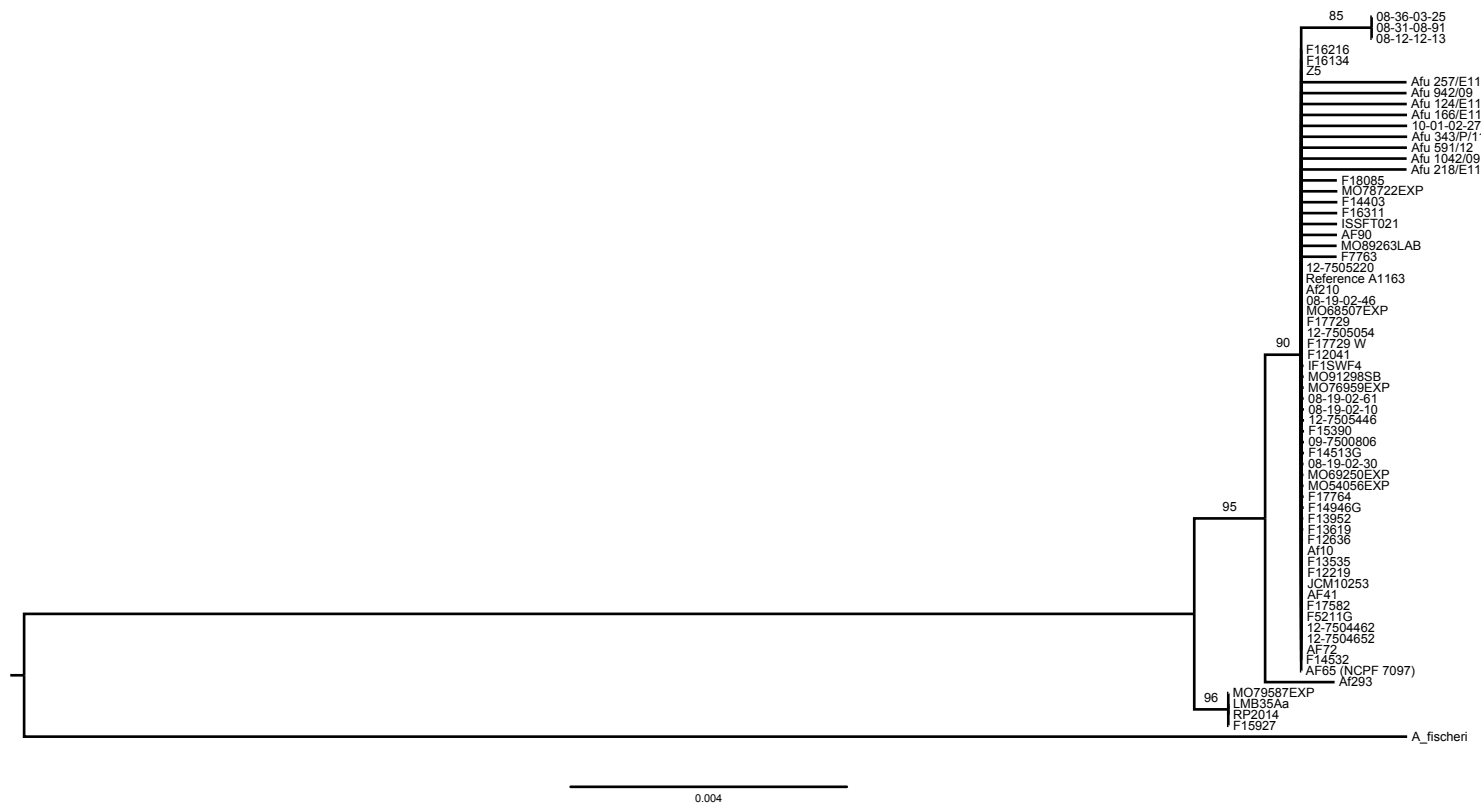

B

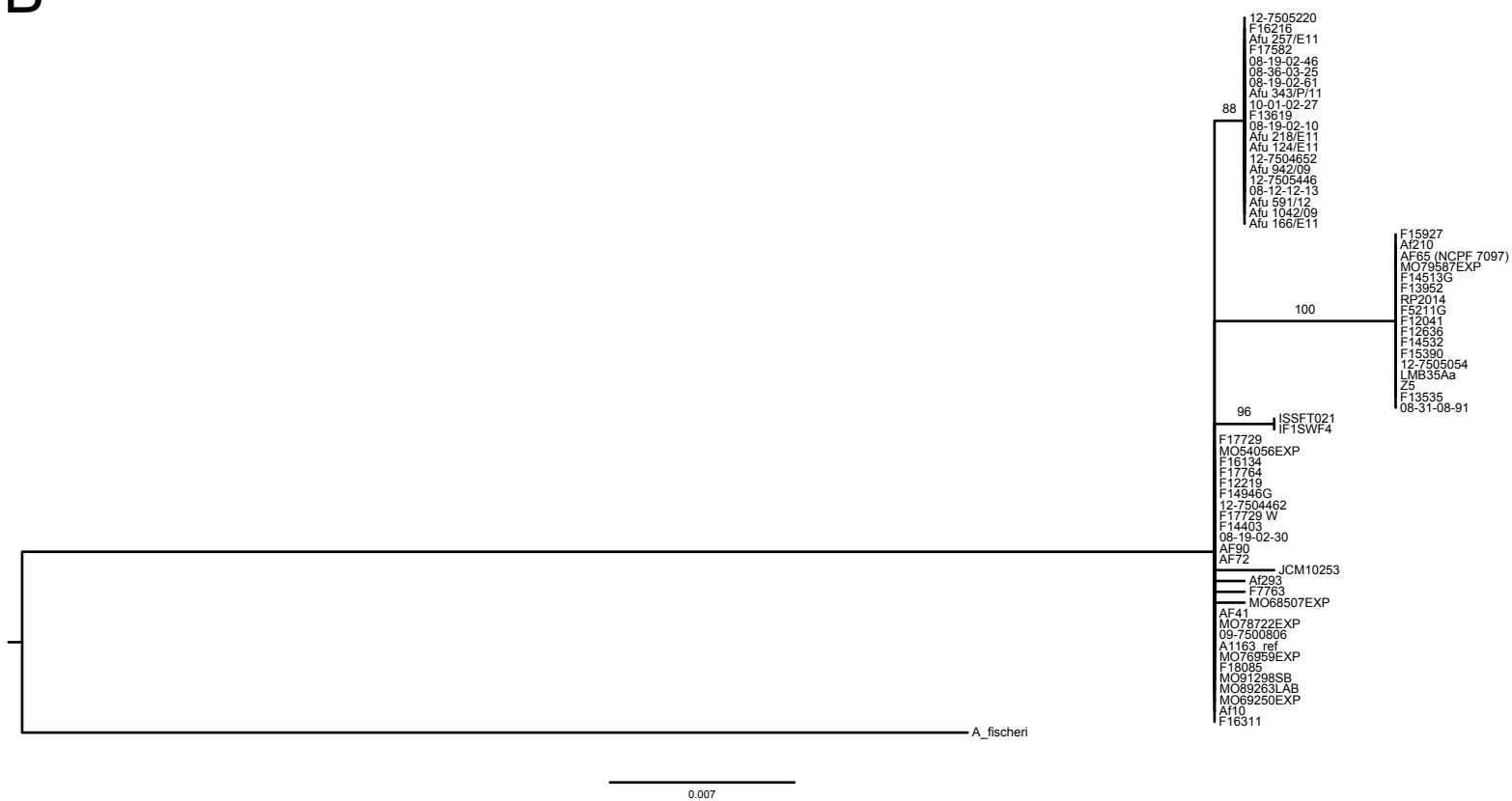

Supplement: S10 Fig — (A) Phylogeny of beta tubulin gene. (B) Phylogeny of calmodulin gene. (PDF) [file pbio.2003583.s010.pdf]
