# Supplementary material for: Drivers of genetic diversity in secondary metabolic gene clusters within a fungal species
Source: PLoS Biol. 2017 Nov 17;15(11):e2003583. doi: 10.1371/journal.pbio.2003583 (PMC5711037; doi:10.1371/journal.pbio.2003583)
Supplement: S2 Fig — These phylogenies are consistent with horizontal transfer between Eurotiomycete and Sordariomycete fungi or with extensive gene loss. SM, secondary metabolite. (PDF) [file pbio.2003583.s002.pdf]

S2A

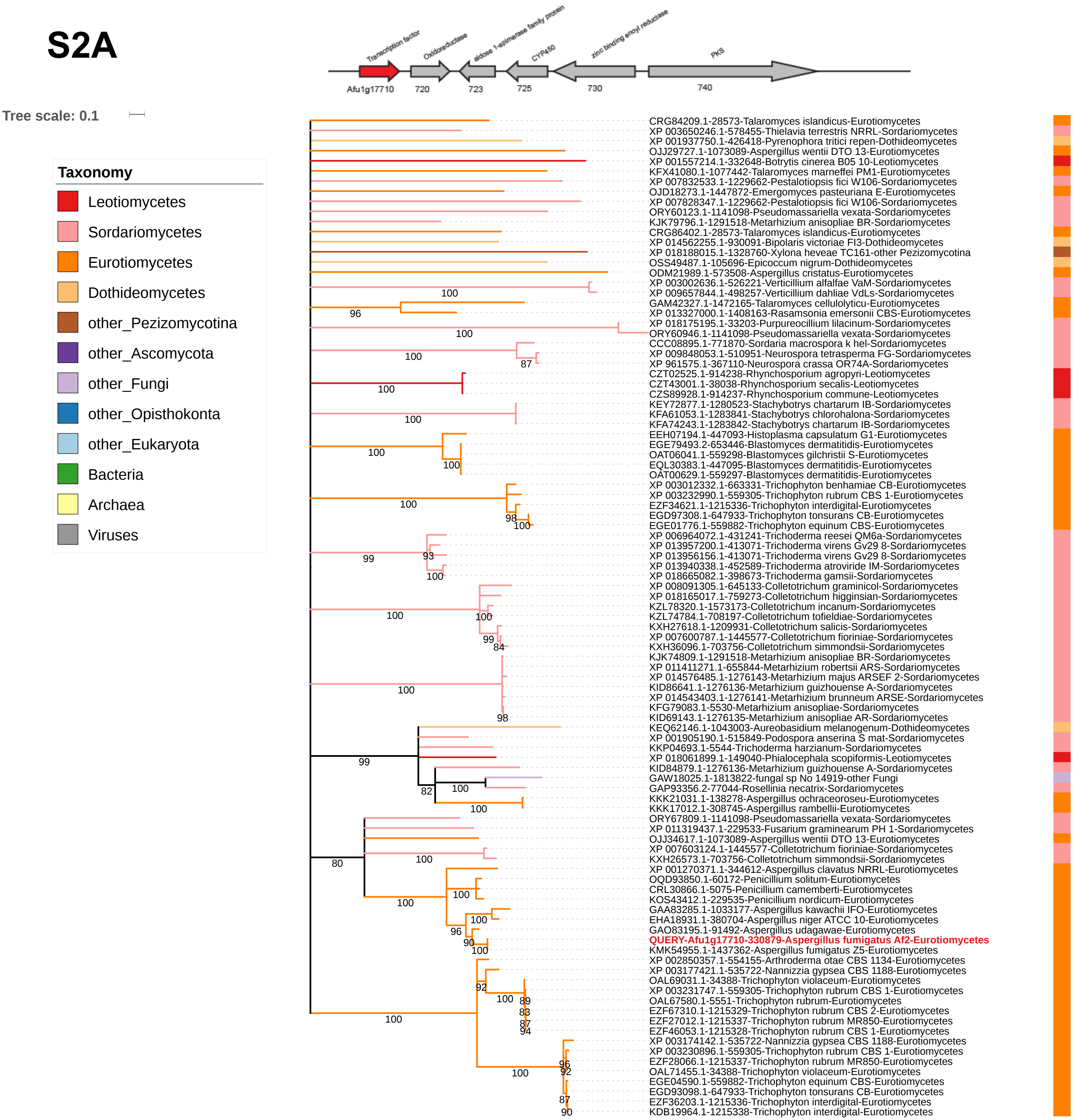

Transcription factor
Oxidoreductase
aldose 1-epimerase family protein
CYP450
zinc binding enoyl reductase
PKS
Afu1g17710
720
723
725
730
740
Tree scale: 0.1
Taxonomy
Leotiomycetes
Sordariomycetes
Eurotiomycetes
Dothideomycetes
other_Pezizomycotina
other_Ascomycota
other_Fungi
other_Opisthokonta
other_Eukaryota
Bacteria
Archaea
Viruses
CRG84209.1-28573-Talaromyces islandicus-Eurotiomycetes
XP 003650246.1-578455-Thielavia terrestris NRRL-Sordariomycetes
XP 001937750.1-426418-Pyrenophora tritici repen-Dothideomycetes
OJJ29727.1-1073089-Aspergillus wentii DTO 13-Eurotiomycetes
XP 001557214.1-332648-Botrytis cinerea B05 10-Leotiomycetes
KFX41080.1-1077442-Talaromyces marneffei PM1-Eurotiomycetes
XP 007832533.1-1229662-Pestalotiopsis fici W106-Sordariomycetes
OJD18273.1-1447872-Emergomyces pasteuriana E-Eurotiomycetes
XP 007828347.1-1229662-Pestalotiopsis fici W106-Sordariomycetes
ORY60123.1-1141098-Pseudomassariella vexata-Sordariomycetes
KJK79796.1-1291518-Metarhizium anisopliae BR-Sordariomycetes
CRG86402.1-28573-Talaromyces islandicus-Eurotiomycetes
XP 014562255.1-930091-Bipolaris victoriae FI3-Dothideomycetes
XP 018188015.1-1328760-Xylona heveae TC161-other Pezizomycotina
OSS49487.1-105696-Epicoccum nigrum-Dothideomycetes
ODM21989.1-573508-Aspergillus cristatus-Eurotiomycetes
XP 003002636.1-526221-Verticillium alfalfae VaM-Sordariomycetes
XP 009657844.1-498257-Verticillium dahliae VdLs-Sordariomycetes
GAM42327.1-1472165-Talaromyces cellulolyticu-Eurotiomycetes
XP 013327000.1-1408163-Rasamsonia emersonii CBS-Eurotiomycetes
XP 018175195.1-33203-Purpureocillium lilacinum-Sordariomycetes
ORY60946.1-1141098-Pseudomassariella vexata-Sordariomycetes
CCC08895.1-771870-Sordaria macrospora k hel-Sordariomycetes
XP 009848053.1-510951-Neurospora tetrasperma FG-Sordariomycetes
XP 961575.1-367110-Neurospora crassa OR74A-Sordariomycetes
CZT02525.1-914238-Rhynchosporium agropyri-Leotiomycetes
CZT43001.1-38038-Rhynchosporium secalis-Leotiomycetes
CZS89928.1-914237-Rhynchosporium commune-Leotiomycetes
KEY72877.1-1280523-Stachybotrys chartarum IB-Sordariomycetes
KFA61053.1-1283841-Stachybotrys chlorohalona-Sordariomycetes
KFA74243.1-1283842-Stachybotrys chartarum IB-Sordariomycetes
EEH07194.1-447093-Histoplasma capsulatum G1-Eurotiomycetes
EGE79493.2-653446-Blastomyces dermatitidis-Eurotiomycetes
OAT06041.1-559298-Blastomyces gilchristii S-Eurotiomycetes
EQL30383.1-447095-Blastomyces dermatitidis-Eurotiomycetes
OAT00629.1-559297-Blastomyces dermatitidis-Eurotiomycetes
XP 003012332.1-663331-Trichophyton benhamiae CB-Eurotiomycetes
XP 003232990.1-559305-Trichophyton rubrum CBS 1-Eurotiomycetes
EZF34621.1-1215336-Trichophyton interdigital-Eurotiomycetes
EGD97308.1-647933-Trichophyton tonsurans CB-Eurotiomycetes
EGE01776.1-559882-Trichophyton equinum CBS-Eurotiomycetes
XP 006964072.1-431241-Trichoderma reesei QM6a-Sordariomycetes
XP 013957200.1-413071-Trichoderma virens Gv29 8-Sordariomycetes
XP 013956156.1-413071-Trichoderma virens Gv29 8-Sordariomycetes
XP 013940338.1-452589-Trichoderma atroviride IM-Sordariomycetes
XP 018665082.1-398673-Trichoderma gamsii-Sordariomycetes
XP 008091305.1-645133-Colletotrichum graminicol-Sordariomycetes
XP 018165017.1-759273-Colletotrichum higginsian-Sordariomycetes
KZL78320.1-1573173-Colletotrichum incanum-Sordariomycetes
KZL74784.1-708197-Colletotrichum tofieldiae-Sordariomycetes
KXH27618.1-1209931-Colletotrichum salicis-Sordariomycetes
XP 007600787.1-1445577-Colletotrichum fioriniae-Sordariomycetes
KXH36096.1-703756-Colletotrichum simmondsii-Sordariomycetes
KJK74809.1-1291518-Metarhizium anisopliae BR-Sordariomycetes
XP 011411271.1-655844-Metarhizium robertsii ARS-Sordariomycetes
XP 014576485.1-1276143-Metarhizium majus ARSEF 2-Sordariomycetes
KID86641.1-1276136-Metarhizium guizhouense A-Sordariomycetes
XP 014543403.1-1276141-Metarhizium brunneum ARSE-Sordariomycetes
KFG79083.1-5530-Metarhizium anisopliae-Sordariomycetes
KID69143.1-1276135-Metarhizium anisopliae AR-Sordariomycetes
KEQ62146.1-1043003-Aureobasidium melanogenum-Dothideomycetes
XP 001905190.1-515849-Podospora anserina S mat-Sordariomycetes
KKP04693.1-5544-Trichoderma harzianum-Sordariomycetes
XP 018061899.1-149040-Phialocephala scopiformis-Leotiomycetes
KID84879.1-1276136-Metarhizium guizhouense A-Sordariomycetes
GAW18025.1-1813822-fungal sp No 14919-other Fungi
GAP93356.2-77044-Rosellinia necatrix-Sordariomycetes
KKK21031.1-138278-Aspergillus ochraceoroseu-Eurotiomycetes
KKK17012.1-308745-Aspergillus rambellii-Eurotiomycetes
ORY67809.1-1141098-Pseudomassariella vexata-Sordariomycetes
XP 011319437.1-229533-Fusarium graminearum PH 1-Sordariomycetes
OJJ34617.1-1073089-Aspergillus wentii DTO 13-Eurotiomycetes
XP 007603124.1-1445577-Colletotrichum fioriniae-Sordariomycetes
KXH26573.1-703756-Colletotrichum simmondsii-Sordariomycetes
XP 001270371.1-344612-Aspergillus clavatus NRRL-Eurotiomycetes
OQD93850.1-60172-Penicillium solitum-Eurotiomycetes
CRL30866.1-5075-Penicillium camemberti-Eurotiomycetes
KOS43412.1-229535-Penicillium nordicum-Eurotiomycetes
GAA83285.1-1033177-Aspergillus kawachii IFO-Eurotiomycetes
EHA18931.1-380704-Aspergillus niger ATCC 10-Eurotiomycetes
GAO83195.1-91492-Aspergillus udagawae-Eurotiomycetes
QUERY-Afu1g17710-330879-Aspergillus fumigatus Af2-Eurotiomycetes
KMK54955.1-1437362-Aspergillus fumigatus Z5-Eurotiomycetes
XP 002850357.1-554155-Arthroderma otae CBS 1134-Eurotiomycetes
XP 003177421.1-535722-Nannizzia gypsea CBS 1188-Eurotiomycetes
OAL69031.1-34388-Trichophyton violaceum-Eurotiomycetes
XP 003231747.1-559305-Trichophyton rubrum CBS 1-Eurotiomycetes
OAL67580.1-5551-Trichophyton rubrum-Eurotiomycetes
EZF67310.1-1215329-Trichophyton rubrum CBS 2-Eurotiomycetes
EZF27012.1-1215337-Trichophyton rubrum MR850-Eurotiomycetes
EZF46053.1-1215328-Trichophyton rubrum CBS 1-Eurotiomycetes
XP 003174142.1-535722-Nannizzia gypsea CBS 1188-Eurotiomycetes
XP 003230896.1-559305-Trichophyton rubrum CBS 1-Eurotiomycetes
EZF28066.1-1215337-Trichophyton rubrum MR850-Eurotiomycetes
OAL71455.1-34388-Trichophyton violaceum-Eurotiomycetes
EGE04590.1-559882-Trichophyton equinum CBS-Eurotiomycetes
EGD93098.1-647933-Trichophyton tonsurans CB-Eurotiomycetes
EZF36203.1-1215336-Trichophyton interdigital-Eurotiomycetes
KDB19964.1-1215338-Trichophyton interdigital-Eurotiomycetes

S2B

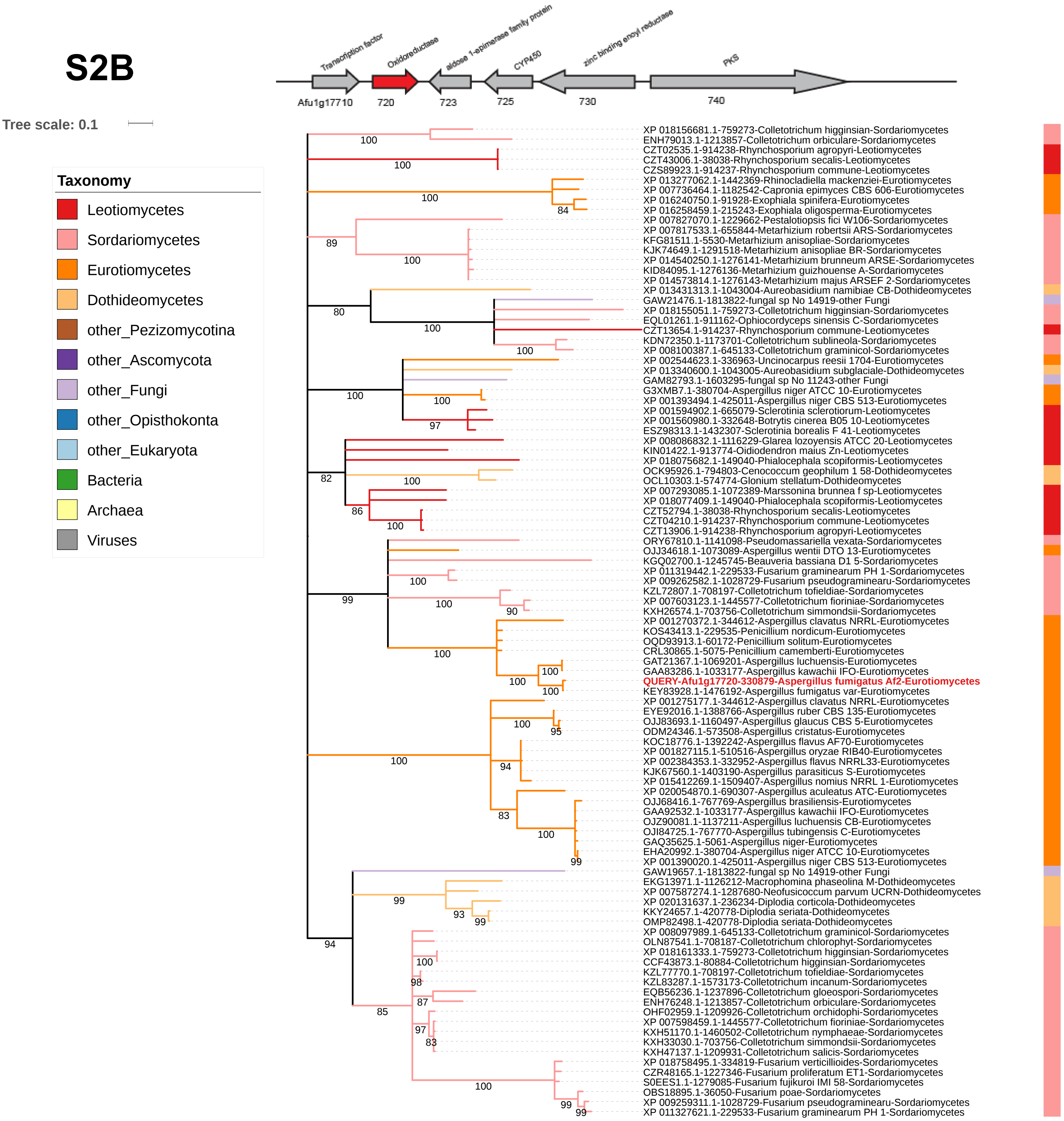

Transcription factor
Oxidoreductase
aldose 1-epimerase family protein
CYP450
zinc binding enoyl reductase
PKS
Afu1g17710
720
723
725
730
740
Tree scale: 0.1
Taxonomy
Leotiomycetes
Sordariomycetes
Eurotiomycetes
Dothideomycetes
other_Pezizomycotina
other_Ascomycota
other_Fungi
other_Opisthokonta
other_Eukaryota
Bacteria
Archaea
Viruses
XP 018156681.1-759273-Colletotrichum higginsian-Sordariomycetes
ENH79013.1-1213857-Colletotrichum orbiculare-Sordariomycetes
CZT02535.1-914238-Rhynchosporium agropyri-Leotiomycetes
CZT43006.1-38038-Rhynchosporium secalis-Leotiomycetes
CZS89923.1-914237-Rhynchosporium commune-Leotiomycetes
XP 013277062.1-1442369-Rhinocladiella mackenziei-Eurotiomycetes
XP 007736464.1-1182542-Capronia epimyces CBS 606-Eurotiomycetes
XP 016240750.1-91928-Exophiala spinifera-Eurotiomycetes
XP 016258459.1-215243-Exophiala oligosperma-Eurotiomycetes
XP 007827070.1-1229662-Pestalotiopsis fici W106-Sordariomycetes
XP 007817533.1-655844-Metarhizium robertsii ARS-Sordariomycetes
KFG81511.1-5530-Metarhizium anisopliae-Sordariomycetes
KJK74649.1-1291518-Metarhizium anisopliae BR-Sordariomycetes
XP 014540250.1-1276141-Metarhizium brunneum ARSE-Sordariomycetes
KID84095.1-1276136-Metarhizium guizhouense A-Sordariomycetes
XP 014573814.1-1276143-Metarhizium majus ARSEF 2-Sordariomycetes
XP 013431313.1-1043004-Aureobasidium namibiae CB-Dothideomycetes
GAW21476.1-1813822-fungal sp No 14919-other Fungi
XP 018155051.1-759273-Colletotrichum higginsian-Sordariomycetes
EQL01261.1-911162-Ophiocordyceps sinensis C-Sordariomycetes
CZT13654.1-914237-Rhynchosporium commune-Leotiomycetes
KDN72350.1-1173701-Colletotrichum sublineola-Sordariomycetes
XP 008100387.1-645133-Colletotrichum graminicol-Sordariomycetes
XP 002544623.1-336963-Uncinocarpus reesii 1704-Eurotiomycetes
XP 013340600.1-1043005-Aureobasidium subglaciale-Dothideomycetes
GAM82793.1-1603295-fungal sp No 11243-other Fungi
G3XMB7.1-380704-Aspergillus niger ATCC 10-Eurotiomycetes
XP 001393494.1-425011-Aspergillus niger CBS 513-Eurotiomycetes
XP 001594902.1-665079-Sclerotinia sclerotiorum-Leotiomycetes
XP 001560980.1-332648-Botrytis cinerea B05 10-Leotiomycetes
ESZ98313.1-1432307-Sclerotinia borealis F 41-Leotiomycetes
XP 008086832.1-1116229-Glarea lozoyensis ATCC 20-Leotiomycetes
KIN01422.1-913774-Oidiodendron maius Zn-Leotiomycetes
XP 018075682.1-149040-Phialocephala scopiformis-Leotiomycetes
OCK95926.1-794803-Cenococcum geophilum 1 58-Dothideomycetes
OCL10303.1-574774-Glonium stellatum-Dothideomycetes
XP 007293085.1-1072389-Marssonina brunnea f sp-Leotiomycetes
XP 018077409.1-149040-Phialocephala scopiformis-Leotiomycetes
CZT52794.1-38038-Rhynchosporium secalis-Leotiomycetes
CZT04210.1-914237-Rhynchosporium commune-Leotiomycetes
CZT13906.1-914238-Rhynchosporium agropyri-Leotiomycetes
ORY67810.1-1141098-Pseudomassariella vexata-Sordariomycetes
OJJ34618.1-1073089-Aspergillus wentii DTO 13-Eurotiomycetes
KGQ02700.1-1245745-Beauveria bassiana D1 5-Sordariomycetes
XP 011319442.1-229533-Fusarium graminearum PH 1-Sordariomycetes
XP 009262582.1-1028729-Fusarium pseudograminearu-Sordariomycetes
KZL72807.1-708197-Colletotrichum tofieldiae-Sordariomycetes
XP 007603123.1-1445577-Colletotrichum fioriniae-Sordariomycetes
KXH26574.1-703756-Colletotrichum simmondsii-Sordariomycetes
XP 001270372.1-344612-Aspergillus clavatus NRRL-Eurotiomycetes
KOS43413.1-229535-Penicillium nordicum-Eurotiomycetes
OQD93913.1-60172-Penicillium solitum-Eurotiomycetes
CRL30865.1-5075-Penicillium camemberti-Eurotiomycetes
GAT21367.1-1069201-Aspergillus luchuensis-Eurotiomycetes
GAA83286.1-1033177-Aspergillus kawachii IFO-Eurotiomycetes
QUERY-Afu1g17720-330879-Aspergillus fumigatus Af2-Eurotiomycetes
KEY83928.1-1476192-Aspergillus fumigatus var-Eurotiomycetes
XP 001275177.1-344612-Aspergillus clavatus NRRL-Eurotiomycetes
EYE92016.1-1388766-Aspergillus ruber CBS 135-Eurotiomycetes
OJJ83693.1-1160497-Aspergillus glaucus CBS 5-Eurotiomycetes
ODM24346.1-573508-Aspergillus cristatus-Eurotiomycetes
KOC18776.1-1392242-Aspergillus flavus AF70-Eurotiomycetes
XP 001827115.1-510516-Aspergillus oryzae RIB40-Eurotiomycetes
XP 002384353.1-332952-Aspergillus flavus NRRL33-Eurotiomycetes
KJK67560.1-1403190-Aspergillus parasiticus S-Eurotiomycetes
XP 015412269.1-1509407-Aspergillus nomius NRRL 1-Eurotiomycetes
XP 020054870.1-690307-Aspergillus aculeatus ATC-Eurotiomycetes
OJJ68416.1-767769-Aspergillus brasiliensis-Eurotiomycetes
GAA92532.1-1033177-Aspergillus kawachii IFO-Eurotiomycetes
OJZ90081.1-1137211-Aspergillus luchuensis CB-Eurotiomycetes
OJI84725.1-767770-Aspergillus tubingensis C-Eurotiomycetes
GAQ35625.1-5061-Aspergillus niger-Eurotiomycetes
EHA20992.1-380704-Aspergillus niger ATCC 10-Eurotiomycetes
XP 001390020.1-425011-Aspergillus niger CBS 513-Eurotiomycetes
GAW19657.1-1813822-fungal sp No 14919-other Fungi
EKG13971.1-1126212-Macrophomina phaseolina M-Dothideomycetes
XP 007587274.1-1287680-Neofusicoccum parvum UCRN-Dothideomycetes
XP 020131637.1-236234-Diplodia corticola-Dothideomycetes
KKY24657.1-420778-Diplodia seriata-Dothideomycetes
OMP82498.1-420778-Diplodia seriata-Dothideomycetes
XP 008097989.1-645133-Colletotrichum graminicol-Sordariomycetes
OLN87541.1-708187-Colletotrichum chlorophyt-Sordariomycetes
XP 018161333.1-759273-Colletotrichum higginsian-Sordariomycetes
CCF43873.1-80884-Colletotrichum higginsian-Sordariomycetes
KZL77770.1-708197-Colletotrichum tofieldiae-Sordariomycetes
KZL83287.1-1573173-Colletotrichum incanum-Sordariomycetes
EQB56236.1-1237896-Colletotrichum gloeospori-Sordariomycetes
ENH76248.1-1213857-Colletotrichum orbiculare-Sordariomycetes
OHF02959.1-1209926-Colletotrichum orchidophi-Sordariomycetes
XP 007598459.1-1445577-Colletotrichum fioriniae-Sordariomycetes
KXH51170.1-1460502-Colletotrichum nymphaeae-Sordariomycetes
KXH33030.1-703756-Colletotrichum simmondsii-Sordariomycetes
KXH47137.1-1209931-Colletotrichum salicis-Sordariomycetes
XP 018758495.1-334819-Fusarium verticillioides-Sordariomycetes
CZR48165.1-1227346-Fusarium proliferatum ET1-Sordariomycetes
S0EES1.1-1279085-Fusarium fujikuroi IMI 58-Sordariomycetes
OBS18895.1-36050-Fusarium poae-Sordariomycetes
XP 009259311.1-1028729-Fusarium pseudograminearu-Sordariomycetes
XP 011327621.1-229533-Fusarium graminearum PH 1-Sordariomycetes

S2C

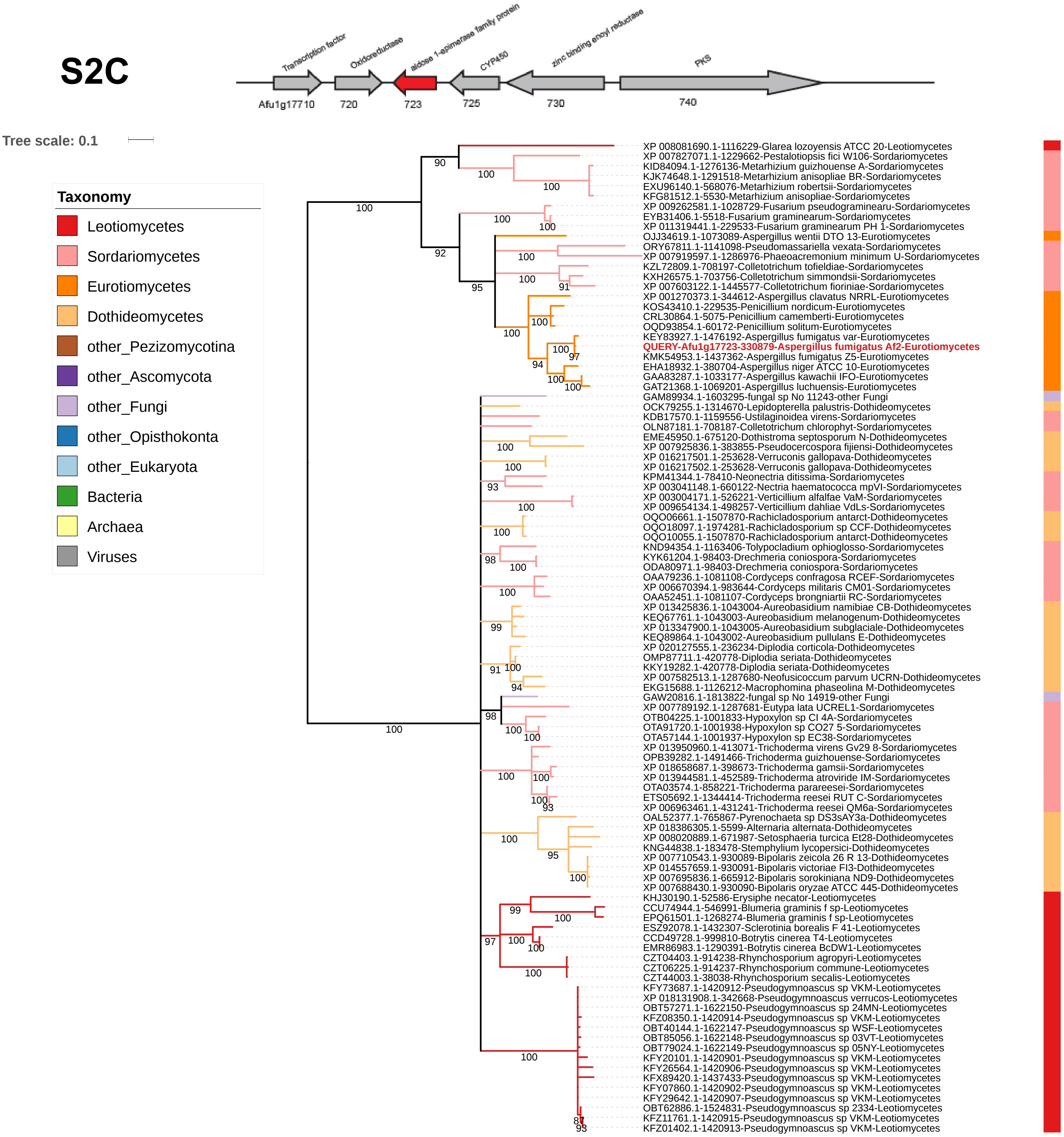
Transcription factor
Oxidoreductase
aldose 1-epimerase family protein
CYP450
zinc binding enoyl reductase
PKS
Afu1g17710
720
723
725
730
740
Tree scale: 0.1
Taxonomy
Leotiomycetes
Sordariomycetes
Eurotiomycetes
Dothideomycetes
other_Pezizomycotina
other_Ascomycota
other_Fungi
other_Opisthokonta
other_Eukaryota
Bacteria
Archaea
Viruses
XP 008081690.1-1116229-Glarea lozoyensis ATCC 20-Leotiomycetes
XP 007827071.1-1229662-Pestalotiopsis fici W106-Sordariomycetes
KID84094.1-1276136-Metarhizium guizhouense A-Sordariomycetes
KJK74648.1-1291518-Metarhizium anisopliae BR-Sordariomycetes
EXU96140.1-568076-Metarhizium robertsii-Sordariomycetes
KFG81512.1-5530-Metarhizium anisopliae-Sordariomycetes
XP 009262581.1-1028729-Fusarium pseudograminearu-Sordariomycetes
EYB31406.1-5518-Fusarium graminearum-Sordariomycetes
XP 011319441.1-229533-Fusarium graminearum PH 1-Sordariomycetes
OJJ34619.1-1073089-Aspergillus wentii DTO 13-Eurotiomycetes
ORY67811.1-1141098-Pseudomassariella vexata-Sordariomycetes
XP 007919597.1-1286976-Phaeoacremonium minimum U-Sordariomycetes
KZL72809.1-708197-Colletotrichum tofieldiae-Sordariomycetes
KXH26575.1-703756-Colletotrichum simmondsii-Sordariomycetes
XP 007603122.1-1445577-Colletotrichum fioriniae-Sordariomycetes
XP 001270373.1-344612-Aspergillus clavatus NRRL-Eurotiomycetes
KOS43410.1-229535-Penicillium nordicum-Eurotiomycetes
CRL30864.1-5075-Penicillium camemberti-Eurotiomycetes
OQD93854.1-60172-Penicillium solitum-Eurotiomycetes
KEY83927.1-1476192-Aspergillus fumigatus var-Eurotiomycetes
QUERY-Afu1g17723-330879-Aspergillus fumigatus Af2-Eurotiomycetes
KMK54953.1-1437362-Aspergillus fumigatus Z5-Eurotiomycetes
EHA18932.1-380704-Aspergillus niger ATCC 10-Eurotiomycetes
GAA83287.1-1033177-Aspergillus kawachii IFO-Eurotiomycetes
GAT21368.1-1069201-Aspergillus luchuensis-Eurotiomycetes
GAM89934.1-1603295-fungal sp No 11243-other Fungi
OCK79255.1-1314670-Lepidopterella palustris-Dothideomycetes
KDB17570.1-1159556-Ustilaginoidea virens-Sordariomycetes
OLN87181.1-708187-Colletotrichum chlorophyt-Sordariomycetes
EME45950.1-675120-Dothistroma septosporum N-Dothideomycetes
XP 007925836.1-383855-Pseudocercospora fijiensi-Dothideomycetes
XP 016217501.1-253628-Verruconis gallopava-Dothideomycetes
XP 016217502.1-253628-Verruconis gallopava-Dothideomycetes
KPM41344.1-78410-Neonectria ditissima-Sordariomycetes
XP 003041148.1-660122-Nectria haematococca mpVI-Sordariomycetes
XP 003004171.1-526221-Verticillium alfalfae VaM-Sordariomycetes
XP 009654134.1-498257-Verticillium dahliae VdLs-Sordariomycetes
OQO06661.1-1507870-Rachicladosporium antarct-Dothideomycetes
OQO18097.1-1974281-Rachicladosporium sp CCF-Dothideomycetes
OQO10055.1-1507870-Rachicladosporium antarct-Dothideomycetes
KND94354.1-1163406-Tolypocladium ophioglosso-Sordariomycetes
KYK61204.1-98403-Drechmeria coniospora-Sordariomycetes
ODA80971.1-98403-Drechmeria coniospora-Sordariomycetes
OAA79236.1-1081108-Cordyceps confragosa RCEF-Sordariomycetes
XP 006670394.1-983644-Cordyceps militaris CM01-Sordariomycetes
OAA52451.1-1081107-Cordyceps brongniartii RC-Sordariomycetes
XP 013425836.1-1043004-Aureobasidium namibiae CB-Dothideomycetes
KEQ67761.1-1043003-Aureobasidium melanogenum-Dothideomycetes
XP 013347900.1-1043005-Aureobasidium subglaciale-Dothideomycetes
KEQ89864.1-1043002-Aureobasidium pullulans E-Dothideomycetes
XP 020127555.1-236234-Diplodia corticola-Dothideomycetes
OMP87711.1-420778-Diplodia seriata-Dothideomycetes
KKY19282.1-420778-Diplodia seriata-Dothideomycetes
XP 007582513.1-1287680-Neofusicoccum parvum UCRN-Dothideomycetes
EKG15688.1-1126212-Macrophomina phaseolina M-Dothideomycetes
GAW20816.1-1813822-fungal sp No 14919-other Fungi
XP 007789192.1-1287681-Eutypa lata UCREL1-Sordariomycetes
OTB04225.1-1001833-Hypoxylon sp CI 4A-Sordariomycetes
OTA91720.1-1001938-Hypoxylon sp CO27 5-Sordariomycetes
OTA57144.1-1001937-Hypoxylon sp EC38-Sordariomycetes
XP 013950960.1-413071-Trichoderma virens Gv29 8-Sordariomycetes
OPB39282.1-1491466-Trichoderma guizhouense-Sordariomycetes
XP 018658687.1-398673-Trichoderma gamsii-Sordariomycetes
XP 013944581.1-452589-Trichoderma atroviride IM-Sordariomycetes
OTA03574.1-858221-Trichoderma parareesei-Sordariomycetes
ETS05692.1-1344414-Trichoderma reesei RUT C-Sordariomycetes
XP 006963461.1-431241-Trichoderma reesei QM6a-Sordariomycetes
OAL52377.1-765867-Pyrenochaeta sp DS3sAY3a-Dothideomycetes
XP 018386305.1-5599-Alternaria alternata-Dothideomycetes
XP 008020889.1-671987-Setosphaeria turcica Et28-Dothideomycetes
KNG44838.1-183478-Stemphylium lycopersici-Dothideomycetes
XP 007710543.1-930089-Bipolaris zeicola 26 R 13-Dothideomycetes
XP 014557659.1-930091-Bipolaris victoriae FI3-Dothideomycetes
XP 007695836.1-665912-Bipolaris sorokiniana ND9-Dothideomycetes
XP 007688430.1-930090-Bipolaris oryzae ATCC 445-Dothideomycetes
KHJ30190.1-52586-Erysiphe necator-Leotiomycetes
CCU74944.1-546991-Blumeria graminis f sp-Leotiomycetes
EPQ61501.1-1268274-Blumeria graminis f sp-Leotiomycetes
ESZ92078.1-1432307-Sclerotinia borealis F 41-Leotiomycetes
CCD49728.1-999810-Botrytis cinerea T4-Leotiomycetes
EMR86983.1-1290391-Botrytis cinerea BcDW1-Leotiomycetes
CZT04403.1-914238-Rhynchosporium agropyri-Leotiomycetes
CZT06225.1-914237-Rhynchosporium commune-Leotiomycetes
CZT44003.1-38038-Rhynchosporium secalis-Leotiomycetes
KFY73687.1-1420912-Pseudogymnoascus sp VKM-Leotiomycetes
XP 018131908.1-342668-Pseudogymnoascus verrucos-Leotiomycetes
OBT57271.1-1622150-Pseudogymnoascus sp 24MN-Leotiomycetes
KFZ08350.1-1420914-Pseudogymnoascus sp VKM-Leotiomycetes
OBT40144.1-1622147-Pseudogymnoascus sp WSF-Leotiomycetes
OBT85056.1-1622148-Pseudogymnoascus sp 03VT-Leotiomycetes
OBT79024.1-1622149-Pseudogymnoascus sp 05NY-Leotiomycetes
KFY20101.1-1420901-Pseudogymnoascus sp VKM-Leotiomycetes
KFY26564.1-1420906-Pseudogymnoascus sp VKM-Leotiomycetes
KFX89420.1-1437433-Pseudogymnoascus sp VKM-Leotiomycetes
KFY07860.1-1420902-Pseudogymnoascus sp VKM-Leotiomycetes
KFY29642.1-1420907-Pseudogymnoascus sp VKM-Leotiomycetes
OBT62886.1-1524831-Pseudogymnoascus sp 2334-Leotiomycetes
KFZ11761.1-1420915-Pseudogymnoascus sp VKM-Leotiomycetes
KFZ01402.1-1420913-Pseudogymnoascus sp VKM-Leotiomycetes

S2D

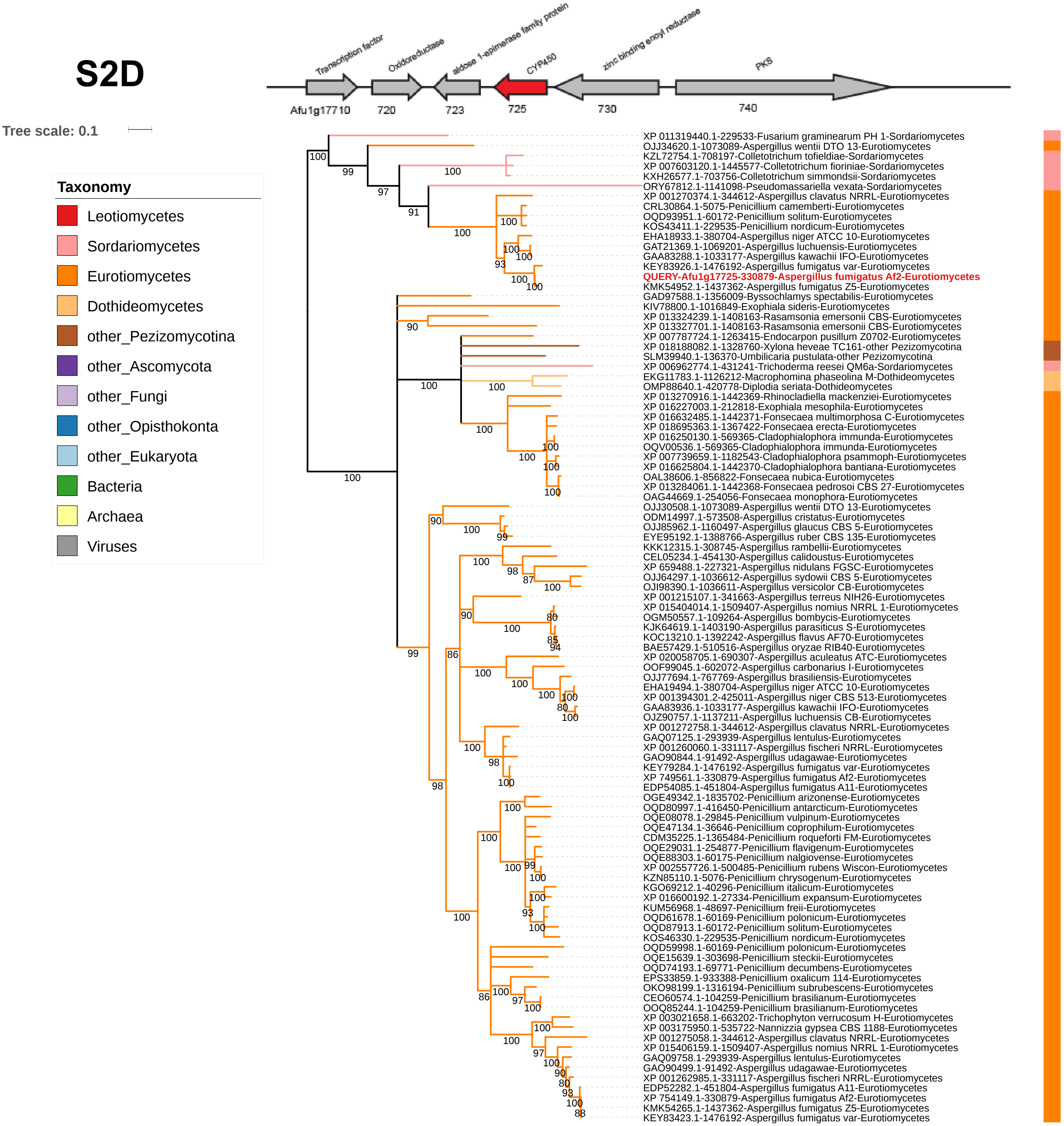
Transcription factor
Oxidoreductase
aldose 1-epimerase family protein
CYP450
zinc binding enoyl reductase
PKS
Afu1g17710
720
723
725
730
740
Tree scale: 0.1
Taxonomy
Leotiomycetes
Sordariomycetes
Eurotiomycetes
Dothideomycetes
other_Pezizomycotina
other_Ascomycota
other_Fungi
other_Opisthokonta
other_Eukaryota
Bacteria
Archaea
Viruses
XP 011319440.1-229533-Fusarium graminearum PH 1-Sordariomycetes
OJJ34620.1-1073089-Aspergillus wentii DTO 13-Eurotiomycetes
KZL72754.1-708197-Colletotrichum tofieldiae-Sordariomycetes
XP 007603120.1-1445577-Colletotrichum fioriniae-Sordariomycetes
KXH26577.1-703756-Colletotrichum simmondsii-Sordariomycetes
ORY67812.1-1141098-Pseudomassariella vexata-Sordariomycetes
XP 001270374.1-344612-Aspergillus clavatus NRRL-Eurotiomycetes
CRL30864.1-5075-Penicillium camemberti-Eurotiomycetes
OQD93951.1-60172-Penicillium solitum-Eurotiomycetes
KOS43411.1-229535-Penicillium nordicum-Eurotiomycetes
EHA18933.1-380704-Aspergillus niger ATCC 10-Eurotiomycetes
GAT21369.1-1069201-Aspergillus luchuensis-Eurotiomycetes
GAA83288.1-1033177-Aspergillus kawachii IFO-Eurotiomycetes
KEY83926.1-1476192-Aspergillus fumigatus var-Eurotiomycetes
QUERY-Afu1g17725-330879-Aspergillus fumigatus Af2-Eurotiomycetes
KMK54952.1-1437362-Aspergillus fumigatus Z5-Eurotiomycetes
GAD97588.1-1356009-Byssochlamys spectabilis-Eurotiomycetes
KIV78800.1-1016849-Exophiala sideris-Eurotiomycetes
XP 013324239.1-1408163-Rasamsonia emersonii CBS-Eurotiomycetes
XP 013327701.1-1408163-Rasamsonia emersonii CBS-Eurotiomycetes
XP 007787724.1-1263415-Endocarpon pusillum Z0702-Eurotiomycetes
XP 018188082.1-1328760-Xylona heveae TC161-other Pezizomycotina
SLM39940.1-136370-Umbilicaria pustulata-other Pezizomycotina
XP 006962774.1-431241-Trichoderma reesei QM6a-Sordariomycetes
EKG11783.1-1126212-Macrophomina phaseolina M-Dothideomycetes
OMP88640.1-420778-Diplodia seriata-Dothideomycetes
XP 013270916.1-1442369-Rhinocladiella mackenziei-Eurotiomycetes
XP 016227003.1-212818-Exophiala mesophila-Eurotiomycetes
XP 016632485.1-1442371-Fonsecaea multimorphosa C-Eurotiomycetes
XP 018695363.1-1367422-Fonsecaea erecta-Eurotiomycetes
XP 016250130.1-569365-Cladophialophora immunda-Eurotiomycetes
OQV00536.1-569365-Cladophialophora immunda-Eurotiomycetes
XP 007739659.1-1182543-Cladophialophora psammoph-Eurotiomycetes
XP 016625804.1-1442370-Cladophialophora bantiana-Eurotiomycetes
OAL38606.1-856822-Fonsecaea nubica-Eurotiomycetes
XP 013284061.1-1442368-Fonsecaea pedrosoi CBS 27-Eurotiomycetes
OAG44669.1-254056-Fonsecaea monophora-Eurotiomycetes
OJJ30508.1-1073089-Aspergillus wentii DTO 13-Eurotiomycetes
ODM14997.1-573508-Aspergillus cristatus-Eurotiomycetes
OJJ85962.1-1160497-Aspergillus glaucus CBS 5-Eurotiomycetes
EYE95192.1-1388766-Aspergillus ruber CBS 135-Eurotiomycetes
KKK12315.1-308745-Aspergillus rambellii-Eurotiomycetes
CEL05234.1-454130-Aspergillus calidoustus-Eurotiomycetes
XP 659488.1-227321-Aspergillus nidulans FGSC-Eurotiomycetes
OJJ64297.1-1036612-Aspergillus sydowii CBS 5-Eurotiomycetes
OJI98390.1-1036611-Aspergillus versicolor CB-Eurotiomycetes
XP 001215107.1-341663-Aspergillus terreus NIH26-Eurotiomycetes
XP 015404014.1-1509407-Aspergillus nomius NRRL 1-Eurotiomycetes
OGM50557.1-109264-Aspergillus bombycis-Eurotiomycetes
KJK64619.1-1403190-Aspergillus parasiticus S-Eurotiomycetes
KOC13210.1-1392242-Aspergillus flavus AF70-Eurotiomycetes
BAE57429.1-510516-Aspergillus oryzae RIB40-Eurotiomycetes
XP 020058705.1-690307-Aspergillus aculeatus ATC-Eurotiomycetes
OOF99045.1-602072-Aspergillus carbonarius I-Eurotiomycetes
OJJ77694.1-767769-Aspergillus brasiliensis-Eurotiomycetes
EHA19494.1-380704-Aspergillus niger ATCC 10-Eurotiomycetes
XP 001394301.2-425011-Aspergillus niger CBS 513-Eurotiomycetes
GAA83936.1-1033177-Aspergillus kawachii IFO-Eurotiomycetes
OJZ90757.1-1137211-Aspergillus luchuensis CB-Eurotiomycetes
XP 001272758.1-344612-Aspergillus clavatus NRRL-Eurotiomycetes
GAQ07125.1-293939-Aspergillus lentulus-Eurotiomycetes
XP 001260060.1-331117-Aspergillus fischeri NRRL-Eurotiomycetes
GAO90844.1-91492-Aspergillus udagawae-Eurotiomycetes
KEY79284.1-1476192-Aspergillus fumigatus var-Eurotiomycetes
XP 749561.1-330879-Aspergillus fumigatus Af2-Eurotiomycetes
EDP54085.1-451804-Aspergillus fumigatus A11-Eurotiomycetes
OGE49342.1-1835702-Penicillium arizonense-Eurotiomycetes
OQD80997.1-416450-Penicillium antarcticum-Eurotiomycetes
OQE08078.1-29845-Penicillium vulpinum-Eurotiomycetes
OQE47134.1-36646-Penicillium coprophilum-Eurotiomycetes
CDM35225.1-1365484-Penicillium roqueforti FM-Eurotiomycetes
OQE29031.1-254877-Penicillium flavigenum-Eurotiomycetes
OQE88303.1-60175-Penicillium nalgiovense-Eurotiomycetes
XP 002557726.1-500485-Penicillium rubens Wiscon-Eurotiomycetes
KZN85110.1-5076-Penicillium chrysogenum-Eurotiomycetes
KGO69212.1-40296-Penicillium italicum-Eurotiomycetes
XP 016600192.1-27334-Penicillium expansum-Eurotiomycetes
KUM56968.1-48697-Penicillium freii-Eurotiomycetes
OQD61678.1-60169-Penicillium polonicum-Eurotiomycetes
OQD87913.1-60172-Penicillium solitum-Eurotiomycetes
KOS46330.1-229535-Penicillium nordicum-Eurotiomycetes
OQD59998.1-60169-Penicillium polonicum-Eurotiomycetes
OQE15639.1-303698-Penicillium steckii-Eurotiomycetes
OQD74193.1-69771-Penicillium decumbens-Eurotiomycetes
EPS33859.1-933388-Penicillium oxalicum 114-Eurotiomycetes
OKO98199.1-1316194-Penicillium subrubescens-Eurotiomycetes
CEO60574.1-104259-Penicillium brasilianum-Eurotiomycetes
OOQ85244.1-104259-Penicillium brasilianum-Eurotiomycetes
XP 003021658.1-663202-Trichophyton verrucosum H-Eurotiomycetes
XP 003175950.1-535722-Nannizzia gypsea CBS 1188-Eurotiomycetes
XP 001275058.1-344612-Aspergillus clavatus NRRL-Eurotiomycetes
XP 015406159.1-1509407-Aspergillus nomius NRRL 1-Eurotiomycetes
GAQ09758.1-293939-Aspergillus lentulus-Eurotiomycetes
GAO90499.1-91492-Aspergillus udagawae-Eurotiomycetes
XP 001262985.1-331117-Aspergillus fischeri NRRL-Eurotiomycetes
EDP52282.1-451804-Aspergillus fumigatus A11-Eurotiomycetes
XP 754149.1-330879-Aspergillus fumigatus Af2-Eurotiomycetes
KMK54265.1-1437362-Aspergillus fumigatus Z5-Eurotiomycetes
KEY83423.1-1476192-Aspergillus fumigatus var-Eurotiomycetes

S2E

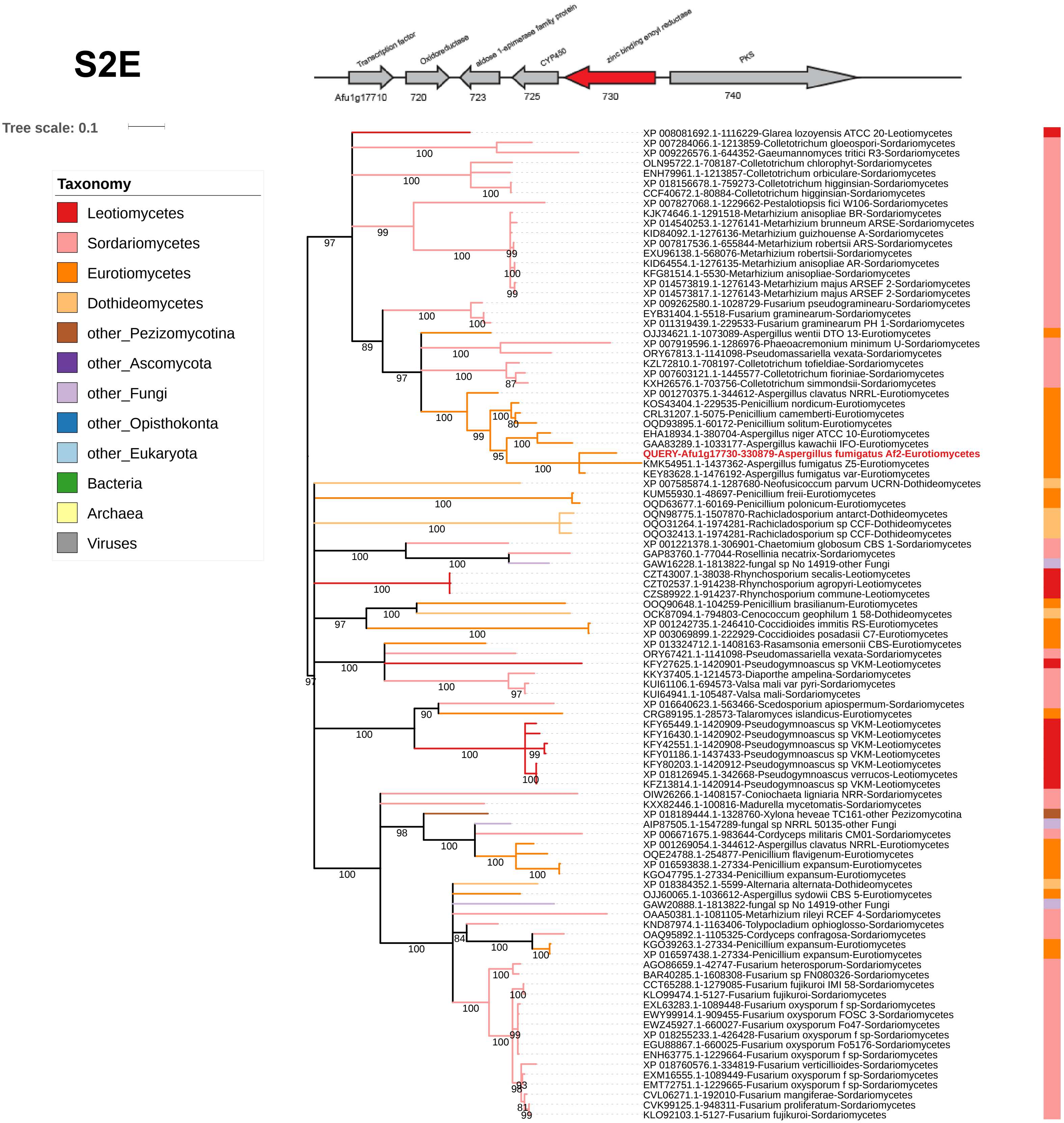

Transcription factor
Oxidoreductase
aldose 1-epimerase family protein
CYP450
zinc binding enoyl reductase
PKS
Afu1g17710
720
723
725
730
740
Tree scale: 0.1
Taxonomy
Leotiomycetes
Sordariomycetes
Eurotiomycetes
Dothideomycetes
other_Pezizomycotina
other_Ascomycota
other_Fungi
other_Opisthokonta
other_Eukaryota
Bacteria
Archaea
Viruses
XP 008081692.1-1116229-Glarea lozoyensis ATCC 20-Leotiomycetes
XP 007284066.1-1213859-Colletotrichum gloeospori-Sordariomycetes
XP 009226576.1-644352-Gaeumannomyces tritici R3-Sordariomycetes
OLN95722.1-708187-Colletotrichum chlorophyt-Sordariomycetes
ENH79961.1-1213857-Colletotrichum orbiculare-Sordariomycetes
XP 018156678.1-759273-Colletotrichum higginsian-Sordariomycetes
CCF40672.1-80884-Colletotrichum higginsian-Sordariomycetes
XP 007827068.1-1229662-Pestalotiopsis fici W106-Sordariomycetes
KJK74646.1-1291518-Metarhizium anisopliae BR-Sordariomycetes
XP 014540253.1-1276141-Metarhizium brunneum ARSE-Sordariomycetes
KID84092.1-1276136-Metarhizium guizhouense A-Sordariomycetes
XP 007817536.1-655844-Metarhizium robertsii ARS-Sordariomycetes
EXU96138.1-568076-Metarhizium robertsii-Sordariomycetes
KID64554.1-1276135-Metarhizium anisopliae AR-Sordariomycetes
KFG81514.1-5530-Metarhizium anisopliae-Sordariomycetes
XP 014573819.1-1276143-Metarhizium majus ARSEF 2-Sordariomycetes
XP 014573817.1-1276143-Metarhizium majus ARSEF 2-Sordariomycetes
XP 009262580.1-1028729-Fusarium pseudograminearu-Sordariomycetes
EYB31404.1-5518-Fusarium graminearum-Sordariomycetes
XP 011319439.1-229533-Fusarium graminearum PH 1-Sordariomycetes
OJJ34621.1-1073089-Aspergillus wentii DTO 13-Eurotiomycetes
XP 007919596.1-1286976-Phaeoacremonium minimum U-Sordariomycetes
ORY67813.1-1141098-Pseudomassariella vexata-Sordariomycetes
KZL72810.1-708197-Colletotrichum tofieldiae-Sordariomycetes
XP 007603121.1-1445577-Colletotrichum fioriniae-Sordariomycetes
KXH26576.1-703756-Colletotrichum simmondsii-Sordariomycetes
XP 001270375.1-344612-Aspergillus clavatus NRRL-Eurotiomycetes
KOS43404.1-229535-Penicillium nordicum-Eurotiomycetes
CRL31207.1-5075-Penicillium camemberti-Eurotiomycetes
OQD93895.1-60172-Penicillium solitum-Eurotiomycetes
EHA18934.1-380704-Aspergillus niger ATCC 10-Eurotiomycetes
GAA83289.1-1033177-Aspergillus kawachii IFO-Eurotiomycetes
QUERY-Afu1g17730-330879-Aspergillus fumigatus Af2-Eurotiomycetes
KMK54951.1-1437362-Aspergillus fumigatus Z5-Eurotiomycetes
KEY83628.1-1476192-Aspergillus fumigatus var-Eurotiomycetes
XP 007585874.1-1287680-Neofusicoccum parvum UCRN-Dothideomycetes
KUM55930.1-48697-Penicillium freii-Eurotiomycetes
OQD63677.1-60169-Penicillium polonicum-Eurotiomycetes
OQN98775.1-1507870-Rachicladosporium antarct-Dothideomycetes
OQO31264.1-1974281-Rachicladosporium sp CCF-Dothideomycetes
OQO32413.1-1974281-Rachicladosporium sp CCF-Dothideomycetes
XP 001221378.1-306901-Chaetomium globosum CBS 1-Sordariomycetes
GAP83760.1-77044-Rosellinia necatrix-Sordariomycetes
GAW16228.1-1813822-fungal sp No 14919-other Fungi
CZT43007.1-38038-Rhynchosporium secalis-Leotiomycetes
CZT02537.1-914238-Rhynchosporium agropyri-Leotiomycetes
CZS89922.1-914237-Rhynchosporium commune-Leotiomycetes
OOQ90648.1-104259-Penicillium brasilianum-Eurotiomycetes
OCK87094.1-794803-Cenococcum geophilum 1 58-Dothideomycetes
XP 001242735.1-246410-Coccidioides immitis RS-Eurotiomycetes
XP 003069899.1-222929-Coccidioides posadasii C7-Eurotiomycetes
XP 013324712.1-1408163-Rasamsonia emersonii CBS-Eurotiomycetes
ORY67421.1-1141098-Pseudomassariella vexata-Sordariomycetes
KFY27625.1-1420901-Pseudogymnoascus sp VKM-Leotiomycetes
KKY37405.1-1214573-Diaporthe ampelina-Sordariomycetes
KUI61106.1-694573-Valsa mali var pyri-Sordariomycetes
KUI64941.1-105487-Valsa mali-Sordariomycetes
XP 016640623.1-563466-Scedosporium apiospermum-Sordariomycetes
CRG89195.1-28573-Talaromyces islandicus-Eurotiomycetes
KFY65449.1-1420909-Pseudogymnoascus sp VKM-Leotiomycetes
KFY16430.1-1420902-Pseudogymnoascus sp VKM-Leotiomycetes
KFY42551.1-1420908-Pseudogymnoascus sp VKM-Leotiomycetes
KFY01186.1-1437433-Pseudogymnoascus sp VKM-Leotiomycetes
KFY80203.1-1420912-Pseudogymnoascus sp VKM-Leotiomycetes
XP 018126945.1-342668-Pseudogymnoascus verrucos-Leotiomycetes
KFZ13814.1-1420914-Pseudogymnoascus sp VKM-Leotiomycetes
OIW26266.1-1408157-Coniochaeta ligniaria NRR-Sordariomycetes
KXX82446.1-100816-Madurella mycetomatis-Sordariomycetes
XP 018189444.1-1328760-Xylona heveae TC161-other Pezizomycotina
AIP87505.1-1547289-fungal sp NRRL 50135-other Fungi
XP 006671675.1-983644-Cordyceps militaris CM01-Sordariomycetes
XP 001269054.1-344612-Aspergillus clavatus NRRL-Eurotiomycetes
OQE24788.1-254877-Penicillium flavigenum-Eurotiomycetes
XP 016593838.1-27334-Penicillium expansum-Eurotiomycetes
KGO47795.1-27334-Penicillium expansum-Eurotiomycetes
XP 018384352.1-5599-Alternaria alternata-Dothideomycetes
OJJ60065.1-1036612-Aspergillus sydowii CBS 5-Eurotiomycetes
GAW20888.1-1813822-fungal sp No 14919-other Fungi
OAA50381.1-1081105-Metarhizium rileyi RCEF 4-Sordariomycetes
KND87974.1-1163406-Tolypocladium ophioglosso-Sordariomycetes
OAQ95892.1-1105325-Cordyceps confragosa-Sordariomycetes
KGO39263.1-27334-Penicillium expansum-Eurotiomycetes
XP 016597438.1-27334-Penicillium expansum-Eurotiomycetes
AGO86659.1-42747-Fusarium heterosporum-Sordariomycetes
BAR40285.1-1608308-Fusarium sp FN080326-Sordariomycetes
CCT65288.1-1279085-Fusarium fujikuroi IMI 58-Sordariomycetes
KLO99474.1-5127-Fusarium fujikuroi-Sordariomycetes
EXL63283.1-1089448-Fusarium oxysporum f sp-Sordariomycetes
EWY99914.1-909455-Fusarium oxysporum FOSC 3-Sordariomycetes
EWZ45927.1-660027-Fusarium oxysporum Fo47-Sordariomycetes
XP 018255233.1-426428-Fusarium oxysporum f sp-Sordariomycetes
EGU88867.1-660025-Fusarium oxysporum Fo5176-Sordariomycetes
ENH63775.1-1229664-Fusarium oxysporum f sp-Sordariomycetes
XP 018760576.1-334819-Fusarium verticillioides-Sordariomycetes
EXM16555.1-1089449-Fusarium oxysporum f sp-Sordariomycetes
EMT72751.1-1229665-Fusarium oxysporum f sp-Sordariomycetes
CVL06271.1-192010-Fusarium mangiferae-Sordariomycetes
CVK99125.1-948311-Fusarium proliferatum-Sordariomycetes
KLO92103.1-5127-Fusarium fujikuroi-Sordariomycetes

S2F

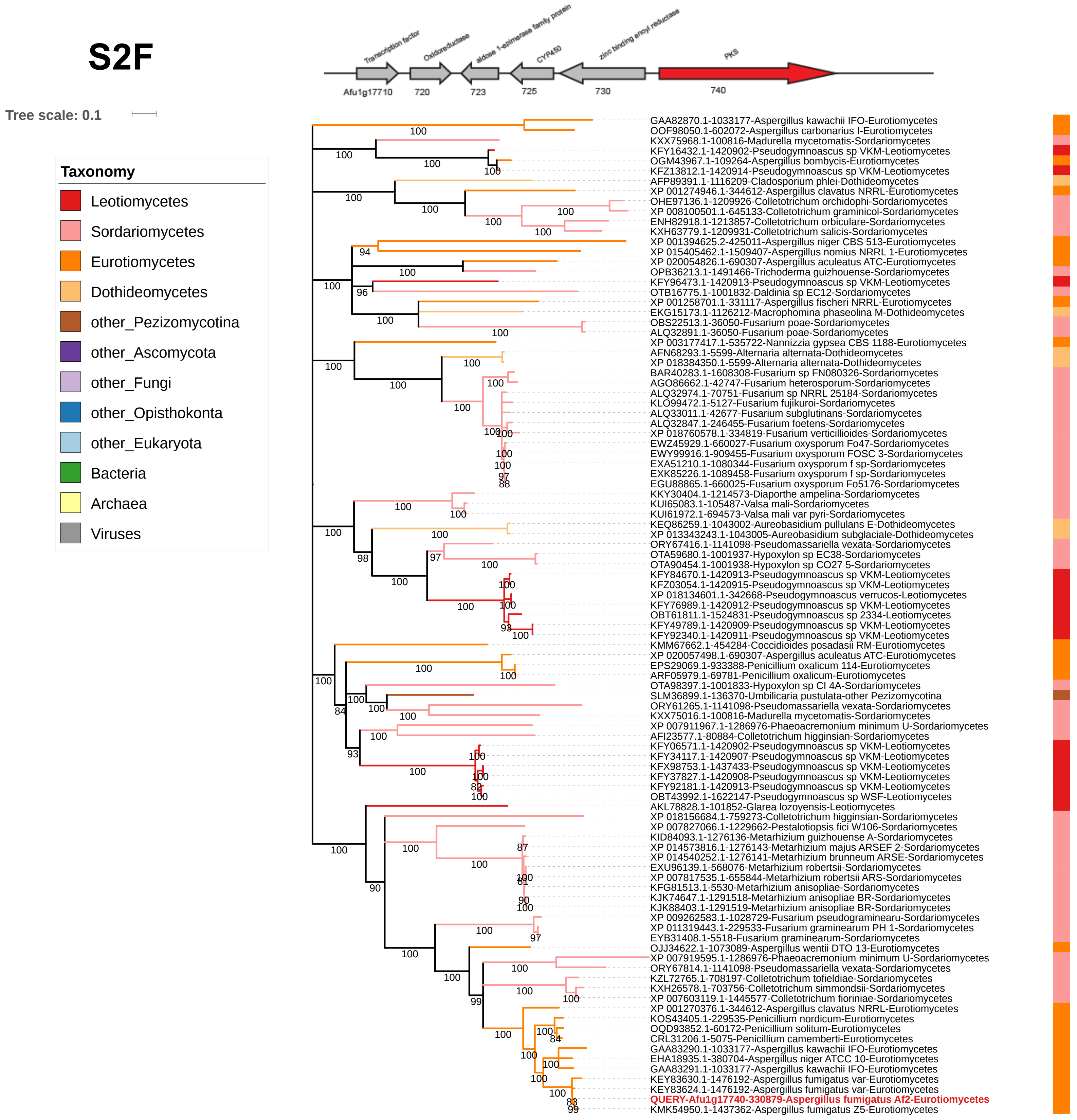

Transcription factor
Oxidoreductase
aldose 1-epimerase family protein
CYP450
zinc binding enoyl reductase
PKS
Afu1g17710
720
723
725
730
740
Tree scale: 0.1
Taxonomy
Leotiomycetes
Sordariomycetes
Eurotiomycetes
Dothideomycetes
other_Pezizomycotina
other_Ascomycota
other_Fungi
other_Opisthokonta
other_Eukaryota
Bacteria
Archaea
Viruses
GAA82870.1-1033177-Aspergillus kawachii IFO-Eurotiomycetes
OOF98050.1-602072-Aspergillus carbonarius I-Eurotiomycetes
KXX75968.1-100816-Madurella mycetomatis-Sordariomycetes
KFY16432.1-1420902-Pseudogymnoascus sp VKM-Leotiomycetes
OGM43967.1-109264-Aspergillus bombycis-Eurotiomycetes
KFZ13812.1-1420914-Pseudogymnoascus sp VKM-Leotiomycetes
AFP89391.1-1116209-Cladosporium phlei-Dothideomycetes
XP 001274946.1-344612-Aspergillus clavatus NRRL-Eurotiomycetes
OHE97136.1-1209926-Colletotrichum orchidophi-Sordariomycetes
XP 008100501.1-645133-Colletotrichum graminicol-Sordariomycetes
ENH82918.1-1213857-Colletotrichum orbiculare-Sordariomycetes
KXH63779.1-1209931-Colletotrichum salicis-Sordariomycetes
XP 001394625.2-425011-Aspergillus niger CBS 513-Eurotiomycetes
XP 015405462.1-1509407-Aspergillus nomius NRRL 1-Eurotiomycetes
XP 020054826.1-690307-Aspergillus aculeatus ATC-Eurotiomycetes
OPB36213.1-1491466-Trichoderma guizhouense-Sordariomycetes
KFY96473.1-1420913-Pseudogymnoascus sp VKM-Leotiomycetes
OTB16775.1-1001832-Daldinia sp EC12-Sordariomycetes
XP 001258701.1-331117-Aspergillus fischeri NRRL-Eurotiomycetes
EKG15173.1-1126212-Macrophomina phaseolina M-Dothideomycetes
OBS22513.1-36050-Fusarium poae-Sordariomycetes
ALQ32891.1-36050-Fusarium poae-Sordariomycetes
XP 003177417.1-535722-Nannizzia gypsea CBS 1188-Eurotiomycetes
AFN68293.1-5599-Alternaria alternata-Dothideomycetes
XP 018384350.1-5599-Alternaria alternata-Dothideomycetes
BAR40283.1-1608308-Fusarium sp FN080326-Sordariomycetes
AGO86662.1-42747-Fusarium heterosporum-Sordariomycetes
ALQ32974.1-70751-Fusarium sp NRRL 25184-Sordariomycetes
KLO99472.1-5127-Fusarium fujikuroi-Sordariomycetes
ALQ33011.1-42677-Fusarium subglutinans-Sordariomycetes
ALQ32847.1-246455-Fusarium foetens-Sordariomycetes
XP 018760578.1-334819-Fusarium verticillioides-Sordariomycetes
EWZ45929.1-660027-Fusarium oxysporum Fo47-Sordariomycetes
EWY99916.1-909455-Fusarium oxysporum FOSC 3-Sordariomycetes
EXA51210.1-1080344-Fusarium oxysporum f sp-Sordariomycetes
EXK85226.1-1089458-Fusarium oxysporum f sp-Sordariomycetes
EGU88865.1-660025-Fusarium oxysporum Fo5176-Sordariomycetes
KKY30404.1-1214573-Diaporthe ampelina-Sordariomycetes
KUI65083.1-105487-Valsa mali-Sordariomycetes
KUI61972.1-694573-Valsa mali var pyri-Sordariomycetes
KEQ86259.1-1043002-Aureobasidium pullulans E-Dothideomycetes
XP 013343243.1-1043005-Aureobasidium subglaciale-Dothideomycetes
ORY67416.1-1141098-Pseudomassariella vexata-Sordariomycetes
OTA59680.1-1001937-Hypoxylon sp EC38-Sordariomycetes
OTA90454.1-1001938-Hypoxylon sp CO27 5-Sordariomycetes
KFY84670.1-1420913-Pseudogymnoascus sp VKM-Leotiomycetes
KFZ03054.1-1420915-Pseudogymnoascus sp VKM-Leotiomycetes
XP 018134601.1-342668-Pseudogymnoascus verrucos-Leotiomycetes
KFY76989.1-1420912-Pseudogymnoascus sp VKM-Leotiomycetes
OBT61811.1-1524831-Pseudogymnoascus sp 2334-Leotiomycetes
KFY49789.1-1420909-Pseudogymnoascus sp VKM-Leotiomycetes
KFY92340.1-1420911-Pseudogymnoascus sp VKM-Leotiomycetes
KMM67662.1-454284-Coccidioides posadasii RM-Eurotiomycetes
XP 020057498.1-690307-Aspergillus aculeatus ATC-Eurotiomycetes
EPS29069.1-933388-Penicillium oxalicum 114-Eurotiomycetes
ARF05979.1-69781-Penicillium oxalicum-Eurotiomycetes
OTA98397.1-1001833-Hypoxylon sp CI 4A-Sordariomycetes
SLM36899.1-136370-Umbilicaria pustulata-other Pezizomycotina
ORY61265.1-1141098-Pseudomassariella vexata-Sordariomycetes
KXX75016.1-100816-Madurella mycetomatis-Sordariomycetes
XP 007911967.1-1286976-Phaeoacremonium minimum U-Sordariomycetes
AFI23577.1-80884-Colletotrichum higginsian-Sordariomycetes
KFY06571.1-1420902-Pseudogymnoascus sp VKM-Leotiomycetes
KFY34117.1-1420907-Pseudogymnoascus sp VKM-Leotiomycetes
KFX98753.1-1437433-Pseudogymnoascus sp VKM-Leotiomycetes
KFY37827.1-1420908-Pseudogymnoascus sp VKM-Leotiomycetes
KFY92181.1-1420913-Pseudogymnoascus sp VKM-Leotiomycetes
OBT43992.1-1622147-Pseudogymnoascus sp WSF-Leotiomycetes
AKL78828.1-101852-Glarea lozoyensis-Leotiomycetes
XP 018156684.1-759273-Colletotrichum higginsian-Sordariomycetes
XP 007827066.1-1229662-Pestalotiopsis fici W106-Sordariomycetes
KID84093.1-1276136-Metarhizium guizhouense A-Sordariomycetes
XP 014573816.1-1276143-Metarhizium majus ARSEF 2-Sordariomycetes
XP 014540252.1-1276141-Metarhizium brunneum ARSE-Sordariomycetes
EXU96139.1-568076-Metarhizium robertsii-Sordariomycetes
XP 007817535.1-655844-Metarhizium robertsii ARS-Sordariomycetes
KFG81513.1-5530-Metarhizium anisopliae-Sordariomycetes
KJK74647.1-1291518-Metarhizium anisopliae BR-Sordariomycetes
KJK88403.1-1291519-Metarhizium anisopliae BR-Sordariomycetes
XP 009262583.1-1028729-Fusarium pseudograminearu-Sordariomycetes
XP 011319443.1-229533-Fusarium graminearum PH 1-Sordariomycetes
EYB31408.1-5518-Fusarium graminearum-Sordariomycetes
OJJ34622.1-1073089-Aspergillus wentii DTO 13-Eurotiomycetes
XP 007919595.1-1286976-Phaeoacremonium minimum U-Sordariomycetes
ORY67814.1-1141098-Pseudomassariella vexata-Sordariomycetes
KZL72765.1-708197-Colletotrichum tofieldiae-Sordariomycetes
KXH26578.1-703756-Colletotrichum simmondsii-Sordariomycetes
XP 007603119.1-1445577-Colletotrichum fioriniae-Sordariomycetes
XP 001270376.1-344612-Aspergillus clavatus NRRL-Eurotiomycetes
KOS43405.1-229535-Penicillium nordicum-Eurotiomycetes
OQD93852.1-60172-Penicillium solitum-Eurotiomycetes
CRL31206.1-5075-Penicillium camemberti-Eurotiomycetes
GAA83290.1-1033177-Aspergillus kawachii IFO-Eurotiomycetes
EHA18935.1-380704-Aspergillus niger ATCC 10-Eurotiomycetes
GAA83291.1-1033177-Aspergillus kawachii IFO-Eurotiomycetes
KEY83630.1-1476192-Aspergillus fumigatus var-Eurotiomycetes
KEY83624.1-1476192-Aspergillus fumigatus var-Eurotiomycetes
QUERY-Afu1g17740-330879-Aspergillus fumigatus Af2-Eurotiomycetes
KMK54950.1-1437362-Aspergillus fumigatus Z5-Eurotiomycetes
