# Supplementary material for: Drivers of genetic diversity in secondary metabolic gene clusters within a fungal species
Source: PLoS Biol. 2017 Nov 17;15(11):e2003583. doi: 10.1371/journal.pbio.2003583 (PMC5711037; doi:10.1371/journal.pbio.2003583)
Supplement: S5 Fig — The phylogenies of several genes in this cluster are consistent with horizontal transfer between Aspergillus fumigatus and Metarhizium fungi. SM, secondary metabolite. (PDF) [file pbio.2003583.s005.pdf]

S5A

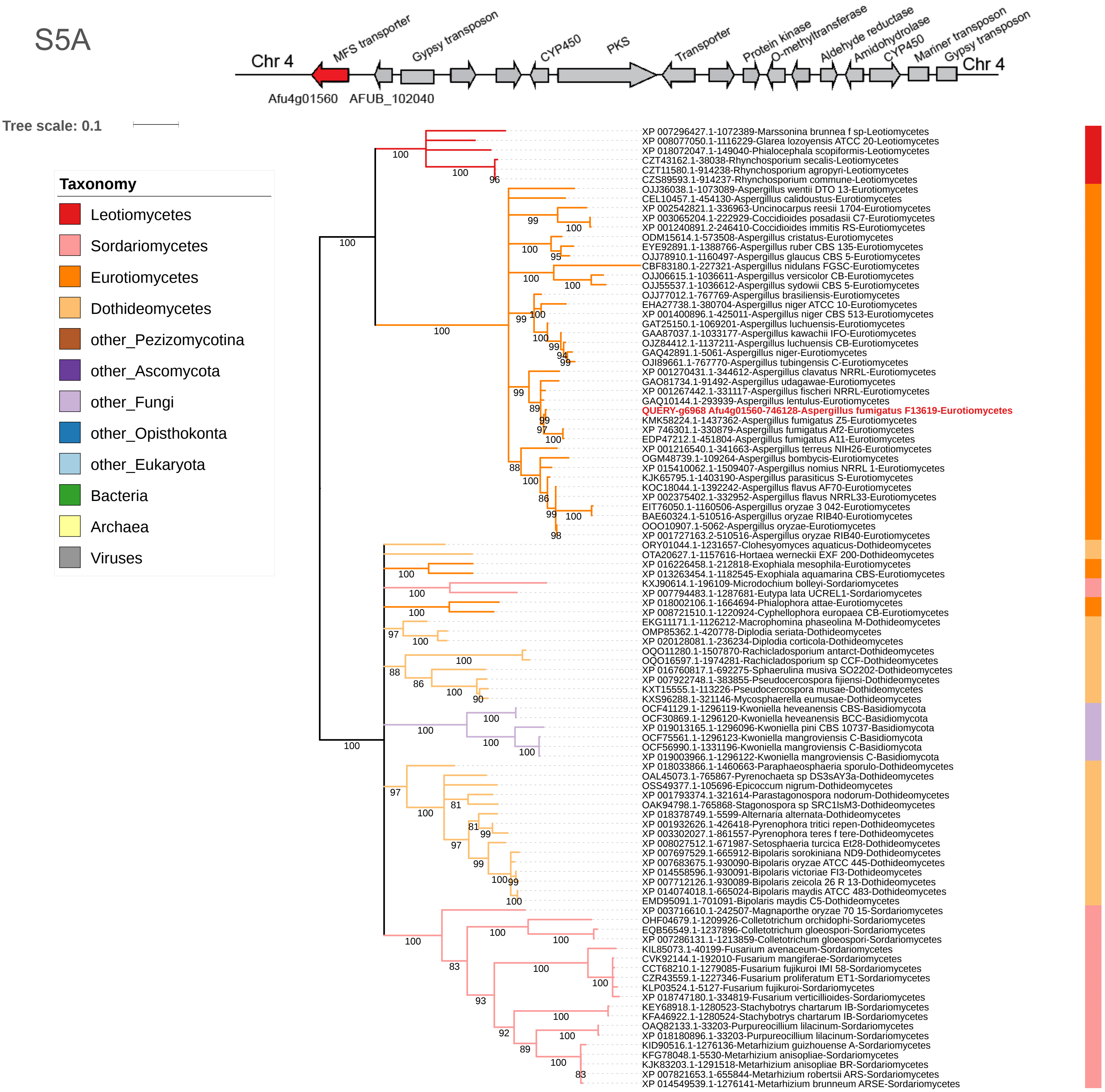

Chr 4
MFS transporter
Gypsy transposon
CYP450
PKS
Transporter
Protein kinase
O-methyltransferase
Aldehyde reductase
Amidohydrolase
CYP450
Mariner transposon
Gypsy transposon
Chr 4
Afu4g01560
AFUB_102040
Tree scale: 0.1
Taxonomy
Leotiomycetes
Sordariomycetes
Eurotiomycetes
Dothideomycetes
other_Pezizomycotina
other_Ascomycota
other_Fungi
other_Opisthokonta
other_Eukaryota
Bacteria
Archaea
Viruses
XP 007296427.1-1072389-Marssonina brunnea f sp-Leotiomycetes
XP 008077050.1-1116229-Glarea lozoyensis ATCC 20-Leotiomycetes
XP 018072047.1-149040-Phialocephala scopiformis-Leotiomycetes
CZT43162.1-38038-Rhynchosporium secalis-Leotiomycetes
CZT11580.1-914238-Rhynchosporium agropyri-Leotiomycetes
CZS89593.1-914237-Rhynchosporium commune-Leotiomycetes
OJJ36038.1-1073089-Aspergillus wentii DTO 13-Eurotiomycetes
CEL10457.1-454130-Aspergillus calidoustus-Eurotiomycetes
XP 002542821.1-336963-Uncinocarpus reesii 1704-Eurotiomycetes
XP 003065204.1-222929-Coccidioides posadasii C7-Eurotiomycetes
XP 001240891.2-246410-Coccidioides immitis RS-Eurotiomycetes
ODM15614.1-573508-Aspergillus cristatus-Eurotiomycetes
EYE92891.1-1388766-Aspergillus ruber CBS 135-Eurotiomycetes
OJJ78910.1-1160497-Aspergillus glaucus CBS 5-Eurotiomycetes
CBF83180.1-227321-Aspergillus nidulans FGSC-Eurotiomycetes
OJJ06615.1-1036611-Aspergillus versicolor CB-Eurotiomycetes
OJJ55537.1-1036612-Aspergillus sydowii CBS 5-Eurotiomycetes
OJJ77012.1-767769-Aspergillus brasiliensis-Eurotiomycetes
EHA27738.1-380704-Aspergillus niger ATCC 10-Eurotiomycetes
XP 001400896.1-425011-Aspergillus niger CBS 513-Eurotiomycetes
GAT25150.1-1069201-Aspergillus luchuensis-Eurotiomycetes
GAA87037.1-1033177-Aspergillus kawachii IFO-Eurotiomycetes
OJZ84412.1-1137211-Aspergillus luchuensis CB-Eurotiomycetes
GAQ42891.1-5061-Aspergillus niger-Eurotiomycetes
OJI89661.1-767770-Aspergillus tubingensis C-Eurotiomycetes
XP 001270431.1-344612-Aspergillus clavatus NRRL-Eurotiomycetes
GAO81734.1-91492-Aspergillus udagawae-Eurotiomycetes
XP 001267442.1-331117-Aspergillus fischeri NRRL-Eurotiomycetes
GAQ10144.1-293939-Aspergillus lentulus-Eurotiomycetes
QUERY-g6968 Afu4g01560-746128-Aspergillus fumigatus F13619-Eurotiomycetes
KMK58224.1-1437362-Aspergillus fumigatus Z5-Eurotiomycetes
XP 746301.1-330879-Aspergillus fumigatus Af2-Eurotiomycetes
EDP47212.1-451804-Aspergillus fumigatus A11-Eurotiomycetes
XP 001216540.1-341663-Aspergillus terreus NIH26-Eurotiomycetes
OGM48739.1-109264-Aspergillus bombycis-Eurotiomycetes
XP 015410062.1-1509407-Aspergillus nomius NRRL 1-Eurotiomycetes
KJK65795.1-1403190-Aspergillus parasiticus S-Eurotiomycetes
KOC18044.1-1392242-Aspergillus flavus AF70-Eurotiomycetes
XP 002375402.1-332952-Aspergillus flavus NRRL33-Eurotiomycetes
EIT76050.1-1160506-Aspergillus oryzae 3 042-Eurotiomycetes
BAE60324.1-510516-Aspergillus oryzae RIB40-Eurotiomycetes
OOO10907.1-5062-Aspergillus oryzae-Eurotiomycetes
XP 001727163.2-510516-Aspergillus oryzae RIB40-Eurotiomycetes
ORY01044.1-1231657-Clohesyomyces aquaticus-Dothideomycetes
OTA20627.1-1157616-Hortaea werneckii EXF 200-Dothideomycetes
XP 016226458.1-212818-Exophiala mesophila-Eurotiomycetes
XP 013263454.1-1182545-Exophiala aquamarina CBS-Eurotiomycetes
KXJ90614.1-196109-Microdochium bolleyi-Sordariomycetes
XP 007794483.1-1287681-Eutypa lata UCREL1-Sordariomycetes
XP 018002106.1-1664694-Phialophora attae-Eurotiomycetes
XP 008721510.1-1220924-Cyphellophora europaea CB-Eurotiomycetes
EKG11171.1-1126212-Macrophomina phaseolina M-Dothideomycetes
OMP85362.1-420778-Diplodia seriata-Dothideomycetes
XP 020128081.1-236234-Diplodia corticola-Dothideomycetes
OQO11280.1-1507870-Rachicladosporium antarct-Dothideomycetes
OQO16597.1-1974281-Rachicladosporium sp CCF-Dothideomycetes
XP 016760817.1-692275-Sphaerulina musiva SO2202-Dothideomycetes
XP 007922748.1-383855-Pseudocercospora fijiensi-Dothideomycetes
KXT15555.1-113226-Pseudocercospora musae-Dothideomycetes
KXS96288.1-321146-Mycosphaerella eumusae-Dothideomycetes
OCF41129.1-1296119-Kwoniella heveanensis CBS-Basidiomycota
OCF30869.1-1296120-Kwoniella heveanensis BCC-Basidiomycota
XP 019013165.1-1296096-Kwoniella pini CBS 10737-Basidiomycota
OCF75561.1-1296123-Kwoniella mangroviensis C-Basidiomycota
OCF56990.1-1331196-Kwoniella mangroviensis C-Basidiomycota
XP 019003966.1-1296122-Kwoniella mangroviensis C-Basidiomycota
XP 018033866.1-1460663-Paraphaeosphaeria sporulo-Dothideomycetes
OAL45073.1-765867-Pyrenochaeta sp DS3sAY3a-Dothideomycetes
OSS49377.1-105696-Epicoccum nigrum-Dothideomycetes
XP 001793374.1-321614-Parastagonospora nodorum-Dothideomycetes
OAK94798.1-765868-Stagonospora sp SRC1lsM3-Dothideomycetes
XP 018378749.1-5599-Alternaria alternata-Dothideomycetes
XP 001932626.1-426418-Pyrenophora tritici repen-Dothideomycetes
XP 003302027.1-861557-Pyrenophora teres f tere-Dothideomycetes
XP 008027512.1-671987-Setosphaeria turcica Et28-Dothideomycetes
XP 007697529.1-665912-Bipolaris sorokiniana ND9-Dothideomycetes
XP 007683675.1-930090-Bipolaris oryzae ATCC 445-Dothideomycetes
XP 014558596.1-930091-Bipolaris victoriae FI3-Dothideomycetes
XP 007712126.1-930089-Bipolaris zeicola 26 R 13-Dothideomycetes
XP 014074018.1-665024-Bipolaris maydis ATCC 483-Dothideomycetes
EMD95091.1-701091-Bipolaris maydis C5-Dothideomycetes
XP 003716610.1-242507-Magnaporthe oryzae 70 15-Sordariomycetes
OHF04679.1-1209926-Colletotrichum orchidophi-Sordariomycetes
EQB56549.1-1237896-Colletotrichum gloeospori-Sordariomycetes
XP 007286131.1-1213859-Colletotrichum gloeospori-Sordariomycetes
KIL85073.1-40199-Fusarium avenaceum-Sordariomycetes
CVK92144.1-192010-Fusarium mangiferae-Sordariomycetes
CCT68210.1-1279085-Fusarium fujikuroi IMI 58-Sordariomycetes
CZR43559.1-1227346-Fusarium proliferatum ET1-Sordariomycetes
KLP03524.1-5127-Fusarium fujikuroi-Sordariomycetes
XP 018747180.1-334819-Fusarium verticillioides-Sordariomycetes
KEY68918.1-1280523-Stachybotrys chartarum IB-Sordariomycetes
KFA46922.1-1280524-Stachybotrys chartarum IB-Sordariomycetes
OAQ82133.1-33203-Purpureocillium lilacinum-Sordariomycetes
XP 018180896.1-33203-Purpureocillium lilacinum-Sordariomycetes
KID90516.1-1276136-Metarhizium guizhouense A-Sordariomycetes
KFG78048.1-5530-Metarhizium anisopliae-Sordariomycetes
KJK83203.1-1291518-Metarhizium anisopliae BR-Sordariomycetes
XP 007821653.1-655844-Metarhizium robertsii ARS-Sordariomycetes
XP 014549539.1-1276141-Metarhizium brunneum ARSE-Sordariomycetes

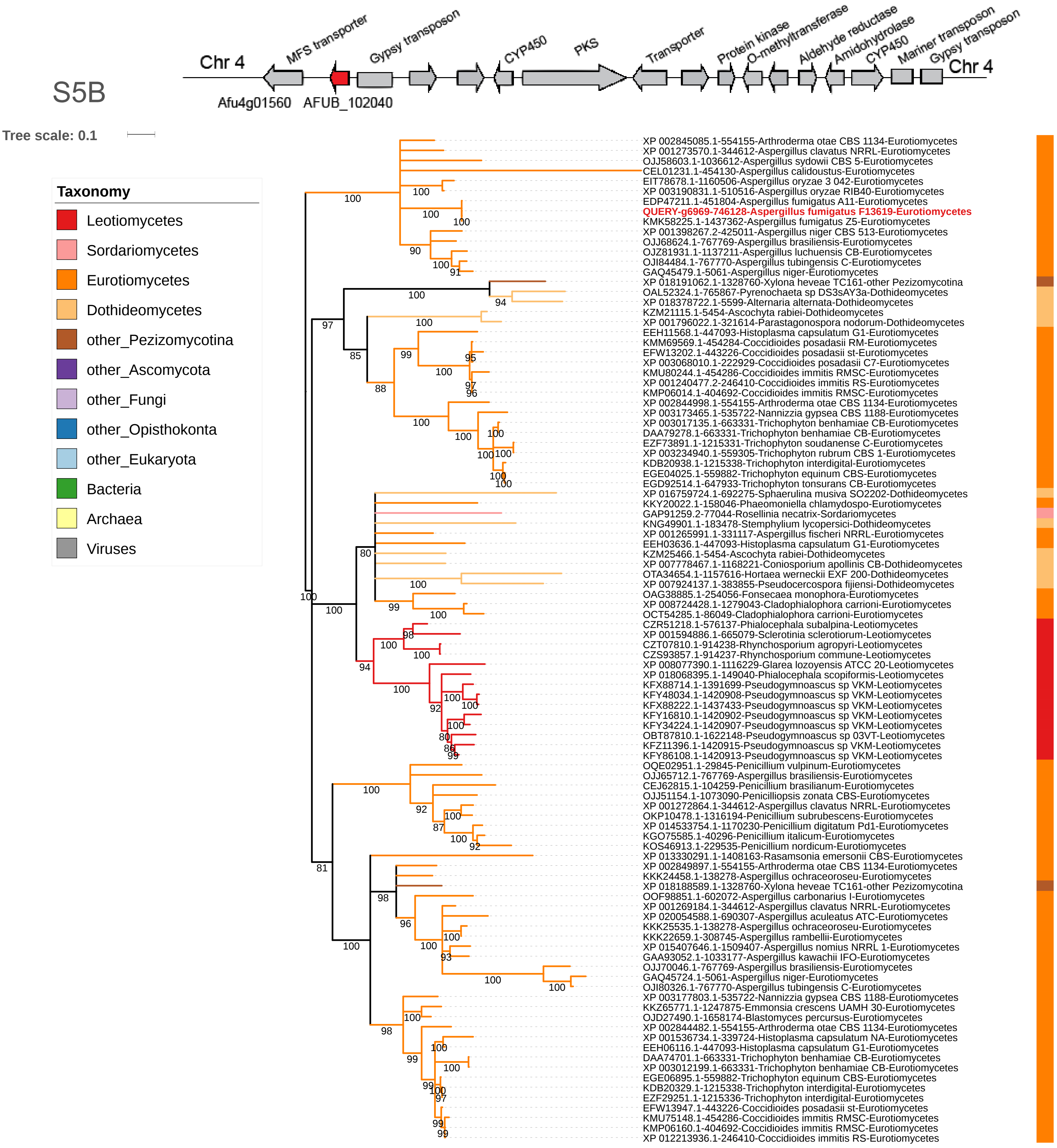
S5B
Chr 4
MFS transporter
Gypsy transposon
CYP450
PKS
Transporter
Protein kinase
O-methyltransferase
Aldehyde reductase
Amidohydrolase
CYP450
Mariner transposon
Gypsy transposon
Chr 4
Afu4g01560
AFUB_102040
Tree scale: 0.1
Taxonomy
Leotiomycetes
Sordariomycetes
Eurotiomycetes
Dothideomycetes
other_Pezizomycotina
other_Ascomycota
other_Fungi
other_Opisthokonta
other_Eukaryota
Bacteria
Archaea
Viruses
XP 002845085.1-554155-Arthroderma otae CBS 1134-Eurotiomycetes
XP 001273570.1-344612-Aspergillus clavatus NRRL-Eurotiomycetes
OJJ58603.1-1036612-Aspergillus sydowii CBS 5-Eurotiomycetes
CEL01231.1-454130-Aspergillus calidoustus-Eurotiomycetes
EIT78678.1-1160506-Aspergillus oryzae 3 042-Eurotiomycetes
XP 003190831.1-510516-Aspergillus oryzae RIB40-Eurotiomycetes
EDP47211.1-451804-Aspergillus fumigatus A11-Eurotiomycetes
QUERY-g6969-746128-Aspergillus fumigatus F13619-Eurotiomycetes
KMK58225.1-1437362-Aspergillus fumigatus Z5-Eurotiomycetes
XP 001398267.2-425011-Aspergillus niger CBS 513-Eurotiomycetes
OJJ68624.1-767769-Aspergillus brasiliensis-Eurotiomycetes
OJZ81931.1-1137211-Aspergillus luchuensis CB-Eurotiomycetes
OJI84484.1-767770-Aspergillus tubingensis C-Eurotiomycetes
GAQ45479.1-5061-Aspergillus niger-Eurotiomycetes
XP 018191062.1-1328760-Xylona heveae TC161-other Pezizomycotina
OAL52324.1-765867-Pyrenochaeta sp DS3sAY3a-Dothideomycetes
XP 018378722.1-5599-Alternaria alternata-Dothideomycetes
KZM21115.1-5454-Ascochyta rabiei-Dothideomycetes
XP 001796022.1-321614-Parastagonospora nodorum-Dothideomycetes
EEH11568.1-447093-Histoplasma capsulatum G1-Eurotiomycetes
KMM69569.1-454284-Coccidioides posadasii RM-Eurotiomycetes
EFW13202.1-443226-Coccidioides posadasii st-Eurotiomycetes
XP 003068010.1-222929-Coccidioides posadasii C7-Eurotiomycetes
KMU80244.1-454286-Coccidioides immitis RMSC-Eurotiomycetes
XP 001240477.2-246410-Coccidioides immitis RS-Eurotiomycetes
KMP06014.1-404692-Coccidioides immitis RMSC-Eurotiomycetes
XP 002844998.1-554155-Arthroderma otae CBS 1134-Eurotiomycetes
XP 003173465.1-535722-Nannizzia gypsea CBS 1188-Eurotiomycetes
XP 003017135.1-663331-Trichophyton benhamiae CB-Eurotiomycetes
DAA79278.1-663331-Trichophyton benhamiae CB-Eurotiomycetes
EZF73891.1-1215331-Trichophyton soudanense C-Eurotiomycetes
XP 003234940.1-559305-Trichophyton rubrum CBS 1-Eurotiomycetes
KDB20938.1-1215338-Trichophyton interdigital-Eurotiomycetes
EGE04025.1-559882-Trichophyton equinum CBS-Eurotiomycetes
EGD92514.1-647933-Trichophyton tonsurans CB-Eurotiomycetes
XP 016759724.1-692275-Sphaerulina musiva SO2202-Dothideomycetes
KKY20022.1-158046-Phaeomoniella chlamydospo-Eurotiomycetes
GAP91259.2-77044-Rosellinia necatrix-Sordariomycetes
KNG49901.1-183478-Stemphylium lycopersici-Dothideomycetes
XP 001265991.1-331117-Aspergillus fischeri NRRL-Eurotiomycetes
EEH03636.1-447093-Histoplasma capsulatum G1-Eurotiomycetes
KZM25466.1-5454-Ascochyta rabiei-Dothideomycetes
XP 007778467.1-1168221-Coniosporium apollinis CB-Dothideomycetes
OTA34654.1-1157616-Hortaea werneckii EXF 200-Dothideomycetes
XP 007924137.1-383855-Pseudocercospora fijiensi-Dothideomycetes
OAG38885.1-254056-Fonsecaea monophora-Eurotiomycetes
XP 008724428.1-1279043-Cladophialophora carrioni-Eurotiomycetes
OCT54285.1-86049-Cladophialophora carrioni-Eurotiomycetes
CZR51218.1-576137-Phialocephala subalpina-Leotiomycetes
XP 001594886.1-665079-Sclerotinia sclerotiorum-Leotiomycetes
CZT07810.1-914238-Rhynchosporium agropyri-Leotiomycetes
CZS93857.1-914237-Rhynchosporium commune-Leotiomycetes
XP 008077390.1-1116229-Glarea lozoyensis ATCC 20-Leotiomycetes
XP 018068395.1-149040-Phialocephala scopiformis-Leotiomycetes
KFX88714.1-1391699-Pseudogymnoascus sp VKM-Leotiomycetes
KFY48034.1-1420908-Pseudogymnoascus sp VKM-Leotiomycetes
KFX88222.1-1437433-Pseudogymnoascus sp VKM-Leotiomycetes
KFY16810.1-1420902-Pseudogymnoascus sp VKM-Leotiomycetes
KFY34224.1-1420907-Pseudogymnoascus sp VKM-Leotiomycetes
OBT87810.1-1622148-Pseudogymnoascus sp 03VT-Leotiomycetes
KFZ11396.1-1420915-Pseudogymnoascus sp VKM-Leotiomycetes
KFY86108.1-1420913-Pseudogymnoascus sp VKM-Leotiomycetes
OQE02951.1-29845-Penicillium vulpinum-Eurotiomycetes
OJJ65712.1-767769-Aspergillus brasiliensis-Eurotiomycetes
CEJ62815.1-104259-Penicillium brasilianum-Eurotiomycetes
OJJ51154.1-1073090-Penicilliopsis zonata CBS-Eurotiomycetes
XP 001272864.1-344612-Aspergillus clavatus NRRL-Eurotiomycetes
OKP10478.1-1316194-Penicillium subrubescens-Eurotiomycetes
XP 014533754.1-1170230-Penicillium digitatum Pd1-Eurotiomycetes
KGO75585.1-40296-Penicillium italicum-Eurotiomycetes
KOS46913.1-229535-Penicillium nordicum-Eurotiomycetes
XP 013330291.1-1408163-Rasamsonia emersonii CBS-Eurotiomycetes
XP 002849897.1-554155-Arthroderma otae CBS 1134-Eurotiomycetes
KKK24458.1-138278-Aspergillus ochraceoroseu-Eurotiomycetes
XP 018188589.1-1328760-Xylona heveae TC161-other Pezizomycotina
OOF98851.1-602072-Aspergillus carbonarius I-Eurotiomycetes
XP 001269184.1-344612-Aspergillus clavatus NRRL-Eurotiomycetes
XP 020054588.1-690307-Aspergillus aculeatus ATC-Eurotiomycetes
KKK25535.1-138278-Aspergillus ochraceoroseu-Eurotiomycetes
KKK22659.1-308745-Aspergillus rambellii-Eurotiomycetes
XP 015407646.1-1509407-Aspergillus nomius NRRL 1-Eurotiomycetes
GAA93052.1-1033177-Aspergillus kawachii IFO-Eurotiomycetes
OJJ70046.1-767769-Aspergillus brasiliensis-Eurotiomycetes
GAQ45724.1-5061-Aspergillus niger-Eurotiomycetes
OJI80326.1-767770-Aspergillus tubingensis C-Eurotiomycetes
XP 003177803.1-535722-Nannizzia gypsea CBS 1188-Eurotiomycetes
KKZ65771.1-1247875-Emmonsia crescens UAMH 30-Eurotiomycetes
OJD27490.1-1658174-Blastomyces percursus-Eurotiomycetes
XP 002844482.1-554155-Arthroderma otae CBS 1134-Eurotiomycetes
XP 001536734.1-339724-Histoplasma capsulatum NA-Eurotiomycetes
EEH06116.1-447093-Histoplasma capsulatum G1-Eurotiomycetes
DAA74701.1-663331-Trichophyton benhamiae CB-Eurotiomycetes
XP 003012199.1-663331-Trichophyton benhamiae CB-Eurotiomycetes
EGE06895.1-559882-Trichophyton equinum CBS-Eurotiomycetes
KDB20329.1-1215338-Trichophyton interdigital-Eurotiomycetes
EZF29251.1-1215336-Trichophyton interdigital-Eurotiomycetes
EFW13947.1-443226-Coccidioides posadasii st-Eurotiomycetes
KMU75148.1-454286-Coccidioides immitis RMSC-Eurotiomycetes
KMP06160.1-404692-Coccidioides immitis RMSC-Eurotiomycetes
XP 012213936.1-246410-Coccidioides immitis RS-Eurotiomycetes

S5C

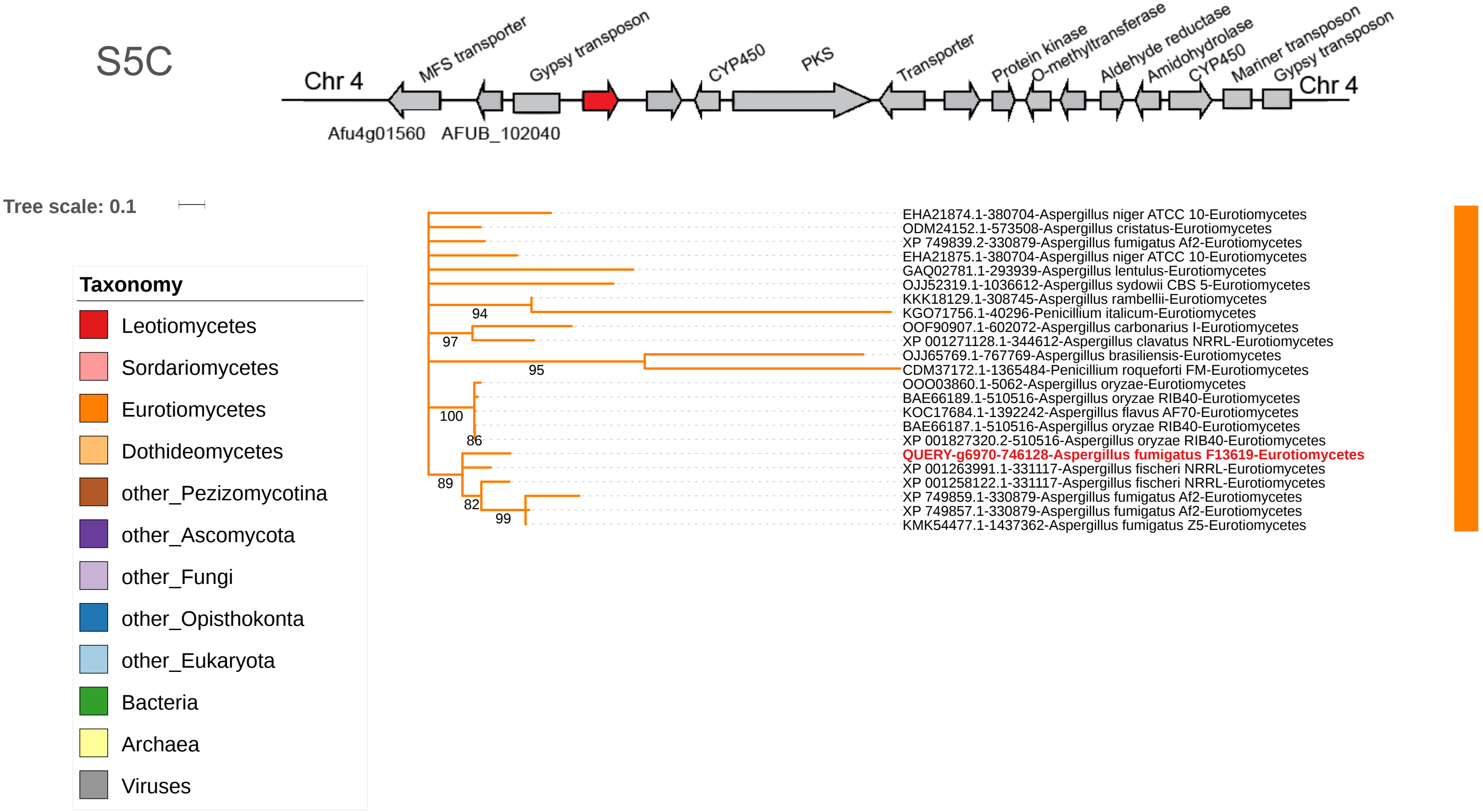

Chr 4
MFS transporter
Gypsy transposon
CYP450
PKS
Transporter
Protein kinase
O-methyltransferase
Aldehyde reductase
Amidohydrolase
CYP450
Mariner transposon
Gypsy transposon
Chr 4
Afu4g01560
AFUB_102040
Tree scale: 0.1
Taxonomy
Leotiomycetes
Sordariomycetes
Eurotiomycetes
Dothideomycetes
other_Pezizomycotina
other_Ascomycota
other_Fungi
other_Opisthokonta
other_Eukaryota
Bacteria
Archaea
Viruses
EHA21874.1-380704-Aspergillus niger ATCC 10-Eurotiomycetes
ODM24152.1-573508-Aspergillus cristatus-Eurotiomycetes
XP 749839.2-330879-Aspergillus fumigatus Af2-Eurotiomycetes
EHA21875.1-380704-Aspergillus niger ATCC 10-Eurotiomycetes
GAQ02781.1-293939-Aspergillus lentulus-Eurotiomycetes
OJJ52319.1-1036612-Aspergillus sydowii CBS 5-Eurotiomycetes
KKK18129.1-308745-Aspergillus rambellii-Eurotiomycetes
KGO71756.1-40296-Penicillium italicum-Eurotiomycetes
OOF90907.1-602072-Aspergillus carbonarius I-Eurotiomycetes
XP 001271128.1-344612-Aspergillus clavatus NRRL-Eurotiomycetes
OJJ65769.1-767769-Aspergillus brasiliensis-Eurotiomycetes
CDM37172.1-1365484-Penicillium roqueforti FM-Eurotiomycetes
OOO03860.1-5062-Aspergillus oryzae-Eurotiomycetes
BAE66189.1-510516-Aspergillus oryzae RIB40-Eurotiomycetes
KOC17684.1-1392242-Aspergillus flavus AF70-Eurotiomycetes
BAE66187.1-510516-Aspergillus oryzae RIB40-Eurotiomycetes
XP 001827320.2-510516-Aspergillus oryzae RIB40-Eurotiomycetes
QUERY-g6970-746128-Aspergillus fumigatus F13619-Eurotiomycetes
XP 001263991.1-331117-Aspergillus fischeri NRRL-Eurotiomycetes
XP 001258122.1-331117-Aspergillus fischeri NRRL-Eurotiomycetes
XP 749859.1-330879-Aspergillus fumigatus Af2-Eurotiomycetes
XP 749857.1-330879-Aspergillus fumigatus Af2-Eurotiomycetes
KMK54477.1-1437362-Aspergillus fumigatus Z5-Eurotiomycetes
94
97
95
100
86
89
82
99

S5D

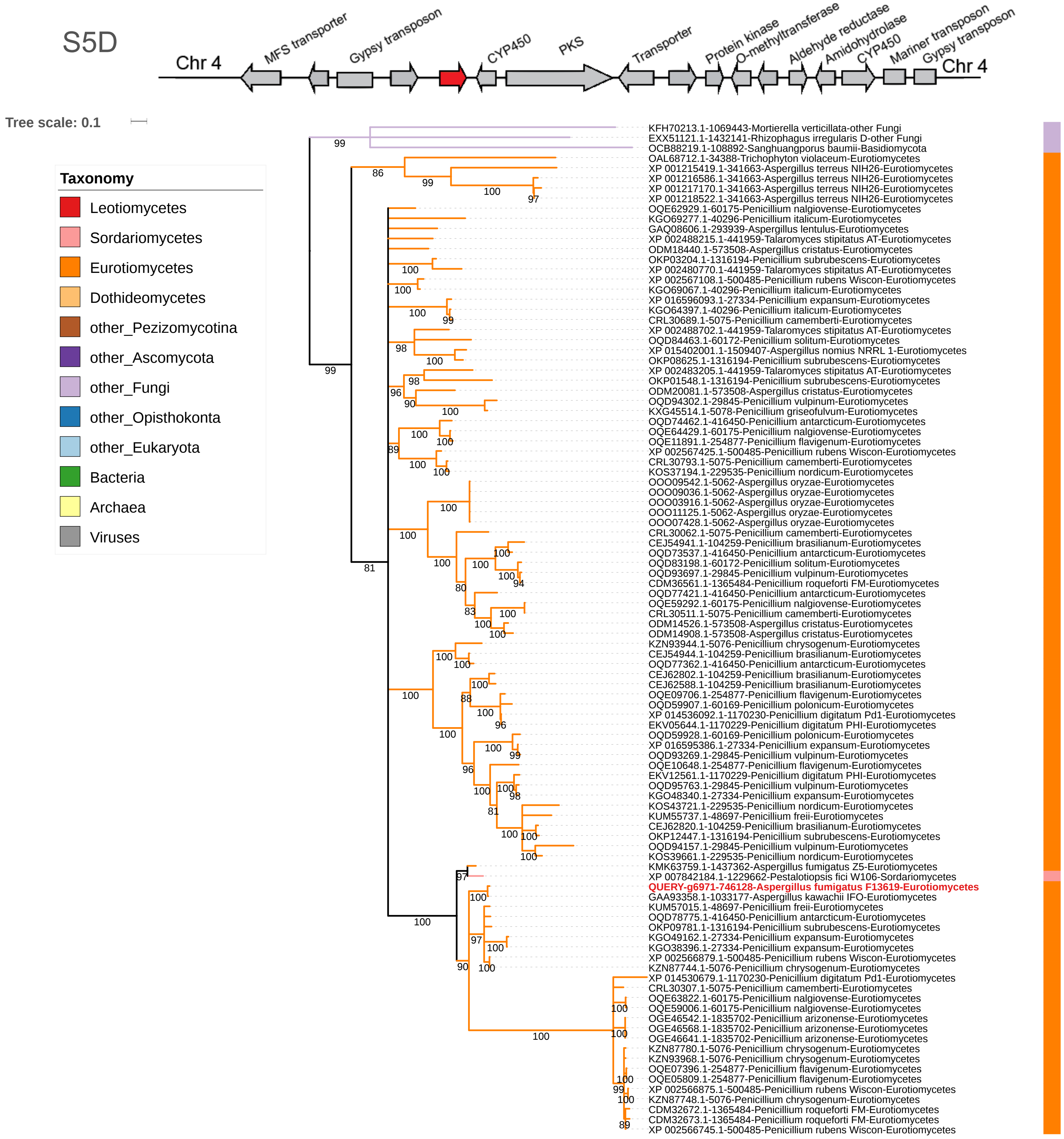

Chr 4
MFS transporter
Gypsy transposon
CYP450
PKS
Transporter
Protein kinase
O-methyltransferase
Aldehyde reductase
Amidohydrolase
CYP450
Mariner transposon
Gypsy transposon
Chr 4
Tree scale: 0.1
Taxonomy
Leotiomycetes
Sordariomycetes
Eurotiomycetes
Dothideomycetes
other_Pezizomycotina
other_Ascomycota
other_Fungi
other_Opisthokonta
other_Eukaryota
Bacteria
Archaea
Viruses
KFH70213.1-1069443-Mortierella verticillata-other Fungi
EXX51121.1-1432141-Rhizophagus irregularis D-other Fungi
OCB88219.1-108892-Sanghuangporus baumii-Basidiomycota
OAL68712.1-34388-Trichophyton violaceum-Eurotiomycetes
XP 001215419.1-341663-Aspergillus terreus NIH26-Eurotiomycetes
XP 001216586.1-341663-Aspergillus terreus NIH26-Eurotiomycetes
XP 001217170.1-341663-Aspergillus terreus NIH26-Eurotiomycetes
XP 001218522.1-341663-Aspergillus terreus NIH26-Eurotiomycetes
OQE62929.1-60175-Penicillium nalgiovense-Eurotiomycetes
KGO69277.1-40296-Penicillium italicum-Eurotiomycetes
GAQ08606.1-293939-Aspergillus lentulus-Eurotiomycetes
XP 002488215.1-441959-Talaromyces stipitatus AT-Eurotiomycetes
ODM18440.1-573508-Aspergillus cristatus-Eurotiomycetes
OKP03204.1-1316194-Penicillium subrubescens-Eurotiomycetes
XP 002480770.1-441959-Talaromyces stipitatus AT-Eurotiomycetes
XP 002567108.1-500485-Penicillium rubens Wiscon-Eurotiomycetes
KGO69067.1-40296-Penicillium italicum-Eurotiomycetes
XP 016596093.1-27334-Penicillium expansum-Eurotiomycetes
KGO64397.1-40296-Penicillium italicum-Eurotiomycetes
CRL30689.1-5075-Penicillium camemberti-Eurotiomycetes
XP 002488702.1-441959-Talaromyces stipitatus AT-Eurotiomycetes
OQD84463.1-60172-Penicillium solitum-Eurotiomycetes
XP 015402001.1-1509407-Aspergillus nomius NRRL 1-Eurotiomycetes
OKP08625.1-1316194-Penicillium subrubescens-Eurotiomycetes
XP 002483205.1-441959-Talaromyces stipitatus AT-Eurotiomycetes
OKP01548.1-1316194-Penicillium subrubescens-Eurotiomycetes
ODM20081.1-573508-Aspergillus cristatus-Eurotiomycetes
OQD94302.1-29845-Penicillium vulpinum-Eurotiomycetes
KXG45514.1-5078-Penicillium griseofulvum-Eurotiomycetes
OQD74462.1-416450-Penicillium antarcticum-Eurotiomycetes
OQE64429.1-60175-Penicillium nalgiovense-Eurotiomycetes
OQE11891.1-254877-Penicillium flavigenum-Eurotiomycetes
XP 002567425.1-500485-Penicillium rubens Wiscon-Eurotiomycetes
CRL30793.1-5075-Penicillium camemberti-Eurotiomycetes
KOS37194.1-229535-Penicillium nordicum-Eurotiomycetes
OOO09542.1-5062-Aspergillus oryzae-Eurotiomycetes
OOO09036.1-5062-Aspergillus oryzae-Eurotiomycetes
OOO03916.1-5062-Aspergillus oryzae-Eurotiomycetes
OOO11125.1-5062-Aspergillus oryzae-Eurotiomycetes
OOO07428.1-5062-Aspergillus oryzae-Eurotiomycetes
CRL30062.1-5075-Penicillium camemberti-Eurotiomycetes
CEJ54941.1-104259-Penicillium brasilianum-Eurotiomycetes
OQD73537.1-416450-Penicillium antarcticum-Eurotiomycetes
OQD83198.1-60172-Penicillium solitum-Eurotiomycetes
OQD93697.1-29845-Penicillium vulpinum-Eurotiomycetes
CDM36561.1-1365484-Penicillium roqueforti FM-Eurotiomycetes
OQD77421.1-416450-Penicillium antarcticum-Eurotiomycetes
OQE59292.1-60175-Penicillium nalgiovense-Eurotiomycetes
CRL30511.1-5075-Penicillium camemberti-Eurotiomycetes
ODM14526.1-573508-Aspergillus cristatus-Eurotiomycetes
ODM14908.1-573508-Aspergillus cristatus-Eurotiomycetes
KZN93944.1-5076-Penicillium chrysogenum-Eurotiomycetes
CEJ54944.1-104259-Penicillium brasilianum-Eurotiomycetes
OQD77362.1-416450-Penicillium antarcticum-Eurotiomycetes
CEJ62802.1-104259-Penicillium brasilianum-Eurotiomycetes
CEJ62588.1-104259-Penicillium brasilianum-Eurotiomycetes
OQE09706.1-254877-Penicillium flavigenum-Eurotiomycetes
OQD59907.1-60169-Penicillium polonicum-Eurotiomycetes
XP 014536092.1-1170230-Penicillium digitatum Pd1-Eurotiomycetes
EKV05644.1-1170229-Penicillium digitatum PHI-Eurotiomycetes
OQD59928.1-60169-Penicillium polonicum-Eurotiomycetes
XP 016595386.1-27334-Penicillium expansum-Eurotiomycetes
OQD93269.1-29845-Penicillium vulpinum-Eurotiomycetes
OQE10648.1-254877-Penicillium flavigenum-Eurotiomycetes
EKV12561.1-1170229-Penicillium digitatum PHI-Eurotiomycetes
OQD95763.1-29845-Penicillium vulpinum-Eurotiomycetes
KGO48340.1-27334-Penicillium expansum-Eurotiomycetes
KOS43721.1-229535-Penicillium nordicum-Eurotiomycetes
KUM55737.1-48697-Penicillium freii-Eurotiomycetes
CEJ62820.1-104259-Penicillium brasilianum-Eurotiomycetes
OKP12447.1-1316194-Penicillium subrubescens-Eurotiomycetes
OQD94157.1-29845-Penicillium vulpinum-Eurotiomycetes
KOS39661.1-229535-Penicillium nordicum-Eurotiomycetes
KMK63759.1-1437362-Aspergillus fumigatus Z5-Eurotiomycetes
XP 007842184.1-1229662-Pestalotiopsis fici W106-Sordariomycetes
QUERY-g6971-746128-Aspergillus fumigatus F13619-Eurotiomycetes
GAA93358.1-1033177-Aspergillus kawachii IFO-Eurotiomycetes
KUM57015.1-48697-Penicillium freii-Eurotiomycetes
OQD78775.1-416450-Penicillium antarcticum-Eurotiomycetes
OKP09781.1-1316194-Penicillium subrubescens-Eurotiomycetes
KGO49162.1-27334-Penicillium expansum-Eurotiomycetes
KGO38396.1-27334-Penicillium expansum-Eurotiomycetes
XP 002566879.1-500485-Penicillium rubens Wiscon-Eurotiomycetes
KZN87744.1-5076-Penicillium chrysogenum-Eurotiomycetes
XP 014530679.1-1170230-Penicillium digitatum Pd1-Eurotiomycetes
CRL30307.1-5075-Penicillium camemberti-Eurotiomycetes
OQE63822.1-60175-Penicillium nalgiovense-Eurotiomycetes
OQE59006.1-60175-Penicillium nalgiovense-Eurotiomycetes
OGE46542.1-1835702-Penicillium arizonense-Eurotiomycetes
OGE46568.1-1835702-Penicillium arizonense-Eurotiomycetes
OGE46641.1-1835702-Penicillium arizonense-Eurotiomycetes
KZN87780.1-5076-Penicillium chrysogenum-Eurotiomycetes
KZN93968.1-5076-Penicillium chrysogenum-Eurotiomycetes
OQE07396.1-254877-Penicillium flavigenum-Eurotiomycetes
OQE05809.1-254877-Penicillium flavigenum-Eurotiomycetes
XP 002566875.1-500485-Penicillium rubens Wiscon-Eurotiomycetes
KZN87748.1-5076-Penicillium chrysogenum-Eurotiomycetes
CDM32672.1-1365484-Penicillium roqueforti FM-Eurotiomycetes
CDM32673.1-1365484-Penicillium roqueforti FM-Eurotiomycetes
XP 002566745.1-500485-Penicillium rubens Wiscon-Eurotiomycetes

S5E

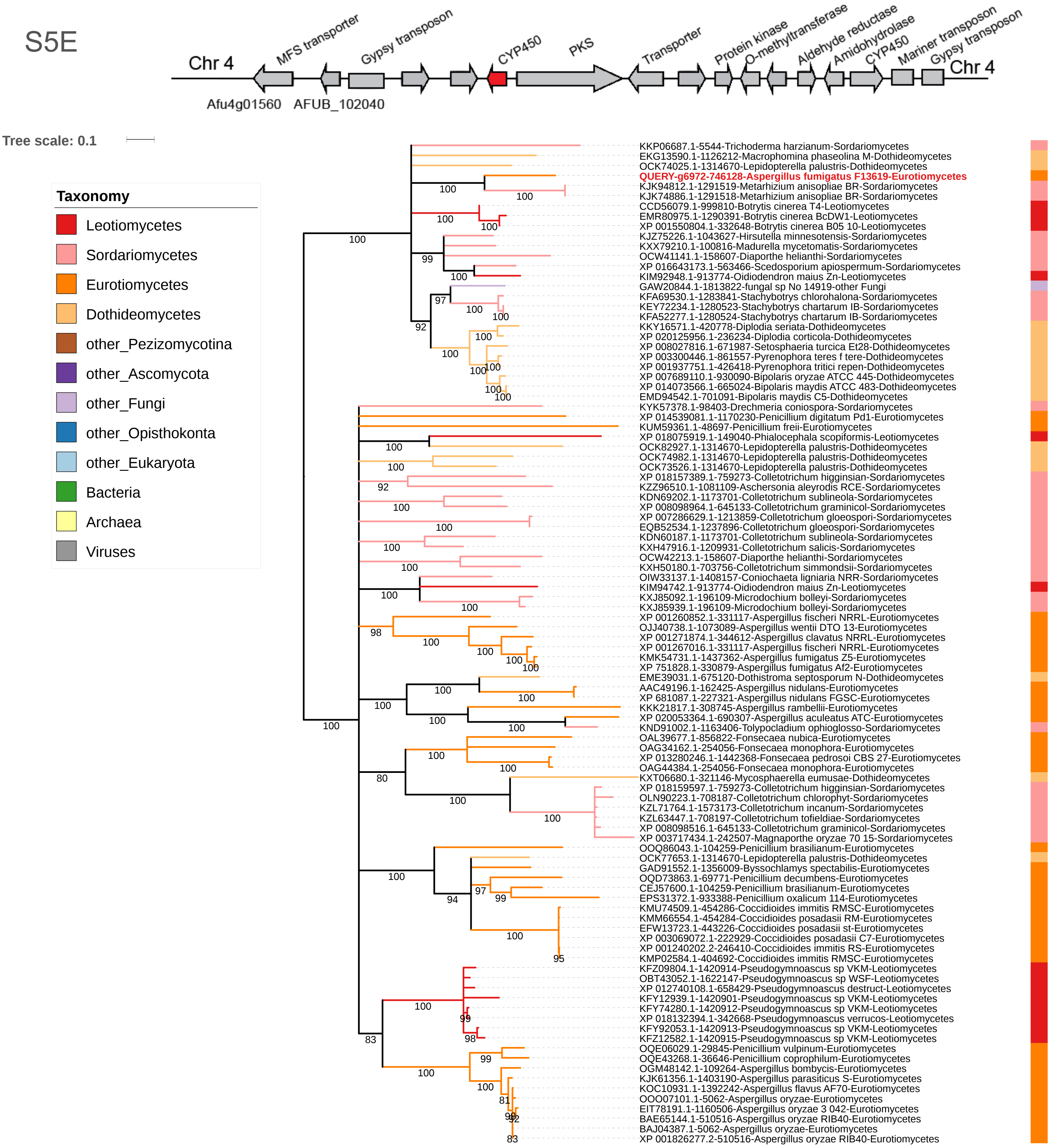

Chr 4
MFS transporter
Gypsy transposon
CYP450
PKS
Transporter
Protein kinase
O-methyltransferase
Aldehyde reductase
Amidohydrolase
CYP450
Mariner transposon
Gypsy transposon
Chr 4
Afu4g01560
AFUB_102040
Tree scale: 0.1
Taxonomy
Leotiomycetes
Sordariomycetes
Eurotiomycetes
Dothideomycetes
other_Pezizomycotina
other_Ascomycota
other_Fungi
other_Opisthokonta
other_Eukaryota
Bacteria
Archaea
Viruses
KKP06687.1-5544-Trichoderma harzianum-Sordariomycetes
EKG13590.1-1126212-Macrophomina phaseolina M-Dothideomycetes
OCK74025.1-1314670-Lepidopterella palustris-Dothideomycetes
QUERY-g6972-746128-Aspergillus fumigatus F13619-Eurotiomycetes
KJK94812.1-1291519-Metarhizium anisopliae BR-Sordariomycetes
KJK74886.1-1291518-Metarhizium anisopliae BR-Sordariomycetes
CCD56079.1-999810-Botrytis cinerea T4-Leotiomycetes
EMR80975.1-1290391-Botrytis cinerea BcDW1-Leotiomycetes
XP 001550804.1-332648-Botrytis cinerea B05 10-Leotiomycetes
KJZ75226.1-1043627-Hirsutella minnesotensis-Sordariomycetes
KXX79210.1-100816-Madurella mycetomatis-Sordariomycetes
OCW41141.1-158607-Diaporthe helianthi-Sordariomycetes
XP 016643173.1-563466-Scedosporium apiospermum-Sordariomycetes
KIM92948.1-913774-Oidiodendron maius Zn-Leotiomycetes
GAW20844.1-1813822-fungal sp No 14919-other Fungi
KFA69530.1-1283841-Stachybotrys chlorohalona-Sordariomycetes
KEY72234.1-1280523-Stachybotrys chartarum IB-Sordariomycetes
KFA52277.1-1280524-Stachybotrys chartarum IB-Sordariomycetes
KKY16571.1-420778-Diplodia seriata-Dothideomycetes
XP 020125956.1-236234-Diplodia corticola-Dothideomycetes
XP 008027816.1-671987-Setosphaeria turcica Et28-Dothideomycetes
XP 003300446.1-861557-Pyrenophora teres f tere-Dothideomycetes
XP 001937751.1-426418-Pyrenophora tritici repen-Dothideomycetes
XP 007689110.1-930090-Bipolaris oryzae ATCC 445-Dothideomycetes
XP 014073566.1-665024-Bipolaris maydis ATCC 483-Dothideomycetes
EMD94542.1-701091-Bipolaris maydis C5-Dothideomycetes
KYK57378.1-98403-Drechmeria coniospora-Sordariomycetes
XP 014539081.1-1170230-Penicillium digitatum Pd1-Eurotiomycetes
KUM59361.1-48697-Penicillium freii-Eurotiomycetes
XP 018075919.1-149040-Phialocephala scopiformis-Leotiomycetes
OCK82927.1-1314670-Lepidopterella palustris-Dothideomycetes
OCK74982.1-1314670-Lepidopterella palustris-Dothideomycetes
OCK73526.1-1314670-Lepidopterella palustris-Dothideomycetes
XP 018157389.1-759273-Colletotrichum higginsian-Sordariomycetes
KZZ96510.1-1081109-Aschersonia aleyrodis RCE-Sordariomycetes
KDN69202.1-1173701-Colletotrichum sublineola-Sordariomycetes
XP 008098964.1-645133-Colletotrichum graminicol-Sordariomycetes
XP 007286629.1-1213859-Colletotrichum gloeospori-Sordariomycetes
EQB52534.1-1237896-Colletotrichum gloeospori-Sordariomycetes
KDN60187.1-1173701-Colletotrichum sublineola-Sordariomycetes
KXH47916.1-1209931-Colletotrichum salicis-Sordariomycetes
OCW42213.1-158607-Diaporthe helianthi-Sordariomycetes
KXH50180.1-703756-Colletotrichum simmondsii-Sordariomycetes
OIW33137.1-1408157-Coniochaeta ligniaria NRR-Sordariomycetes
KIM94742.1-913774-Oidiodendron maius Zn-Leotiomycetes
KXJ85092.1-196109-Microdochium bolleyi-Sordariomycetes
KXJ85939.1-196109-Microdochium bolleyi-Sordariomycetes
XP 001260852.1-331117-Aspergillus fischeri NRRL-Eurotiomycetes
OJJ40738.1-1073089-Aspergillus wentii DTO 13-Eurotiomycetes
XP 001271874.1-344612-Aspergillus clavatus NRRL-Eurotiomycetes
XP 001267016.1-331117-Aspergillus fischeri NRRL-Eurotiomycetes
KMK54731.1-1437362-Aspergillus fumigatus Z5-Eurotiomycetes
XP 751828.1-330879-Aspergillus fumigatus Af2-Eurotiomycetes
EME39031.1-675120-Dothistroma septosporum N-Dothideomycetes
AAC49196.1-162425-Aspergillus nidulans-Eurotiomycetes
XP 681087.1-227321-Aspergillus nidulans FGSC-Eurotiomycetes
KKK21817.1-308745-Aspergillus rambellii-Eurotiomycetes
XP 020053364.1-690307-Aspergillus aculeatus ATC-Eurotiomycetes
KND91002.1-1163406-Tolypocladium ophioglosso-Sordariomycetes
OAL39677.1-856822-Fonsecaea nubica-Eurotiomycetes
OAG34162.1-254056-Fonsecaea monophora-Eurotiomycetes
XP 013280246.1-1442368-Fonsecaea pedrosoi CBS 27-Eurotiomycetes
OAG44384.1-254056-Fonsecaea monophora-Eurotiomycetes
KXT06680.1-321146-Mycosphaerella eumusae-Dothideomycetes
XP 018159597.1-759273-Colletotrichum higginsian-Sordariomycetes
OLN90223.1-708187-Colletotrichum chlorophyt-Sordariomycetes
KZL71764.1-1573173-Colletotrichum incanum-Sordariomycetes
KZL63447.1-708197-Colletotrichum tofieldiae-Sordariomycetes
XP 008098516.1-645133-Colletotrichum graminicol-Sordariomycetes
XP 003717434.1-242507-Magnaporthe oryzae 70 15-Sordariomycetes
OOQ86043.1-104259-Penicillium brasilianum-Eurotiomycetes
OCK77653.1-1314670-Lepidopterella palustris-Dothideomycetes
GAD91552.1-1356009-Byssochlamys spectabilis-Eurotiomycetes
OQD73863.1-69771-Penicillium decumbens-Eurotiomycetes
CEJ57600.1-104259-Penicillium brasilianum-Eurotiomycetes
EPS31372.1-933388-Penicillium oxalicum 114-Eurotiomycetes
KMU74509.1-454286-Coccidioides immitis RMSC-Eurotiomycetes
KMM66554.1-454284-Coccidioides posadasii RM-Eurotiomycetes
EFW13723.1-443226-Coccidioides posadasii st-Eurotiomycetes
XP 003069072.1-222929-Coccidioides posadasii C7-Eurotiomycetes
XP 001240202.2-246410-Coccidioides immitis RS-Eurotiomycetes
KMP02584.1-404692-Coccidioides immitis RMSC-Eurotiomycetes
KFZ09804.1-1420914-Pseudogymnoascus sp VKM-Leotiomycetes
OBT43052.1-1622147-Pseudogymnoascus sp WSF-Leotiomycetes
XP 012740108.1-658429-Pseudogymnoascus destruct-Leotiomycetes
KFY12939.1-1420901-Pseudogymnoascus sp VKM-Leotiomycetes
KFY74280.1-1420912-Pseudogymnoascus sp VKM-Leotiomycetes
XP 018132394.1-342668-Pseudogymnoascus verrucos-Leotiomycetes
KFY92053.1-1420913-Pseudogymnoascus sp VKM-Leotiomycetes
KFZ12582.1-1420915-Pseudogymnoascus sp VKM-Leotiomycetes
OQE06029.1-29845-Penicillium vulpinum-Eurotiomycetes
OQE43268.1-36646-Penicillium coprophilum-Eurotiomycetes
OGM48142.1-109264-Aspergillus bombycis-Eurotiomycetes
KJK61356.1-1403190-Aspergillus parasiticus S-Eurotiomycetes
KOC10931.1-1392242-Aspergillus flavus AF70-Eurotiomycetes
OOO07101.1-5062-Aspergillus oryzae-Eurotiomycetes
EIT78191.1-1160506-Aspergillus oryzae 3 042-Eurotiomycetes
BAE65144.1-510516-Aspergillus oryzae RIB40-Eurotiomycetes
BAJ04387.1-5062-Aspergillus oryzae-Eurotiomycetes
XP 001826277.2-510516-Aspergillus oryzae RIB40-Eurotiomycetes

S5F

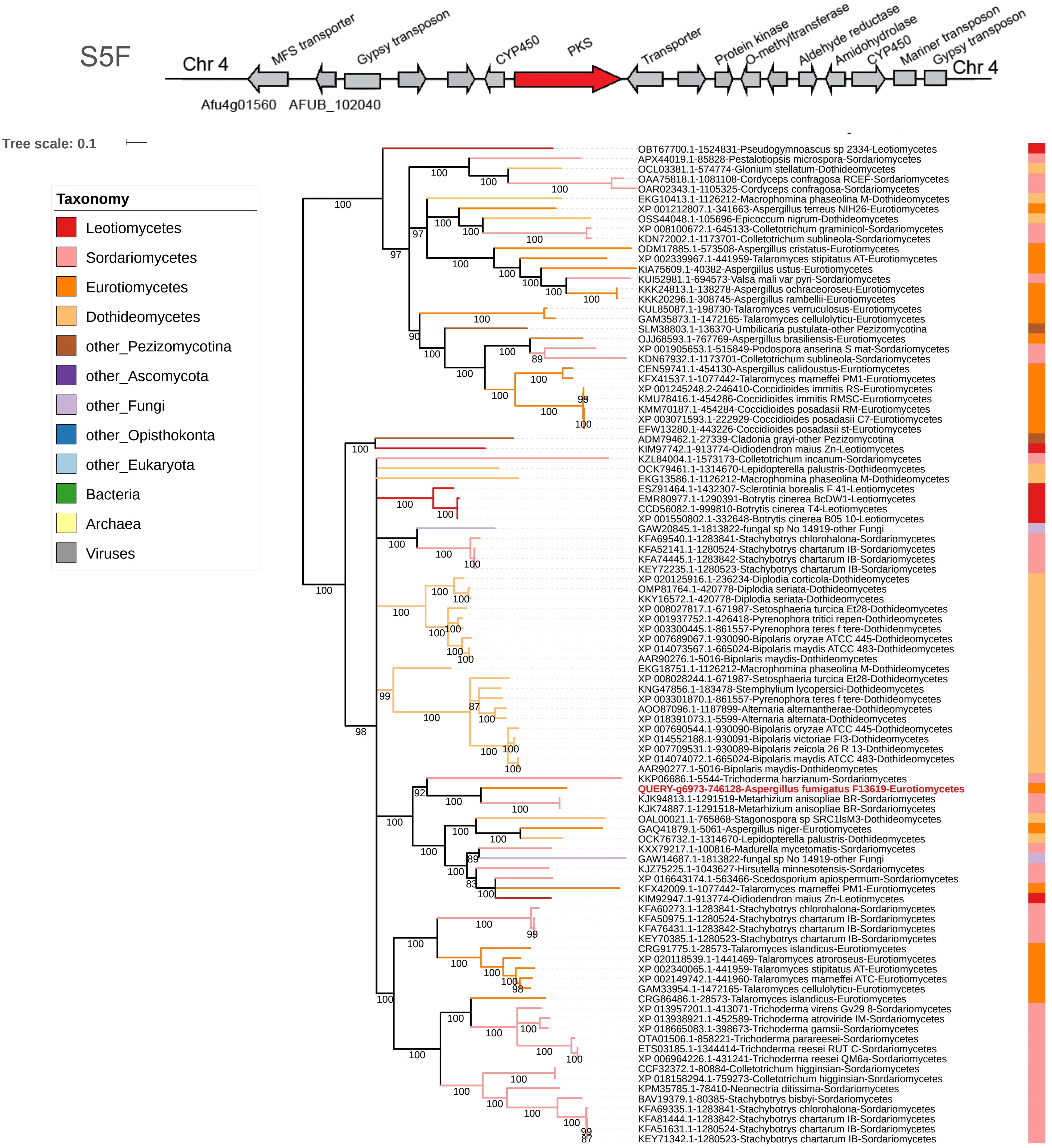

Chr 4
MFS transporter
Gypsy transposon
CYP450
PKS
Transporter
Protein kinase
O-methyltransferase
Aldehyde reductase
Amidohydrolase
CYP450
Mariner transposon
Gypsy transposon
Chr 4
Afu4g01560
AFUB_102040
Tree scale: 0.1
Taxonomy
Leotiomycetes
Sordariomycetes
Eurotiomycetes
Dothideomycetes
other_Pezizomycotina
other_Ascomycota
other_Fungi
other_Opisthokonta
other_Eukaryota
Bacteria
Archaea
Viruses
OBT67700.1-1524831-Pseudogymnoascus sp 2334-Leotiomycetes
APX44019.1-85828-Pestalotiopsis microspora-Sordariomycetes
OCL03381.1-574774-Glonium stellatum-Dothideomycetes
OAA75818.1-1081108-Cordyceps confragosa RCEF-Sordariomycetes
OAR02343.1-1105325-Cordyceps confragosa-Sordariomycetes
EKG10413.1-1126212-Macrophomina phaseolina M-Dothideomycetes
XP 001212807.1-341663-Aspergillus terreus NIH26-Eurotiomycetes
OSS44048.1-105696-Epicoccum nigrum-Dothideomycetes
XP 008100672.1-645133-Colletotrichum graminicol-Sordariomycetes
KDN72002.1-1173701-Colletotrichum sublineola-Sordariomycetes
ODM17885.1-573508-Aspergillus cristatus-Eurotiomycetes
XP 002339967.1-441959-Talaromyces stipitatus AT-Eurotiomycetes
KIA75609.1-40382-Aspergillus ustus-Eurotiomycetes
KUI52981.1-694573-Valsa mali var pyri-Sordariomycetes
KKK24813.1-138278-Aspergillus ochraceoroseu-Eurotiomycetes
KKK20296.1-308745-Aspergillus rambellii-Eurotiomycetes
KUL85087.1-198730-Talaromyces verruculosus-Eurotiomycetes
GAM35873.1-1472165-Talaromyces cellulolyticu-Eurotiomycetes
SLM38803.1-136370-Umbilicaria pustulata-other Pezizomycotina
OJJ68593.1-767769-Aspergillus brasiliensis-Eurotiomycetes
XP 001905653.1-515849-Podospora anserina S mat-Sordariomycetes
KDN67932.1-1173701-Colletotrichum sublineola-Sordariomycetes
CEN59741.1-454130-Aspergillus calidoustus-Eurotiomycetes
KFX41537.1-1077442-Talaromyces marneffei PM1-Eurotiomycetes
XP 001245248.2-246410-Coccidioides immitis RS-Eurotiomycetes
KMU78416.1-454286-Coccidioides immitis RMSC-Eurotiomycetes
KMM70187.1-454284-Coccidioides posadasii RM-Eurotiomycetes
XP 003071593.1-222929-Coccidioides posadasii C7-Eurotiomycetes
EFW13280.1-443226-Coccidioides posadasii st-Eurotiomycetes
ADM79462.1-27339-Cladonia grayi-other Pezizomycotina
KIM97742.1-913774-Oidiodendron maius Zn-Leotiomycetes
KZL84004.1-1573173-Colletotrichum incanum-Sordariomycetes
OCK79461.1-1314670-Lepidopterella palustris-Dothideomycetes
EKG13586.1-1126212-Macrophomina phaseolina M-Dothideomycetes
ESZ91464.1-1432307-Sclerotinia borealis F 41-Leotiomycetes
EMR80977.1-1290391-Botrytis cinerea BcDW1-Leotiomycetes
CCD56082.1-999810-Botrytis cinerea T4-Leotiomycetes
XP 001550802.1-332648-Botrytis cinerea B05 10-Leotiomycetes
GAW20845.1-1813822-fungal sp No 14919-other Fungi
KFA69540.1-1283841-Stachybotrys chlorohalona-Sordariomycetes
KFA52141.1-1280524-Stachybotrys chartarum IB-Sordariomycetes
KFA74445.1-1283842-Stachybotrys chartarum IB-Sordariomycetes
KEY72235.1-1280523-Stachybotrys chartarum IB-Sordariomycetes
XP 020125916.1-236234-Diplodia corticola-Dothideomycetes
OMP81764.1-420778-Diplodia seriata-Dothideomycetes
KKY16572.1-420778-Diplodia seriata-Dothideomycetes
XP 008027817.1-671987-Setosphaeria turcica Et28-Dothideomycetes
XP 001937752.1-426418-Pyrenophora tritici repen-Dothideomycetes
XP 003300445.1-861557-Pyrenophora teres f tere-Dothideomycetes
XP 007689067.1-930090-Bipolaris oryzae ATCC 445-Dothideomycetes
XP 014073567.1-665024-Bipolaris maydis ATCC 483-Dothideomycetes
AAR90276.1-5016-Bipolaris maydis-Dothideomycetes
EKG18751.1-1126212-Macrophomina phaseolina M-Dothideomycetes
XP 008028244.1-671987-Setosphaeria turcica Et28-Dothideomycetes
KNG47856.1-183478-Stemphylium lycopersici-Dothideomycetes
XP 003301870.1-861557-Pyrenophora teres f tere-Dothideomycetes
AOO87096.1-1187899-Alternaria alternantherae-Dothideomycetes
XP 018391073.1-5599-Alternaria alternata-Dothideomycetes
XP 007690544.1-930090-Bipolaris oryzae ATCC 445-Dothideomycetes
XP 014552188.1-930091-Bipolaris victoriae FI3-Dothideomycetes
XP 007709531.1-930089-Bipolaris zeicola 26 R 13-Dothideomycetes
XP 014074072.1-665024-Bipolaris maydis ATCC 483-Dothideomycetes
AAR90277.1-5016-Bipolaris maydis-Dothideomycetes
KKP06686.1-5544-Trichoderma harzianum-Sordariomycetes
QUERY-g6973-746128-Aspergillus fumigatus F13619-Eurotiomycetes
KJK94813.1-1291519-Metarhizium anisopliae BR-Sordariomycetes
KJK74887.1-1291518-Metarhizium anisopliae BR-Sordariomycetes
OAL00021.1-765868-Stagonospora sp SRC1lsM3-Dothideomycetes
GAQ41879.1-5061-Aspergillus niger-Eurotiomycetes
OCK76732.1-1314670-Lepidopterella palustris-Dothideomycetes
KXX79217.1-100816-Madurella mycetomatis-Sordariomycetes
GAW14687.1-1813822-fungal sp No 14919-other Fungi
KJZ75225.1-1043627-Hirsutella minnesotensis-Sordariomycetes
XP 016643174.1-563466-Scedosporium apiospermum-Sordariomycetes
KFX42009.1-1077442-Talaromyces marneffei PM1-Eurotiomycetes
KIM92947.1-913774-Oidiodendron maius Zn-Leotiomycetes
KFA60273.1-1283841-Stachybotrys chlorohalona-Sordariomycetes
KFA50975.1-1280524-Stachybotrys chartarum IB-Sordariomycetes
KFA76431.1-1283842-Stachybotrys chartarum IB-Sordariomycetes
KEY70385.1-1280523-Stachybotrys chartarum IB-Sordariomycetes
CRG91775.1-28573-Talaromyces islandicus-Eurotiomycetes
XP 020118539.1-1441469-Talaromyces atroroseus-Eurotiomycetes
XP 002340065.1-441959-Talaromyces stipitatus AT-Eurotiomycetes
XP 002149742.1-441960-Talaromyces marneffei ATC-Eurotiomycetes
GAM33954.1-1472165-Talaromyces cellulolyticu-Eurotiomycetes
CRG86486.1-28573-Talaromyces islandicus-Eurotiomycetes
XP 013957201.1-413071-Trichoderma virens Gv29 8-Sordariomycetes
XP 013938921.1-452589-Trichoderma atroviride IM-Sordariomycetes
XP 018665083.1-398673-Trichoderma gamsii-Sordariomycetes
OTA01506.1-858221-Trichoderma parareesei-Sordariomycetes
ETS03185.1-1344414-Trichoderma reesei RUT C-Sordariomycetes
XP 006964241.1-431241-Trichoderma reesei QM6a-Sordariomycetes
CCF32372.1-80884-Colletotrichum higginsianum-Sordariomycetes
XP 018158294.1-759273-Colletotrichum higginsianum-Sordariomycetes
KPM35785.1-78410-Neonectria ditissima-Sordariomycetes
BAV19379.1-80385-Stachybotrys bisbyi-Sordariomycetes
KFA69335.1-1283841-Stachybotrys chlorohalona-Sordariomycetes
KFA81444.1-1283842-Stachybotrys chartarum IB-Sordariomycetes
KFA51631.1-1280524-Stachybotrys chartarum IB-Sordariomycetes
KEY71342.1-1280523-Stachybotrys chartarum IB-Sordariomycetes

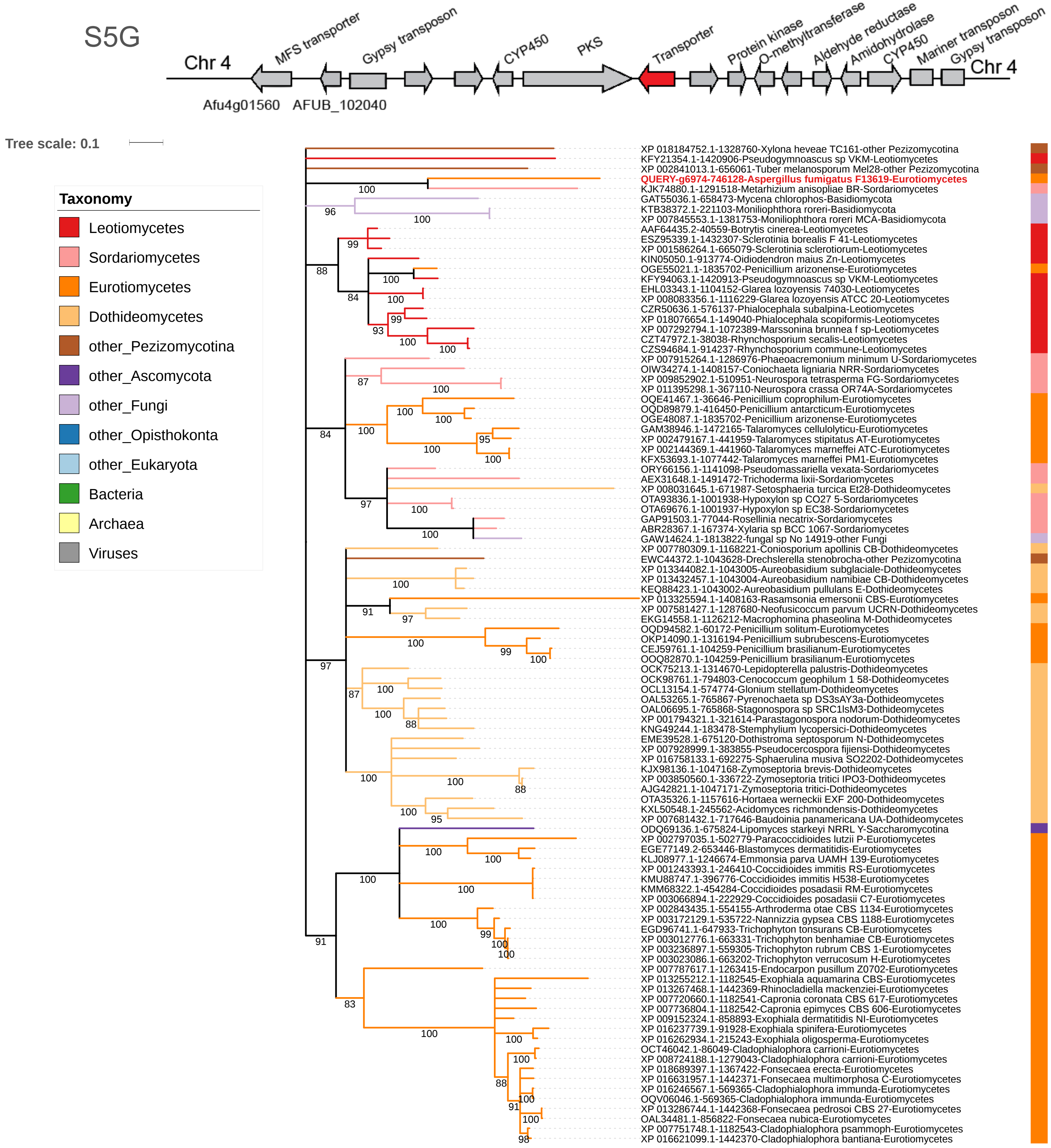

S5G
Chr 4
MFS transporter
Gypsy transposon
CYP450
PKS
Transporter
Protein kinase
O-methyltransferase
Aldehyde reductase
Amidohydrolase
CYP450
Mariner transposon
Gypsy transposon
Chr 4
Afu4g01560
AFUB_102040
Tree scale: 0.1
Taxonomy
Leotiomycetes
Sordariomycetes
Eurotiomycetes
Dothideomycetes
other_Pezizomycotina
other_Ascomycota
other_Fungi
other_Opisthokonta
other_Eukaryota
Bacteria
Archaea
Viruses
XP 018184752.1-1328760-Xylona heveae TC161-other Pezizomycotina
KFY21354.1-1420906-Pseudogymnoascus sp VKM-Leotiomycetes
XP 002841013.1-656061-Tuber melanosporum Mel28-other Pezizomycotina
QUERY-g6974-746128-Aspergillus fumigatus F13619-Eurotiomycetes
KJK74880.1-1291518-Metarhizium anisopliae BR-Sordariomycetes
GAT55036.1-658473-Mycena chlorophos-Basidiomycota
KTB38372.1-221103-Moniliophthora roreri-Basidiomycota
XP 007845553.1-1381753-Moniliophthora roreri MCA-Basidiomycota
AAF64435.2-40559-Botrytis cinerea-Leotiomycetes
ESZ95339.1-1432307-Sclerotinia borealis F 41-Leotiomycetes
XP 001586264.1-665079-Sclerotinia sclerotiorum-Leotiomycetes
KIN05050.1-913774-Oidiodendron maius Zn-Leotiomycetes
OGE55021.1-1835702-Penicillium arizonense-Eurotiomycetes
KFY94063.1-1420913-Pseudogymnoascus sp VKM-Leotiomycetes
EHL03343.1-1104152-Glarea lozoyensis 74030-Leotiomycetes
XP 008083356.1-1116229-Glarea lozoyensis ATCC 20-Leotiomycetes
CZR50636.1-576137-Phialocephala subalpina-Leotiomycetes
XP 018076654.1-149040-Phialocephala scopiformis-Leotiomycetes
XP 007292794.1-1072389-Marssonina brunnea f sp-Leotiomycetes
CZT47972.1-38038-Rhynchosporium secalis-Leotiomycetes
CZS94684.1-914237-Rhynchosporium commune-Leotiomycetes
XP 007915264.1-1286976-Phaeoacremonium minimum U-Sordariomycetes
OIW34274.1-1408157-Coniochaeta ligniaria NRR-Sordariomycetes
XP 009852902.1-510951-Neurospora tetrasperma FG-Sordariomycetes
XP 011395298.1-367110-Neurospora crassa OR74A-Sordariomycetes
OQE41467.1-36646-Penicillium coprophilum-Eurotiomycetes
OQD89879.1-416450-Penicillium antarcticum-Eurotiomycetes
OGE48087.1-1835702-Penicillium arizonense-Eurotiomycetes
GAM38946.1-1472165-Talaromyces cellulolyticu-Eurotiomycetes
XP 002479167.1-441959-Talaromyces stipitatus AT-Eurotiomycetes
XP 002144369.1-441960-Talaromyces marneffei ATC-Eurotiomycetes
KFX53693.1-1077442-Talaromyces marneffei PM1-Eurotiomycetes
ORY66156.1-1141098-Pseudomassariella vexata-Sordariomycetes
AEX31648.1-1491472-Trichoderma lixii-Sordariomycetes
XP 008031645.1-671987-Setosphaeria turcica Et28-Dothideomycetes
OTA93836.1-1001938-Hypoxylon sp CO27 5-Sordariomycetes
OTA69676.1-1001937-Hypoxylon sp EC38-Sordariomycetes
GAP91503.1-77044-Rosellinia necatrix-Sordariomycetes
ABR28367.1-167374-Xylaria sp BCC 1067-Sordariomycetes
GAW14624.1-1813822-fungal sp No 14919-other Fungi
XP 007780309.1-1168221-Coniosporium apollinis CB-Dothideomycetes
EWC44372.1-1043628-Drechslerella stenobrocha-other Pezizomycotina
XP 013344082.1-1043005-Aureobasidium subglaciale-Dothideomycetes
XP 013432457.1-1043004-Aureobasidium namibiae CB-Dothideomycetes
KEQ88423.1-1043002-Aureobasidium pullulans E-Dothideomycetes
XP 013325594.1-1408163-Rasamsonia emersonii CBS-Eurotiomycetes
XP 007581427.1-1287680-Neofusicoccum parvum UCRN-Dothideomycetes
EKG14558.1-1126212-Macrophomina phaseolina M-Dothideomycetes
OQD94582.1-60172-Penicillium solitum-Eurotiomycetes
OKP14090.1-1316194-Penicillium subrubescens-Eurotiomycetes
CEJ59761.1-104259-Penicillium brasilianum-Eurotiomycetes
OOQ82870.1-104259-Penicillium brasilianum-Eurotiomycetes
OCK75213.1-1314670-Lepidopterella palustris-Dothideomycetes
OCK98761.1-794803-Cenococcum geophilum 1 58-Dothideomycetes
OCL13154.1-574774-Glonium stellatum-Dothideomycetes
OAL53265.1-765867-Pyrenochaeta sp DS3sAY3a-Dothideomycetes
OAL06695.1-765868-Stagonospora sp SRC1lsM3-Dothideomycetes
XP 001794321.1-321614-Parastagonospora nodorum-Dothideomycetes
KNG49244.1-183478-Stemphylium lycopersici-Dothideomycetes
EME39528.1-675120-Dothistroma septosporum N-Dothideomycetes
XP 007928999.1-383855-Pseudocercospora fijiensi-Dothideomycetes
XP 016758133.1-692275-Sphaerulina musiva SO2202-Dothideomycetes
KJX98136.1-1047168-Zymoseptoria brevis-Dothideomycetes
XP 003850560.1-336722-Zymoseptoria tritici IPO3-Dothideomycetes
AJG42821.1-1047171-Zymoseptoria tritici-Dothideomycetes
OTA35326.1-1157616-Hortaea werneckii EXF 200-Dothideomycetes
KXL50548.1-245562-Acidomyces richmondensis-Dothideomycetes
XP 007681432.1-717646-Baudoinia panamericana UA-Dothideomycetes
ODQ69136.1-675824-Lipomyces starkeyi NRRL Y-Saccharomycotina
XP 002797035.1-502779-Paracoccidioides lutzii P-Eurotiomycetes
EGE77149.2-653446-Blastomyces dermatitidis-Eurotiomycetes
KLJ08977.1-1246674-Emmonsia parva UAMH 139-Eurotiomycetes
XP 001243393.1-246410-Coccidioides immitis RS-Eurotiomycetes
KMU88747.1-396776-Coccidioides immitis H538-Eurotiomycetes
KMM68322.1-454284-Coccidioides posadasii RM-Eurotiomycetes
XP 003066894.1-222929-Coccidioides posadasii C7-Eurotiomycetes
XP 002843435.1-554155-Arthroderma otae CBS 1134-Eurotiomycetes
XP 003172129.1-535722-Nannizzia gypsea CBS 1188-Eurotiomycetes
EGD96741.1-647933-Trichophyton tonsurans CB-Eurotiomycetes
XP 003012776.1-663331-Trichophyton benhamiae CB-Eurotiomycetes
XP 003236897.1-559305-Trichophyton rubrum CBS 1-Eurotiomycetes
XP 003023086.1-663202-Trichophyton verrucosum H-Eurotiomycetes
XP 007787617.1-1263415-Endocarpon pusillum Z0702-Eurotiomycetes
XP 013255212.1-1182545-Exophiala aquamarina CBS-Eurotiomycetes
XP 013267468.1-1442369-Rhinocladiella mackenziei-Eurotiomycetes
XP 007720660.1-1182541-Capronia coronata CBS 617-Eurotiomycetes
XP 007736804.1-1182542-Capronia epimyces CBS 606-Eurotiomycetes
XP 009152324.1-858893-Exophiala dermatitidis NI-Eurotiomycetes
XP 016237739.1-91928-Exophiala spinifera-Eurotiomycetes
XP 016262934.1-215243-Exophiala oligosperma-Eurotiomycetes
OCT46042.1-86049-Cladophialophora carrioni-Eurotiomycetes
XP 008724188.1-1279043-Cladophialophora carrioni-Eurotiomycetes
XP 018689397.1-1367422-Fonsecaea erecta-Eurotiomycetes
XP 016631957.1-1442371-Fonsecaea multimorphosa C-Eurotiomycetes
XP 016246567.1-569365-Cladophialophora immunda-Eurotiomycetes
OQV06046.1-569365-Cladophialophora immunda-Eurotiomycetes
XP 013286744.1-1442368-Fonsecaea pedrosoi CBS 27-Eurotiomycetes
OAL34481.1-856822-Fonsecaea nubica-Eurotiomycetes
XP 007751748.1-1182543-Cladophialophora psammoph-Eurotiomycetes
XP 016621099.1-1442370-Cladophialophora bantiana-Eurotiomycetes

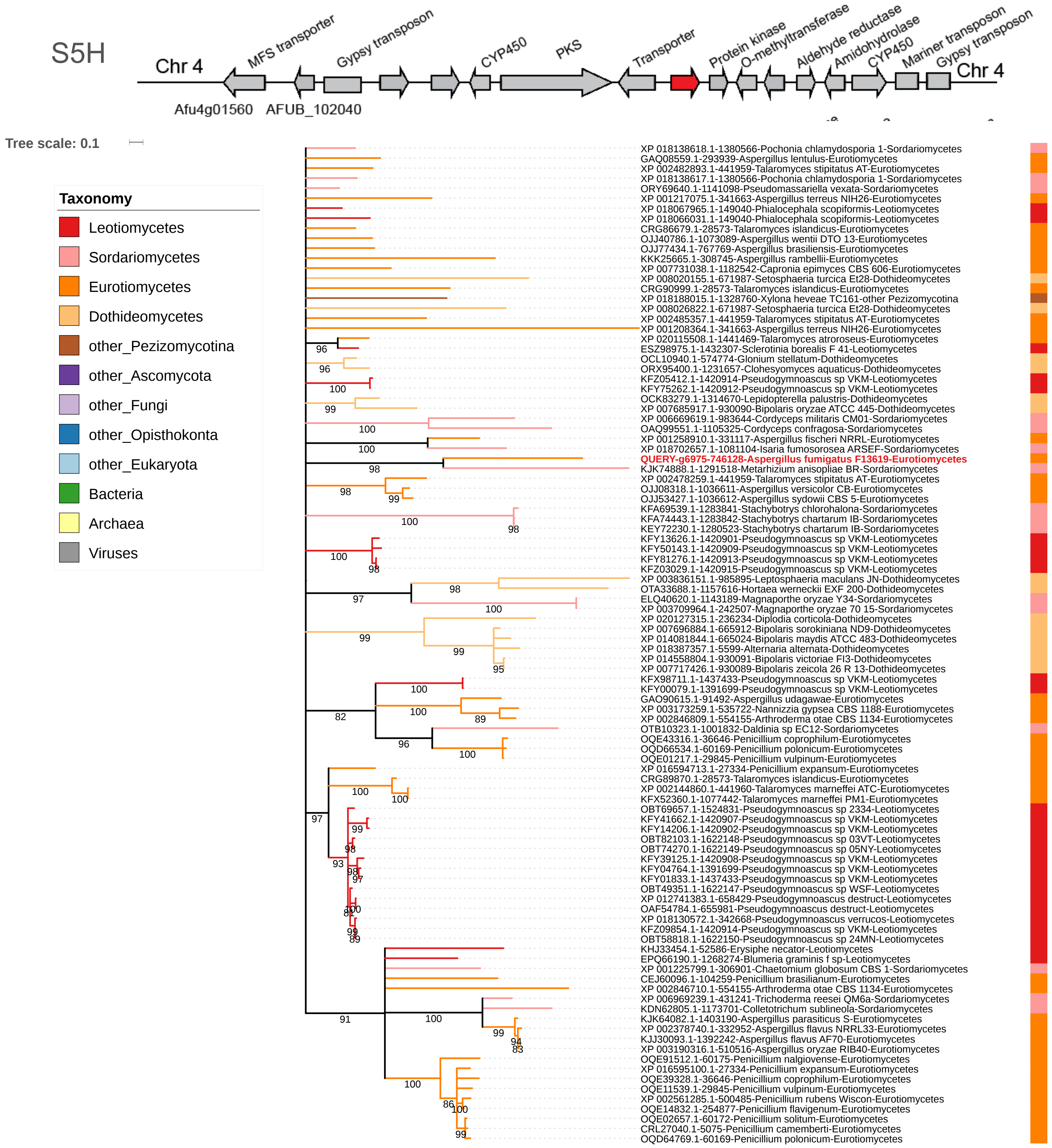
S5H
Chr 4
MFS transporter
Gypsy transposon
CYP450
PKS
Transporter
Protein kinase
O-methyltransferase
Aldehyde reductase
Amidohydrolase
CYP450
Mariner transposon
Gypsy transposon
Chr 4
Afu4g01560
AFUB_102040
Tree scale: 0.1
Taxonomy
Leotiomycetes
Sordariomycetes
Eurotiomycetes
Dothideomycetes
other_Pezizomycotina
other_Ascomycota
other_Fungi
other_Opisthokonta
other_Eukaryota
Bacteria
Archaea
Viruses
XP 018138618.1-1380566-Pochonia chlamydosporia 1-Sordariomycetes
GAQ08559.1-293939-Aspergillus lentulus-Eurotiomycetes
XP 002482893.1-441959-Talaromyces stipitatus AT-Eurotiomycetes
XP 018138617.1-1380566-Pochonia chlamydosporia 1-Sordariomycetes
ORY69640.1-1141098-Pseudomassariella vexata-Sordariomycetes
XP 001217075.1-341663-Aspergillus terreus NIH26-Eurotiomycetes
XP 018067965.1-149040-Phialocephala scopiformis-Leotiomycetes
XP 018066031.1-149040-Phialocephala scopiformis-Leotiomycetes
CRG86679.1-28573-Talaromyces islandicus-Eurotiomycetes
OJJ40786.1-1073089-Aspergillus wentii DTO 13-Eurotiomycetes
OJJ77434.1-767769-Aspergillus brasiliensis-Eurotiomycetes
KKK25665.1-308745-Aspergillus rambellii-Eurotiomycetes
XP 007731038.1-1182542-Capronia epimyces CBS 606-Eurotiomycetes
XP 008020155.1-671987-Setosphaeria turcica Et28-Dothideomycetes
CRG90999.1-28573-Talaromyces islandicus-Eurotiomycetes
XP 018188015.1-1328760-Xylona heveae TC161-other Pezizomycotina
XP 008026822.1-671987-Setosphaeria turcica Et28-Dothideomycetes
XP 002485357.1-441959-Talaromyces stipitatus AT-Eurotiomycetes
XP 001208364.1-341663-Aspergillus terreus NIH26-Eurotiomycetes
XP 020115508.1-1441469-Talaromyces atroroseus-Eurotiomycetes
ESZ98975.1-1432307-Sclerotinia borealis F 41-Leotiomycetes
OCL10940.1-574774-Glonium stellatum-Dothideomycetes
ORX95400.1-1231657-Clohesyomyces aquaticus-Dothideomycetes
KFZ05412.1-1420914-Pseudogymnoascus sp VKM-Leotiomycetes
KFY75262.1-1420912-Pseudogymnoascus sp VKM-Leotiomycetes
OCK83279.1-1314670-Lepidopterella palustris-Dothideomycetes
XP 007685917.1-930090-Bipolaris oryzae ATCC 445-Dothideomycetes
XP 006669619.1-983644-Cordyceps militaris CM01-Sordariomycetes
OAQ99551.1-1105325-Cordyceps confragosa-Sordariomycetes
XP 001258910.1-331117-Aspergillus fischeri NRRL-Eurotiomycetes
XP 018702657.1-1081104-Isaria fumosorosea ARSEF-Sordariomycetes
QUERY-g6975-746128-Aspergillus fumigatus F13619-Eurotiomycetes
KJK74888.1-1291518-Metarhizium anisopliae BR-Sordariomycetes
XP 002478259.1-441959-Talaromyces stipitatus AT-Eurotiomycetes
OJJ08318.1-1036611-Aspergillus versicolor CB-Eurotiomycetes
OJJ53427.1-1036612-Aspergillus sydowii CBS 5-Eurotiomycetes
KFA69539.1-1283841-Stachybotrys chlorohalona-Sordariomycetes
KFA74443.1-1283842-Stachybotrys chartarum IB-Sordariomycetes
KEY72230.1-1280523-Stachybotrys chartarum IB-Sordariomycetes
KFY13626.1-1420901-Pseudogymnoascus sp VKM-Leotiomycetes
KFY50143.1-1420909-Pseudogymnoascus sp VKM-Leotiomycetes
KFY81276.1-1420913-Pseudogymnoascus sp VKM-Leotiomycetes
KFZ03029.1-1420915-Pseudogymnoascus sp VKM-Leotiomycetes
XP 003836151.1-985895-Leptosphaeria maculans JN-Dothideomycetes
OTA33688.1-1157616-Hortaea werneckii EXF 200-Dothideomycetes
ELQ40620.1-1143189-Magnaporthe oryzae Y34-Sordariomycetes
XP 003709964.1-242507-Magnaporthe oryzae 70 15-Sordariomycetes
XP 020127315.1-236234-Diplodia corticola-Dothideomycetes
XP 007696884.1-665912-Bipolaris sorokiniana ND9-Dothideomycetes
XP 014081844.1-665024-Bipolaris maydis ATCC 483-Dothideomycetes
XP 018387357.1-5599-Alternaria alternata-Dothideomycetes
XP 014558804.1-930091-Bipolaris victoriae FI3-Dothideomycetes
XP 007717426.1-930089-Bipolaris zeicola 26 R 13-Dothideomycetes
KFX98711.1-1437433-Pseudogymnoascus sp VKM-Leotiomycetes
KFY00079.1-1391699-Pseudogymnoascus sp VKM-Leotiomycetes
GAO90615.1-91492-Aspergillus udagawae-Eurotiomycetes
XP 003173259.1-535722-Nannizzia gypsea CBS 1188-Eurotiomycetes
XP 002846809.1-554155-Arthroderma otae CBS 1134-Eurotiomycetes
OTB10323.1-1001832-Daldinia sp EC12-Sordariomycetes
OQE43316.1-36646-Penicillium coprophilum-Eurotiomycetes
OQD66534.1-60169-Penicillium polonicum-Eurotiomycetes
OQE01217.1-29845-Penicillium vulpinum-Eurotiomycetes
XP 016594713.1-27334-Penicillium expansum-Eurotiomycetes
CRG89870.1-28573-Talaromyces islandicus-Eurotiomycetes
XP 002144860.1-441960-Talaromyces marneffei ATC-Eurotiomycetes
KFX52360.1-1077442-Talaromyces marneffei PM1-Eurotiomycetes
OBT69657.1-1524831-Pseudogymnoascus sp 2334-Leotiomycetes
KFY41662.1-1420907-Pseudogymnoascus sp VKM-Leotiomycetes
KFY14206.1-1420902-Pseudogymnoascus sp VKM-Leotiomycetes
OBT82103.1-1622148-Pseudogymnoascus sp 03VT-Leotiomycetes
OBT74270.1-1622149-Pseudogymnoascus sp 05NY-Leotiomycetes
KFY39125.1-1420908-Pseudogymnoascus sp VKM-Leotiomycetes
KFY04764.1-1391699-Pseudogymnoascus sp VKM-Leotiomycetes
KFY01833.1-1437433-Pseudogymnoascus sp VKM-Leotiomycetes
OBT49351.1-1622147-Pseudogymnoascus sp WSF-Leotiomycetes
XP 012741383.1-658429-Pseudogymnoascus destruct-Leotiomycetes
OAF54784.1-655981-Pseudogymnoascus destruct-Leotiomycetes
XP 018130572.1-342668-Pseudogymnoascus verrucos-Leotiomycetes
KFZ09854.1-1420914-Pseudogymnoascus sp VKM-Leotiomycetes
OBT58818.1-1622150-Pseudogymnoascus sp 24MN-Leotiomycetes
KHJ33454.1-52586-Erysiphe necator-Leotiomycetes
EPQ66190.1-1268274-Blumeria graminis f sp-Leotiomycetes
XP 001225799.1-306901-Chaetomium globosum CBS 1-Sordariomycetes
CEJ60096.1-104259-Penicillium brasilianum-Eurotiomycetes
XP 002846710.1-554155-Arthroderma otae CBS 1134-Eurotiomycetes
XP 006969239.1-431241-Trichoderma reesei QM6a-Sordariomycetes
KDN62805.1-1173701-Colletotrichum sublineola-Sordariomycetes
KJK64082.1-1403190-Aspergillus parasiticus S-Eurotiomycetes
XP 002378740.1-332952-Aspergillus flavus NRRL33-Eurotiomycetes
KJJ30093.1-1392242-Aspergillus flavus AF70-Eurotiomycetes
XP 003190316.1-510516-Aspergillus oryzae RIB40-Eurotiomycetes
OQE91512.1-60175-Penicillium nalgiovense-Eurotiomycetes
XP 016595100.1-27334-Penicillium expansum-Eurotiomycetes
OQE39328.1-36646-Penicillium coprophilum-Eurotiomycetes
OQE11539.1-29845-Penicillium vulpinum-Eurotiomycetes
XP 002561285.1-500485-Penicillium rubens Wiscon-Eurotiomycetes
OQE14832.1-254877-Penicillium flavigenum-Eurotiomycetes
OQE02657.1-60172-Penicillium solitum-Eurotiomycetes
CRL27040.1-5075-Penicillium camemberti-Eurotiomycetes
OQD64769.1-60169-Penicillium polonicum-Eurotiomycetes

S5I

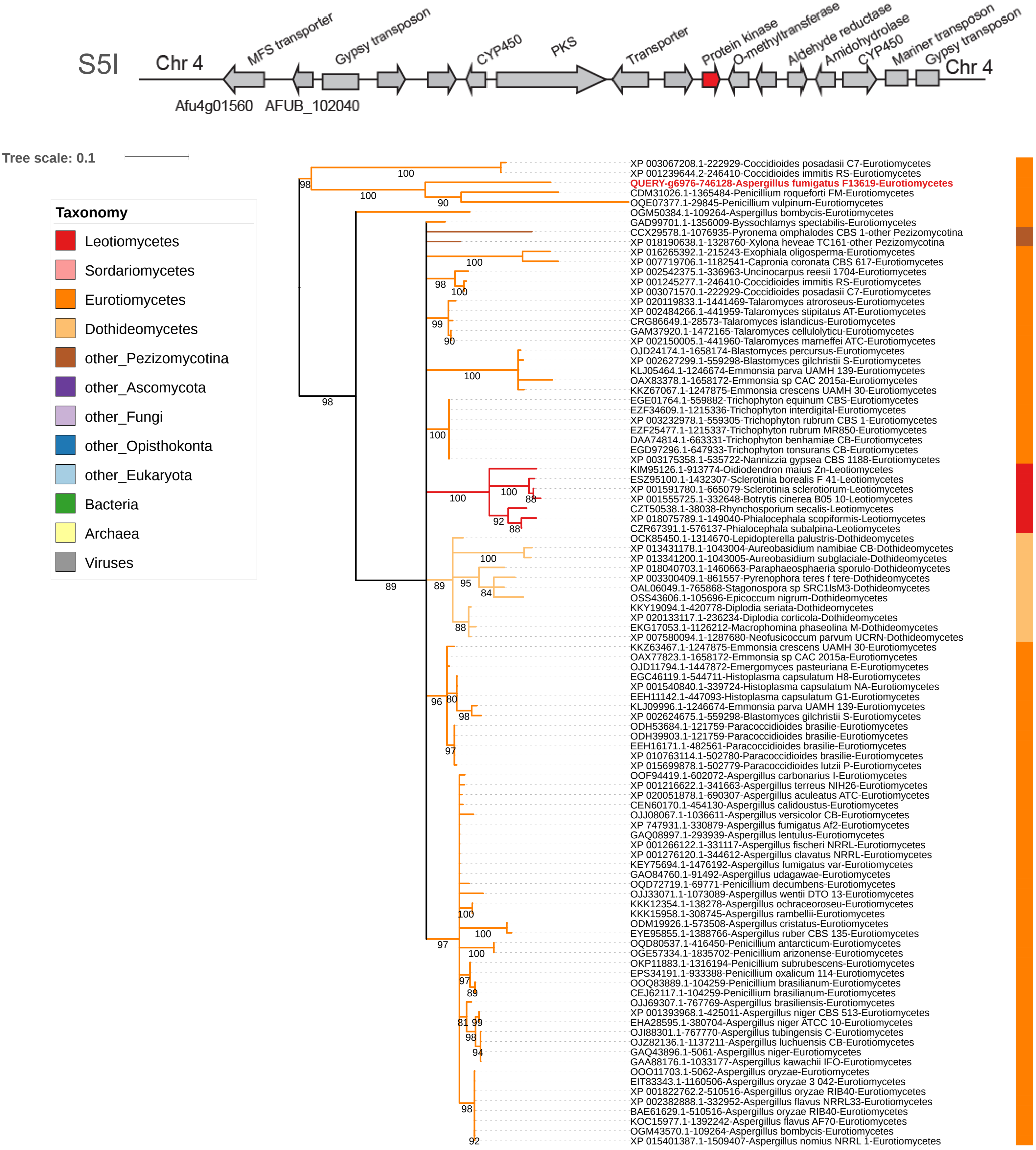

Chr 4
MFS transporter
Gypsy transposon
CYP450
PKS
Transporter
Protein kinase
O-methyltransferase
Aldehyde reductase
Amidohydrolase
CYP450
Mariner transposon
Gypsy transposon
Chr 4
Afu4g01560
AFUB_102040
Tree scale: 0.1
Taxonomy
Leotiomycetes
Sordariomycetes
Eurotiomycetes
Dothideomycetes
other_Pezizomycotina
other_Ascomycota
other_Fungi
other_Opisthokonta
other_Eukaryota
Bacteria
Archaea
Viruses
XP 003067208.1-222929-Coccidioides posadasii C7-Eurotiomycetes
XP 001239644.2-246410-Coccidioides immitis RS-Eurotiomycetes
QUERY-g6976-746128-Aspergillus fumigatus F13619-Eurotiomycetes
CDM31026.1-1365484-Penicillium roqueforti FM-Eurotiomycetes
OQE07377.1-29845-Penicillium vulpinum-Eurotiomycetes
OGM50384.1-109264-Aspergillus bombycis-Eurotiomycetes
GAD99701.1-1356009-Byssochlamys spectabilis-Eurotiomycetes
CCX29578.1-1076935-Pyronema omphalodes CBS 1-other Pezizomycotina
XP 018190638.1-1328760-Xylona heveae TC161-other Pezizomycotina
XP 016265392.1-215243-Exophiala oligosperma-Eurotiomycetes
XP 007719706.1-1182541-Capronia coronata CBS 617-Eurotiomycetes
XP 002542375.1-336963-Uncinocarpus reesii 1704-Eurotiomycetes
XP 001245277.1-246410-Coccidioides immitis RS-Eurotiomycetes
XP 003071570.1-222929-Coccidioides posadasii C7-Eurotiomycetes
XP 020119833.1-1441469-Talaromyces atroroseus-Eurotiomycetes
XP 002484266.1-441959-Talaromyces stipitatus AT-Eurotiomycetes
CRG86649.1-28573-Talaromyces islandicus-Eurotiomycetes
GAM37920.1-1472165-Talaromyces cellulolyticu-Eurotiomycetes
XP 002150005.1-441960-Talaromyces marneffei ATC-Eurotiomycetes
OJD24174.1-1658174-Blastomyces percursus-Eurotiomycetes
XP 002627299.1-559298-Blastomyces gilchristii S-Eurotiomycetes
KLJ05464.1-1246674-Emmonsia parva UAMH 139-Eurotiomycetes
OAX83378.1-1658172-Emmonsia sp CAC 2015a-Eurotiomycetes
KKZ67067.1-1247875-Emmonsia crescens UAMH 30-Eurotiomycetes
EGE01764.1-559882-Trichophyton equinum CBS-Eurotiomycetes
EZF34609.1-1215336-Trichophyton interdigital-Eurotiomycetes
XP 003232978.1-559305-Trichophyton rubrum CBS 1-Eurotiomycetes
EZF25477.1-1215337-Trichophyton rubrum MR850-Eurotiomycetes
DAA74814.1-663331-Trichophyton benhamiae CB-Eurotiomycetes
EGD97296.1-647933-Trichophyton tonsurans CB-Eurotiomycetes
XP 003175358.1-535722-Nannizzia gypsea CBS 1188-Eurotiomycetes
KIM95126.1-913774-Oidiodendron maius Zn-Leotiomycetes
ESZ95100.1-1432307-Sclerotinia borealis F 41-Leotiomycetes
XP 001591780.1-665079-Sclerotinia sclerotiorum-Leotiomycetes
XP 001555725.1-332648-Botrytis cinerea B05 10-Leotiomycetes
CZT50538.1-38038-Rhynchosporium secalis-Leotiomycetes
XP 018075789.1-149040-Phialocephala scopiformis-Leotiomycetes
CZR67391.1-576137-Phialocephala subalpina-Leotiomycetes
OCK85450.1-1314670-Lepidopterella palustris-Dothideomycetes
XP 013431178.1-1043004-Aureobasidium namibiae CB-Dothideomycetes
XP 013341200.1-1043005-Aureobasidium subglaciale-Dothideomycetes
XP 018040703.1-1460663-Paraphaeosphaeria sporulo-Dothideomycetes
XP 003300409.1-861557-Pyrenophora teres f tere-Dothideomycetes
OAL06049.1-765868-Stagonospora sp SRC1lsM3-Dothideomycetes
OSS43606.1-105696-Epicoccum nigrum-Dothideomycetes
KKY19094.1-420778-Diplodia seriata-Dothideomycetes
XP 020133117.1-236234-Diplodia corticola-Dothideomycetes
EKG17053.1-1126212-Macrophomina phaseolina M-Dothideomycetes
XP 007580094.1-1287680-Neofusicoccum parvum UCRN-Dothideomycetes
KKZ63467.1-1247875-Emmonsia crescens UAMH 30-Eurotiomycetes
OAX77823.1-1658172-Emmonsia sp CAC 2015a-Eurotiomycetes
OJD11794.1-1447872-Emergomyces pasteuriana E-Eurotiomycetes
EGC46119.1-544711-Histoplasma capsulatum H8-Eurotiomycetes
XP 001540840.1-339724-Histoplasma capsulatum NA-Eurotiomycetes
EEH11142.1-447093-Histoplasma capsulatum G1-Eurotiomycetes
KLJ09996.1-1246674-Emmonsia parva UAMH 139-Eurotiomycetes
XP 002624675.1-559298-Blastomyces gilchristii S-Eurotiomycetes
ODH53684.1-121759-Paracoccidioides brasilie-Eurotiomycetes
ODH39903.1-121759-Paracoccidioides brasilie-Eurotiomycetes
EEH16171.1-482561-Paracoccidioides brasilie-Eurotiomycetes
XP 010763114.1-502780-Paracoccidioides brasilie-Eurotiomycetes
XP 015699878.1-502779-Paracoccidioides lutzii P-Eurotiomycetes
OOF94419.1-602072-Aspergillus carbonarius I-Eurotiomycetes
XP 001216622.1-341663-Aspergillus terreus NIH26-Eurotiomycetes
XP 020051878.1-690307-Aspergillus aculeatus ATC-Eurotiomycetes
CEN60170.1-454130-Aspergillus calidoustus-Eurotiomycetes
OJJ08067.1-1036611-Aspergillus versicolor CB-Eurotiomycetes
XP 747931.1-330879-Aspergillus fumigatus Af2-Eurotiomycetes
GAQ08997.1-293939-Aspergillus lentulus-Eurotiomycetes
XP 001266122.1-331117-Aspergillus fischeri NRRL-Eurotiomycetes
XP 001276120.1-344612-Aspergillus clavatus NRRL-Eurotiomycetes
KEY75694.1-1476192-Aspergillus fumigatus var-Eurotiomycetes
GAO84760.1-91492-Aspergillus udagawae-Eurotiomycetes
OQD72719.1-69771-Penicillium decumbens-Eurotiomycetes
OJJ33071.1-1073089-Aspergillus wentii DTO 13-Eurotiomycetes
KKK12354.1-138278-Aspergillus ochraceoroseu-Eurotiomycetes
KKK15958.1-308745-Aspergillus rambellii-Eurotiomycetes
ODM19926.1-573508-Aspergillus cristatus-Eurotiomycetes
EYE95855.1-1388766-Aspergillus ruber CBS 135-Eurotiomycetes
OQD80537.1-416450-Penicillium antarcticum-Eurotiomycetes
OGE57334.1-1835702-Penicillium arizonense-Eurotiomycetes
OKP11883.1-1316194-Penicillium subrubescens-Eurotiomycetes
EPS34191.1-933388-Penicillium oxalicum 114-Eurotiomycetes
OOQ83889.1-104259-Penicillium brasilianum-Eurotiomycetes
CEJ62117.1-104259-Penicillium brasilianum-Eurotiomycetes
OJJ69307.1-767769-Aspergillus brasiliensis-Eurotiomycetes
XP 001393968.1-425011-Aspergillus niger CBS 513-Eurotiomycetes
EHA28595.1-380704-Aspergillus niger ATCC 10-Eurotiomycetes
OJI88301.1-767770-Aspergillus tubingensis C-Eurotiomycetes
OJZ82136.1-1137211-Aspergillus luchuensis CB-Eurotiomycetes
GAQ43896.1-5061-Aspergillus niger-Eurotiomycetes
GAA88176.1-1033177-Aspergillus kawachii IFO-Eurotiomycetes
OOO11703.1-5062-Aspergillus oryzae-Eurotiomycetes
EIT83343.1-1160506-Aspergillus oryzae 3 042-Eurotiomycetes
XP 001822762.2-510516-Aspergillus oryzae RIB40-Eurotiomycetes
XP 002382888.1-332952-Aspergillus flavus NRRL33-Eurotiomycetes
BAE61629.1-510516-Aspergillus oryzae RIB40-Eurotiomycetes
KOC15977.1-1392242-Aspergillus flavus AF70-Eurotiomycetes
OGM43570.1-109264-Aspergillus bombycis-Eurotiomycetes
XP 015401387.1-1509407-Aspergillus nomius NRRL 1-Eurotiomycetes

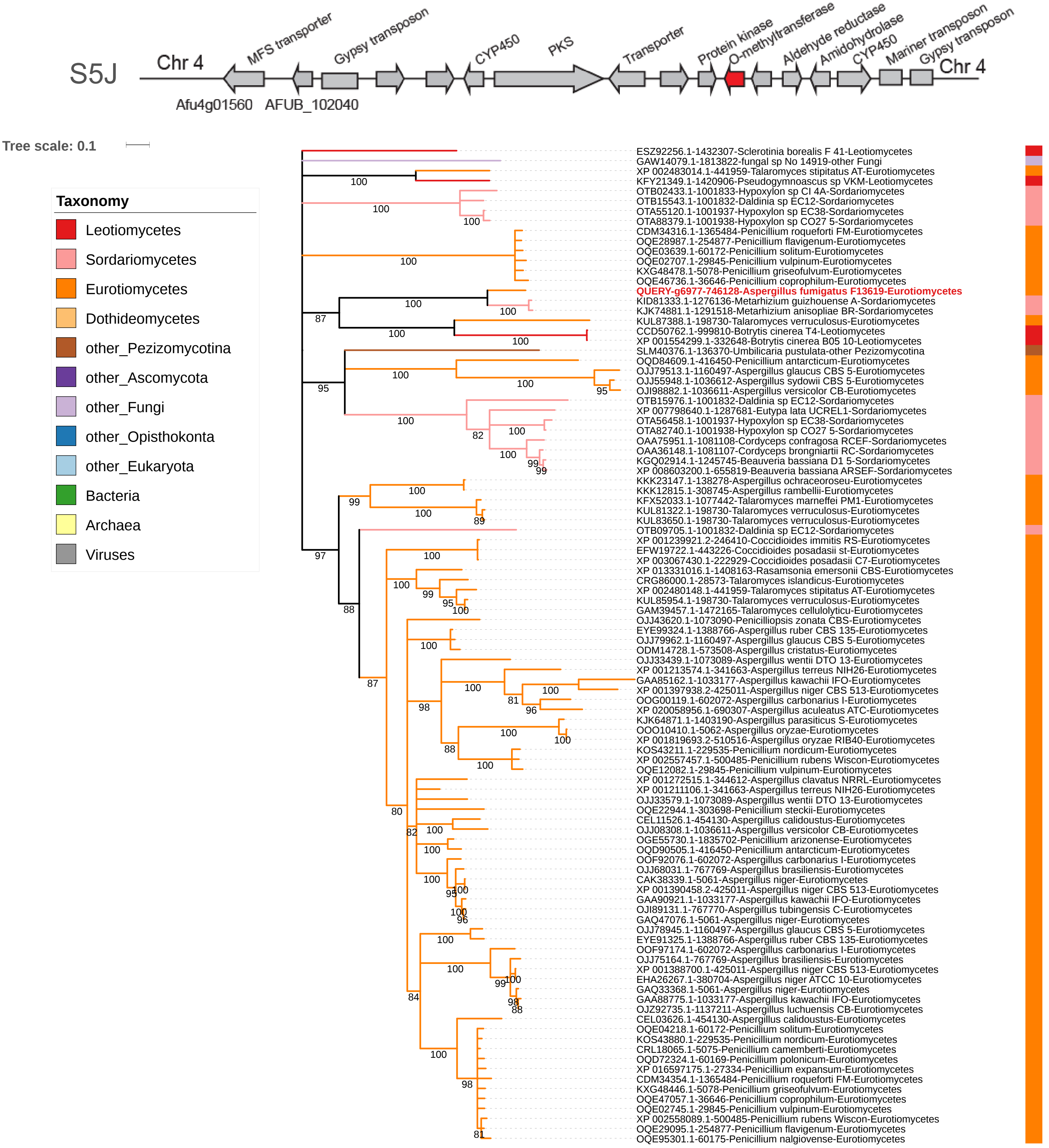
S5J
Chr 4
MFS transporter
Gypsy transposon
CYP450
PKS
Transporter
Protein kinase
O-methyltransferase
Aldehyde reductase
Amidohydrolase
CYP450
Mariner transposon
Gypsy transposon
Chr 4
Afu4g01560
AFUB_102040
Tree scale: 0.1
Taxonomy
Leotiomycetes
Sordariomycetes
Eurotiomycetes
Dothideomycetes
other_Pezizomycotina
other_Ascomycota
other_Fungi
other_Opisthokonta
other_Eukaryota
Bacteria
Archaea
Viruses
ESZ92256.1-1432307-Sclerotinia borealis F 41-Leotiomycetes
GAW14079.1-1813822-fungal sp No 14919-other Fungi
XP 002483014.1-441959-Talaromyces stipitatus AT-Eurotiomycetes
KFY21349.1-1420906-Pseudogymnoascus sp VKM-Leotiomycetes
OTB02433.1-1001833-Hypoxylon sp CI 4A-Sordariomycetes
OTB15543.1-1001832-Daldinia sp EC12-Sordariomycetes
OTA55120.1-1001937-Hypoxylon sp EC38-Sordariomycetes
OTA88379.1-1001938-Hypoxylon sp CO27 5-Sordariomycetes
CDM34316.1-1365484-Penicillium roqueforti FM-Eurotiomycetes
OQE28987.1-254877-Penicillium flavigenum-Eurotiomycetes
OQE03639.1-60172-Penicillium solitum-Eurotiomycetes
OQE02707.1-29845-Penicillium vulpinum-Eurotiomycetes
KXG48478.1-5078-Penicillium griseofulvum-Eurotiomycetes
OQE46736.1-36646-Penicillium coprophilum-Eurotiomycetes
QUERY-g6977-746128-Aspergillus fumigatus F13619-Eurotiomycetes
KID81333.1-1276136-Metarhizium guizhouense A-Sordariomycetes
KJK74881.1-1291518-Metarhizium anisopliae BR-Sordariomycetes
KUL87388.1-198730-Talaromyces verruculosus-Eurotiomycetes
CCD50762.1-999810-Botrytis cinerea T4-Leotiomycetes
XP 001554299.1-332648-Botrytis cinerea B05 10-Leotiomycetes
SLM40376.1-136370-Umbilicaria pustulata-other Pezizomycotina
OQD84609.1-416450-Penicillium antarcticum-Eurotiomycetes
OJJ79513.1-1160497-Aspergillus glaucus CBS 5-Eurotiomycetes
OJJ55948.1-1036612-Aspergillus sydowii CBS 5-Eurotiomycetes
OJI98882.1-1036611-Aspergillus versicolor CB-Eurotiomycetes
OTB15976.1-1001832-Daldinia sp EC12-Sordariomycetes
XP 007798640.1-1287681-Eutypa lata UCREL1-Sordariomycetes
OTA56458.1-1001937-Hypoxylon sp EC38-Sordariomycetes
OTA82740.1-1001938-Hypoxylon sp CO27 5-Sordariomycetes
OAA75951.1-1081108-Cordyceps confragosa RCEF-Sordariomycetes
OAA36148.1-1081107-Cordyceps brongniartii RC-Sordariomycetes
KGQ02914.1-1245745-Beauveria bassiana D1 5-Sordariomycetes
XP 008603200.1-655819-Beauveria bassiana ARSEF-Sordariomycetes
KKK23147.1-138278-Aspergillus ochraceoroseu-Eurotiomycetes
KKK12815.1-308745-Aspergillus rambellii-Eurotiomycetes
KFX52033.1-1077442-Talaromyces marneffei PM1-Eurotiomycetes
KUL81322.1-198730-Talaromyces verruculosus-Eurotiomycetes
KUL83650.1-198730-Talaromyces verruculosus-Eurotiomycetes
OTB09705.1-1001832-Daldinia sp EC12-Sordariomycetes
XP 001239921.2-246410-Coccidioides immitis RS-Eurotiomycetes
EFW19722.1-443226-Coccidioides posadasii st-Eurotiomycetes
XP 003067430.1-222929-Coccidioides posadasii C7-Eurotiomycetes
XP 013331016.1-1408163-Rasamsonia emersonii CBS-Eurotiomycetes
CRG86000.1-28573-Talaromyces islandicus-Eurotiomycetes
XP 002480148.1-441959-Talaromyces stipitatus AT-Eurotiomycetes
KUL85954.1-198730-Talaromyces verruculosus-Eurotiomycetes
GAM39457.1-1472165-Talaromyces cellulolyticu-Eurotiomycetes
OJJ43620.1-1073090-Penicilliopsis zonata CBS-Eurotiomycetes
EYE99324.1-1388766-Aspergillus ruber CBS 135-Eurotiomycetes
OJJ79962.1-1160497-Aspergillus glaucus CBS 5-Eurotiomycetes
ODM14728.1-573508-Aspergillus cristatus-Eurotiomycetes
OJJ33439.1-1073089-Aspergillus wentii DTO 13-Eurotiomycetes
XP 001213574.1-341663-Aspergillus terreus NIH26-Eurotiomycetes
GAA85162.1-1033177-Aspergillus kawachii IFO-Eurotiomycetes
XP 001397938.2-425011-Aspergillus niger CBS 513-Eurotiomycetes
OOG00119.1-602072-Aspergillus carbonarius I-Eurotiomycetes
XP 020058956.1-690307-Aspergillus aculeatus ATC-Eurotiomycetes
KJK64871.1-1403190-Aspergillus parasiticus S-Eurotiomycetes
OOO10410.1-5062-Aspergillus oryzae-Eurotiomycetes
XP 001819693.2-510516-Aspergillus oryzae RIB40-Eurotiomycetes
KOS43211.1-229535-Penicillium nordicum-Eurotiomycetes
XP 002557457.1-500485-Penicillium rubens Wiscon-Eurotiomycetes
OQE12082.1-29845-Penicillium vulpinum-Eurotiomycetes
XP 001272515.1-344612-Aspergillus clavatus NRRL-Eurotiomycetes
XP 001211106.1-341663-Aspergillus terreus NIH26-Eurotiomycetes
OJJ33579.1-1073089-Aspergillus wentii DTO 13-Eurotiomycetes
OQE22944.1-303698-Penicillium steckii-Eurotiomycetes
CEL11526.1-454130-Aspergillus calidoustus-Eurotiomycetes
OJJ08308.1-1036611-Aspergillus versicolor CB-Eurotiomycetes
OGE55730.1-1835702-Penicillium arizonense-Eurotiomycetes
OQD90505.1-416450-Penicillium antarcticum-Eurotiomycetes
OOF92076.1-602072-Aspergillus carbonarius I-Eurotiomycetes
OJJ68031.1-767769-Aspergillus brasiliensis-Eurotiomycetes
CAK38339.1-5061-Aspergillus niger-Eurotiomycetes
XP 001390458.2-425011-Aspergillus niger CBS 513-Eurotiomycetes
GAA90921.1-1033177-Aspergillus kawachii IFO-Eurotiomycetes
OJI89131.1-767770-Aspergillus tubingensis C-Eurotiomycetes
GAQ47076.1-5061-Aspergillus niger-Eurotiomycetes
OJJ78945.1-1160497-Aspergillus glaucus CBS 5-Eurotiomycetes
EYE91325.1-1388766-Aspergillus ruber CBS 135-Eurotiomycetes
OOF97174.1-602072-Aspergillus carbonarius I-Eurotiomycetes
OJJ75164.1-767769-Aspergillus brasiliensis-Eurotiomycetes
XP 001388700.1-425011-Aspergillus niger CBS 513-Eurotiomycetes
EHA26267.1-380704-Aspergillus niger ATCC 10-Eurotiomycetes
GAQ33368.1-5061-Aspergillus niger-Eurotiomycetes
GAA88775.1-1033177-Aspergillus kawachii IFO-Eurotiomycetes
OJZ92735.1-1137211-Aspergillus luchuensis CB-Eurotiomycetes
CEL03626.1-454130-Aspergillus calidoustus-Eurotiomycetes
OQE04218.1-60172-Penicillium solitum-Eurotiomycetes
KOS43880.1-229535-Penicillium nordicum-Eurotiomycetes
CRL18065.1-5075-Penicillium camemberti-Eurotiomycetes
OQD72324.1-60169-Penicillium polonicum-Eurotiomycetes
XP 016597175.1-27334-Penicillium expansum-Eurotiomycetes
CDM34354.1-1365484-Penicillium roqueforti FM-Eurotiomycetes
KXG48446.1-5078-Penicillium griseofulvum-Eurotiomycetes
OQE47057.1-36646-Penicillium coprophilum-Eurotiomycetes
OQE02745.1-29845-Penicillium vulpinum-Eurotiomycetes
XP 002558089.1-500485-Penicillium rubens Wiscon-Eurotiomycetes
OQE29095.1-254877-Penicillium flavigenum-Eurotiomycetes
OQE95301.1-60175-Penicillium nalgiovense-Eurotiomycetes

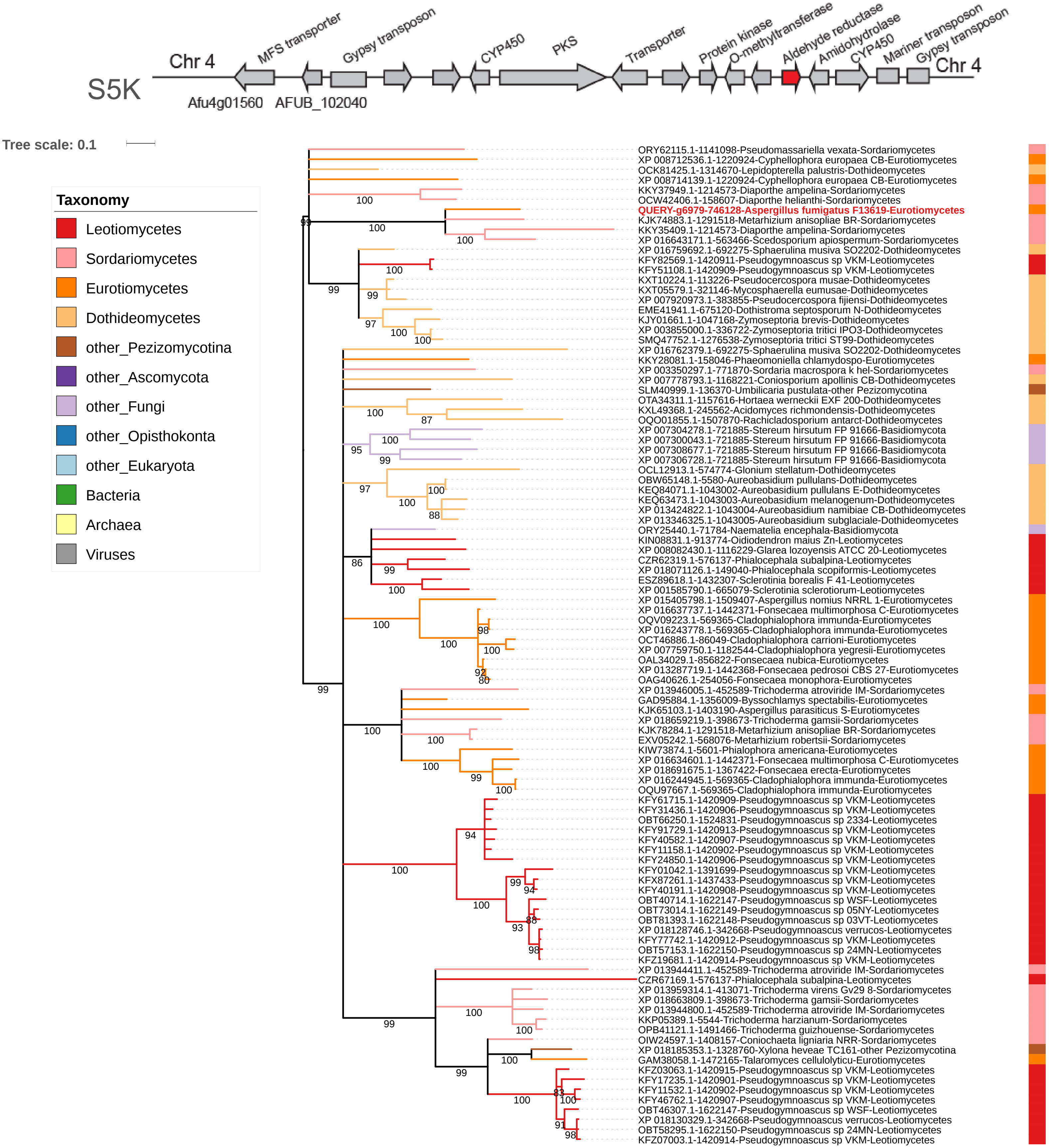
S5K
Chr 4
MFS transporter
Gypsy transposon
CYP450
PKS
Transporter
Protein kinase
O-methyltransferase
Aldehyde reductase
Amidohydrolase
CYP450
Mariner transposon
Gypsy transposon
Chr 4
Afu4g01560
AFUB_102040
Tree scale: 0.1
Taxonomy
Leotiomycetes
Sordariomycetes
Eurotiomycetes
Dothideomycetes
other_Pezizomycotina
other_Ascomycota
other_Fungi
other_Opisthokonta
other_Eukaryota
Bacteria
Archaea
Viruses
ORY62115.1-1141098-Pseudomassariella vexata-Sordariomycetes
XP 008712536.1-1220924-Cyphellophora europaea CB-Eurotiomycetes
OCK81425.1-1314670-Lepidopterella palustris-Dothideomycetes
XP 008714139.1-1220924-Cyphellophora europaea CB-Eurotiomycetes
KKY37949.1-1214573-Diaporthe ampelina-Sordariomycetes
OCW42406.1-158607-Diaporthe helianthi-Sordariomycetes
QUERY-g6979-746128-Aspergillus fumigatus F13619-Eurotiomycetes
KJK74883.1-1291518-Metarhizium anisopliae BR-Sordariomycetes
KKY35409.1-1214573-Diaporthe ampelina-Sordariomycetes
XP 016643171.1-563466-Scedosporium apiospermum-Sordariomycetes
XP 016759692.1-692275-Sphaerulina musiva SO2202-Dothideomycetes
KFY82569.1-1420911-Pseudogymnoascus sp VKM-Leotiomycetes
KFY51108.1-1420909-Pseudogymnoascus sp VKM-Leotiomycetes
KXT10224.1-113226-Pseudocercospora musae-Dothideomycetes
KXT05579.1-321146-Mycosphaerella eumusae-Dothideomycetes
XP 007920973.1-383855-Pseudocercospora fijiensis-Dothideomycetes
EME41941.1-675120-Dothistroma septosporum N-Dothideomycetes
KJY01661.1-1047168-Zymoseptoria brevis-Dothideomycetes
XP 003855000.1-336722-Zymoseptoria tritici IPO3-Dothideomycetes
SMQ47752.1-1276538-Zymoseptoria tritici ST99-Dothideomycetes
XP 016762379.1-692275-Sphaerulina musiva SO2202-Dothideomycetes
KKY28081.1-1316127-Phaeomoniella chlamydospo-Eurotiomycetes
XP 003350297.1-771870-Sordaria macrospora k hel-Sordariomycetes
XP 007778793.1-1168221-Coniosporium apollinis CB-Dothideomycetes
SLM40999.1-136370-Umbilicaria pustulata-other Pezizomycotina
OTA34311.1-1157616-Hortaea werneckii EXF 200-Dothideomycetes
KXL49368.1-245562-Acidomyces richmondensis-Dothideomycetes
OQO01855.1-1507870-Rachicladosporium antarct-Dothideomycetes
XP 007304278.1-721885-Stereum hirsutum FP 91666-Basidiomycota
XP 007300043.1-721885-Stereum hirsutum FP 91666-Basidiomycota
XP 007308677.1-721885-Stereum hirsutum FP 91666-Basidiomycota
XP 007306728.1-721885-Stereum hirsutum FP 91666-Basidiomycota
OCL12913.1-574774-Glonium stellatum-Dothideomycetes
OBW65148.1-5580-Aureobasidium pullulans-Dothideomycetes
KEQ84071.1-1043002-Aureobasidium pullulans E-Dothideomycetes
KEQ63473.1-1043003-Aureobasidium melanogenum-Dothideomycetes
XP 013424822.1-1043004-Aureobasidium namibiae CB-Dothideomycetes
XP 013346325.1-1043005-Aureobasidium subglaciale-Dothideomycetes
ORY25440.1-71784-Naematelia encephala-Basidiomycota
KIN08831.1-913774-Oidiodendron maius Zn-Leotiomycetes
XP 008082430.1-1116229-Glarea lozoyensis ATCC 20-Leotiomycetes
CZR62319.1-576137-Phialocephala subalpina-Leotiomycetes
XP 018071126.1-149040-Phialocephala scopiformis-Leotiomycetes
ESZ89618.1-1432307-Sclerotinia borealis F 41-Leotiomycetes
XP 001585790.1-665079-Sclerotinia sclerotiorum-Leotiomycetes
XP 015405798.1-1509407-Aspergillus nomius NRRL 1-Eurotiomycetes
XP 016637737.1-1442371-Fonsecaea multimorphosa C-Eurotiomycetes
OQV09223.1-569365-Cladophialophora immunda-Eurotiomycetes
XP 016243778.1-569365-Cladophialophora immunda-Eurotiomycetes
OCT46886.1-86049-Cladophialophora carrioni-Eurotiomycetes
XP 007759750.1-1182544-Cladophialophora yegresii-Eurotiomycetes
OAL34029.1-856822-Fonsecaea nubica-Eurotiomycetes
XP 013287719.1-1442368-Fonsecaea pedrosoi CBS 27-Eurotiomycetes
OAG40626.1-254056-Fonsecaea monophora-Eurotiomycetes
XP 013946005.1-452589-Trichoderma atroviride IM-Sordariomycetes
GAD95884.1-1356009-Byssochlamys spectabilis-Eurotiomycetes
KJK65103.1-1403190-Aspergillus parasiticus S-Eurotiomycetes
XP 018659219.1-398673-Trichoderma gamsii-Sordariomycetes
KJK78284.1-1291518-Metarhizium anisopliae BR-Sordariomycetes
EXV05242.1-568076-Metarhizium robertsii-Sordariomycetes
KIW73874.1-5601-Phialophora americana-Eurotiomycetes
XP 016634601.1-1442371-Fonsecaea multimorphosa C-Eurotiomycetes
XP 018691675.1-1367422-Fonsecaea erecta-Eurotiomycetes
XP 016244945.1-569365-Cladophialophora immunda-Eurotiomycetes
OQU97667.1-569365-Cladophialophora immunda-Eurotiomycetes
KFY61715.1-1420909-Pseudogymnoascus sp VKM-Leotiomycetes
KFY31436.1-1420906-Pseudogymnoascus sp VKM-Leotiomycetes
OBT66250.1-1524831-Pseudogymnoascus sp 2334-Leotiomycetes
KFY91729.1-1420913-Pseudogymnoascus sp VKM-Leotiomycetes
KFY40582.1-1420907-Pseudogymnoascus sp VKM-Leotiomycetes
KFY11158.1-1420902-Pseudogymnoascus sp VKM-Leotiomycetes
KFY24850.1-1420906-Pseudogymnoascus sp VKM-Leotiomycetes
KFY01042.1-1391699-Pseudogymnoascus sp VKM-Leotiomycetes
KFX87261.1-1437433-Pseudogymnoascus sp VKM-Leotiomycetes
KFY40191.1-1420908-Pseudogymnoascus sp VKM-Leotiomycetes
OBT40714.1-1622147-Pseudogymnoascus sp WSF-Leotiomycetes
OBT73014.1-1622149-Pseudogymnoascus sp 05NY-Leotiomycetes
OBT81393.1-1622148-Pseudogymnoascus sp 03VT-Leotiomycetes
XP 018128746.1-342668-Pseudogymnoascus verrucos-Leotiomycetes
KFY77742.1-1420912-Pseudogymnoascus sp VKM-Leotiomycetes
OBT57153.1-1622150-Pseudogymnoascus sp 24MN-Leotiomycetes
KFZ19681.1-1420914-Pseudogymnoascus sp VKM-Leotiomycetes
XP 013944411.1-452589-Trichoderma atroviride IM-Sordariomycetes
CZR67169.1-576137-Phialocephala subalpina-Leotiomycetes
XP 013959314.1-413071-Trichoderma virens Gv29 8-Sordariomycetes
XP 018663809.1-398673-Trichoderma gamsii-Sordariomycetes
XP 013944800.1-452589-Trichoderma atroviride IM-Sordariomycetes
KKP05389.1-5544-Trichoderma harzianum-Sordariomycetes
OPB41121.1-1491466-Trichoderma guizhouense-Sordariomycetes
OIW24597.1-1408157-Coniochaeta ligniaria NRR-Sordariomycetes
XP 018185353.1-1328760-Xylona heveae TC161-other Pezizomycotina
GAM38058.1-1472165-Talaromyces cellulolyticu-Eurotiomycetes
KFZ03063.1-1420915-Pseudogymnoascus sp VKM-Leotiomycetes
KFY17235.1-1420901-Pseudogymnoascus sp VKM-Leotiomycetes
KFY11532.1-1420902-Pseudogymnoascus sp VKM-Leotiomycetes
KFY46762.1-1420907-Pseudogymnoascus sp VKM-Leotiomycetes
OBT46307.1-1622147-Pseudogymnoascus sp WSF-Leotiomycetes
XP 018130329.1-342668-Pseudogymnoascus verrucos-Leotiomycetes
OBT58295.1-1622150-Pseudogymnoascus sp 24MN-Leotiomycetes
KFZ07003.1-1420914-Pseudogymnoascus sp VKM-Leotiomycetes

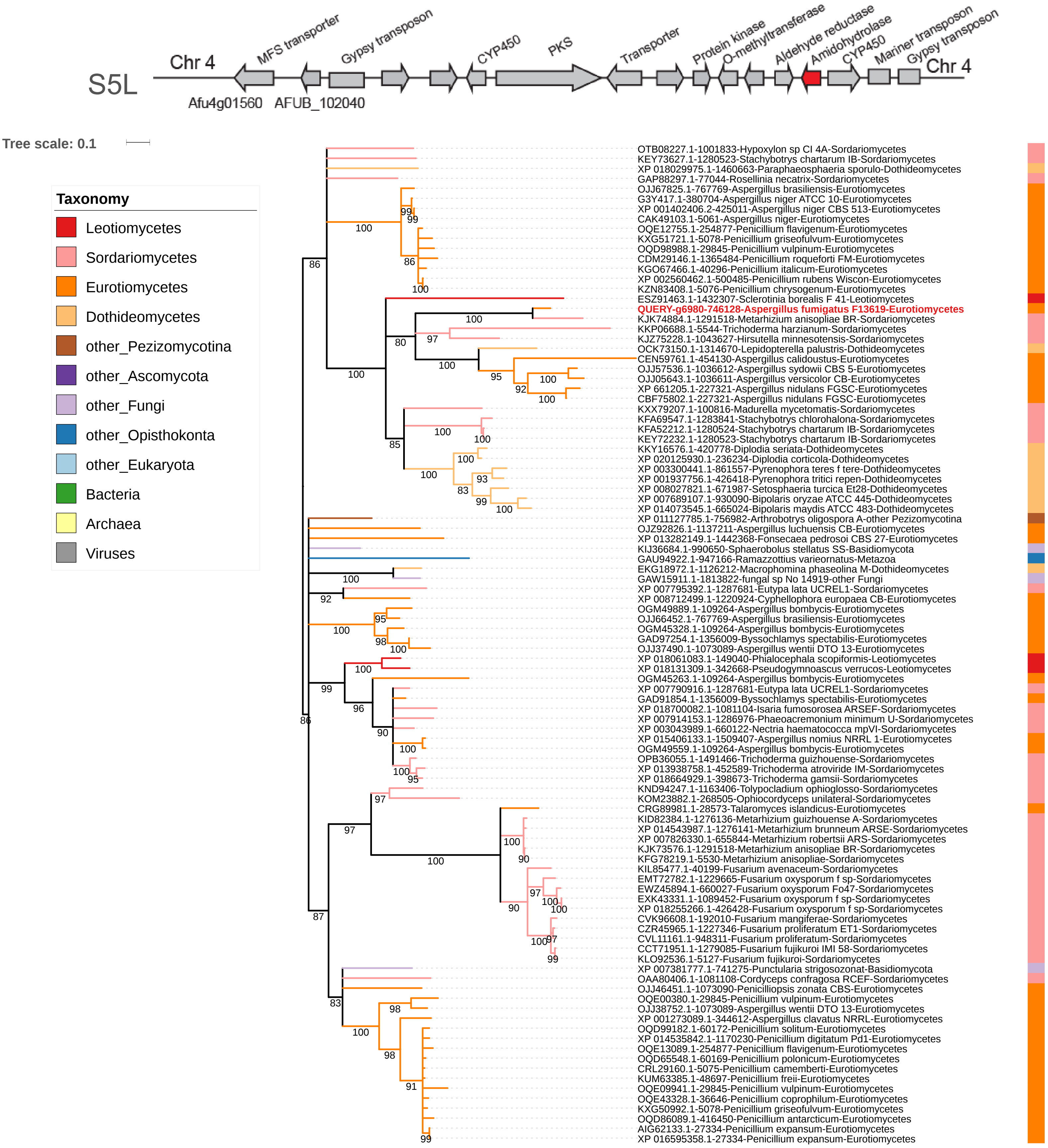

S5L
Chr 4
MFS transporter
Gypsy transposon
CYP450
PKS
Transporter
Protein kinase
O-methyltransferase
Aldehyde reductase
Amidohydrolase
CYP450
Mariner transposon
Gypsy transposon
Chr 4
Afu4g01560
AFUB_102040
Tree scale: 0.1
Taxonomy
Leotiomycetes
Sordariomycetes
Eurotiomycetes
Dothideomycetes
other_Pezizomycotina
other_Ascomycota
other_Fungi
other_Opisthokonta
other_Eukaryota
Bacteria
Archaea
Viruses
OTB08227.1-1001833-Hypoxylon sp CI 4A-Sordariomycetes
KEY73627.1-1280523-Stachybotrys chartarum IB-Sordariomycetes
XP 018029975.1-1460663-Paraphaeosphaeria sporulo-Dothideomycetes
GAP88297.1-77044-Rosellinia necatrix-Sordariomycetes
OJJ67825.1-767769-Aspergillus brasiliensis-Eurotiomycetes
G3Y417.1-380704-Aspergillus niger ATCC 10-Eurotiomycetes
XP 001402406.2-425011-Aspergillus niger CBS 513-Eurotiomycetes
CAK49103.1-5061-Aspergillus niger-Eurotiomycetes
OQE12755.1-254877-Penicillium flavigenum-Eurotiomycetes
KXG51721.1-5078-Penicillium griseofulvum-Eurotiomycetes
OQD98988.1-29845-Penicillium vulpinum-Eurotiomycetes
CDM29146.1-1365484-Penicillium roqueforti FM-Eurotiomycetes
KGO67466.1-40296-Penicillium italicum-Eurotiomycetes
XP 002560462.1-500485-Penicillium rubens Wiscon-Eurotiomycetes
KZN83408.1-5076-Penicillium chrysogenum-Eurotiomycetes
ESZ91463.1-1432307-Sclerotinia borealis F 41-Leotiomycetes
QUERY-g6980-746128-Aspergillus fumigatus F13619-Eurotiomycetes
KJK74884.1-1291518-Metarhizium anisopliae BR-Sordariomycetes
KKP06688.1-5544-Trichoderma harzianum-Sordariomycetes
KJZ75228.1-1043627-Hirsutella minnesotensis-Sordariomycetes
OCK73150.1-1314670-Lepidopterella palustris-Dothideomycetes
CEN59761.1-454130-Aspergillus calidoustus-Eurotiomycetes
OJJ57536.1-1036612-Aspergillus sydowii CBS 5-Eurotiomycetes
OJJ05643.1-1036611-Aspergillus versicolor CB-Eurotiomycetes
XP 661205.1-227321-Aspergillus nidulans FGSC-Eurotiomycetes
CBF75802.1-227321-Aspergillus nidulans FGSC-Eurotiomycetes
KXX79207.1-100816-Madurella mycetomatis-Sordariomycetes
KFA69547.1-1283841-Stachybotrys chlorohalona-Sordariomycetes
KFA52212.1-1280524-Stachybotrys chartarum IB-Sordariomycetes
KEY72232.1-1280523-Stachybotrys chartarum IB-Sordariomycetes
KKY16576.1-420778-Diplodia seriata-Dothideomycetes
XP 020125930.1-236234-Diplodia corticola-Dothideomycetes
XP 003300441.1-861557-Pyrenophora teres f tere-Dothideomycetes
XP 001937756.1-426418-Pyrenophora tritici repen-Dothideomycetes
XP 008027821.1-671987-Setosphaeria turcica Et28-Dothideomycetes
XP 007689107.1-930090-Bipolaris oryzae ATCC 445-Dothideomycetes
XP 014073545.1-665024-Bipolaris maydis ATCC 483-Dothideomycetes
XP 011127785.1-756982-Arthrobotrys oligospora A-other Pezizomycotina
OJZ92826.1-1137211-Aspergillus luchuensis CB-Eurotiomycetes
XP 013282149.1-1442368-Fonsecaea pedrosoi CBS 27-Eurotiomycetes
KIJ36684.1-990650-Sphaerobolus stellatus SS-Basidiomycota
GAU94922.1-947166-Ramazzottius varieornatus-Metazoa
EKG18972.1-1126212-Macrophomina phaseolina M-Dothideomycetes
GAW15911.1-1813822-fungal sp No 14919-other Fungi
XP 007795392.1-1287681-Eutypa lata UCREL1-Sordariomycetes
XP 008712499.1-1220924-Cyphellophora europaea CB-Eurotiomycetes
OGM49889.1-109264-Aspergillus bombycis-Eurotiomycetes
OJJ66452.1-767769-Aspergillus brasiliensis-Eurotiomycetes
OGM45328.1-109264-Aspergillus bombycis-Eurotiomycetes
GAD97254.1-1356009-Byssochlamys spectabilis-Eurotiomycetes
OJJ37490.1-1073089-Aspergillus wentii DTO 13-Eurotiomycetes
XP 018061083.1-149040-Phialocephala scopiformis-Leotiomycetes
XP 018131309.1-342668-Pseudogymnoascus verrucos-Leotiomycetes
OGM45263.1-109264-Aspergillus bombycis-Eurotiomycetes
XP 007790916.1-1287681-Eutypa lata UCREL1-Sordariomycetes
GAD91854.1-1356009-Byssochlamys spectabilis-Eurotiomycetes
XP 018700082.1-1081104-Isaria fumosorosea ARSEF-Sordariomycetes
XP 007914153.1-1286976-Phaeoacremonium minimum U-Sordariomycetes
XP 003043989.1-660122-Nectria haematococca mpVI-Sordariomycetes
XP 015406133.1-1509407-Aspergillus nomius NRRL 1-Eurotiomycetes
OGM49559.1-109264-Aspergillus bombycis-Eurotiomycetes
OPB36055.1-1491466-Trichoderma guizhouense-Sordariomycetes
XP 013938758.1-452589-Trichoderma atroviride IM-Sordariomycetes
XP 018664929.1-398673-Trichoderma gamsii-Sordariomycetes
KND94247.1-1163406-Tolypocladium ophioglosso-Sordariomycetes
KOM23882.1-268505-Ophiocordyceps unilateral-Sordariomycetes
CRG89981.1-28573-Talaromyces islandicus-Eurotiomycetes
KID82384.1-1276136-Metarhizium guizhouense A-Sordariomycetes
XP 014543987.1-1276141-Metarhizium brunneum ARSE-Sordariomycetes
XP 007826330.1-655844-Metarhizium robertsii ARS-Sordariomycetes
KJK73576.1-1291518-Metarhizium anisopliae BR-Sordariomycetes
KFG78219.1-5530-Metarhizium anisopliae-Sordariomycetes
KIL85477.1-40199-Fusarium avenaceum-Sordariomycetes
EMT72782.1-1229665-Fusarium oxysporum f sp-Sordariomycetes
EWZ45894.1-660027-Fusarium oxysporum Fo47-Sordariomycetes
EXK43331.1-1089452-Fusarium oxysporum f sp-Sordariomycetes
XP 018255266.1-426428-Fusarium oxysporum f sp-Sordariomycetes
CVK96608.1-192010-Fusarium mangiferae-Sordariomycetes
CZR45965.1-1227346-Fusarium proliferatum ET1-Sordariomycetes
CVL11161.1-948311-Fusarium proliferatum-Sordariomycetes
CCT71951.1-1279085-Fusarium fujikuroi IMI 58-Sordariomycetes
KLO92536.1-5127-Fusarium fujikuroi-Sordariomycetes
XP 007381777.1-741275-Punctularia strigosozonat-Basidiomycota
OAA80406.1-1081108-Cordyceps confragosa RCEF-Sordariomycetes
OJJ46451.1-1073090-Penicilliopsis zonata CBS-Eurotiomycetes
OQE00380.1-29845-Penicillium vulpinum-Eurotiomycetes
OJJ38752.1-1073089-Aspergillus wentii DTO 13-Eurotiomycetes
XP 001273089.1-344612-Aspergillus clavatus NRRL-Eurotiomycetes
OQD99182.1-60172-Penicillium solitum-Eurotiomycetes
XP 014535842.1-1170230-Penicillium digitatum Pd1-Eurotiomycetes
OQE13089.1-254877-Penicillium flavigenum-Eurotiomycetes
OQD65548.1-60169-Penicillium polonicum-Eurotiomycetes
CRL29160.1-5075-Penicillium camemberti-Eurotiomycetes
KUM63385.1-48697-Penicillium freii-Eurotiomycetes
OQE09941.1-29845-Penicillium vulpinum-Eurotiomycetes
OQE43328.1-36646-Penicillium coprophilum-Eurotiomycetes
KXG50992.1-5078-Penicillium griseofulvum-Eurotiomycetes
OQD86089.1-416450-Penicillium antarcticum-Eurotiomycetes
AIG62133.1-27334-Penicillium expansum-Eurotiomycetes
XP 016595358.1-27334-Penicillium expansum-Eurotiomycetes

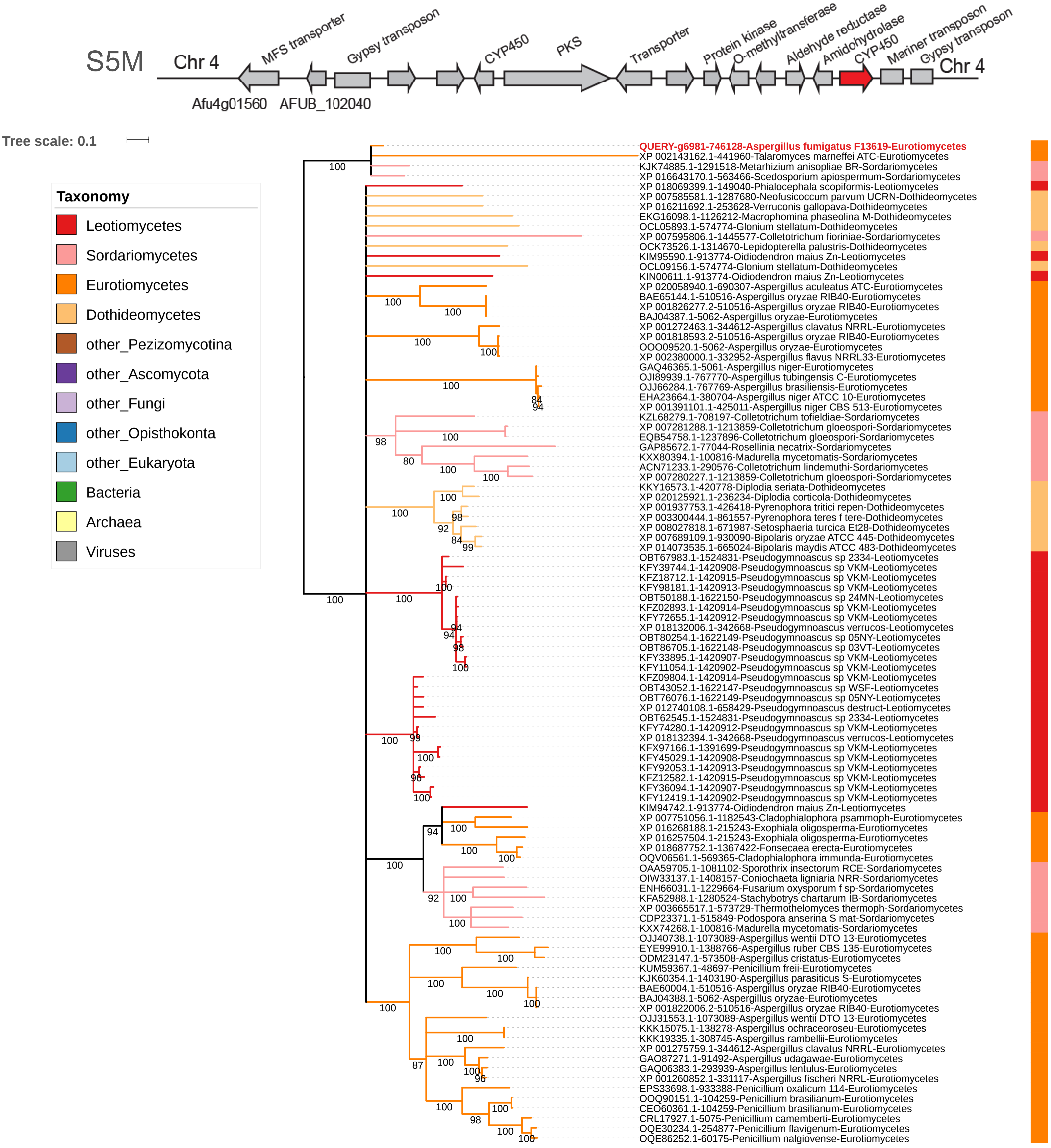
S5M
Chr 4
MFS transporter
Gypsy transposon
CYP450
PKS
Transporter
Protein kinase
O-methyltransferase
Aldehyde reductase
Amidohydrolase
CYP450
Mariner transposon
Gypsy transposon
Chr 4
Afu4g01560
AFUB_102040
Tree scale: 0.1
Taxonomy
Leotiomycetes
Sordariomycetes
Eurotiomycetes
Dothideomycetes
other_Pezizomycotina
other_Ascomycota
other_Fungi
other_Opisthokonta
other_Eukaryota
Bacteria
Archaea
Viruses
QUERY-g6981-746128-Aspergillus fumigatus F13619-Eurotiomycetes
XP 002143162.1-441960-Talaromyces marneffei ATC-Eurotiomycetes
KJK74885.1-1291518-Metarhizium anisopliae BR-Sordariomycetes
XP 016643170.1-563466-Scedosporium apiospermum-Sordariomycetes
XP 018069399.1-149040-Phialocephala scopiformis-Leotiomycetes
XP 007585581.1-1287680-Neofusicoccum parvum UCRN-Dothideomycetes
XP 016211692.1-253628-Verruconis gallopava-Dothideomycetes
EKG16098.1-1126212-Macrophomina phaseolina M-Dothideomycetes
OCL05893.1-574774-Glonium stellatum-Dothideomycetes
XP 007595806.1-1445577-Colletotrichum fioriniae-Sordariomycetes
OCK73526.1-1314670-Lepidopterella palustris-Dothideomycetes
KIM95590.1-913774-Oidiodendron maius Zn-Leotiomycetes
OCL09156.1-574774-Glonium stellatum-Dothideomycetes
KIN00611.1-913774-Oidiodendron maius Zn-Leotiomycetes
XP 020058940.1-690307-Aspergillus aculeatus ATC-Eurotiomycetes
BAE65144.1-510516-Aspergillus oryzae RIB40-Eurotiomycetes
XP 001826277.2-510516-Aspergillus oryzae RIB40-Eurotiomycetes
BAJ04387.1-5062-Aspergillus oryzae-Eurotiomycetes
XP 001272463.1-344612-Aspergillus clavatus NRRL-Eurotiomycetes
XP 001818593.2-510516-Aspergillus oryzae RIB40-Eurotiomycetes
OOO09520.1-5062-Aspergillus oryzae-Eurotiomycetes
XP 002380000.1-332952-Aspergillus flavus NRRL33-Eurotiomycetes
GAQ46365.1-5061-Aspergillus niger-Eurotiomycetes
OJI89939.1-767770-Aspergillus tubingensis C-Eurotiomycetes
OJJ66284.1-767769-Aspergillus brasiliensis-Eurotiomycetes
EHA23664.1-380704-Aspergillus niger ATCC 10-Eurotiomycetes
XP 001391101.1-425011-Aspergillus niger CBS 513-Eurotiomycetes
KZL68279.1-708197-Colletotrichum tofieldiae-Sordariomycetes
XP 007281288.1-1213859-Colletotrichum gloeospori-Sordariomycetes
EQB54758.1-1237896-Colletotrichum gloeospori-Sordariomycetes
GAP85672.1-77044-Rosellinia necatrix-Sordariomycetes
KXX80394.1-100816-Madurella mycetomatis-Sordariomycetes
ACN71233.1-290576-Colletotrichum lindemuthi-Sordariomycetes
XP 007280227.1-1213859-Colletotrichum gloeospori-Sordariomycetes
KKY16573.1-420778-Diplodia seriata-Dothideomycetes
XP 020125921.1-236234-Diplodia corticola-Dothideomycetes
XP 001937753.1-426418-Pyrenophora tritici repen-Dothideomycetes
XP 003300444.1-861557-Pyrenophora teres f tere-Dothideomycetes
XP 008027818.1-671987-Setosphaeria turcica Et28-Dothideomycetes
XP 007689109.1-930090-Bipolaris oryzae ATCC 445-Dothideomycetes
XP 014073535.1-665024-Bipolaris maydis ATCC 483-Dothideomycetes
OBT67983.1-1524831-Pseudogymnoascus sp 2334-Leotiomycetes
KFY39744.1-1420908-Pseudogymnoascus sp VKM-Leotiomycetes
KFZ18712.1-1420915-Pseudogymnoascus sp VKM-Leotiomycetes
KFY98181.1-1420913-Pseudogymnoascus sp VKM-Leotiomycetes
OBT50188.1-1622150-Pseudogymnoascus sp 24MN-Leotiomycetes
KFZ02893.1-1420914-Pseudogymnoascus sp VKM-Leotiomycetes
KFY72655.1-1420912-Pseudogymnoascus sp VKM-Leotiomycetes
XP 018132006.1-342668-Pseudogymnoascus verrucos-Leotiomycetes
OBT80254.1-1622149-Pseudogymnoascus sp 05NY-Leotiomycetes
OBT86705.1-1622148-Pseudogymnoascus sp 03VT-Leotiomycetes
KFY33895.1-1420907-Pseudogymnoascus sp VKM-Leotiomycetes
KFY11054.1-1420902-Pseudogymnoascus sp VKM-Leotiomycetes
KFZ09804.1-1420914-Pseudogymnoascus sp VKM-Leotiomycetes
OBT43052.1-1622147-Pseudogymnoascus sp WSF-Leotiomycetes
OBT76076.1-1622149-Pseudogymnoascus sp 05NY-Leotiomycetes
XP 012740108.1-658429-Pseudogymnoascus destruct-Leotiomycetes
OBT62545.1-1524831-Pseudogymnoascus sp 2334-Leotiomycetes
KFY74280.1-1420912-Pseudogymnoascus sp VKM-Leotiomycetes
XP 018132394.1-342668-Pseudogymnoascus verrucos-Leotiomycetes
KFX97166.1-1391699-Pseudogymnoascus sp VKM-Leotiomycetes
KFY45029.1-1420908-Pseudogymnoascus sp VKM-Leotiomycetes
KFY92053.1-1420913-Pseudogymnoascus sp VKM-Leotiomycetes
KFZ12582.1-1420915-Pseudogymnoascus sp VKM-Leotiomycetes
KFY36094.1-1420907-Pseudogymnoascus sp VKM-Leotiomycetes
KFY12419.1-1420902-Pseudogymnoascus sp VKM-Leotiomycetes
KIM94742.1-913774-Oidiodendron maius Zn-Leotiomycetes
XP 007751056.1-1182543-Cladophialophora psammoph-Eurotiomycetes
XP 016268188.1-215243-Exophiala oligosperma-Eurotiomycetes
XP 016257504.1-215243-Exophiala oligosperma-Eurotiomycetes
XP 018687752.1-1367422-Fonsecaea erecta-Eurotiomycetes
OQV06561.1-569365-Cladophialophora immunda-Eurotiomycetes
OAA59705.1-1081102-Sporothrix insectorum RCE-Sordariomycetes
OIW33137.1-1408157-Coniochaeta ligniaria NRR-Sordariomycetes
ENH66031.1-1229664-Fusarium oxysporum f sp-Sordariomycetes
KFA52988.1-1280524-Stachybotrys chartarum IB-Sordariomycetes
XP 003665517.1-573729-Thermothelomyces thermoph-Sordariomycetes
CDP23371.1-515849-Podospora anserina S mat-Sordariomycetes
KXX74268.1-100816-Madurella mycetomatis-Sordariomycetes
OJJ40738.1-1073089-Aspergillus wentii DTO 13-Eurotiomycetes
EYE99910.1-1388766-Aspergillus ruber CBS 135-Eurotiomycetes
ODM23147.1-573508-Aspergillus cristatus-Eurotiomycetes
KUM59367.1-48697-Penicillium freii-Eurotiomycetes
KJK60354.1-1403190-Aspergillus parasiticus S-Eurotiomycetes
BAE60004.1-510516-Aspergillus oryzae RIB40-Eurotiomycetes
BAJ04388.1-5062-Aspergillus oryzae-Eurotiomycetes
XP 001822006.2-510516-Aspergillus oryzae RIB40-Eurotiomycetes
OJJ31553.1-1073089-Aspergillus wentii DTO 13-Eurotiomycetes
KKK15075.1-138278-Aspergillus ochraceoroseu-Eurotiomycetes
KKK19335.1-308745-Aspergillus rambellii-Eurotiomycetes
XP 001275759.1-344612-Aspergillus clavatus NRRL-Eurotiomycetes
GAO87271.1-91492-Aspergillus udagawae-Eurotiomycetes
GAQ06383.1-293939-Aspergillus lentulus-Eurotiomycetes
XP 001260852.1-331117-Aspergillus fischeri NRRL-Eurotiomycetes
EPS33698.1-933388-Penicillium oxalicum 114-Eurotiomycetes
OOQ90151.1-104259-Penicillium brasilianum-Eurotiomycetes
CEO60361.1-104259-Penicillium brasilianum-Eurotiomycetes
CRL17927.1-5075-Penicillium camemberti-Eurotiomycetes
OQE30234.1-254877-Penicillium flavigenum-Eurotiomycetes
OQE86252.1-60175-Penicillium nalgiovense-Eurotiomycetes
