# Supplementary material for: Drivers of genetic diversity in secondary metabolic gene clusters within a fungal species
Source: PLoS Biol. 2017 Nov 17;15(11):e2003583. doi: 10.1371/journal.pbio.2003583 (PMC5711037; doi:10.1371/journal.pbio.2003583)
Supplement: S9 Fig — These phylogenies are consistent with horizontal transfer between Eurotiomycete, Dothidiomycete, Leotiomycete, and Sordariomycete fungi. SM, secondary metabolite. (PDF) [file pbio.2003583.s009.pdf]

S9A

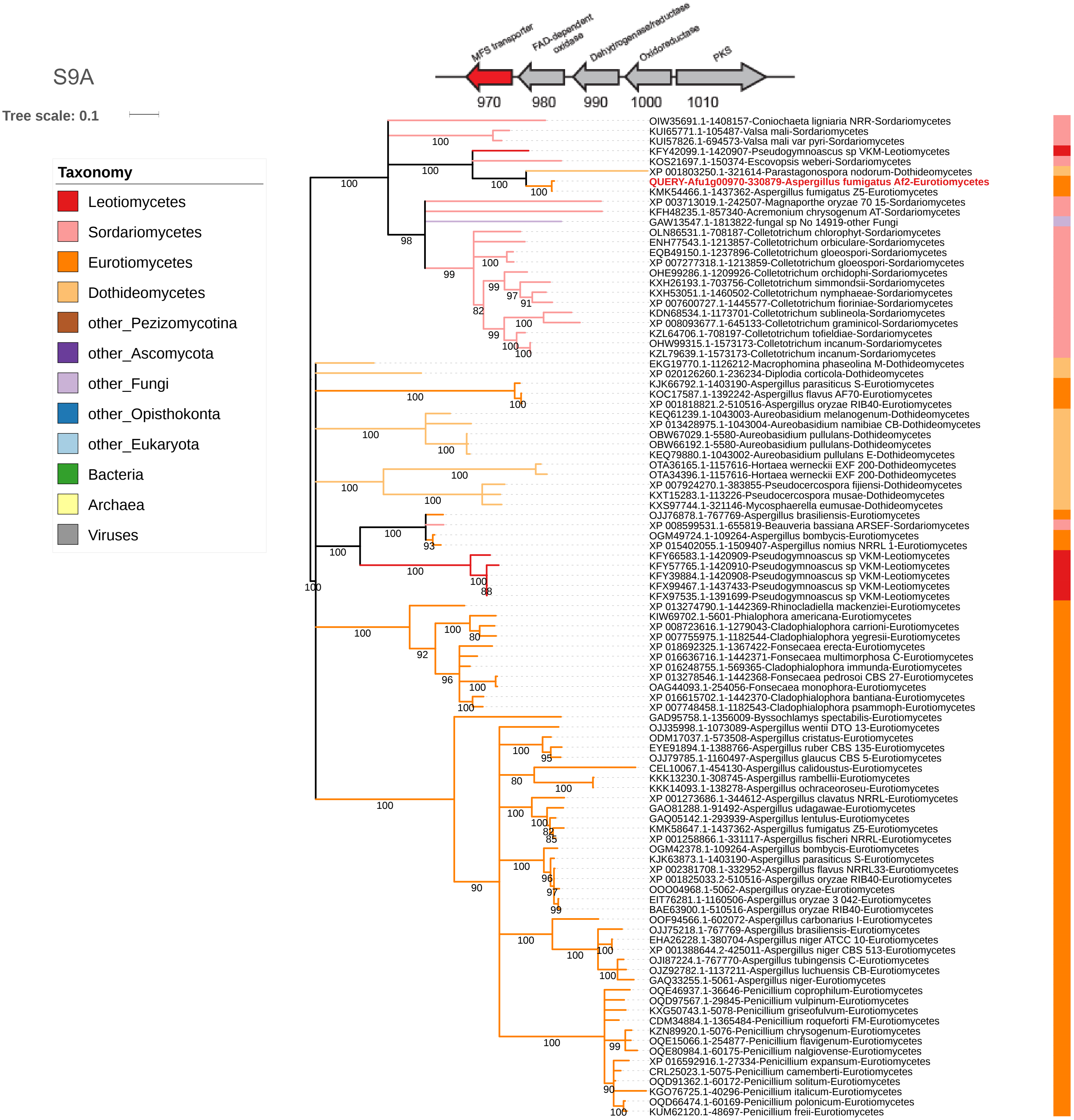

MFS transporter
FAD-dependent oxidase
Dehydrogenase/reductase
Oxidoreductase
PKS
970
980
990
1000
1010
Tree scale: 0.1
Taxonomy
Leotiomycetes
Sordariomycetes
Eurotiomycetes
Dothideomycetes
other_Pezizomycotina
other_Ascomycota
other_Fungi
other_Opisthokonta
other_Eukaryota
Bacteria
Archaea
Viruses
OIW35691.1-1408157-Coniochaeta ligniaria NRR-Sordariomycetes
KUI65771.1-105487-Valsa mali-Sordariomycetes
KUI57826.1-694573-Valsa mali var pyri-Sordariomycetes
KFY42099.1-1420907-Pseudogymnoascus sp VKM-Leotiomycetes
KOS21697.1-150374-Escovopsis weberi-Sordariomycetes
XP 001803250.1-321614-Parastagonospora nodorum-Dothideomycetes
QUERY-Afu1g00970-330879-Aspergillus fumigatus Af2-Eurotiomycetes
KMK54466.1-1437362-Aspergillus fumigatus Z5-Eurotiomycetes
XP 003713019.1-242507-Magnaporthe oryzae 70 15-Sordariomycetes
KFH48235.1-857340-Acremonium chrysogenum AT-Sordariomycetes
GAW13547.1-1813822-fungal sp No 14919-other Fungi
OLN86531.1-708187-Colletotrichum chlorophyt-Sordariomycetes
ENH77543.1-1213857-Colletotrichum orbiculare-Sordariomycetes
EQB49150.1-1237896-Colletotrichum gloeospori-Sordariomycetes
XP 007277318.1-1213859-Colletotrichum gloeospori-Sordariomycetes
OHE99286.1-1209926-Colletotrichum orchidophi-Sordariomycetes
KXH26193.1-703756-Colletotrichum simmondsii-Sordariomycetes
KXH53051.1-1460502-Colletotrichum nymphaeae-Sordariomycetes
XP 007600727.1-1445577-Colletotrichum fioriniae-Sordariomycetes
KDN68534.1-1173701-Colletotrichum sublineola-Sordariomycetes
XP 008093677.1-645133-Colletotrichum graminicol-Sordariomycetes
KZL64706.1-708197-Colletotrichum tofieldiae-Sordariomycetes
OHW99315.1-1573173-Colletotrichum incanum-Sordariomycetes
KZL79639.1-1573173-Colletotrichum incanum-Sordariomycetes
EKG19770.1-1126212-Macrophomina phaseolina M-Dothideomycetes
XP 020126260.1-236234-Diplodia corticola-Dothideomycetes
KJK66792.1-1403190-Aspergillus parasiticus S-Eurotiomycetes
KOC17587.1-1392242-Aspergillus flavus AF70-Eurotiomycetes
XP 001818821.2-510516-Aspergillus oryzae RIB40-Eurotiomycetes
KEQ61239.1-1043003-Aureobasidium melanogenum-Dothideomycetes
XP 013428975.1-1043004-Aureobasidium namibiae CB-Dothideomycetes
OBW67029.1-5580-Aureobasidium pullulans-Dothideomycetes
OBW66192.1-5580-Aureobasidium pullulans-Dothideomycetes
KEQ79880.1-1043002-Aureobasidium pullulans E-Dothideomycetes
OTA36165.1-1157616-Hortaea werneckii EXF 200-Dothideomycetes
OTA34396.1-1157616-Hortaea werneckii EXF 200-Dothideomycetes
XP 007924270.1-383855-Pseudocercospora fijiensi-Dothideomycetes
KXT15283.1-113226-Pseudocercospora musae-Dothideomycetes
KXS97744.1-321146-Mycosphaerella eumusae-Dothideomycetes
OJJ76878.1-767769-Aspergillus brasiliensis-Eurotiomycetes
XP 008599531.1-655819-Beauveria bassiana ARSEF-Sordariomycetes
OGM49724.1-109264-Aspergillus bombycis-Eurotiomycetes
XP 015402055.1-1509407-Aspergillus nomius NRRL 1-Eurotiomycetes
KFY66583.1-1420909-Pseudogymnoascus sp VKM-Leotiomycetes
KFY57765.1-1420910-Pseudogymnoascus sp VKM-Leotiomycetes
KFY39884.1-1420908-Pseudogymnoascus sp VKM-Leotiomycetes
KFX99467.1-1437433-Pseudogymnoascus sp VKM-Leotiomycetes
KFX97535.1-1391699-Pseudogymnoascus sp VKM-Leotiomycetes
XP 013274790.1-1442369-Rhinocladiella mackenziei-Eurotiomycetes
KIW69702.1-5601-Phialophora americana-Eurotiomycetes
XP 008723616.1-1279043-Cladophialophora carrioni-Eurotiomycetes
XP 007755975.1-1182544-Cladophialophora yegresii-Eurotiomycetes
XP 018692325.1-1367422-Fonsecaea erecta-Eurotiomycetes
XP 016636716.1-1442371-Fonsecaea multimorphosa C-Eurotiomycetes
XP 016248755.1-569365-Cladophialophora immunda-Eurotiomycetes
XP 013278546.1-1442368-Fonsecaea pedrosoi CBS 27-Eurotiomycetes
OAG44093.1-254056-Fonsecaea monophora-Eurotiomycetes
XP 016615702.1-1442370-Cladophialophora bantiana-Eurotiomycetes
XP 007748458.1-1182543-Cladophialophora psammoph-Eurotiomycetes
GAD95758.1-1356009-Byssochlamys spectabilis-Eurotiomycetes
OJJ35998.1-1073089-Aspergillus wentii DTO 13-Eurotiomycetes
ODM17037.1-573508-Aspergillus cristatus-Eurotiomycetes
EYE91894.1-1388766-Aspergillus ruber CBS 135-Eurotiomycetes
OJJ79785.1-1160497-Aspergillus glaucus CBS 5-Eurotiomycetes
CEL10067.1-454130-Aspergillus calidoustus-Eurotiomycetes
KKK13230.1-308745-Aspergillus rambellii-Eurotiomycetes
KKK14093.1-138278-Aspergillus ochraceoroseu-Eurotiomycetes
XP 001273686.1-344612-Aspergillus clavatus NRRL-Eurotiomycetes
GAO81288.1-91492-Aspergillus udagawae-Eurotiomycetes
GAQ05142.1-293939-Aspergillus lentulus-Eurotiomycetes
KMK58647.1-1437362-Aspergillus fumigatus Z5-Eurotiomycetes
XP 001258866.1-331117-Aspergillus fischeri NRRL-Eurotiomycetes
OGM42378.1-109264-Aspergillus bombycis-Eurotiomycetes
KJK63873.1-1403190-Aspergillus parasiticus S-Eurotiomycetes
XP 002381708.1-332952-Aspergillus flavus NRRL33-Eurotiomycetes
XP 001825033.2-510516-Aspergillus oryzae RIB40-Eurotiomycetes
OOO04968.1-5062-Aspergillus oryzae-Eurotiomycetes
EIT76281.1-1160506-Aspergillus oryzae 3 042-Eurotiomycetes
BAE63900.1-510516-Aspergillus oryzae RIB40-Eurotiomycetes
OOF94566.1-602072-Aspergillus carbonarius I-Eurotiomycetes
OJJ75218.1-767769-Aspergillus brasiliensis-Eurotiomycetes
EHA26228.1-380704-Aspergillus niger ATCC 10-Eurotiomycetes
XP 001388644.2-425011-Aspergillus niger CBS 513-Eurotiomycetes
OJI87224.1-767770-Aspergillus tubingensis C-Eurotiomycetes
OJZ92782.1-1137211-Aspergillus luchuensis CB-Eurotiomycetes
GAQ33255.1-5061-Aspergillus niger-Eurotiomycetes
OQE46937.1-36646-Penicillium coprophilum-Eurotiomycetes
OQD97567.1-29845-Penicillium vulpinum-Eurotiomycetes
KXG50743.1-5078-Penicillium griseofulvum-Eurotiomycetes
CDM34884.1-1365484-Penicillium roqueforti FM-Eurotiomycetes
KZN89920.1-5076-Penicillium chrysogenum-Eurotiomycetes
OQE15066.1-254877-Penicillium flavigenum-Eurotiomycetes
OQE80984.1-60175-Penicillium nalgiovense-Eurotiomycetes
XP 016592916.1-27334-Penicillium expansum-Eurotiomycetes
CRL25023.1-5075-Penicillium camemberti-Eurotiomycetes
OQD91362.1-60172-Penicillium solitum-Eurotiomycetes
KGO76725.1-40296-Penicillium italicum-Eurotiomycetes
OQD66474.1-60169-Penicillium polonicum-Eurotiomycetes
KUM62120.1-48697-Penicillium freii-Eurotiomycetes

S9B

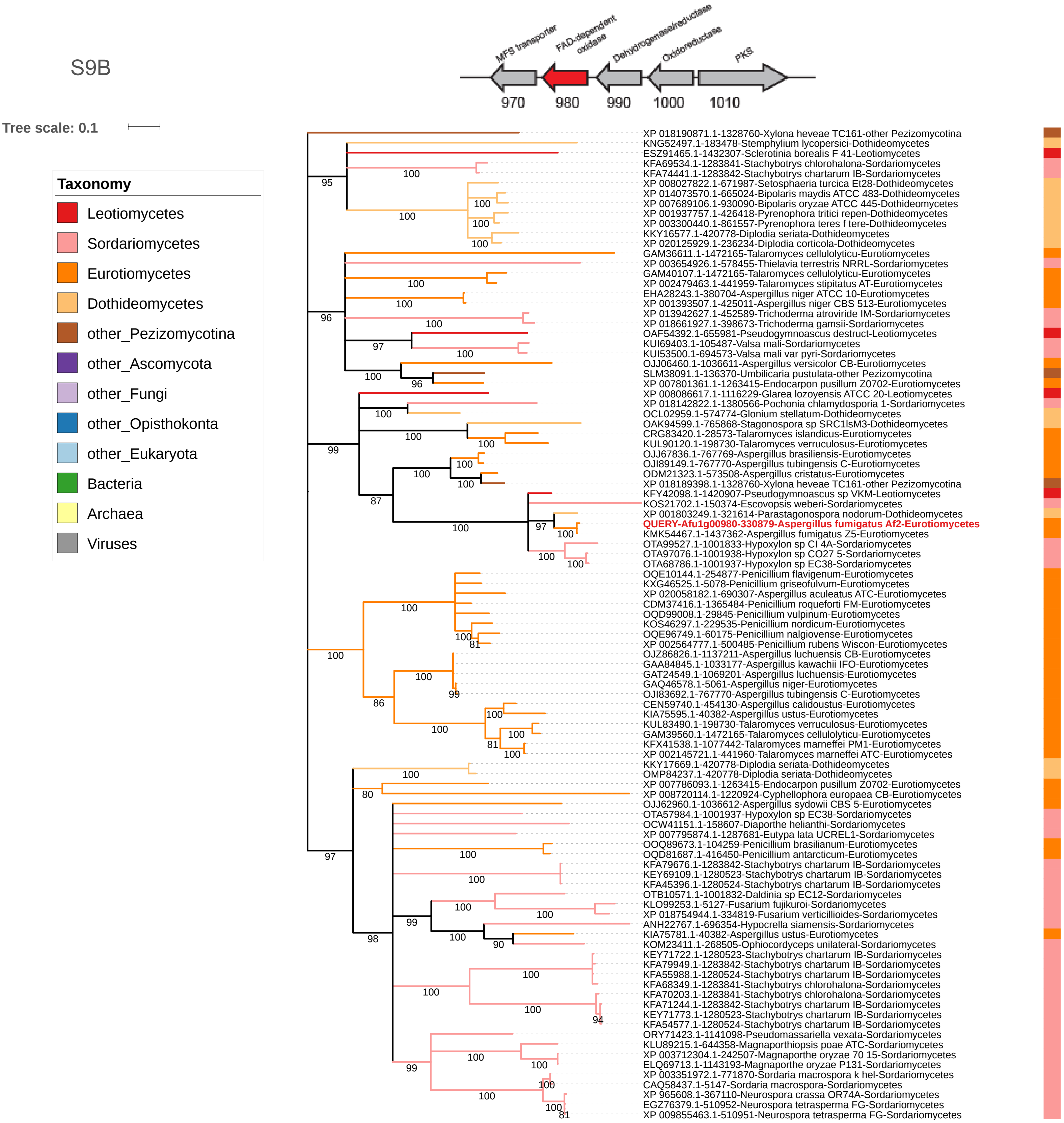
MFS transporter
FAD-dependent oxidase
Dehydrogenase/reductase
Oxidoreductase
PKS
970
980
990
1000
1010
Tree scale: 0.1
Taxonomy
Leotiomycetes
Sordariomycetes
Eurotiomycetes
Dothideomycetes
other_Pezizomycotina
other_Ascomycota
other_Fungi
other_Opisthokonta
other_Eukaryota
Bacteria
Archaea
Viruses
XP 018190871.1-1328760-Xylona heveae TC161-other Pezizomycotina
KNG52497.1-183478-Stemphylium lycopersici-Dothideomycetes
ESZ91465.1-1432307-Sclerotinia borealis F 41-Leotiomycetes
KFA69534.1-1283841-Stachybotrys chlorohalona-Sordariomycetes
KFA74441.1-1283842-Stachybotrys chartarum IB-Sordariomycetes
XP 008027822.1-671987-Setosphaeria turcica Et28-Dothideomycetes
XP 014073570.1-665024-Bipolaris maydis ATCC 483-Dothideomycetes
XP 007689106.1-930090-Bipolaris oryzae ATCC 445-Dothideomycetes
XP 001937757.1-426418-Pyrenophora tritici repen-Dothideomycetes
XP 003300440.1-861557-Pyrenophora teres f tere-Dothideomycetes
KKY16577.1-420778-Diplodia seriata-Dothideomycetes
XP 020125929.1-236234-Diplodia corticola-Dothideomycetes
GAM36611.1-1472165-Talaromyces cellulolyticu-Eurotiomycetes
XP 003654926.1-578455-Thielavia terrestris NRRL-Sordariomycetes
GAM40107.1-1472165-Talaromyces cellulolyticu-Eurotiomycetes
XP 002479463.1-441959-Talaromyces stipitatus AT-Eurotiomycetes
EHA28243.1-380704-Aspergillus niger ATCC 10-Eurotiomycetes
XP 001393507.1-425011-Aspergillus niger CBS 513-Eurotiomycetes
XP 013942627.1-452589-Trichoderma atroviride IM-Sordariomycetes
XP 018661927.1-398673-Trichoderma gamsii-Sordariomycetes
OAF54392.1-655981-Pseudogymnoascus destruct-Leotiomycetes
KUI69403.1-105487-Valsa mali-Sordariomycetes
KUI53500.1-694573-Valsa mali var pyri-Sordariomycetes
OJJ06460.1-1036611-Aspergillus versicolor CB-Eurotiomycetes
SLM38091.1-136370-Umbilicaria pustulata-other Pezizomycotina
XP 007801361.1-1263415-Endocarpon pusillum Z0702-Eurotiomycetes
XP 008086617.1-1116229-Glarea lozoyensis ATCC 20-Leotiomycetes
XP 018142822.1-1380566-Pochonia chlamydosporia 1-Sordariomycetes
OCL02959.1-574774-Glonium stellatum-Dothideomycetes
OAK94599.1-765868-Stagonospora sp SRC1lsM3-Dothideomycetes
CRG83420.1-28573-Talaromyces islandicus-Eurotiomycetes
KUL90120.1-198730-Talaromyces verruculosus-Eurotiomycetes
OJJ67836.1-767769-Aspergillus brasiliensis-Eurotiomycetes
OJI89149.1-767770-Aspergillus tubingensis C-Eurotiomycetes
ODM21323.1-573508-Aspergillus cristatus-Eurotiomycetes
XP 018189398.1-1328760-Xylona heveae TC161-other Pezizomycotina
KFY42098.1-1420907-Pseudogymnoascus sp VKM-Leotiomycetes
KOS21702.1-150374-Escovopsis weberi-Sordariomycetes
XP 001803249.1-321614-Parastagonospora nodorum-Dothideomycetes
QUERY-Afu1g00980-330879-Aspergillus fumigatus Af2-Eurotiomycetes
KMK54467.1-1437362-Aspergillus fumigatus Z5-Eurotiomycetes
OTA99527.1-1001833-Hypoxylon sp CI 4A-Sordariomycetes
OTA97076.1-1001938-Hypoxylon sp CO27 5-Sordariomycetes
OTA68786.1-1001937-Hypoxylon sp EC38-Sordariomycetes
OQE10144.1-254877-Penicillium flavigenum-Eurotiomycetes
KXG46525.1-5078-Penicillium griseofulvum-Eurotiomycetes
XP 020058182.1-690307-Aspergillus aculeatus ATC-Eurotiomycetes
CDM37416.1-1365484-Penicillium roqueforti FM-Eurotiomycetes
OQD99008.1-29845-Penicillium vulpinum-Eurotiomycetes
KOS46297.1-229535-Penicillium nordicum-Eurotiomycetes
OQE96749.1-60175-Penicillium nalgiovense-Eurotiomycetes
XP 002564777.1-500485-Penicillium rubens Wiscon-Eurotiomycetes
OJZ86826.1-1137211-Aspergillus luchuensis CB-Eurotiomycetes
GAA84845.1-1033177-Aspergillus kawachii IFO-Eurotiomycetes
GAT24549.1-1069201-Aspergillus luchuensis-Eurotiomycetes
GAQ46578.1-5061-Aspergillus niger-Eurotiomycetes
OJI83692.1-767770-Aspergillus tubingensis C-Eurotiomycetes
CEN59740.1-454130-Aspergillus calidoustus-Eurotiomycetes
KIA75595.1-40382-Aspergillus ustus-Eurotiomycetes
KUL83490.1-198730-Talaromyces verruculosus-Eurotiomycetes
GAM39560.1-1472165-Talaromyces cellulolyticu-Eurotiomycetes
KFX41538.1-1077442-Talaromyces marneffei PM1-Eurotiomycetes
XP 002145721.1-441960-Talaromyces marneffei ATC-Eurotiomycetes
KKY17669.1-420778-Diplodia seriata-Dothideomycetes
OMP84237.1-420778-Diplodia seriata-Dothideomycetes
XP 007786093.1-1263415-Endocarpon pusillum Z0702-Eurotiomycetes
XP 008720114.1-1220924-Cyphellophora europaea CB-Eurotiomycetes
OJJ62960.1-1036612-Aspergillus sydowii CBS 5-Eurotiomycetes
OTA57984.1-1001937-Hypoxylon sp EC38-Sordariomycetes
OCW41151.1-158607-Diaporthe helianthi-Sordariomycetes
XP 007795874.1-1287681-Eutypa lata UCREL1-Sordariomycetes
OOQ89673.1-104259-Penicillium brasilianum-Eurotiomycetes
OQD81687.1-416450-Penicillium antarcticum-Eurotiomycetes
KFA79676.1-1283842-Stachybotrys chartarum IB-Sordariomycetes
KEY69109.1-1280523-Stachybotrys chartarum IB-Sordariomycetes
KFA45396.1-1280524-Stachybotrys chartarum IB-Sordariomycetes
OTB10571.1-1001832-Daldinia sp EC12-Sordariomycetes
KLO99253.1-5127-Fusarium fujikuroi-Sordariomycetes
XP 018754944.1-334819-Fusarium verticillioides-Sordariomycetes
ANH22767.1-696354-Hypocrella siamensis-Sordariomycetes
KIA75781.1-40382-Aspergillus ustus-Eurotiomycetes
KOM23411.1-268505-Ophiocordyceps unilateral-Sordariomycetes
KEY71722.1-1280523-Stachybotrys chartarum IB-Sordariomycetes
KFA79949.1-1283842-Stachybotrys chartarum IB-Sordariomycetes
KFA55988.1-1280524-Stachybotrys chartarum IB-Sordariomycetes
KFA68349.1-1283841-Stachybotrys chlorohalona-Sordariomycetes
KFA70203.1-1283841-Stachybotrys chlorohalona-Sordariomycetes
KFA71244.1-1283842-Stachybotrys chartarum IB-Sordariomycetes
KEY71773.1-1280523-Stachybotrys chartarum IB-Sordariomycetes
KFA54577.1-1280524-Stachybotrys chartarum IB-Sordariomycetes
ORY71423.1-1141098-Pseudomassariella vexata-Sordariomycetes
KLU89215.1-644358-Magnaporthiopsis poae ATC-Sordariomycetes
XP 003712304.1-242507-Magnaporthe oryzae 70 15-Sordariomycetes
ELQ69713.1-1143193-Magnaporthe oryzae P131-Sordariomycetes
XP 003351972.1-771870-Sordaria macrospora k hel-Sordariomycetes
CAQ58437.1-5147-Sordaria macrospora-Sordariomycetes
XP 965608.1-367110-Neurospora crassa OR74A-Sordariomycetes
EGZ76379.1-510952-Neurospora tetrasperma FG-Sordariomycetes
XP 009855463.1-510951-Neurospora tetrasperma FG-Sordariomycetes

S9C

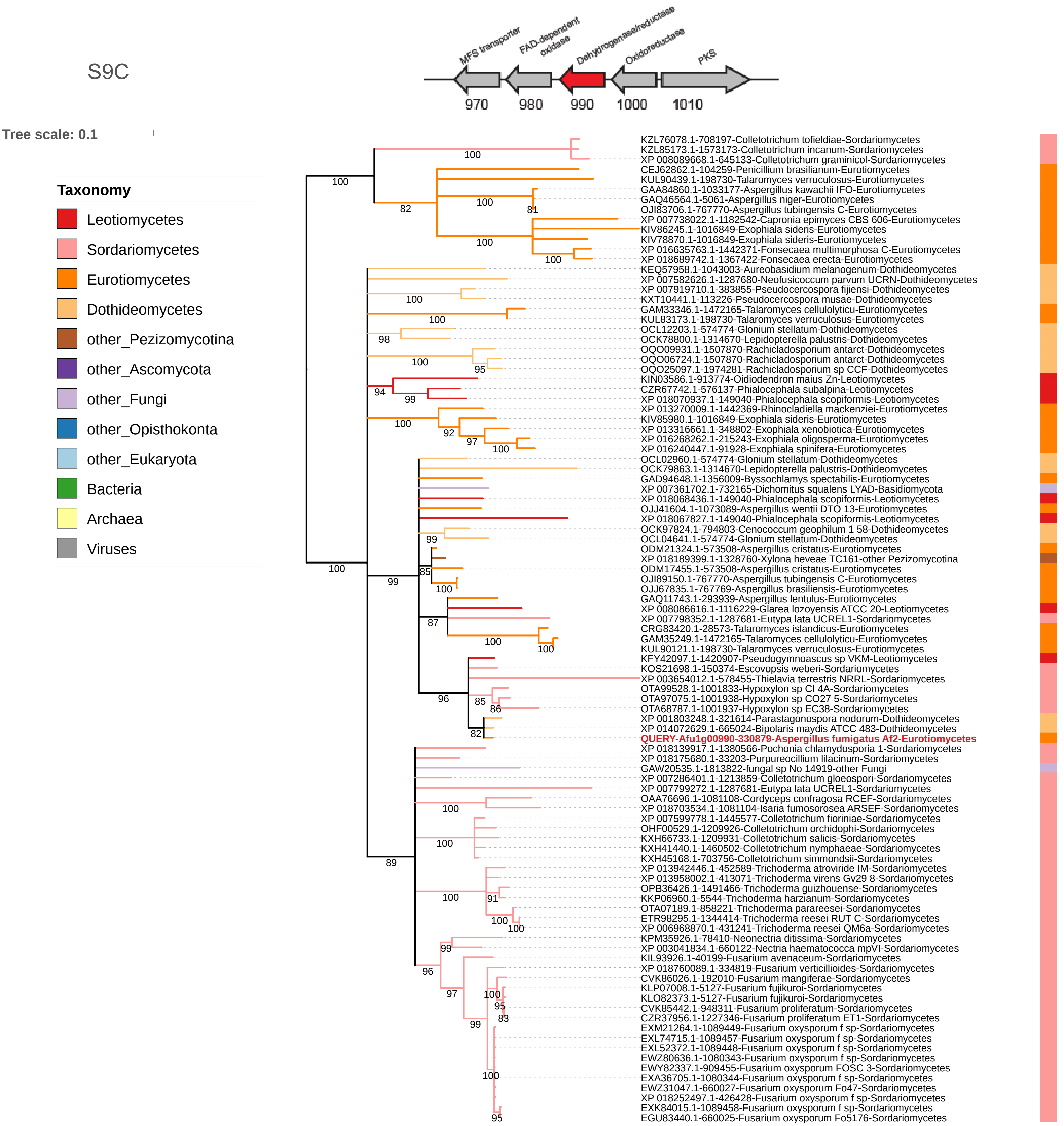
MFS transporter
FAD-dependent oxidase
Dehydrogenase/reductase
Oxidoreductase
PKS
970
980
990
1000
1010
Tree scale: 0.1
Taxonomy
Leotiomycetes
Sordariomycetes
Eurotiomycetes
Dothideomycetes
other_Pezizomycotina
other_Ascomycota
other_Fungi
other_Opisthokonta
other_Eukaryota
Bacteria
Archaea
Viruses
KZL76078.1-708197-Colletotrichum tofieldiae-Sordariomycetes
KZL85173.1-1573173-Colletotrichum incanum-Sordariomycetes
XP 008089668.1-645133-Colletotrichum graminicol-Sordariomycetes
CEJ62862.1-104259-Penicillium brasilianum-Eurotiomycetes
KUL90439.1-198730-Talaromyces verruculosus-Eurotiomycetes
GAA84860.1-1033177-Aspergillus kawachii IFO-Eurotiomycetes
GAQ46564.1-5061-Aspergillus niger-Eurotiomycetes
OJI83706.1-767770-Aspergillus tubingensis C-Eurotiomycetes
XP 007738022.1-1182542-Capronia epimyces CBS 606-Eurotiomycetes
KIV86245.1-1016849-Exophiala sideris-Eurotiomycetes
KIV78870.1-1016849-Exophiala sideris-Eurotiomycetes
XP 016635763.1-1442371-Fonsecaea multimorphosa C-Eurotiomycetes
XP 018689742.1-1367422-Fonsecaea erecta-Eurotiomycetes
KEQ57958.1-1043003-Aureobasidium melanogenum-Dothideomycetes
XP 007582626.1-1287680-Neofusicoccum parvum UCRN-Dothideomycetes
XP 007919710.1-383855-Pseudocercospora fijiensi-Dothideomycetes
KXT10441.1-113226-Pseudocercospora musae-Dothideomycetes
GAM33346.1-1472165-Talaromyces cellulolyticu-Eurotiomycetes
KUL83173.1-198730-Talaromyces verruculosus-Eurotiomycetes
OCL12203.1-574774-Glonium stellatum-Dothideomycetes
OCK78800.1-1314670-Lepidopterella palustris-Dothideomycetes
OQO09931.1-1507870-Rachicladosporium antarct-Dothideomycetes
OQO06724.1-1507870-Rachicladosporium antarct-Dothideomycetes
OQO25097.1-1974281-Rachicladosporium sp CCF-Dothideomycetes
KIN03586.1-913774-Oidiodendron maius Zn-Leotiomycetes
CZR67742.1-576137-Phialocephala subalpina-Leotiomycetes
XP 018070937.1-149040-Phialocephala scopiformis-Leotiomycetes
XP 013270009.1-1442369-Rhinocladiella mackenziei-Eurotiomycetes
KIV85980.1-1016849-Exophiala sideris-Eurotiomycetes
XP 013316661.1-348802-Exophiala xenobiotica-Eurotiomycetes
XP 016268262.1-215243-Exophiala oligosperma-Eurotiomycetes
XP 016240447.1-91928-Exophiala spinifera-Eurotiomycetes
OCL02960.1-574774-Glonium stellatum-Dothideomycetes
OCK79863.1-1314670-Lepidopterella palustris-Dothideomycetes
GAD94648.1-1356009-Byssochlamys spectabilis-Eurotiomycetes
XP 007361702.1-732165-Dichomitus squalens LYAD-Basidiomycota
XP 018068436.1-149040-Phialocephala scopiformis-Leotiomycetes
OJJ41604.1-1073089-Aspergillus wentii DTO 13-Eurotiomycetes
XP 018067827.1-149040-Phialocephala scopiformis-Leotiomycetes
OCK97824.1-794803-Cenococcum geophilum 1 58-Dothideomycetes
OCL04641.1-574774-Glonium stellatum-Dothideomycetes
ODM21324.1-573508-Aspergillus cristatus-Eurotiomycetes
XP 018189399.1-1328760-Xylona heveae TC161-other Pezizomycotina
ODM17455.1-573508-Aspergillus cristatus-Eurotiomycetes
OJI89150.1-767770-Aspergillus tubingensis C-Eurotiomycetes
OJJ67835.1-767769-Aspergillus brasiliensis-Eurotiomycetes
GAQ11743.1-293939-Aspergillus lentulus-Eurotiomycetes
XP 008086616.1-1116229-Glarea lozoyensis ATCC 20-Leotiomycetes
XP 007798352.1-1287681-Eutypa lata UCREL1-Sordariomycetes
CRG83420.1-28573-Talaromyces islandicus-Eurotiomycetes
GAM35249.1-1472165-Talaromyces cellulolyticu-Eurotiomycetes
KUL90121.1-198730-Talaromyces verruculosus-Eurotiomycetes
KFY42097.1-1420907-Pseudogymnoascus sp VKM-Leotiomycetes
KOS21698.1-150374-Escovopsis weberi-Sordariomycetes
XP 003654012.1-578455-Thielavia terrestris NRRL-Sordariomycetes
OTA99528.1-1001833-Hypoxylon sp CI 4A-Sordariomycetes
OTA97075.1-1001938-Hypoxylon sp CO27 5-Sordariomycetes
OTA68787.1-1001937-Hypoxylon sp EC38-Sordariomycetes
XP 001803248.1-321614-Parastagonospora nodorum-Dothideomycetes
XP 014072629.1-665024-Bipolaris maydis ATCC 483-Dothideomycetes
QUERY-Afu1g00990-330879-Aspergillus fumigatus Af2-Eurotiomycetes
XP 018139917.1-1380566-Pochonia chlamydosporia 1-Sordariomycetes
XP 018175680.1-33203-Purpureocillium lilacinum-Sordariomycetes
GAW20535.1-1813822-fungal sp No 14919-other Fungi
XP 007286401.1-1213859-Colletotrichum gloeospori-Sordariomycetes
XP 007799272.1-1287681-Eutypa lata UCREL1-Sordariomycetes
OAA76696.1-1081108-Cordyceps confragosa RCEF-Sordariomycetes
XP 018703534.1-1081104-Isaria fumosorosea ARSEF-Sordariomycetes
XP 007599778.1-1445577-Colletotrichum fioriniae-Sordariomycetes
OHF00529.1-1209926-Colletotrichum orchidophi-Sordariomycetes
KXH66733.1-1209931-Colletotrichum salicis-Sordariomycetes
KXH41440.1-1460502-Colletotrichum nymphaeae-Sordariomycetes
KXH45168.1-703756-Colletotrichum simmondsii-Sordariomycetes
XP 013942446.1-452589-Trichoderma atroviride IM-Sordariomycetes
XP 013958002.1-413071-Trichoderma virens Gv29 8-Sordariomycetes
OPB36426.1-1491466-Trichoderma guizhouense-Sordariomycetes
KKP06960.1-5544-Trichoderma harzianum-Sordariomycetes
OTA07189.1-858221-Trichoderma parareesei-Sordariomycetes
ETR98295.1-1344414-Trichoderma reesei RUT C-Sordariomycetes
XP 006968870.1-431241-Trichoderma reesei QM6a-Sordariomycetes
KPM35926.1-78410-Neonectria ditissima-Sordariomycetes
XP 003041834.1-660122-Nectria haematococca mpVI-Sordariomycetes
KIL93926.1-40199-Fusarium avenaceum-Sordariomycetes
XP 018760089.1-334819-Fusarium verticillioides-Sordariomycetes
CVK86026.1-192010-Fusarium mangiferae-Sordariomycetes
KLP07008.1-5127-Fusarium fujikuroi-Sordariomycetes
KLO82373.1-5127-Fusarium fujikuroi-Sordariomycetes
CVK85442.1-948311-Fusarium proliferatum-Sordariomycetes
CZR37956.1-1227346-Fusarium proliferatum ET1-Sordariomycetes
EXM21264.1-1089449-Fusarium oxysporum f sp-Sordariomycetes
EXL74715.1-1089457-Fusarium oxysporum f sp-Sordariomycetes
EXL52372.1-1089448-Fusarium oxysporum f sp-Sordariomycetes
EWZ80636.1-1080343-Fusarium oxysporum f sp-Sordariomycetes
EWY82337.1-909455-Fusarium oxysporum FOSC 3-Sordariomycetes
EXA36705.1-1080344-Fusarium oxysporum f sp-Sordariomycetes
EWZ31047.1-660027-Fusarium oxysporum Fo47-Sordariomycetes
XP 018252497.1-426428-Fusarium oxysporum f sp-Sordariomycetes
EXK84015.1-1089458-Fusarium oxysporum f sp-Sordariomycetes
EGU83440.1-660025-Fusarium oxysporum Fo5176-Sordariomycetes

S9D

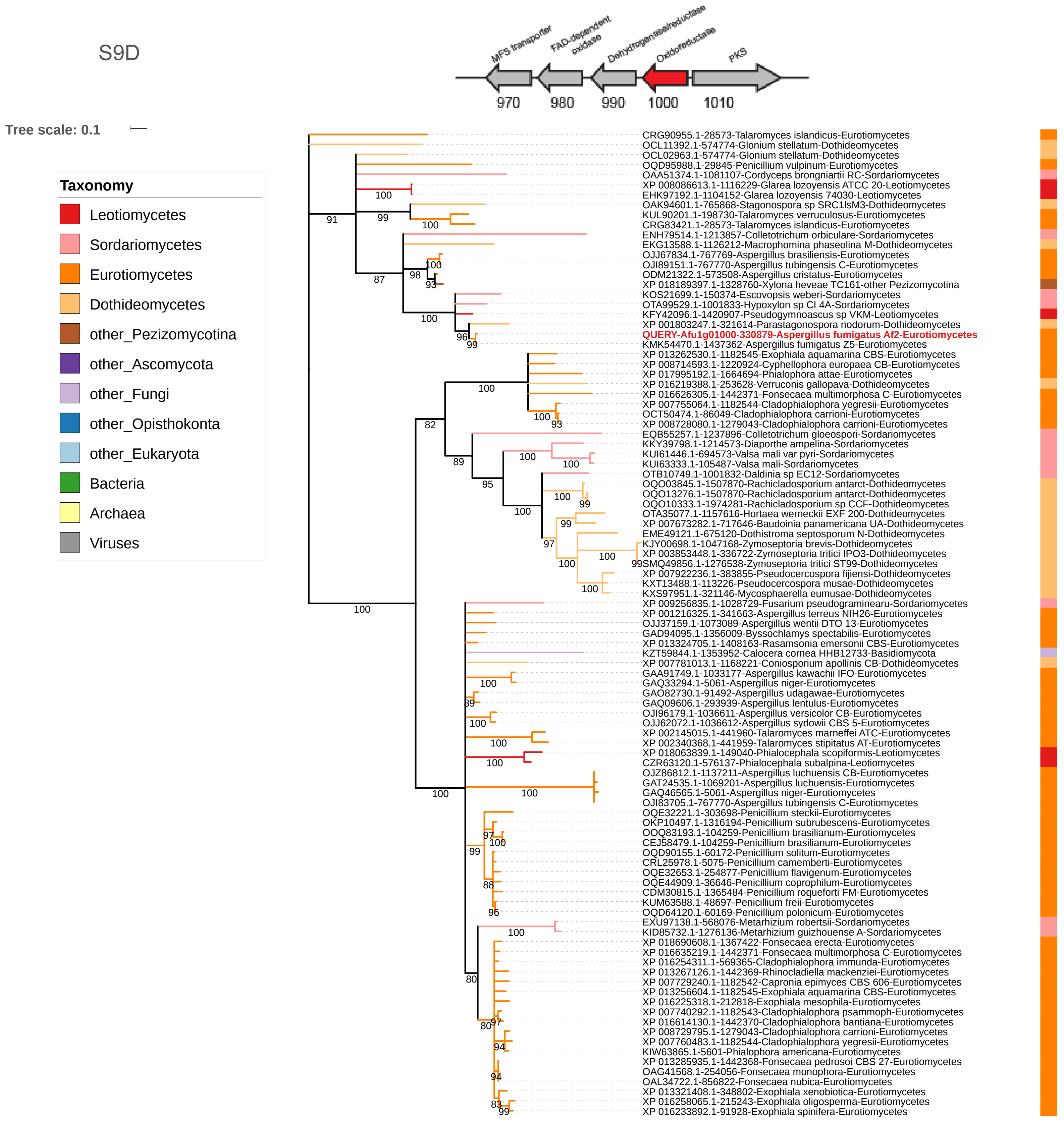
MFS transporter
FAD-dependent oxidase
Dehydrogenase/reductase
Oxidoreductase
PKS
970
980
990
1000
1010
Tree scale: 0.1
Taxonomy
Leotiomycetes
Sordariomycetes
Eurotiomycetes
Dothideomycetes
other_Pezizomycotina
other_Ascomycota
other_Fungi
other_Opisthokonta
other_Eukaryota
Bacteria
Archaea
Viruses
CRG90955.1-28573-Talaromyces islandicus-Eurotiomycetes
OCL11392.1-574774-Glonium stellatum-Dothideomycetes
OCL02963.1-574774-Glonium stellatum-Dothideomycetes
OQD95988.1-29845-Penicillium vulpinum-Eurotiomycetes
OAA51374.1-1081107-Cordyceps brongniartii RC-Sordariomycetes
XP 008086613.1-1116229-Glarea lozoyensis ATCC 20-Leotiomycetes
EHK97192.1-1104152-Glarea lozoyensis 74030-Leotiomycetes
OAK94601.1-765868-Stagonospora sp SRC1lsM3-Dothideomycetes
KUL90201.1-198730-Talaromyces verruculosus-Eurotiomycetes
CRG83421.1-28573-Talaromyces islandicus-Eurotiomycetes
ENH79514.1-1213857-Colletotrichum orbiculare-Sordariomycetes
EKG13588.1-1126212-Macrophomina phaseolina M-Dothideomycetes
OJJ67834.1-767769-Aspergillus brasiliensis-Eurotiomycetes
OJI89151.1-767770-Aspergillus tubingensis C-Eurotiomycetes
ODM21322.1-573508-Aspergillus cristatus-Eurotiomycetes
XP 018189397.1-1328760-Xylona heveae TC161-other Pezizomycotina
KOS21699.1-150374-Escovopsis weberi-Sordariomycetes
OTA99529.1-1001833-Hypoxylon sp CI 4A-Sordariomycetes
KFY42096.1-1420907-Pseudogymnoascus sp VKM-Leotiomycetes
XP 001803247.1-321614-Parastagonospora nodorum-Dothideomycetes
QUERY-Afu1g01000-330879-Aspergillus fumigatus Af2-Eurotiomycetes
KMK54470.1-1437362-Aspergillus fumigatus Z5-Eurotiomycetes
XP 013262530.1-1182545-Exophiala aquamarina CBS-Eurotiomycetes
XP 008714593.1-1220924-Cyphellophora europaea CB-Eurotiomycetes
XP 017995192.1-1664694-Phialophora attae-Eurotiomycetes
XP 016219388.1-253628-Verruconis gallopava-Dothideomycetes
XP 016626305.1-1442371-Fonsecaea multimorphosa C-Eurotiomycetes
XP 007755064.1-1182544-Cladophialophora yegresii-Eurotiomycetes
OCT50474.1-86049-Cladophialophora carrioni-Eurotiomycetes
XP 008728080.1-1279043-Cladophialophora carrioni-Eurotiomycetes
EQB55257.1-1237896-Colletotrichum gloeospori-Sordariomycetes
KKY39798.1-1214573-Diaporthe ampelina-Sordariomycetes
KUI61446.1-694573-Valsa mali var pyri-Sordariomycetes
KUI63333.1-105487-Valsa mali-Sordariomycetes
OTB10749.1-1001832-Daldinia sp EC12-Sordariomycetes
OQO03845.1-1507870-Rachicladosporium antarct-Dothideomycetes
OQO13276.1-1507870-Rachicladosporium antarct-Dothideomycetes
OQO10333.1-1974281-Rachicladosporium sp CCF-Dothideomycetes
OTA35077.1-1157616-Hortaea werneckii EXF 200-Dothideomycetes
XP 007673282.1-717646-Baudoinia panamericana UA-Dothideomycetes
EME49121.1-675120-Dothistroma septosporum N-Dothideomycetes
KJY00698.1-1047168-Zymoseptoria brevis-Dothideomycetes
XP 003853448.1-336722-Zymoseptoria tritici IPO3-Dothideomycetes
SMQ49856.1-1276538-Zymoseptoria tritici ST99-Dothideomycetes
XP 007922236.1-383855-Pseudocercospora fijiensi-Dothideomycetes
KXT13488.1-113226-Pseudocercospora musae-Dothideomycetes
KXS97951.1-321146-Mycosphaerella eumusae-Dothideomycetes
XP 009256835.1-1028729-Fusarium pseudograminearu-Sordariomycetes
XP 001216325.1-341663-Aspergillus terreus NIH26-Eurotiomycetes
OJJ37159.1-1073089-Aspergillus wentii DTO 13-Eurotiomycetes
GAD94095.1-1356009-Byssochlamys spectabilis-Eurotiomycetes
XP 013324705.1-1408163-Rasamsonia emersonii CBS-Eurotiomycetes
KZT59844.1-1353952-Calocera cornea HHB12733-Basidiomycota
XP 007781013.1-1168221-Coniosporium apollinis CB-Dothideomycetes
GAA91749.1-1033177-Aspergillus kawachii IFO-Eurotiomycetes
GAQ33294.1-5061-Aspergillus niger-Eurotiomycetes
GAO82730.1-91492-Aspergillus udagawae-Eurotiomycetes
GAQ09606.1-293939-Aspergillus lentulus-Eurotiomycetes
OJI96179.1-1036611-Aspergillus versicolor CB-Eurotiomycetes
OJJ62072.1-1036612-Aspergillus sydowii CBS 5-Eurotiomycetes
XP 002145015.1-441960-Talaromyces marneffei ATC-Eurotiomycetes
XP 002340368.1-441959-Talaromyces stipitatus AT-Eurotiomycetes
XP 018063839.1-149040-Phialocephala scopiformis-Leotiomycetes
CZR63120.1-576137-Phialocephala subalpina-Leotiomycetes
OJZ86812.1-1137211-Aspergillus luchuensis CB-Eurotiomycetes
GAT24535.1-1069201-Aspergillus luchuensis-Eurotiomycetes
GAQ46565.1-5061-Aspergillus niger-Eurotiomycetes
OJI83705.1-767770-Aspergillus tubingensis C-Eurotiomycetes
OQE32221.1-303698-Penicillium steckii-Eurotiomycetes
OKP10497.1-1316194-Penicillium subrubescens-Eurotiomycetes
OOQ83193.1-104259-Penicillium brasilianum-Eurotiomycetes
CEJ58479.1-104259-Penicillium brasilianum-Eurotiomycetes
OQD90155.1-60172-Penicillium solitum-Eurotiomycetes
CRL25978.1-5075-Penicillium camemberti-Eurotiomycetes
OQE32653.1-254877-Penicillium flavigenum-Eurotiomycetes
OQE44909.1-36646-Penicillium coprophilum-Eurotiomycetes
CDM30815.1-1365484-Penicillium roqueforti FM-Eurotiomycetes
KUM63588.1-48697-Penicillium freii-Eurotiomycetes
OQD64120.1-60169-Penicillium polonicum-Eurotiomycetes
EXU97138.1-568076-Metarhizium robertsii-Sordariomycetes
KID85732.1-1276136-Metarhizium guizhouense A-Sordariomycetes
XP 018690608.1-1367422-Fonsecaea erecta-Eurotiomycetes
XP 016635219.1-1442371-Fonsecaea multimorphosa C-Eurotiomycetes
XP 016254311.1-569365-Cladophialophora immunda-Eurotiomycetes
XP 013267126.1-1442369-Rhinocladiella mackenziei-Eurotiomycetes
XP 007729240.1-1182542-Capronia epimyces CBS 606-Eurotiomycetes
XP 013256604.1-1182545-Exophiala aquamarina CBS-Eurotiomycetes
XP 016225318.1-212818-Exophiala mesophila-Eurotiomycetes
XP 007740292.1-1182543-Cladophialophora psammoph-Eurotiomycetes
XP 016614130.1-1442370-Cladophialophora bantiana-Eurotiomycetes
XP 008729795.1-1279043-Cladophialophora carrioni-Eurotiomycetes
XP 007760483.1-1182544-Cladophialophora yegresii-Eurotiomycetes
KIW63865.1-5601-Phialophora americana-Eurotiomycetes
XP 013285935.1-1442368-Fonsecaea pedrosoi CBS 27-Eurotiomycetes
OAG41568.1-254056-Fonsecaea monophora-Eurotiomycetes
OAL34722.1-856822-Fonsecaea nubica-Eurotiomycetes
XP 013321408.1-348802-Exophiala xenobiotica-Eurotiomycetes
XP 016258065.1-215243-Exophiala oligosperma-Eurotiomycetes
XP 016233892.1-91928-Exophiala spinifera-Eurotiomycetes

S9E

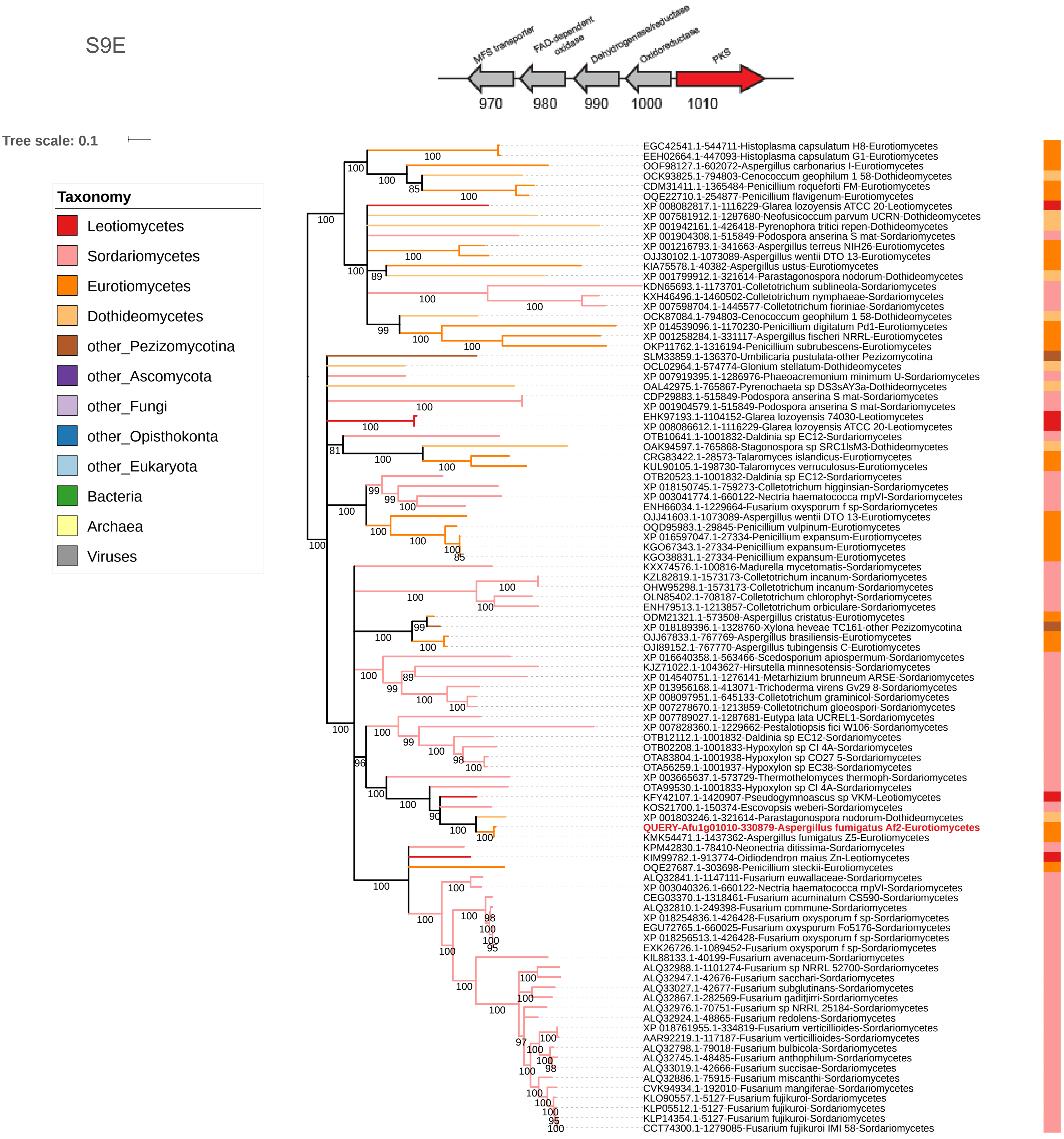
MFS transporter
FAD-dependent oxidase
Dehydrogenase/reductase
Oxidoreductase
PKS
970
980
990
1000
1010
Tree scale: 0.1
Taxonomy
Leotiomycetes
Sordariomycetes
Eurotiomycetes
Dothideomycetes
other_Pezizomycotina
other_Ascomycota
other_Fungi
other_Opisthokonta
other_Eukaryota
Bacteria
Archaea
Viruses
EGC42541.1-544711-Histoplasma capsulatum H8-Eurotiomycetes
EEH02664.1-447093-Histoplasma capsulatum G1-Eurotiomycetes
OOF98127.1-602072-Aspergillus carbonarius I-Eurotiomycetes
OCK93825.1-794803-Cenococcum geophilum 1 58-Dothideomycetes
CDM31411.1-1365484-Penicillium roqueforti FM-Eurotiomycetes
OQE22710.1-254877-Penicillium flavigenum-Eurotiomycetes
XP 008082817.1-1116229-Glarea lozoyensis ATCC 20-Leotiomycetes
XP 007581912.1-1287680-Neofusicoccum parvum UCRN-Dothideomycetes
XP 001942161.1-426418-Pyrenophora tritici repen-Dothideomycetes
XP 001904308.1-515849-Podospora anserina S mat-Sordariomycetes
XP 001216793.1-341663-Aspergillus terreus NIH26-Eurotiomycetes
OJJ30102.1-1073089-Aspergillus wentii DTO 13-Eurotiomycetes
KIA75578.1-40382-Aspergillus ustus-Eurotiomycetes
XP 001799912.1-321614-Parastagonospora nodorum-Dothideomycetes
KDN65693.1-1173701-Colletotrichum sublineola-Sordariomycetes
KXH46496.1-1460502-Colletotrichum nymphaeae-Sordariomycetes
XP 007598704.1-1445577-Colletotrichum fioriniae-Sordariomycetes
OCK87084.1-794803-Cenococcum geophilum 1 58-Dothideomycetes
XP 014539096.1-1170230-Penicillium digitatum Pd1-Eurotiomycetes
XP 001258284.1-331117-Aspergillus fischeri NRRL-Eurotiomycetes
OKP11762.1-1316194-Penicillium subrubescens-Eurotiomycetes
SLM33859.1-136370-Umbilicaria pustulata-other Pezizomycotina
OCL02964.1-574774-Glonium stellatum-Dothideomycetes
XP 007919395.1-1286976-Phaeoacremonium minimum U-Sordariomycetes
OAL42975.1-765867-Pyrenochaeta sp DS3sAY3a-Dothideomycetes
CDP29883.1-515849-Podospora anserina S mat-Sordariomycetes
XP 001904579.1-515849-Podospora anserina S mat-Sordariomycetes
EHK97193.1-1104152-Glarea lozoyensis 74030-Leotiomycetes
XP 008086612.1-1116229-Glarea lozoyensis ATCC 20-Leotiomycetes
OTB10641.1-1001832-Daldinia sp EC12-Sordariomycetes
OAK94597.1-765868-Stagonospora sp SRC1lsM3-Dothideomycetes
CRG83422.1-28573-Talaromyces islandicus-Eurotiomycetes
KUL90105.1-198730-Talaromyces verruculosus-Eurotiomycetes
OTB20523.1-1001832-Daldinia sp EC12-Sordariomycetes
XP 018150745.1-759273-Colletotrichum higginsian-Sordariomycetes
XP 003041774.1-660122-Nectria haematococca mpVI-Sordariomycetes
ENH66034.1-1229664-Fusarium oxysporum f sp-Sordariomycetes
OJJ41603.1-1073089-Aspergillus wentii DTO 13-Eurotiomycetes
OQD95983.1-29845-Penicillium vulpinum-Eurotiomycetes
XP 016597047.1-27334-Penicillium expansum-Eurotiomycetes
KGO67343.1-27334-Penicillium expansum-Eurotiomycetes
KGO38831.1-27334-Penicillium expansum-Eurotiomycetes
KXX74576.1-100816-Madurella mycetomatis-Sordariomycetes
KZL82819.1-1573173-Colletotrichum incanum-Sordariomycetes
OHW95298.1-1573173-Colletotrichum incanum-Sordariomycetes
OLN85402.1-708187-Colletotrichum chlorophyt-Sordariomycetes
ENH79513.1-1213857-Colletotrichum orbiculare-Sordariomycetes
ODM21321.1-573508-Aspergillus cristatus-Eurotiomycetes
XP 018189396.1-1328760-Xylona heveae TC161-other Pezizomycotina
OJJ67833.1-767769-Aspergillus brasiliensis-Eurotiomycetes
OJI89152.1-767770-Aspergillus tubingensis C-Eurotiomycetes
XP 016640358.1-563466-Scedosporium apiospermum-Sordariomycetes
KJZ71022.1-1043627-Hirsutella minnesotensis-Sordariomycetes
XP 014540751.1-1276141-Metarhizium brunneum ARSE-Sordariomycetes
XP 013956168.1-413071-Trichoderma virens Gv29 8-Sordariomycetes
XP 008097951.1-645133-Colletotrichum graminicol-Sordariomycetes
XP 007278670.1-1213859-Colletotrichum gloeospori-Sordariomycetes
XP 007789027.1-1287681-Eutypa lata UCREL1-Sordariomycetes
XP 007828360.1-1229662-Pestalotiopsis fici W106-Sordariomycetes
OTB12112.1-1001832-Daldinia sp EC12-Sordariomycetes
OTB02208.1-1001833-Hypoxylon sp CI 4A-Sordariomycetes
OTA83804.1-1001938-Hypoxylon sp CO27 5-Sordariomycetes
OTA56259.1-1001937-Hypoxylon sp EC38-Sordariomycetes
XP 003665637.1-573729-Thermothelomyces thermoph-Sordariomycetes
OTA99530.1-1001833-Hypoxylon sp CI 4A-Sordariomycetes
KFY42107.1-1420907-Pseudogymnoascus sp VKM-Leotiomycetes
KOS21700.1-150374-Escovopsis weberi-Sordariomycetes
XP 001803246.1-321614-Parastagonospora nodorum-Dothideomycetes
QUERY-Afu1g01010-330879-Aspergillus fumigatus Af2-Eurotiomycetes
KMK54471.1-1437362-Aspergillus fumigatus Z5-Eurotiomycetes
KPM42830.1-78410-Neonectria ditissima-Sordariomycetes
KIM99782.1-913774-Oidiodendron maius Zn-Leotiomycetes
OQE27687.1-303698-Penicillium steckii-Eurotiomycetes
ALQ32841.1-1147111-Fusarium euwallaceae-Sordariomycetes
XP 003040326.1-660122-Nectria haematococca mpVI-Sordariomycetes
CEG03370.1-1318461-Fusarium acuminatum CS590-Sordariomycetes
ALQ32810.1-249398-Fusarium commune-Sordariomycetes
XP 018254836.1-426428-Fusarium oxysporum f sp-Sordariomycetes
EGU72765.1-660025-Fusarium oxysporum Fo5176-Sordariomycetes
XP 018256513.1-426428-Fusarium oxysporum f sp-Sordariomycetes
EXK26726.1-1089452-Fusarium oxysporum f sp-Sordariomycetes
KIL88133.1-40199-Fusarium avenaceum-Sordariomycetes
ALQ32988.1-1101274-Fusarium sp NRRL 52700-Sordariomycetes
ALQ32947.1-42676-Fusarium sacchari-Sordariomycetes
ALQ33027.1-42677-Fusarium subglutinans-Sordariomycetes
ALQ32867.1-282569-Fusarium gaditjirri-Sordariomycetes
ALQ32976.1-70751-Fusarium sp NRRL 25184-Sordariomycetes
ALQ32924.1-48865-Fusarium redolens-Sordariomycetes
XP 018761955.1-334819-Fusarium verticillioides-Sordariomycetes
AAR92219.1-117187-Fusarium verticillioides-Sordariomycetes
ALQ32798.1-79018-Fusarium bulbicola-Sordariomycetes
ALQ32745.1-48485-Fusarium anthophilum-Sordariomycetes
ALQ33019.1-42666-Fusarium succisae-Sordariomycetes
ALQ32886.1-75915-Fusarium miscanthi-Sordariomycetes
CVK94934.1-192010-Fusarium mangiferae-Sordariomycetes
KLO90557.1-5127-Fusarium fujikuroi-Sordariomycetes
KLP05512.1-5127-Fusarium fujikuroi-Sordariomycetes
KLP14354.1-5127-Fusarium fujikuroi-Sordariomycetes
CCT74300.1-1279085-Fusarium fujikuroi IMI 58-Sordariomycetes
